# Stigma and Quality of Life in Women With Breast Cancer: Mediation and Moderation Model of Social Support, Sense of Coherence, and Coping Strategies

**DOI:** 10.3389/fpsyg.2022.657992

**Published:** 2022-02-14

**Authors:** Hadi Zamanian, Mohammadali Amini-Tehrani, Zahra Jalali, Mona Daryaafzoon, Fatemeh Ramezani, Negin Malek, Maede Adabimohazab, Roghayeh Hozouri, Fereshteh Rafiei Taghanaky

**Affiliations:** ^1^Department of Health Education and Promotion, School of Health, Qom University of Medical Sciences, Qom, Iran; ^2^Health Psychology and Behavior Medicine Research Group, Students’ Scientific Research Center, Tehran University of Medical Sciences, Tehran, Iran; ^3^Department of Psychology, Faculty of Psychology and Education, University of Tehran, Tehran, Iran; ^4^Connective Tissue Diseases Research Center, Tabriz University of Medical Sciences, Tabriz, Iran; ^5^Department of Psychology, Karaj Branch, Islamic Azad University, Karaj, Iran

**Keywords:** psych-oncology, stigma, sense of coherence, social support, coping, quality of life, breast cancer

## Abstract

**Objectives:**

The breast cancer stigma affects Health-related quality of life (HRQoL), while general resilience resources (GRRs), namely, sense of coherence (SOC), social support, and coping skills, are thought to alleviate this effect. The study aimed to explore the mediating/moderation role of GRRs in the relationship between stigma and HRQoL and its dimensions in Iranian patients with breast cancer.

**Methods:**

In this cross-sectional study, Stigma Scale for Chronic Illness 8-item version (SSCI-8), SOC-13, Medical Outcome Survey- Social Support Scale (MOS-SSS), Brief COPE, and Functional Assessment of Cancer Therapy-Breast (FACT-B) were investigated in a convenience sample of Iranian women with confirmed non-metastatic breast cancer. Following the establishment of correlations using Pearson’s correlation, single and parallel mediation analysis and moderation analysis were conducted to determine the extent to which each GRR might be impacted by stigma or decrease the adverse impact of stigma on HRQoL.

**Results:**

An analysis of 221 women (response rate of 87.5%) with the mean age of 47.14 (9.13) showed that stigma was negatively correlated to all HRQoL’s dimensions (*r* = −0.27∼0.51, *p* < 0.05), SOC (*r* = −0.26∼0.35, *p* < 0.01), social support (*r* = −0.23∼0.30, *p* < 0.01), and the bulk of coping skills. In the single mediation analysis, stigma affected all facets of SOC, all subscales of social support, and positive reframing, which partially reduced breast cancer HRQoL. Stigma affects general HRQoL through damaging meaningfulness, social support (except for tangible), and positive reframing. Meaningfulness was marked as the most impacted GRR in terms of all domains of HRQoL. In parallel mediation, reduced meaningfulness, total social support, and positive reframing were highlighted as the pathways of diminished breast cancer HRQoL. Moderation analysis indicated the higher levels of humor, behavioral disengagement, and use of instrumental support behaviors to be functional in protecting different dimensions of HRQoL, while the results were mixed for venting, especially in patients with mastectomy surgery.

**Conclusion:**

While GRRs may be impacted by stigma, they exert a relatively small protective effect against the impact of stigma on HRQoL. This study provides some novel findings, but longitudinal studies are needed to further verify these before any causal conclusion or recommendations for health policy can be drawn.

## Introduction

Breast cancer is the most common cancer of women worldwide (Bray et al., 2018). Owing to the recent advances in screening and treatment, breast cancer mortality has significantly decreased (Van Schoor et al., 2011; Narod et al., 2015), and resultantly, breast cancer has transformed from a lethal to a physically and psychologically disabling condition (DeSantis et al., 2019). Thus, patients’ quality of life has become a prominent factor in their course of survival.

Health-related quality of life (HRQoL) addresses the impact of chronic illnesses and treatments on patients’ physical, emotional, and social functioning (de Wit and Hajos, 2013; Post, 2014). Patients with better access to palliative care and support groups have reported higher levels of HRQoL (Avis et al., 2005). Additionally, social and cultural contexts largely impact HRQoL (Wilson and Cleary, 1995; Gerasimčik-Pulko et al., 2009). In Iran, where women with breast cancer have reported a moderate HRQoL (Salehoddin Bouya et al., 2018), there is rising concern about the stigmatization of the condition in patients and the public (Khakbazan et al., 2014; Najmabadi et al., 2014; Daryaafzoon et al., 2020; Amini-Tehrani et al., 2021).

Cancers carry a stigma, which is the negative attitude of society toward patients and prevents them from healthy coping (Wang et al., 2017). Cancer stigma originates from individuals’ fear of being reminded of their vulnerability to developing a fatal disease, abhorring the physical alterations, and trying to cope with their shattered view of the just-world hypothesis, which assumes that the world is a fair place where individuals get what they deserve, so good things happen to good people and vice versa (Butts Stahly, 1989; Else-Quest and Jackson, 2014). Enacted stigma refers to the experiences of discrimination, while internalized stigma is about the cognitive absorption of devaluations (Gray, 2002). The latter induces negative emotions of self-blame and shame, which in turn makes them engage in maladaptive behaviors such as social withdrawal, reduced help-seeking, and therapy refusal which results in diminished HRQoL (Phelan et al., 2013; Nyblade et al., 2017).

Depending on the cancer type and cultural characteristics, the prevalence of perceived stigmatization greatly varies, ranging from 13 to 80% (Cho et al., 2013; Yılmaz et al., 2019). About 26% of Iranian patients with cancer are psychologically stressed out by stigma (Hasan Shiri et al., 2018). Breast cancer may fall into the category of less stigmatized cancers in the sense that, unlike cancer types that are commonly believed to be controllable (e.g., lung cancer in smokers), it has no recognized modifiable risk factor (Sarah Knapp et al., 2014). However, disfiguring resections may harm the body image, social interactions, and the overall HRQoL of patients with breast cancer (Waljee et al., 2011). Nonetheless, the stigma associated with breast cancer impairs the patients’ HRQoL (Ernst et al., 2017).

Of particular interest is how and why the HRQoL of people afflicted with the same disease is differently affected by stigma (Chronister et al., 2013). The variability in HRQoL outcomes of stigma might be attributable to the psychosocial resources of patients. In this regard, the sense of coherence (SOC) (Antonovsky, 1987; Griffiths et al., 2011), coping strategies (Carver, 1997), and social support (Sherbourne and Stewart, 1991) have been extensively proposed as the general resistance resources (GRRs) in the health domain. On one hand, GRRs may be impacted by stigma as a social and interpersonal stressor that interferes with the patients’ social and personal identity (Goffman, 1963; S. Knapp et al., 2014). On the other hand, GRRs may act as a buffer in the stigma-HRQoL link because of their well-established role in reducing perceived stress. Thus, the current study was based on the stress-reduction effect of these GRRs in protecting patients’ HRQoL from experienced stigma, considering that stigma may also diminish these resources’ efficacy.

Sense of coherence (SOC) was developed to identify the factors which enable individuals to maintain their wellbeing under strain (Antonovsky, 1987; Griffiths et al., 2011). There are cognitive, behavioral, and motivational aspects to SOC, which are defined as comprehensibility, manageability, and meaningfulness, respectively (Strang and Strang, 2001). Patients with higher SOC have been shown to comprehend the disease and its complications better (Sarenmalm et al., 2013). The more the patients feel that there are SOC available to them, the more likely they perceive cancer as a manageable challenge, and consequently, they can perceive life as more meaningful (Langius-Eklöf et al., 2008; Lashani et al., 2021). For Iranian women with breast cancer, SOC has been a better predictor of HRQoL compared to coping strategies (Rohani et al., 2015; Zamanian et al., 2021a).

Coping skills are essential for the stabilization of mental health in the face of life stressors (Biggs et al., 2017). They influence the HRQoL of patients with breast cancer such that those who practice passive coping were associated with poor HRQoL (Filazoglu and Griva, 2008; Zamanian et al., 2015). On the contrary, positive coping skills, including fighting spirit, promoting HRQoL (Velasco et al., 2020), and functional coping strategies, including active coping, positive reframing, use of emotional and instrumental support, contribute to higher HRQoL (Zamanian et al., 2021a).

Social support is an individual’s perception of the availability of external assistance and contributes to various domains of HRQoL (Zamanian et al., 2021c). Sherbourne and Stewart (1991) held social support as a unidimensional construct that includes various aspects of emotional-informational support, affectionate support, tangible support, and positive social interactions. The emotional-informational subscale was a strong predictor of both the mental and physical health of women with breast cancer (Leung et al., 2014). Positive social interactions were also revealed to contribute to the emotional, social, and functional dimensions of HRQoL (Zamanian et al., 2021c). In addition, where social support is lower in cancer survivors, they might employ maladaptive coping strategies more frequently to the cost of a declined HRQoL(Durá-Ferrandis et al., 2017).

Thus far, the role of social support in reducing the destructive effect of stigma on HRQoL has been addressed in various chronic conditions (Earnshaw et al., 2012; Nearchou et al., 2017), people with HIV (Larios et al., 2009), and lung cancer (Lei et al., 2021). It is also suggested by the social support deterioration deterrence model that stigma as an impactful stressor can break the people’s support system resulting in diminished mental health (Kaniasty and Norris, 1993; Norris and Kaniasty, 1996; Chang et al., 2021). Likewise, SOC was observed as a protective factor for HRQoL in the stigmatizing context of patients with chronic diseases (Broersma et al., 2018), and its mediating effect was shown in the stigma-HRQoL link (Świtaj et al., 2017), suggesting an insidious effect of stigma on patients’ GRRs. Whereas one may find various reports on the role of coping strategies in tackling stigma in people with mental health issues, few studies have addressed this pivotal GRR in cancer populations; for example, self-efficacy for coping was shown effective in preserving HRQoL from self-stigma in patients with breast cancer (Chu et al., 2021). Although some empirical studies have provided preliminary evidence for the role of coping strategies and social support in psychological outcomes among patients with breast cancer (Kang et al., 2020), their contribution to specific domains of HRQoL, especially in Iranian patients, needs further investigation. In fact, the use of a multidimensional approach involving societal knowledge promotion initiatives and the establishment of support centers has been suggested to improve the currently moderate level of the HRQoL in Iranian patients (S. Bouya et al., 2018), implying that the efforts should be implemented in both individual and social levels.

**FIGURE 1:**
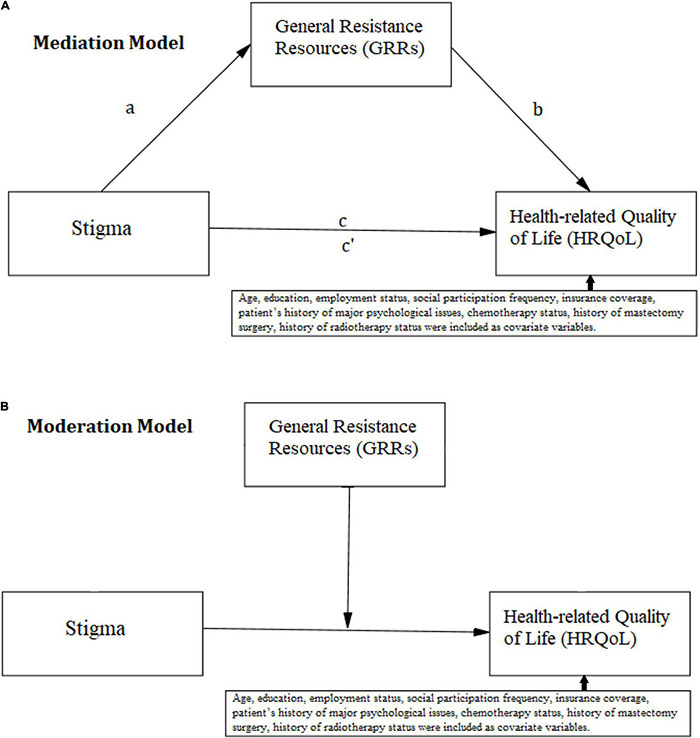
Conceptual model for **(A)** mediation analysis, **(B)** moderation analysis, including the covariate variables.

To address the inter-relations among stigma and GRRs, we have adopted both mediation and moderation analysis to address the research hypotheses illustrated by Figure 1, as per the specifications applied in the current literature (Świtaj et al., 2017; Wang et al., 2019; Yu and Xiao, 2021). We have based our strategy on the notion of “competing models” explained by Rose et al. (2004, pp. 59). Accordingly, competing models of mediation and moderation are explored to examine whether the GRRs could either become affected by stigma, resulting in reduced HRQoL, or buffer the stigma-HRQoL link in the context of breast cancer in Iranian women.

## Materials and Methods

### Participants

In this cross-sectional study, 223 women with breast cancer were recruited from October 2014 to May 2015 *via* convenient sampling from three cancer centers in Tehran, Iran. Along with the previous publications (Zamanian et al., 2015; Zamanian et al., 2020; Zamanian et al., 2021c), the current study was derived from a Ph.D. research project conducted on the health concerns of Iranian patients with breast cancer (PS−BrC2015) and the Ethics Committee of Tehran University of Medical Sciences has approved of it under the code TUMS.1394.6049. To be included, patients had to meet the following criteria: age ≥ 18; pathology-confirmed breast cancer diagnosis at least 1 month before recruitment; basic Farsi language skills. They were asked to provide written informed consent and were excluded if they had a past psychiatric history or showed any evidence of metastatic brain disease.

### Measures

#### Demographic and Clinical Information

Demographic and clinical information was gathered using a self-report checklist completed by patients, which include patient’s age, education (under diploma versus a diploma or higher), spouse’s education (under diploma versus a diploma or higher), patient’s employment status (unemployed versus employed), insurance coverage (Yes/No), housemate (living with the spouse, living with other than the spouse), self-reported history of hospitalization because of cancer or its related conditions (Yes/No), self-reported history of major psychological problems (“*Have you struggled with any psychological issue, such as depression, anxiety, panic, etc., for which you used specific medical treatment or psychotherapy during your life?* Yes/No), self-reported history of mastectomy (no surgery, partial mastectomy, total mastectomy), self-reported chemotherapy status (finished, ongoing, none), self-reported history of radiotherapy (Yes/No), and self-reported time since diagnosis. The patient’s social participation frequency was assessed, asking them a single question which went as: *“In the past week, how many times have you participated in social activities, such as group outdoor or voluntary activities, attending mosque, museum, cinema, park, etc.?”*

#### Stigma

Stigma was assessed using the stigma scale for chronic illnesses 8-item version (SSCI-8), which is developed for patients with chronic conditions (Molina et al., 2013). The original Stigma Scale for Chronic Illnesses (SSCI-24) was initially developed to address both enacted and internalized stigma of patients with neurologic conditions; however, due to redundancies, it was truncated to a shorter version (SSCI-8), allowing for a more efficient yet comprehensive assessment of stigma (Molina et al., 2013). Each item is scored on a 5-point Likert scale, and the total score ranges from 8 to 40, with higher scores indicative of higher stigmatization. The scale has been validated in Iranian patients with breast cancer (Zamanian et al., 2020). In the current sample, its internal consistency was calculated as Cronbach’s alpha of 0.87 for the total stigma.

#### Sense of Coherence

Sense of coherence (SOC) was assessed using the 13-item Orientation to Life Questionnaire (QLQ-13, known as SOC-13) developed by Antonovsky (1993). It consists of three components: comprehensibility (5 items, Cronbach’s alpha = 0.55), manageability (4 items, Cronbach’s alpha = 0.55), and meaningfulness (4 items, Cronbach’s alpha = 0.56), and is scored from 1 to 7, yielding a total attainable score range from 13 to 91 for SOC (Cronbach’s alpha = 0.73). The higher scores are indicative of stronger SOC. Its Persian version is confirmed to be a reliable and valid tool for the Iranian population, as indicated by Cronbach’s α of 0.81 (Rohani et al., 2015).

#### Perceived Social Support

Perceived social support was assessed using the Medical Outcomes Study Social Support Survey (MOS-SSS) (Sherbourne and Stewart, 1991). This instrument contains 19-items designed under five domains of Emotional-Informational support (8 items, Cronbach’s alpha = 0.95); Affectionate support (3 items, Cronbach’s alpha = 0.83); Tangible support (4 items, Cronbach’s alpha = 0.87); Positive social interactions (3 items, Cronbach’s alpha = 0.87). Item #13 does not belong to any specific facet, while it is included in the total social support. The items are rated on a 5-point Likert scale (ranging from 1; never to 5; always). In addition to item #13, the total social support (Cronbach’s alpha = 0.97) ranges from 19 to 95. This scale has been previously validated for the Iranian population (Bahri and Dehghan Manshadi, 2014).

#### Coping Strategies

Coping strategies were assessed using the 28-item Brief COPE developed by Carver (1997). This instrument asks partakers to rate their use of the following 14 strategies consisting of two items on a 4-point Likert scale (1 = “I have not done this at all” to 4 = “I have done this a lot”): use of emotional support (Cronbach’s alpha = 0.45), use of instrumental support (Cronbach’s alpha = 0.68), active coping (Cronbach’s alpha = 0.54), acceptance (Cronbach’s alpha = 0.57), planning (Cronbach’s alpha = 0.44), positive reframing (Cronbach’s alpha = 0.62), humor (Cronbach’s alpha = 0.65), religious cope (Cronbach’s alpha = 0.56), disengagement (Cronbach’s alpha = 0.44), self-distraction (Cronbach’s alpha = 0.48), denial (Cronbach’s alpha = 0.58), venting (Cronbach’s alpha = 0.44), substance use (Cronbach’s alpha = 0.51), and self-blame (Cronbach’s alpha = 0.69) (Carver, 1997). Each subscale would be scored from 2 to 8. It has been previously validated and employed for Iranian population (Aghayousefi, 2010; Khalili et al., 2013).

#### Health-Related Quality of Life

Health-related quality of life (HRQoL) was assessed using the Functional Assessment of Cancer Therapy–Breast Cancer (FACT-B) developed by Brady et al. (1997). The instrument is a 4-point Likert scale questionnaire and consists of two main parts: the functional assessment of cancer therapy-general (FACT-G) measures the general HRQoL including 27 items classified under four subscales of physical wellbeing (PWB: 7 items, Cronbach’s alpha = 0.83), social wellbeing (SWB: 7 items, Cronbach’s alpha = 0.70), emotional wellbeing (EWB: 6 items, Cronbach’s alpha = 0.72), and functional wellbeing (FWB: 7 items, Cronbach’s alpha = 0.76). FACT-B (Cronbach’s alpha = 0.89) is the sum of FACT-G (Cronbach’s alpha = 0.89) and the breast cancer-specific subscale (BCS: 10 items, Cronbach’s alpha = 0.52). The total score for FACT-G ranges from 0 to 108, and for FACT-B ranges from 0 to 148, and higher scores indicate better HRQoL. Its validity has been established for Iranian patients with breast cancer in prior studies with a Cronbach’s α of 0.92 for the total scale (Patoo et al., 2015; Zamanian et al., 2020). Of note is that for 154 patients with a history of mastectomy, to whom arm subscale was relevant, the arm subscale (4 items, Cronbach’s alpha = 0.85) of FACT-B+4 was administered (Brady et al., 1997).

### Data Analysis

Descriptive statistics reported the patients’ characteristics in terms of frequency and percentage and the study variables in terms of mean and SD. The Pearson’s correlation coefficient was used to assess the paired correlation between the study variables. As suggested by Baron and Kenny (1986), zero-order correlations between the study variables were inspected to determine whether there is a significant association between each set of the independent variable independent variable (IV: stigma) and the mediators and the dependent variables (DV: HRQoL variables) (i.e., HRQoL variables), and between the proposed mediators and the outcomes. Thus, only the proposed mediators showing significant associations with both stigma and HRQoL variables were included in the mediation analysis. Conditional process analysis using Andrew Hayes’s PROCESS Macro version 3.5 for SPSS software application version 26, (IBM inc., Armonk, NY, United States), with 5,000 bootstrap replicates and 95% CI was employed for both single and parallel mediation analyses (Model 4) and moderation analysis (Model 1) (Hayes, 2017; Hayes and Rockwood, 2020). In the mediation analysis, while only one mediating variable was entered into the single mediation model, all of the significant mediators for a given outcome were simultaneously entered in a parallel mediation model. Heteroscedasticity was examined using Konker’s test (Daryanto, 2020) and treated using Caribari-Neto’s heteroscedasticity-consistent standard error (HC4) (Hayes and Cai, 2007). The completely standardized indirect effects were reported as per Hayes (2017, pp. 135–136). In the moderation analysis, the pick-a-point approach based on the 16th, 50th, and 80th percentiles of the moderating variable and the Neyman-Johnson (NJ) procedure where *p* < 0.10 for any interaction effect were used to pinpoint at which moderating variable’s level (i.e., turning point) the effect of stigma on HRQoL might vary (Hayes, 2017). To address the multiple testing, the Benjamini–Hochberg procedure with a false discovery rate of 0.10 and 0.2 were applied to all regression analysis in the mediation and moderation models, respectively (Benjamini and Hochberg, 1995). A standard notation of mediation analysis was used to report the mediation results, in which *c′* denotes the direct effect of stigma on the outcome, *a* denotes the effect of stigma on the mediators, *b* denotes the effect of the mediator on the outcome, and *a × b* denotes the indirect effect of stigma on the outcome through the mediator (Hayes, 2017).

The covariates were selected based on the literature, which shows that age (Ernst et al., 2017), education (Durá-Ferrandis et al., 2017), employment status (Rohani et al., 2015), insurance coverage (You et al., 2018), patient’s history of major psychological issues (Ernst et al., 2017; Świtaj et al., 2017), social participation frequency (Nikolić et al., 2015), chemotherapy status (Durá-Ferrandis et al., 2017), history of mastectomy (Rohani et al., 2015), and history of radiotherapy (Durá-Ferrandis et al., 2017) are the common covariates of HRQoL in patients with breast cancer. The covariates were included in all mediation models and were coded as reported in Table 1. In the current regression models, the subjects per variable ratios were from 22 to 15, which were satisfactory enough to address both estimation bias and statistical power (Austin and Steyerberg, 2015).

**TABLE 1:** Sample characteristics (*N* = 221).

Patient’s age, mean (SD)		47.14 (9.13)
Social participation frequency, mean (SD)		0.90 (1.44)
	*n*	%
**Patient’s education**		
≥diploma	42	19.0
<diploma	179	81.0
**Spouse’s education**		
≥diploma	39	21.3
<diploma	144	78.7
**Employment status**		
Unemployed	184	83.3
Employed	37	16.7
**Insurance status**		
Yes	201	91.0
No	20	9.0
**Housemate**		
Living with the spouse	166	75.1
Living with other than the spouse	55	24.9
**Hospitalization**		
No	121	54.8
Yes	100	45.2
**Patient’s history of major psychological problem**		
No	168	76.0
Yes	53	24.0
**History of mastectomy surgery**		
No surgery	67	30.3
Partial mastectomy	78	35.3
Total mastectomy	76	34.4
**Chemotherapy**		
Finished	75	33.9
Ongoing	61	27.6
None	85	38.5
**History of radiotherapy**		
Yes	88	39.8
No	133	60.2
Time since diagnosis (weeks), mean (SD)	18.31 (15.05)	

*Missing data for spouse’s education.*

*SD, standard deviation.*

The normality was evaluated using the standardized skewness index, which was set to be below 3.29 for a sample size of 221 (Kim, 2013); thus, social wellbeing, arm subscale, stigma, all the support types, denial, substance use, behavioral disengagement, humor, acceptance, religious coping were transformed to normal distribution *via* two-step procedure (Templeton, 2011). *P*-value < 0.05 was considered significant. For indirect effects, 95% CI was also reported.

## Results

### Descriptive Statistics

A total number of 256 patients were initially apprised, and 224 patients staged 1–4 were included in the study after the acquisition of informed consent (response rate = 87.5%). In the initial analysis, one patient who terminated her participation was also excluded. Due to a substantial lack of data, two patients were excluded, and the study was wrapped up with data from 221 valid participants. The sample’s mean age was 47.14 ± 9.13, 34.4% (*n* = 76) had undergone total mastectomy and 61.5% had received chemotherapy (*n* = 136). Table 1 demonstrates the demographic and clinical characteristics, and Table 2 summarizes the descriptive statistics of the study variables.

**TABLE 2:** Correlation matrix between study variables (*N* = 221).

Variables		Stigma	CO	MA	MF	SOC	EIS	AFS	TS	PSI	TSS	SD	AC	D	SU	UES	UIS	BD	V	PR	P	H	Acc	CR	SB
	
	M (SD)		20.41 (6.38)	16.75 (5.77)	20.65 (4.89)	57.80 (13.47)	32.32 (8.32)	12.06 (2.88)	16.08 (3.96)	12.36 (3.09)	78.05 (18.01)	2.84 (0.89)	2.81 (0.83)	1.82 (.86)	1.13 (0.45)	2.83 (0.87)	2.77 (0.92)	1.58 (0.76)	2.39 (0.87)	2.70 (0.93)	2.80 (0.81)	1.76 (0.087)	3.25 (0.74)	3.51 (0.67)	2.15 (0.97)
PWB	17.00 (6.71)	−0.38***	0.10	0.13	0.25**	0.19**	0.13	0.13	0.05	0.20**	0.12	0.06	0.24**	–0.08	–0.05	0.17*	0.11	–0.10	0.04	0.23**	0.16*	0.07	0.13	0.05	–0.06
SWB	190.37 (5.00)	−0.27***	0.19**	0.21**	0.34**	0.30**	0.41**	0.43**	0.39**	0.44**	0.43**	0.11	0.06	–0.03	–0.01	0.31**	0.31**	–0.10	0.01	0.21**	–0.01	0.04	0.15*	0.11	−0.19**
EWB	14.84 (5.25)	−0.39***	0.32**	0.26**	0.36**	0.39**	0.21**	0.22**	0.16*	0.25**	0.22**	0.04	0.15*	–0.11	–0.00	0.24**	0.19**	–0.12	–0.12	0.30**	0.18**	0.14*	0.21**	0.09	−0.22**
FWB	18.28 (5.12)	−0.36***	0.25**	0.21**	0.32**	0.33**	0.35**	0.35**	0.20**	0.38**	0.34**	0.16*	0.19**	–0.13	–0.03	0.25**	0.23**	−0.19**	–0.05	0.30**	0.15*	0.15*	0.25**	0.09	–0.12
BCS	21.48 (5.57)	−0.39***	0.31**	0.24**	0.29**	0.36**	0.17**	0.19**	0.18**	0.21**	0.19**	–0.02	0.12	–0.10	–0.06	0.12	0.01	–0.13	–0.08	0.17*	0.07	–.03	0.17*	0.06	−0.25**
Arm*^a^*	13.47 (5.33)	−0.28***	0.19**	0.22**	0.07	0.21**	0.04	0.08	0.08	0.07	0.06	0.04	0.17*	–0.07	0.04	0.09	0.03	–0.08	–0.03	0.21**	0.03	–0.04	0.08	0.12	−0.16*
FACT_G	68.83 (16.54)	−0.49***	0.28**	0.27**	0.43**	0.40**	0.35**	0.37**	0.25**	0.42**	0.36**	0.14*	0.23**	–0.12	–0.03	0.31**	0.27**	−0.18**	–0.03	0.34**	0.18**	0.15*	0.24**	0.11	−0.18**
FACT-B	90.32 (20.07)	−0.51***	0.32**	0.29**	0.43**	0.43**	0.34**	0.36**	0.25**	0.40**	0.35**	0.11	0.23**	–0.13	–0.05	0.29**	0.22**	−0.18**	–0.05	0.33**	0.15*	0.10	0.25**	0.10	−0.22**
Stigma	11.75 (5.57)		−0.27**	−0.26**	−0.30**	−0.35**	−0.26**	−0.25**	−0.23**	−0.30**	−0.25**	–0.1	−0.19**	0.17*	–0.04	−0.15*	–0.11	0.26**	0.11	−0.28**	–0.11	0.05	−0.18**	–0.01	0.31**

*P < 0.05, ** P < 0.01, *** P < 0.001.

*M (SD), mean (standard deviation); PWB, Physical wellbeing; SWB, Social wellbeing; EWB, Emotional wellbeing; FWB, Functional wellbeing; BCS, Breast cancer subscale; Arm, Arm subscale; FACT-G, Functional assessment cancer therapy–General; FACT-B, Functional assessment cancer therapy–breast cancer; CO, Comprehensibility; MA, Manageability; MF, Meaningfulness; SOC, Sense of Coherence; EIS, Emotional/informational support; AFS, Affectionate support; TS, Tangible support; PSI, Positive social interactions; TSS, total Social Support; SD, Self-distraction; AC, Active Coping; D, Denial; SU, Substance use; UES, Use of emotional support; UIS, Use of instrumental support; PR, Positive reframing; P, Planning; H, Humor; Acc, Acceptance; CR, Cope Religious; SB, Self-blame.*

*One missing data for positive reframing, behavioral disengagement, substance use, and venting.*

*^a^In the subsample undergone mastectomy surgery (n = 154).*

### Correlational Analysis

Table 2 presents the zero-order correlations between study variables to appraise the pre-conditions of mediation analysis (Baron and Kenny, 1986). There were significant relationships between stigma and the HRQoL outcomes, from FACT-B (*r* = −0.51, *P* < 0.001) to arm subscale (*r* = −0.26, *P* < 0.001). Therefore, the relationship between the independent variable and all the outcomes was established. In terms of the proposed mediators, self-distraction, substance use, use of instrumental support, venting, planning, humor, and religious coping showed a non-significant association with stigma. The denial, while indicating a significant association with stigma (*r* = 0.17, *P* < 0.05), did not show any significant relationship with HRQoL outcomes. Therefore, these potential coping mediators were completely excluded from mediation analysis. Likewise, some other proposed mediators with a non-significant relationship with some of the HRQoL outcomes, as illustrated by asterisk-free correlation coefficients in Table 2, were only included in mediation analysis where they showed eligible correlations.

### Mediation Analysis

The details of the mediation results are presented as the Supplementary Material of the supporting results, in which the results of the Benjamini–Hochberg procedure are reported. For simplicity and a concise illustration, only the significant indirect and direct effects are reported in Table 3, in which the model numbers in the Supplementary Material (model figures) are introduced. The Supplementary Material of model figures illustrates the details of each model.

**TABLE 3:** Mediation analysis of sense of coherence, social support, and coping behaviors in the effect of stigma on HRQoL (*N* = 221–219).

																																	
IV = Stigma	DV																																
	PWB (Model 1)			SWB (Models 2–9)					EWB (Models 10–14)					FWB (Models 15–22)					BCS (Models 23–26)					FACT-G (Models 27–34)					FACT-B (Models 35–45)				
	Single mediation			Single mediation			Parallel mediation (c′ = −0.14*, *P* = 0.026)		Single mediation			Parallel mediation (c′ = −0.25***)		Single mediation			Parallel mediation (c′ = −0.21**)	Single mediation			Parallel mediation (c′ = −0.31**)		Single mediation			Parallel mediation (c′ = −0.32***)		Single mediation			Parallel mediation (c′ = −0.34***)	
Mediators	c′	ab	95%CI	c′	ab	95%CI	ab	95%CI	c′	ab	95%CI	ab	95%CI	c′	ab	95%CI			c′	ab	95%CI	ab	95%CI	c′	ab	95%CI	ab	95%CI	c′	ab	95%CI	ab	95%CI
																																	
Comprehensibility									−0.32***	−0.05	**HC4 [−0.09, −0.01]**	*−0.01*	*[−0.01, 0.00]*						−0.33***	−0.05	**[−0.10, −0.02]**	**−0.04**	**[−0.09, −0.01]**						−0.44***	−0.04	**[−0.08, −0.01]**	*−0.02*	*[−0.05, 0.02]*
Manageability																													−0.44***	−0.04	**[−0.07, −0.01]**	*−0.01*	*[−0.04, 0.02]*
Meaningfulness	−0.33***	−0.04	**[−0.09, −0.006]**	−0.18**	−0.06	**[−0.11, −0.02]**	−0.04	**[−0.08, −0.01]**	−0.31***	−0.06	**HC4 [−0.12, −0.02]**	−0.05	**HC4 [−0.10, −0.01]**	−0.27***	−0.05	**[−0.10, −0.01]**	*−0.03*	*[−0.08, 0.01]*	−0.34***	−0.04	**[−0.08, −0.01]**	*−0.03*	*[−0.07, 0.004]*	−0.38***	−0.07	**[−0.12, −0.03]**	−0.06	**[−0.10, −0.02]**	−0.41***	−0.07	**[−0.12, −0.03]**	**−0.05**	**[−0.09, −0.01]**
Sense of coherence				−0.18**	−0.06	**[−0.11, −0.02]**			−0.29***	−0.08	**HC4 [−0.13, −0.04]**			−0.26***	−0.06	**[−0.10, −0.02]**			−0.31***	−0.07	**[−0.13, −0.03]**			−0.38***	−0.08	**[−0.12, −0.03]**			−0.40***	−0.08	**[−0.13, −0.04]**		
Emotional-informational				−0.17**	−0.07	**[−0.12, −0.02]**								−0.26***	−0.05	**[−0.10, −0.02]**								−0.41***	−0.05	**[−0.09, −0.02]**			−0.44***	−0.04	**[−0.08, −0.01]**		
Affectionate				−0.18**	−0.06	**[−0.11, −0.01]**								−0.27***	−0.05	**[−0.09, −0.01]**								−0.41***	−0.04	**[−0.09, −0.01]**			−0.44***	−0.04	**[−0.08, −0.01]**		
Tangible				−0.19**	−0.05	**[−0.10, −0.01]**																							−0.45	−0.02	**[−0.05, −0.001]**		
Positive social interaction				−0.15**	−0.09	**[−0.15, −0.04]**								−0.24***	−0.07	**[−0.13, −0.03]**								−0.38***	−0.07	**[−0.12, −0.03]**			−0.41***	−0.07	**[−0.11, −0.03]**		
Total social support				−0.17**	−0.07	**[−0.12, −0.02]**	**−0.06**	**[−0.11, −0.02]**						−0.26***	−0.05	**[−0.09, −0.02]**	−0.03	**[−0.07, −0.01]**						−0.41***	−0.05	**[−0.08, −0.01]**	−0.03	**[−0.06, −0.003]**	−0.44***	−0.04	**[−0.08, −0.01]**	*−0.02*	*[−0.05, 0.00]*
Positive reframing									−0.32***	−0.06	**HC4 [−0.10, −0.02]**	−0.04	**HC4 [−0.08, −0.005]**	−0.26***	−0.05	**[−0.10, −0.02]**	−0.04	**[−0.08, −0.003]**						−0.40***	−0.06	**[−0.10, −0.02]**	−0.03	**[−0.07, −0.004]**	−0.43***	−0.05	**[−0.10, −0.02]**	*−0.03*	*[−0.07, −0.002]*

*P < 0.05, ** P < 0.01, ** P < 0.001.

*HRQol, Health-related quality of life; IV, Independent variable; DV, Dependent variable; PWB, Physical wellbeing; SWB, Social wellbeing; EWB, Emotional wellbeing; FWB, Functional wellbeing; BCS, Breast cancer subscale; Arm, Arm subscale; FACT-G, Functional assessment cancer therapy–General; FACT-B, Functional assessment cancer therapy–breast cancer; c′, Direct effect of stigma on DVs; ab (a × b), Indirect effect of stigma on DVs; CI, Confidence interval.*

*One missing value for positive reframing (N = 220).*

*Age, education, employment status, social participation frequency, insurance coverage, patient’s history of major psychological problems, chemotherapy status, history of mastectomy surgery, history of radiotherapy status was included as covariates.*

*All Cis are bolded to denote significant as per Benjamini-Hochberg Procedure reported in Supplementary Material of supporting results in detail.*

*HC4: Heteroscedasticity consistent standard error of Caribari-Neto is used.*

*Models numbers denote the figures included in Supplementary Material of model figures.*

As Table 3 presents, all facets of sense of coherence, all social support types, and coping strategy of positive reframing could mediate the relationship between stigma and different HRQoL domains. Notably, only meaningfulness played a significant mediating role for all HRQoL domains and total scores, and it was the sole significant mediator in parallel mediation for FACT-B and the main mediator for FACT-G. Social support mostly exerted its mediation effect for SWB, FWB, FACT-G, and FACT-B, in which tangible support showed a significant mediation effect only for SWB and FACT-B. In sum, while the arm subscale did not yield significant results over and above covariate variables, the SWB and FWB domains most enjoyed the mediating role of GRRs, and the mediation effects were more highlighted for FACT-B. All of the mediation effects were partial, as the direct effect of stigma on HRQoL variables remained significant and strongly far from zero (Baron and Kenny, 1986).

### Moderation Analysis

Moderation analysis was run for all GRRs regardless of their association with the IV (i.e., stigma) or DVs (HRQoL variables), the results of the Benjamini–Hochberg procedure for models indicating a significant interaction effect (*P* < 0.10) are presented in the Supplementary Material-moderation results, the corresponding figures are presented in Supplementary Material-moderation figures.

The stigma-SWB linkage was dampened by a high level of humor (*P* = 0.0031, turning point at values > 0.61, 26.82%, *n* = 59, Supplementary Figure 1). The stigma-EWB linkage was lower at higher levels of behavioral disengagement (*P* = 0.0395, turning point values < 0.70, 6.39%, *n* = 14, Supplementary Figure 2). The stigma-FWB linkage was lower at higher levels of humor (*P* = 0.0982, turning point values > 0.77, 13.63%, *n* = 30, Supplementary Figure 3), and it was dampened by a high level of use of instrumental support (*P* = 0.0651, turning point values > 3.57, 20.45%, *n* = 45, Supplementary Figure 4), a high level of behavioral disengagement (*P* = 0.0299, turning point values < 0.66, 19.63%, *n* = 43, Supplementary Figure 5), and a high level of venting (*P* = 0.0104, turning point values < 3.15, 17.35%, *n* = 38, Supplementary Figure 6). The stigma-Arm subscale linkage was dampened by a “*low*” level of venting (*P* = 0.0827, turning point values < 2.18, 47.06%, *n* = 72 of *N* = 153, Supplementary Figure 7), however. In addition, higher levels of behavioral disengagement (*P* < 0.0222, turning point value > 0.75, 6.39%, *n* = 14, Supplementary Figure 8) and venting (*P* = 0.056, turning point value > 3.91, 8.22%, *n* = 18, Supplementary Figure 9) could mitigate the stigma-FACT-G linkage. In stigma-FACT-B linkage, the moderating effect of higher behavioral disengagement (*P* = 0.0489, turning point value > 0.79, Supplementary Figure 10) was significant in 4.11%, *n* = 9, of the sample.

Some anomalous results were seen. The stigma-EWB linkage was lower at lower levels of comprehensibility (*P* = 0.030, turning point values < 12.78, 11.31%, *n* = 25, Supplementary Figure 11). Likewise, a low level of comprehensibility was shown to dampen the stigma-FACT-G (*P* = 0.055, Supplementary Figure 12) and stigma-FACT-B (*P* = 0.0473, Supplementary Figure 13) linkages with respective turning point values < 8.75 and 8.11, both of which included only 3.62%, *n* = 8, of the sample. Some explanations are given in the discussion section.

## Discussion

The current study aimed to explore whether general resistance resources, including the sense of coherence, perceived social support, and coping strategies, could be affected by stigma or mitigate the relationship between stigma and quality of life in Iranian women with breast cancer. In this study, while the significant associations between stigmatization and all domains of quality of life are evident, the adverse effect of stigma on various resources and quality of life was highlighted. It also may be fairly reduced by some coping strategies, and the effects depended on the quality of life domains. In addition, mediation and moderation analysis suggested different ways in which the general resistance resources may interplay with stigma. The cross-sectional nature of the data hinders us from any causal inference; thus, further interpretations and implications should be regarded with caution.

### Sense of Coherence

In the mediation analysis, meaningfulness was the single mainstay of mediation for all quality of life domains except for the arm subscale. Comprehensibility was the mediator for emotional wellbeing, breast cancer-specific domain, and overall breast cancer quality of life, which could be attributed to its association with breast cancer-specific domain, the differentiating subscale between breast cancer and general quality of life. Manageability may merely surface as a mediator for breast cancer quality of life, suggesting that the possible damage of stigma on this facet could exert a general impact on the quality of life of patients with breast cancer. Overall, the adverse effect of stigma on the global sense of coherence was observed in all domains of quality of life except for physical wellbeing. These results suggest that stigma might be emphasized as a disintegrating phenomenon that confers the risk of a diminished sense of coherence, especially meaning of life events, which results in lower quality of life in patients with breast cancer. However, some inconsistent results were observed in the moderating effect of comprehensibility for emotional wellbeing, overall, and breast cancer quality of life.

Contrary to predictions, patients with lower levels of comprehensibility showed a lower effect of stigma on emotional wellbeing, which was reflected again in the global sense of coherence. This finding is unique to the current sample, and it may be addressed in future investigations. Statistically speaking, this result indicates that, while overall stigma was higher and overall emotional wellbeing was lower in the subsample with lower comprehensibility, no linear relationship was observed between the former and the latter, i.e., the patients with higher or lower stigma in this subsample showed inconsistent levels of emotional wellbeing. Nonetheless, the sample’s poor educational backgrounds might affect the results. Lower levels of education may negatively affect both manageability and comprehensibility (Ciairano et al., 2008). However, partnered individuals with lower educational levels could maintain higher meaningfulness (Ciairano et al., 2008). Thus, the current sample’s lower educational level could be implicated in the results of comprehensibility and manageability. However, it may be implied that the sample’s relatively high level of received support could bolster their meaningfulness as a dominant protective factor. The current study did not consider the inter-relations among the resistance factors. Nonetheless, some studies suggest that a sense of coherence is partly constituted by social resources (Idan et al., 2017), and social support can bolster patients’ sense of coherence where education and information are needed (Cecon et al., 2021). Thus, further investigations may shed light on the effect of meaningfulness as a dominant aspect of the sense of coherence with respect to the role of high social support in a stigmatized context.

Meaningfulness, as the principal mediator in the current study, seems to be the key dimension of sense of coherence and might carry off the role of the other two facets (Super et al., 2015). The socially threatened sense of self brought about by stigma endangers meaningful orientation toward life and ongoing (adverse) events, which are crucial for patients with chronic diseases (Wang et al., 2016; Jin et al., 2020). So, stigma may impact the quality of life in patients with breast cancer by diminishing their sense of meaningfulness. On the other way round, the meaningfulness’s extensive functionality might be of added relevance to implementing salutogenic meaning-based treatment and emphasizes the importance of mind-body perspective in psycho-oncology (Chaoul et al., 2014).

### Social Support

Moderation analysis confirmed that stigma could affect the quality of life in all levels of social support, which was contrary to previous studies suggesting the moderating effect of social support for depression, worry, and cancer-related intrusive thoughts (Lewis et al., 2001; Huang and Hsu, 2013; Waters et al., 2013). However, all types of social support played a mediating role on social wellbeing, functional wellbeing, the general quality of life, and breast cancer quality of life, where tangible support only served as a mediator for social wellbeing and breast cancer quality of life. In other words, stigma could tear apart the protective effect of perceived social support resources as the most marked boosters of quality of life in cancer patients. In the same vein, stigma as a social phenomenon has been shown to strongly affect social wellbeing (Ernst et al., 2017). In our study, besides the general and breast cancer quality of life, social wellbeing followed by functional wellbeing were the only domains that were diminished as a result of stigma-induced stress affecting any subtypes of social support. In patients with HIV, social support was suggested to be the full mediator of stigma and global quality of life (Rao et al., 2012) and their functional, physical, and psychological wellbeing (Larios et al., 2009). Overall, these study findings point to the adverse effect of stigma on the critical role of interpersonal support systems in the maintenance of the social and functional wellbeing of patients with breast cancer.

Markedly, as another adverse effect of stigma, social support failed to maintain its well-established positive effect on emotional wellbeing, meaning that stigma could eliminate all the effects of social support on emotional wellbeing. Consistent with previous findings on the diminished protective role of social support in the stigma-depression link (Chang et al., 2021), these findings are in sheer accordance with the social support deterioration deterrence model suggesting stressors can paralyze the social support system at the cost of mental health problems (Kaniasty and Norris, 1993; Norris and Kaniasty, 1996). Two explanations in personal and group dynamic levels may be provided. Where stigma occurs, emotional wellbeing might be more sensitive to self-perceptions, including self-esteem and stigma resistance (Mashiach-Eizenberg et al., 2013; Firmin et al., 2016). In other words, the susceptibility to self-stigma may not be alleviated by external supports, even in emotional and affectionate types. On the other way round, the distress in the patients’ social network (i.e., couples) (Sprung et al., 2011) might be seen as a group emotional vulnerability, the result of which might render the effectiveness of caregivers’ supportive maneuvers fail in the context of stigma. Further studies are needed to appraise these suggestions.

### Coping Skills

Mediation and moderation analysis highlighted different roles for coping strategies in the stigma-HRQoL link. Stigma showed its adverse effect best on positive reframing in diminishing breast cancer quality of life, the general quality of life, emotional, and functional wellbeing. In other words, positive reframing that may contribute to better physical and emotional wellbeing (Kvillemo and Branstrom, 2014) was the marked pathway through which stigma could impact the capability of adoptingl a positive outlook toward the patients’ condition and in turn damaging a vast array of their life. Some studies have promoted psychoeducation interventions for the positive reframing of cancer trajectory because of the fluctuation in the quality of life in patients with breast cancer (Bayram et al., 2014). It also has been highlighted as a key resistance resource promoting the use of psych-oncological care (Cecon et al., 2021). Thus, interventions on positive reframing may be of importance where women with breast cancer perceive stigma as a psychosocial threat. Nonetheless, the current findings highlighted the role of cognitive coping with stigma in women with breast cancer.

Interestingly, moderation analysis indicated the higher levels of humor, behavioral disengagement, and use of instrumental support behavioral to be functional in the stigma-HRQoL link, while the results were mixed for venting, especially in terms of arm subscale in patients with mastectomy. These findings not only suggested the different protective mechanisms for quality of life dimensions but also mirrored previous suggestions as to the variabilities in the effect of a given coping strategy in various situations, especially during the course of cancer (Reddick et al., 2005; Ahlstedt Karlsson et al., 2020). In terms of behavioral disengagement, some studies suggested that when aggregated with denial to form disengagement coping, behavioral disengagement could contribute to heightening emotional distress and physical wellbeing, respectively, in higher states of functional impairment (Merluzzi et al., 2021) and lifetime stressful events (Langford et al., 2017; Merluzzi et al., 2019). Some other studies also suggested venting to be a function of maladaptive mental processes of pessimism in evoking cancer distress (David et al., 2006), which also showed its lower levels to be functional in terms of arm subscale in the current patients with mastectomy (*n* = 153). On the other hand, humor is shown to be functional in tackling the detrimental effect of stigma. As a component of positive thinking strategies (along with acceptance and positive reframing), humor might borrow its effect on the quality of life from social support (Tomai et al., 2019). It may argue that the findings of previous publications might be affected by the aggregation of coping strategies, which is in contrast to Carver’s suggestion (Carver, 1997). Importantly, coping strategies may be employed differently across cultures. Middle-eastern patients with cancer may rely on more cognitive and cultural-based copings while their western counterparts could enjoy more behavioral and social copings (Thein et al., 2020). In the context of stigma, the emphasis on positive reframing and humor may be anchored on some Iranian cultural practices of interpersonal conflicts (Behzadi, 1994), in which reconciliation is valued as an endpoint of any disputation. Overall, further studies are recommended to scrutinize how these coping strategies are employed in the face of stigma in women with breast cancer.

### Implications and Future Directions

The current findings suggest that the protecting role of the resistance resources seems far from enough to negate stigma and maintain quality of life effectively and they may diminish where stigma occurs. The distinction between enacted and internalized facets of stigma could have resulted in different findings. Enacted stigma may evolve into internalized stigma and a sense of shame (Molina et al., 2013), which may explain how patients vary in their response to stigma due to the presence or absence of self-stigma (Rüsch et al., 2006). Moreover, some internal vulnerabilities such as body image may bridge between cancer-associated stigma and the patients’ mental health (Esser et al., 2018; Zamanian et al., 2021b). Future studies may thus investigate whether the resistance resources could exert their protective effects through such pathways.

Regardless, our findings emphasize the gravity of stigma as a psychosocial concern; hence, both personal and social aspects of resistance resources are subject to be decreased by stigma. As the results imply, in addition to social support (Kaniasty and Norris, 1993; Norris and Kaniasty, 1996), stigma vastly affects personal resources of meaningfulness and positive reframing to fail them in protecting overall breast cancer quality of life. In addition, only the patients who may employ higher levels of some coping strategies, namely, humor, venting, and behavioral disengagement, may find benefits in tackling stigma, while patients with mastectomy surgery should not overuse venting. Thus, interventions may aim for the activation and validation of these useful strategies where stigma is concerned, especially in Iranian patients. Nonetheless, Iranian women with breast cancer seem to have a moderate quality of life, and about 26% of all-type cancer patients reported to be struggling with high stigma (Salehoddin Bouya et al., 2018; Hasan Shiri et al., 2018). Given the destructive impact of stigma on all aspects of quality of life and several resistance resources, even relatively low rates of stigma should be taken seriously, and appropriate measures tailored to the cultural context are needed to be taken by health-policy makers.

### Study Strength and Limitations

The strength of the current study lies in its several considerations: As per current literature, we have employed two sets of mediation and moderation models, which could capture different ways of relationship among GRRs and stigma-HRQoL linkage. The other study’s strengths were the use of different covariates, investigation of several resistance resources, and utilization of a breast cancer-specific measure of the quality of life, i.e., FACT-B, as well as investigation of quality of life at a domain level. Conducting research on a sample of Iranian women with breast cancer where no similar study is available is another major strength. In addition, the findings enjoyed the application of the Benjamini–Hochberg procedure to address the estimation bias resulting from multiple testing.

However, some limitations merit consideration in the interpretation of our findings. Primarily, its reliance on a convenience sample from the capital city of Iran limits its generalizability. Patients mainly were low-educated and might over-represent cancer patients with similar educational characteristics. However, to reduce selection bias, patients were recruited from three centers with disparate locations within the city. Although the patients’ documentations were screened, the clinical information was mainly based on patients’ self-reporting, which could impose the risk of response bias. Our study design precludes us from any causal inference based on the findings. Resistance resources that had no significant correlation with stigma and quality of life (e.g., religious coping, substance use, and venting) were excluded from mediation analysis. This might hinder the generalizability of results to the samples with different characteristics and patients in various stages of disease and treatment where the role of specific resistance resources might become prominent. Longitudinal designs are needed to confirm the study findings and adequately consider the possible variations in patients’ stigma, sense of coherence, social support, coping, and quality of life throughout the cancer trajectory. Last but not least, two notable limitations of the applied modeling approach were the lack of considering the interplay between the mediators and equivalence models. For instance, in terms of the former, some studies suggest the mediation effect of sense of coherence between the coping strategies and HRQoL relationship (Zamanian et al., 2021a) and the complex network of influence among resistance resources (Cecon et al., 2021). In terms of the latter, it could be hypothesized that patients with lower physical wellbeing and arm functionality could be more vulnerable to experience stigmatization since social devaluations are suggested to occur more in the face of observable body deformation (Goffman, 1963; Pachankis et al., 2017). We leave these suggestions to be addressed in future studies.

In summary, the present study suggests that, in the face of stigma, breast cancer patients may lose their meaning in their life, perceived social support, and adopting positive reframing resulting in diminished quality of life. Thus, oncology health services may find psychoeducational interventions useful for the patients to combat cancer stigma and maintain quality of life, especially using education on making sense of living with cancer, bolstering social interactions, and improving positive outlook when encountering the stigma-inducing social and interpersonal contexts. At the community level, policymakers are recommended to address cancer stigma in their public health plans.

## Data Availability Statement

The raw data supporting the conclusions of this article will be made available by the authors, without undue reservation, upon reasonable request.

## Ethics Statement

The studies involving human participants were reviewed and approved by Tehran University of Medical Sciences, Tehran, Iran. Written informed consent for participation was not required for this study in accordance with the national legislation and the institutional requirements. Verbal informed consent was obtained from all participans.

## Author Contributions

HZ contributed to the conceptualization and study supervision. MA-T contributed to the formal analysis, interpretation, drafting, and correspondence. ZJ contributed to the interpretation and write-up. MD contributed to the conceptualization, administration, and resource. FR, NM, MA, RH, and FRT extensively contributed to data collection. All authors contributed to the critical review and approved the final version of the manuscript.

## Conflict of Interest

The authors declare that the research was conducted in the absence of any commercial or financial relationships that could be construed as a potential conflict of interest.

## Publisher’s Note

All claims expressed in this article are solely those of the authors and do not necessarily represent those of their affiliated organizations, or those of the publisher, the editors and the reviewers. Any product that may be evaluated in this article, or claim that may be made by its manufacturer, is not guaranteed or endorsed by the publisher.
