# Supplementary material for: Stigma and Quality of Life in Women With Breast Cancer: Mediation and Moderation Model of Social Support, Sense of Coherence, and Coping Strategies
Source: Front Psychol. 2022 Feb 14;13:657992. doi: 10.3389/fpsyg.2022.657992 (PMC8882621; doi:10.3389/fpsyg.2022.657992)
Supplement: Supplementary file 1 [file Data_Sheet_1.docx]

***************** PROCESS Procedure for SPSS Version 3.5 *****************

Written by Andrew F. Hayes, Ph.D. www.afhayes.com

Documentation available in Hayes (2018). www.guilford.com/p/hayes3

**************************************************************************

**Model : 4**

**Y : PWB = Physical Well-being**

**X : Stigma_IV**

**M : Meaningfulness**

Covariates:

Age_cov Educatio Employme Social_p Insuranc Major_Ps Chemo_co Mastecto Radiothe

Sample

Size: 221

**************************************************************************

OUTCOME VARIABLE:

Meaningf

Model Summary

R R-sq MSE F df1 df2 p

.43435 .18866 20.31616 4.88319 10.00000 210.00000 .00000

Model

coeff se t p LLCI ULCI

constant 22.12763 2.67375 8.27588 .00000 16.85680 27.39846

Stigma_I -.23369 .06482 -3.60503 .00039 -.36147 -.10590

Age_cov .05889 .03442 1.71069 .08861 -.00897 .12674

Educatio -1.59914 .92285 -1.73283 .08459 -3.41839 .22010

Employme .60739 .97747 .62139 .53502 -1.31953 2.53430

Social_p .53412 .21518 2.48215 .01384 .10992 .95831

Insuranc .02574 1.08506 .02372 .98110 -2.11327 2.16475

Major_Ps -1.33952 .73036 -1.83404 .06806 -2.77930 .10026

Chemo_co -.26584 .45987 -.57808 .56383 -1.17240 .64071

Mastecto -.84146 .43347 -1.94122 .05357 -1.69596 .01305

Radiothe .61470 .78626 .78180 .43521 -.93528 2.16468

Standardized coefficients

coeff

Stigma_I -.23532

Age_cov .10993

Educatio -.12862

Employme .04649

Social_p .15739

Insuranc .00151

Major_Ps -.11725

Chemo_co -.04631

Mastecto -.13858

Radiothe .06169

| **Benjamini-Hochberg Procedure** | | | | |
| --- | --- | --- | --- | --- |
|  |  | **Ascending P-value s** | **I = ranking** | **(I/10)* 0.10** |
|  | **Stigma** | **.00039** | **1** | **.01** |
|  | **Social participation frequency** | **.0138** | **2** | **.02** |
|  | **Mastectomy** | **~~.053~~** | **3** | **.03** |
|  | **Major Psychological issues** | **~~.068~~** | **4** | **.04** |
|  | **Education** | **~~.084~~** | **5** | **.05** |
|  | **Age** | **~~.088~~** | **6** | **.06** |
|  | **Radiotherapy** | **~~.435~~** | **7** | **.07** |
|  | **Employment** | **~~.535~~** | **8** | **.08** |
|  | **Chemotherapy** | **~~.563~~** | **9** | **.09** |
|  | **Insurance** | **~~.981~~** | **10** | **.10** |

**************************************************************************

OUTCOME VARIABLE:

PWB

Model Summary

R R-sq MSE F df1 df2 p

.46197 .21342 36.54767 5.15511 11.00000 209.00000 .00000

Model

coeff se t p LLCI ULCI

constant 24.57958 4.12976 5.95182 .00000 16.43825 32.72091

Stigma_I -.45170 .08959 -5.04179 .00000 -.62833 -.27508

Meaningf .23582 .09255 2.54794 .01155 .05336 .41829

Age_cov -.07458 .04649 -1.60424 .11017 -.16623 .01707

Educatio -2.61567 1.24659 -2.09826 .03708 -5.07318 -.15816

Employme -2.92365 1.31224 -2.22799 .02695 -5.51056 -.33673

Social_p .22660 .29282 .77385 .43989 -.35066 .80385

Insuranc .63670 1.45534 .43749 .66221 -2.23233 3.50572

Major_Ps .22915 .98741 .23207 .81671 -1.71741 2.17571

Chemo_co -.07096 .61729 -.11495 .90859 -1.28788 1.14596

Mastecto .40106 .58658 .68372 .49491 -.75532 1.55743

Radiothe -1.24403 1.05611 -1.17794 .24016 -3.32601 .83796

Standardized coefficients

coeff

Stigma_I -.33472

Meaningf .17354

Age_cov -.10246

Educatio -.15481

Employme -.16467

Social_p .04914

Insuranc .02756

Major_Ps .01476

Chemo_co -.00910

Mastecto .04861

Radiothe -.09187

| **Benjamini-Hochberg Procedure** | | | | |
| --- | --- | --- | --- | --- |
|  |  | **Ascending P-value s** | **I = ranking** | **(I/11)* 0.10** |
|  | **Stigma** | **.00000** | **1** | **.009** |
|  | **Meaningfulness** | **.011** | **2** | **.018** |
|  | **Employment** | **.0269** | **3** | **.027** |
|  | **Education** | **~~.037~~** | **4** | **.03636** |
|  | **Age** | **~~.110~~** | **5** | **.045** |
|  | **Radiotherapy** | **~~.240~~** | **6** | **.054** |
|  | **Social participation frequency** | **~~.439~~** | **7** | **.063** |
|  | **Mastectomy** | **~~.494~~** | **8** | **.072** |
|  | **Insurance** | **~~.662~~** | **9** | **.081** |
|  | **Major Psychological issues** | **~~.816~~** | **10** | **.0909** |
|  | **Chemotherapy** | **~~.908~~** | **11** | **.100** |

************************** TOTAL EFFECT MODEL ****************************

OUTCOME VARIABLE:

PWB

Model Summary

R R-sq MSE F df1 df2 p

.43472 .18898 37.50348 4.89344 10.00000 210.00000 .00000

Model

coeff se t p LLCI ULCI

constant 29.79782 3.63275 8.20255 .00000 22.63649 36.95916

Stigma_I -.50681 .08807 -5.75455 .00000 -.68043 -.33320

Age_cov -.06069 .04677 -1.29774 .19580 -.15289 .03150

Educatio -2.99279 1.25386 -2.38687 .01788 -5.46454 -.52103

Employme -2.78041 1.32806 -2.09358 .03750 -5.39846 -.16236

Social_p .35256 .29236 1.20588 .22922 -.22379 .92890

Insuranc .64277 1.47424 .43600 .66329 -2.26345 3.54898

Major_Ps -.08674 .99232 -.08741 .93043 -2.04293 1.86945

Chemo_co -.13365 .62482 -.21391 .83083 -1.36537 1.09806

Mastecto .20262 .58894 .34405 .73116 -.95837 1.36362

Radiothe -1.09907 1.06827 -1.02883 .30474 -3.20498 1.00685

Standardized coefficients

coeff

Stigma_I -.37556

Age_cov -.08338

Educatio -.17713

Employme -.15660

Social_p .07645

Insuranc .02782

Major_Ps -.00559

Chemo_co -.01713

Mastecto .02456

Radiothe -.08116

| **Benjamini-Hochberg Procedure** | | | | |
| --- | --- | --- | --- | --- |
|  |  | **Ascending P-value s** | **I = ranking** | **(I/10)* 0.10** |
|  | **Stigma** | **.00000** | **1** | **.01** |
|  | **Education** | **.0178** | **2** | **.02** |
|  | **Employment** | **~~.037~~** | **3** | **.03** |
|  | **Age** | **~~.195~~** | **4** | **.04** |
|  | **Social participation frequency** | **~~.229~~** | **5** | **.05** |
|  | **Radiotherapy** | **~~.304~~** | **6** | **.06** |
|  | **Insurance** | **~~.663~~** | **7** | **.07** |
|  | **Chemotherapy** | **~~.830~~** | **8** | **.08** |
|  | **Mastectomy** | **~~.731~~** | **9** | **.09** |
|  | **Major Psychological issues** | **~~.930~~** | **10** | **.10** |

************** TOTAL, DIRECT, AND INDIRECT EFFECTS OF X ON Y **************

Total effect of X on Y

Effect se t p LLCI ULCI c_ps c_cs

-.50681 .08807 -5.75455 .00000 -.68043 -.33320 -.07628 -.37556

Direct effect of X on Y

Effect se t p LLCI ULCI c'_ps c'_cs

-.45170 .08959 -5.04179 .00000 -.62833 -.27508 -.06799 -.33472

Indirect effect(s) of X on Y:

Effect BootSE BootLLCI BootULCI

Meaningf -.05511 .02752 -.11398 -.00763

Partially standardized indirect effect(s) of X on Y:

Effect BootSE BootLLCI BootULCI

Meaningf -.00829 .00414 -.01711 -.00117

Completely standardized indirect effect(s) of X on Y:

Effect BootSE BootLLCI BootULCI

Meaningf -.04084 .02056 -.08593 -.00582

*********************** ANALYSIS NOTES AND ERRORS ************************

Level of confidence for all confidence intervals in output:

95.0000

Number of bootstrap samples for percentile bootstrap confidence intervals:

5000

***************** PROCESS Procedure for SPSS Version 3.5 *****************

Written by Andrew F. Hayes, Ph.D. www.afhayes.com

Documentation available in Hayes (2018). www.guilford.com/p/hayes3

**************************************************************************

**Model : 4**

**Y : SWB = Social Well-being**

**X : Stigma_IV**

**M : Manageability**

Covariates:

Age_cov Educatio Employme Social_p Insuranc Major_Ps Chemo_co Mastecto Radiothe

Sample

Size: 221

**************************************************************************

OUTCOME VARIABLE:

Manageab

Model Summary

R R-sq MSE F df1 df2 p

.35462 .12576 30.37485 3.02083 10.00000 210.00000 .00137

Model

coeff se t p LLCI ULCI

constant 17.47806 3.26932 5.34609 .00000 11.03318 23.92295

Stigma_I -.26580 .07926 -3.35345 .00095 -.42205 -.10955

Age_cov .06260 .04209 1.48725 .13845 -.02037 .14557

Educatio 1.48418 1.12841 1.31528 .18985 -.74029 3.70865

Employme .90084 1.19520 .75372 .45186 -1.45528 3.25697

Social_p .51544 .26311 1.95900 .05144 -.00324 1.03413

Insuranc -1.09144 1.32676 -.82264 .41165 -3.70690 1.52403

Major_Ps -1.73020 .89305 -1.93742 .05404 -3.49069 .03028

Chemo_co -.05627 .56231 -.10007 .92038 -1.16476 1.05222

Mastecto -.68241 .53002 -1.28751 .19933 -1.72725 .36244

Radiothe .09180 .96140 .09548 .92402 -1.80343 1.98703

Standardized coefficients

coeff

Stigma_I -.22723

Age_cov .09921

Educatio .10134

Employme .05853

Social_p .12894

Insuranc -.05450

Major_Ps -.12857

Chemo_co -.00832

Mastecto -.09541

Radiothe .00782

| **Benjamini-Hochberg Procedure** | | | | |
| --- | --- | --- | --- | --- |
|  |  | **Ascending P-value s** | **I = ranking** | **(I/10)* 0.10** |
|  | **Stigma** | **.00095** | **1** | **.01** |
|  | **Social participation frequency** | **~~.051~~** | **2** | **.02** |
|  | **Major Psychological issues** | **~~.054~~** | **3** | **.03** |
|  | **Education** | **~~.189~~** | **4** | **.04** |
|  | **Age** | **~~.138~~** | **5** | **.05** |
|  | **Mastectomy** | **~~.199~~** | **6** | **.06** |
|  | **Insurance** | **~~.411~~** | **7** | **.07** |
|  | **Employment** | **~~.451~~** | **8** | **.08** |
|  | **Chemotherapy** | **~~.920~~** | **9** | **.09** |
|  | **Radiotherapy** | **~~.924~~** | **10** | **.10** |

**************************************************************************

OUTCOME VARIABLE:

SWBwn

Model Summary

R R-sq MSE F df1 df2 p

.44636 .19924 .06765 4.72744 11.00000 209.00000 .00000

Model

coeff se t p LLCI ULCI

constant .44499 .16446 2.70580 .00738 .12078 .76920

Stigma_I -.01196 .00384 -3.11504 .00210 -.01953 -.00439

Manageab .00714 .00326 2.19168 .02951 .00072 .01356

Age_cov -.00110 .00200 -.55265 .58109 -.00504 .00283

Educatio -.02729 .05347 -.51037 .61033 -.13271 .07813

Employme .00145 .05648 .02572 .97951 -.10990 .11280

Social_p .00515 .01253 .41108 .68143 -.01955 .02985

Insuranc .09368 .06272 1.49380 .13674 -.02995 .21732

Major_Ps -.14907 .04252 -3.50581 .00056 -.23290 -.06525

Chemo_co -.03650 .02654 -1.37520 .17054 -.08881 .01582

Mastecto .00873 .02511 .34778 .72835 -.04077 .05824

Radiothe .09431 .04537 2.07852 .03888 .00486 .18376

Standardized coefficients

coeff

Stigma_I -.20784

Manageab .14509

Age_cov -.03555

Educatio -.03788

Employme .00192

Social_p .02619

Insuranc .09509

Major_Ps -.22518

Chemo_co -.10970

Mastecto .02482

Radiothe .16333

| **Benjamini-Hochberg Procedure** | | | | |
| --- | --- | --- | --- | --- |
|  |  | **Ascending P-value s** | **I = ranking** | **(I/11)* 0.10** |
|  | **Major Psychological issues** | **.0005** | **1** | **.009** |
|  | **Stigma** | **.0021** | **2** | **.018** |
|  | **Manageability** | **~~.029~~** | **3** | **.027** |
|  | **Radiotherapy** | **~~.0388~~** | **4** | **.03636** |
|  | **Insurance** | **~~.136~~** | **5** | **.045** |
|  | **Chemotherapy** | **~~.170~~** | **6** | **.054** |
|  | **Age** | **~~.581~~** | **7** | **.063** |
|  | **Education** | **~~.610~~** | **8** | **.072** |
|  | **Social participation frequency** | **~~.681~~** | **9** | **.081** |
|  | **Mastectomy** | **~~.728~~** | **10** | **.0909** |
|  | **Employment** | **~~.979~~** | **11** | **.100** |

************************** TOTAL EFFECT MODEL ****************************

OUTCOME VARIABLE:

SWBwn

Model Summary

R R-sq MSE F df1 df2 p

.42525 .18084 .06888 4.63587 10.00000 210.00000 .00001

Model

coeff se t p LLCI ULCI

constant .56974 .15568 3.65961 .00032 .26284 .87664

Stigma_I -.01386 .00377 -3.67144 .00031 -.02130 -.00642

Age_cov -.00066 .00200 -.32767 .74349 -.00461 .00329

Educatio -.01670 .05373 -.31075 .75630 -.12263 .08923

Employme .00788 .05691 .13850 .88998 -.10432 .12008

Social_p .00883 .01253 .70475 .48175 -.01587 .03353

Insuranc .08589 .06318 1.35953 .17544 -.03865 .21044

Major_Ps -.16142 .04253 -3.79580 .00019 -.24526 -.07759

Chemo_co -.03690 .02678 -1.37795 .16969 -.08968 .01589

Mastecto .00386 .02524 .15305 .87851 -.04589 .05362

Radiothe .09496 .04578 2.07431 .03927 .00471 .18521

Standardized coefficients

coeff

Stigma_I -.24081

Age_cov -.02116

Educatio -.02318

Employme .01041

Social_p .04490

Insuranc .08718

Major_Ps -.24383

Chemo_co -.11091

Mastecto .01098

Radiothe .16446

| **Benjamini-Hochberg Procedure** | | | | |
| --- | --- | --- | --- | --- |
|  |  | **Ascending P-value s** | **I = ranking** | **(I/10)* 0.10** |
|  | **Major Psychological issues** | **.00019** | **1** | **.01** |
|  | **Stigma** | **.00031** | **2** | **.02** |
|  | **Radiotherapy** | **~~.039~~** | **3** | **.03** |
|  | **Chemotherapy** | **~~.169~~** | **4** | **.04** |
|  | **Insurance** | **~~.175~~** | **5** | **.05** |
|  | **Social participation frequency** | **~~.481~~** | **6** | **.06** |
|  | **Age** | **~~.743~~** | **7** | **.07** |
|  | **Education** | **~~.756~~** | **8** | **.08** |
|  | **Mastectomy** | **~~.878~~** | **9** | **.09** |
|  | **Employment** | **~~.889~~** | **10** | **.10** |

************** TOTAL, DIRECT, AND INDIRECT EFFECTS OF X ON Y **************

Total effect of X on Y

Effect se t p LLCI ULCI c_ps c_cs

-.01386 .00377 -3.67144 .00031 -.02130 -.00642 -.04891 -.24081

Direct effect of X on Y

Effect se t p LLCI ULCI c'_ps c'_cs

-.01196 .00384 -3.11504 .00210 -.01953 -.00439 -.04222 -.20784

Indirect effect(s) of X on Y:

Effect BootSE BootLLCI BootULCI

Manageab -.00190 .00098 -.00397 -.00019

Partially standardized indirect effect(s) of X on Y:

Effect BootSE BootLLCI BootULCI

Manageab -.00670 .00345 -.01397 -.00071

Completely standardized indirect effect(s) of X on Y:

Effect BootSE BootLLCI BootULCI

Manageab -.03297 .01714 -.06927 -.00340

*********************** ANALYSIS NOTES AND ERRORS ************************

Level of confidence for all confidence intervals in output:

95.0000

Number of bootstrap samples for percentile bootstrap confidence intervals:

5000

***************** PROCESS Procedure for SPSS Version 3.5 *****************

Written by Andrew F. Hayes, Ph.D. www.afhayes.com

Documentation available in Hayes (2018). www.guilford.com/p/hayes3

**************************************************************************

**Model : 4**

**Y : SWB = Social Well-being**

**X : Stigma_IV**

**M : Meaningfulness**

Covariates:

Age_cov Educatio Employme Social_p Insuranc Major_Ps Chemo_co Mastecto Radiothe

Sample

Size: 221

**************************************************************************

OUTCOME VARIABLE:

Meaningf

Model Summary

R R-sq MSE F df1 df2 p

.43435 .18866 20.31616 4.88319 10.00000 210.00000 .00000

Model

coeff se t p LLCI ULCI

constant 22.12763 2.67375 8.27588 .00000 16.85680 27.39846

Stigma_I -.23369 .06482 -3.60503 .00039 -.36147 -.10590

Age_cov .05889 .03442 1.71069 .08861 -.00897 .12674

Educatio -1.59914 .92285 -1.73283 .08459 -3.41839 .22010

Employme .60739 .97747 .62139 .53502 -1.31953 2.53430

Social_p .53412 .21518 2.48215 .01384 .10992 .95831

Insuranc .02574 1.08506 .02372 .98110 -2.11327 2.16475

Major_Ps -1.33952 .73036 -1.83404 .06806 -2.77930 .10026

Chemo_co -.26584 .45987 -.57808 .56383 -1.17240 .64071

Mastecto -.84146 .43347 -1.94122 .05357 -1.69596 .01305

Radiothe .61470 .78626 .78180 .43521 -.93528 2.16468

Standardized coefficients

coeff

Stigma_I -.23532

Age_cov .10993

Educatio -.12862

Employme .04649

Social_p .15739

Insuranc .00151

Major_Ps -.11725

Chemo_co -.04631

Mastecto -.13858

Radiothe .06169

| **Benjamini-Hochberg Procedure** | | | | |
| --- | --- | --- | --- | --- |
|  |  | **Ascending P-value s** | **I = ranking** | **(I/10)* 0.10** |
|  | **Stigma** | **.00039** | **1** | **.01** |
|  | **Social participation frequency** | **.013** | **2** | **.02** |
|  | **Mastectomy** | **~~.053~~** | **3** | **.03** |
|  | **Major Psychological issues** | **~~.068~~** | **4** | **.04** |
|  | **Age** | **~~.088~~** | **5** | **.05** |
|  | **Education** | **~~.084~~** | **6** | **.06** |
|  | **Radiotherapy** | **~~.435~~** | **7** | **.07** |
|  | **Employment** | **~~.535~~** | **8** | **.08** |
|  | **Chemotherapy** | **~~.563~~** | **9** | **.09** |
|  | **Insurance** | **~~.981~~** | **10** | **.10** |

**************************************************************************

OUTCOME VARIABLE:

SWBwn

Model Summary

R R-sq MSE F df1 df2 p

.48031 .23070 .06500 5.69767 11.00000 209.00000 .00000

Model

coeff se t p LLCI ULCI

constant .25187 .17416 1.44622 .14961 -.09146 .59519

Stigma_I -.01050 .00378 -2.77921 .00595 -.01795 -.00305

Meaningf .01437 .00390 3.68050 .00030 .00667 .02206

Age_cov -.00150 .00196 -.76647 .44426 -.00537 .00236

Educatio .00627 .05257 .11936 .90511 -.09736 .10991

Employme -.00084 .05534 -.01523 .98786 -.10994 .10825

Social_p .00116 .01235 .09371 .92543 -.02319 .02550

Insuranc .08552 .06137 1.39352 .16494 -.03546 .20651

Major_Ps -.14218 .04164 -3.41449 .00077 -.22427 -.06009

Chemo_co -.03308 .02603 -1.27068 .20526 -.08440 .01824

Mastecto .01595 .02474 .64482 .51975 -.03281 .06472

Radiothe .08613 .04454 1.93400 .05446 -.00166 .17393

Standardized coefficients

coeff

Stigma_I -.18247

Meaningf .24790

Age_cov -.04841

Educatio .00871

Employme -.00111

Social_p .00588

Insuranc .08680

Major_Ps -.21477

Chemo_co -.09943

Mastecto .04533

Radiothe .14917

| **Benjamini-Hochberg Procedure** | | | | |
| --- | --- | --- | --- | --- |
|  |  | **Ascending P-value s** | **I = ranking** | **(I/11)* 0.10** |
|  | **Major Psychological issues** | **.0007** | **1** | **.009** |
|  | **Stigma** | **.0059** | **2** | **.018** |
|  | **Meaningfulness** | **.0003** | **3** | **.027** |
|  | **Radiotherapy** | **~~.054~~** | **4** | **.03636** |
|  | **Insurance** | **~~.165~~** | **5** | **.045** |
|  | **Chemotherapy** | **~~.205~~** | **6** | **.054** |
|  | **Age** | **~~.444~~** | **7** | **.063** |
|  | **Mastectomy** | **~~.519~~** | **8** | **.072** |
|  | **Education** | **~~.905~~** | **9** | **.081** |
|  | **Social participation frequency** | **~~.925~~** | **10** | **.0909** |
|  | **Employment** | **~~.987~~** | **11** | **.100** |

************************** TOTAL EFFECT MODEL ****************************

OUTCOME VARIABLE:

SWBwn

Model Summary

R R-sq MSE F df1 df2 p

.42525 .18084 .06888 4.63587 10.00000 210.00000 .00001

Model

coeff se t p LLCI ULCI

constant .56974 .15568 3.65961 .00032 .26284 .87664

Stigma_I -.01386 .00377 -3.67144 .00031 -.02130 -.00642

Age_cov -.00066 .00200 -.32767 .74349 -.00461 .00329

Educatio -.01670 .05373 -.31075 .75630 -.12263 .08923

Employme .00788 .05691 .13850 .88998 -.10432 .12008

Social_p .00883 .01253 .70475 .48175 -.01587 .03353

Insuranc .08589 .06318 1.35953 .17544 -.03865 .21044

Major_Ps -.16142 .04253 -3.79580 .00019 -.24526 -.07759

Chemo_co -.03690 .02678 -1.37795 .16969 -.08968 .01589

Mastecto .00386 .02524 .15305 .87851 -.04589 .05362

Radiothe .09496 .04578 2.07431 .03927 .00471 .18521

Standardized coefficients

coeff

Stigma_I -.24081

Age_cov -.02116

Educatio -.02318

Employme .01041

Social_p .04490

Insuranc .08718

Major_Ps -.24383

Chemo_co -.11091

Mastecto .01098

Radiothe .16446

| **Benjamini-Hochberg Procedure** | | | | |
| --- | --- | --- | --- | --- |
|  |  | **Ascending P-value s** | **I = ranking** | **(I/10)* 0.10** |
|  | **Major Psychological issues** | **.00019** | **1** | **.01** |
|  | **Stigma** | **.00031** | **2** | **.02** |
|  | **Radiotherapy** | **~~.039~~** | **3** | **.03** |
|  | **Insurance** | **~~.175~~** | **4** | **.04** |
|  | **Chemotherapy** | **~~.169~~** | **5** | **.05** |
|  | **Social participation frequency** | **~~.481~~** | **6** | **.06** |
|  | **Age** | **~~.743~~** | **7** | **.07** |
|  | **Education** | **~~.756~~** | **8** | **.08** |
|  | **Mastectomy** | **~~.878~~** | **9** | **.09** |
|  | **Employment** | **~~.889~~** | **10** | **.10** |

************** TOTAL, DIRECT, AND INDIRECT EFFECTS OF X ON Y **************

Total effect of X on Y

Effect se t p LLCI ULCI c_ps c_cs

-.01386 .00377 -3.67144 .00031 -.02130 -.00642 -.04891 -.24081

Direct effect of X on Y

Effect se t p LLCI ULCI c'_ps c'_cs

-.01050 .00378 -2.77921 .00595 -.01795 -.00305 -.03706 -.18247

Indirect effect(s) of X on Y:

Effect BootSE BootLLCI BootULCI

Meaningf -.00336 .00130 -.00625 -.00116

Partially standardized indirect effect(s) of X on Y:

Effect BootSE BootLLCI BootULCI

Meaningf -.01185 .00456 -.02214 -.00412

Completely standardized indirect effect(s) of X on Y:

Effect BootSE BootLLCI BootULCI

Meaningf -.05834 .02261 -.10895 -.01999

*********************** ANALYSIS NOTES AND ERRORS ************************

Level of confidence for all confidence intervals in output:

95.0000

Number of bootstrap samples for percentile bootstrap confidence intervals:

5000

***************** PROCESS Procedure for SPSS Version 3.5 *****************

Written by Andrew F. Hayes, Ph.D. www.afhayes.com

Documentation available in Hayes (2018). www.guilford.com/p/hayes3

**************************************************************************

**Model : 4**

**Y : SWB = Social Well-being**

**X : Stigma_IV**

**M : SOC = Sense of Coherence**

Covariates:

Age_cov Educatio Employme Social_p Insuranc Major_Ps Chemo_co Mastecto Radiothe

Sample

Size: 221

**************************************************************************

OUTCOME VARIABLE:

SOC

Model Summary

R R-sq MSE F df1 df2 p

.45548 .20746 151.84085 5.49712 10.00000 210.00000 .00000

Model

coeff se t p LLCI ULCI

constant 68.01609 7.30961 9.30502 .00000 53.60647 82.42570

Stigma_I -.82220 .17721 -4.63961 .00001 -1.17154 -.47285

Age_cov .16923 .09410 1.79834 .07356 -.01628 .35474

Educatio -.43019 2.52293 -.17051 .86477 -5.40371 4.54333

Employme 3.33713 2.67225 1.24881 .21313 -1.93075 8.60501

Social_p 1.41471 .58828 2.40485 .01705 .25503 2.57440

Insuranc -1.62359 2.96639 -.54733 .58473 -7.47131 4.22413

Major_Ps -4.96124 1.99669 -2.48473 .01375 -8.89738 -1.02511

Chemo_co -1.48699 1.25722 -1.18276 .23824 -3.96537 .99139

Mastecto -2.99414 1.18503 -2.52663 .01225 -5.33022 -.65805

Radiothe -.38276 2.14952 -.17807 .85884 -4.62015 3.85464

Standardized coefficients

coeff

Stigma_I -.29932

Age_cov .11422

Educatio -.01251

Employme .09234

Social_p .15071

Insuranc -.03452

Major_Ps -.15700

Chemo_co -.09364

Mastecto -.17827

Radiothe -.01389

| **Benjamini-Hochberg Procedure** | | | | |
| --- | --- | --- | --- | --- |
|  |  | **Ascending P-value s** | **I = ranking** | **(I/10)* 0.10** |
|  | **Stigma** | **.00001** | **1** | **.01** |
|  | **Mastectomy** | **.012** | **2** | **.02** |
|  | **Major Psychological issues** | **.0137** | **3** | **.03** |
|  | **Social participation frequency** | **.017** | **4** | **.04** |
|  | **Age** | **~~.073~~** | **5** | **.05** |
|  | **Employment** | **~~.213~~** | **6** | **.06** |
|  | **Chemotherapy** | **~~.238~~** | **7** | **.07** |
|  | **Insurance** | **~~.584~~** | **8** | **.08** |
|  | **Radiotherapy** | **~~.858~~** | **9** | **.09** |
|  | **Education** | **~~.864~~** | **10** | **.10** |

**************************************************************************

OUTCOME VARIABLE:

SWBwn

Model Summary

R R-sq MSE F df1 df2 p

.46276 .21414 .06639 5.17748 11.00000 209.00000 .00000

Model

coeff se t p LLCI ULCI

constant .27762 .18165 1.52836 .12793 -.08047 .63572

Stigma_I -.01033 .00389 -2.65388 .00857 -.01800 -.00266

SOC .00429 .00144 2.97636 .00326 .00145 .00714

Age_cov -.00138 .00198 -.69775 .48611 -.00529 .00253

Educatio -.01485 .05276 -.28147 .77863 -.11886 .08916

Employme -.00645 .05609 -.11500 .90856 -.11702 .10412

Social_p .00275 .01247 .22086 .82541 -.02183 .02734

Insuranc .09287 .06207 1.49608 .13614 -.02950 .21524

Major_Ps -.14011 .04236 -3.30757 .00111 -.22363 -.05660

Chemo_co -.03051 .02638 -1.15672 .24871 -.08251 .02149

Mastecto .01672 .02515 .66480 .50691 -.03287 .06631

Radiothe .09661 .04495 2.14918 .03277 .00799 .18522

Standardized coefficients

coeff

Stigma_I -.17944

SOC .20501

Age_cov -.04457

Educatio -.02061

Employme -.00852

Social_p .01400

Insuranc .09426

Major_Ps -.21165

Chemo_co -.09171

Mastecto .04753

Radiothe .16731

| **Benjamini-Hochberg Procedure** | | | | |
| --- | --- | --- | --- | --- |
|  |  | **Ascending P-value s** | **I = ranking** | **(I/11)* 0.10** |
|  | **Major Psychological issues** | **.001** | **1** | **.009** |
|  | **Sense of Coherence** | **.0032** | **2** | **.018** |
|  | **Stigma** | **.0085** | **3** | **.027** |
|  | **Radiotherapy** | **.0327** | **4** | **.03636** |
|  | **Insurance** | **~~.136~~** | **5** | **.045** |
|  | **Chemotherapy** | **~~.248~~** | **6** | **.054** |
|  | **Age** | **~~486~~** | **7** | **.063** |
|  | **Mastectomy** | **~~.506~~** | **8** | **.072** |
|  | **Education** | **~~.778~~** | **9** | **.081** |
|  | **Social participation frequency** | **~~.825~~** | **10** | **.0909** |
|  | **Employment** | **~~.908~~** | **11** | **.100** |

************************** TOTAL EFFECT MODEL ****************************

OUTCOME VARIABLE:

SWBwn

Model Summary

R R-sq MSE F df1 df2 p

.42525 .18084 .06888 4.63587 10.00000 210.00000 .00001

Model

coeff se t p LLCI ULCI

constant .56974 .15568 3.65961 .00032 .26284 .87664

Stigma_I -.01386 .00377 -3.67144 .00031 -.02130 -.00642

Age_cov -.00066 .00200 -.32767 .74349 -.00461 .00329

Educatio -.01670 .05373 -.31075 .75630 -.12263 .08923

Employme .00788 .05691 .13850 .88998 -.10432 .12008

Social_p .00883 .01253 .70475 .48175 -.01587 .03353

Insuranc .08589 .06318 1.35953 .17544 -.03865 .21044

Major_Ps -.16142 .04253 -3.79580 .00019 -.24526 -.07759

Chemo_co -.03690 .02678 -1.37795 .16969 -.08968 .01589

Mastecto .00386 .02524 .15305 .87851 -.04589 .05362

Radiothe .09496 .04578 2.07431 .03927 .00471 .18521

Standardized coefficients

coeff

Stigma_I -.24081

Age_cov -.02116

Educatio -.02318

Employme .01041

Social_p .04490

Insuranc .08718

Major_Ps -.24383

Chemo_co -.11091

Mastecto .01098

Radiothe .16446

| **Benjamini-Hochberg Procedure** | | | | |
| --- | --- | --- | --- | --- |
|  |  | **Ascending P-value s** | **I = ranking** | **(I/10)* 0.10** |
|  | **Major Psychological issues** | **.00019** | **1** | **.01** |
|  | **Stigma** | **.00031** | **2** | **.02** |
|  | **Radiotherapy** | **~~.039~~** | **3** | **.03** |
|  | **Insurance** | **~~.175~~** | **4** | **.04** |
|  | **Chemotherapy** | **~~.169~~** | **5** | **.05** |
|  | **Social participation frequency** | **~~.481~~** | **6** | **.06** |
|  | **Age** | **~~.743~~** | **7** | **.07** |
|  | **Education** | **~~.756~~** | **8** | **.08** |
|  | **Mastectomy** | **~~.878~~** | **9** | **.09** |
|  | **Employment** | **~~.889~~** | **10** | **.10** |

************** TOTAL, DIRECT, AND INDIRECT EFFECTS OF X ON Y **************

Total effect of X on Y

Effect se t p LLCI ULCI c_ps c_cs

-.01386 .00377 -3.67144 .00031 -.02130 -.00642 -.04891 -.24081

Direct effect of X on Y

Effect se t p LLCI ULCI c'_ps c'_cs

-.01033 .00389 -2.65388 .00857 -.01800 -.00266 -.03645 -.17944

Indirect effect(s) of X on Y:

Effect BootSE BootLLCI BootULCI

SOC -.00353 .00132 -.00627 -.00115

Partially standardized indirect effect(s) of X on Y:

Effect BootSE BootLLCI BootULCI

SOC -.01246 .00462 -.02223 -.00410

Completely standardized indirect effect(s) of X on Y:

Effect BootSE BootLLCI BootULCI

SOC -.06136 .02263 -.10759 -.02016

*********************** ANALYSIS NOTES AND ERRORS ************************

Level of confidence for all confidence intervals in output:

95.0000

Number of bootstrap samples for percentile bootstrap confidence intervals:

5000

***************** PROCESS Procedure for SPSS Version 3.5 *****************

Written by Andrew F. Hayes, Ph.D. www.afhayes.com

Documentation available in Hayes (2018). www.guilford.com/p/hayes3

**************************************************************************

**Model : 4**

**Y : SWB = Social Well-being**

**X : Stigma_IV**

**M : Emoinfo = Emotional Informational social support**

Covariates:

Age_cov Educatio Employme Social_p Insuranc Major_Ps Chemo_co Mastecto Radiothe

Sample

Size: 221

**************************************************************************

OUTCOME VARIABLE:

Emoinfo

Model Summary

R R-sq MSE F df1 df2 p

.36001 .12960 .06415 3.12695 10.00000 210.00000 .00096

Model

coeff se t p LLCI ULCI

constant .51430 .15025 3.42300 .00074 .21811 .81049

Stigma_I -.01133 .00364 -3.10925 .00214 -.01851 -.00414

Age_cov .00164 .00193 .84938 .39664 -.00217 .00546

Educatio -.01597 .05186 -.30795 .75843 -.11820 .08626

Employme -.01900 .05493 -.34599 .72969 -.12728 .08928

Social_p .00346 .01209 .28655 .77474 -.02037 .02730

Insuranc .04089 .06097 .67054 .50325 -.07931 .16108

Major_Ps -.13273 .04104 -3.23408 .00142 -.21364 -.05183

Chemo_co -.00108 .02584 -.04187 .96664 -.05202 .04986

Mastecto -.01604 .02436 -.65839 .51101 -.06406 .03198

Radiothe .03875 .04418 .87701 .38148 -.04835 .12585

Standardized coefficients

coeff

Stigma_I -.21022

Age_cov .05653

Educatio -.02368

Employme -.02681

Social_p .01882

Insuranc .04432

Major_Ps -.21415

Chemo_co -.00347

Mastecto -.04868

Radiothe .07168

| **Benjamini-Hochberg Procedure** | | | | |
| --- | --- | --- | --- | --- |
|  |  | **Ascending P-value s** | **I = ranking** | **(I/10)* 0.10** |
|  | **Major Psychological issues** | **.0014** | **1** | **.01** |
|  | **Stigma** | **.0021** | **2** | **.02** |
|  | **Radiotherapy** | **~~.381~~** | **3** | **.03** |
|  | **Age** | **~~.396~~** | **4** | **.04** |
|  | **Insurance** | **~~.503~~** | **5** | **.05** |
|  | **Mastectomy** | **~~.511~~** | **6** | **.06** |
|  | **Employment** | **~~.729~~** | **7** | **.07** |
|  | **Education** | **~~.758~~** | **8** | **.08** |
|  | **Social participation frequency** | **~~.774~~** | **9** | **.09** |
|  | **Chemotherapy** | **~~.966~~** | **10** | **.10** |

**************************************************************************

OUTCOME VARIABLE:

SWBwn

Model Summary

R R-sq MSE F df1 df2 p

.51974 .27013 .06166 7.03201 11.00000 209.00000 .00000

Model

coeff se t p LLCI ULCI

constant .39380 .15136 2.60175 .00994 .09541 .69218

Stigma_I -.00998 .00365 -2.73313 .00681 -.01718 -.00278

Emoinfo .34211 .06765 5.05663 .00000 .20873 .47548

Age_cov -.00122 .00190 -.64159 .52184 -.00496 .00253

Educatio -.01123 .05085 -.22092 .82537 -.11149 .08902

Employme .01438 .05387 .26703 .78971 -.09181 .12058

Social_p .00764 .01186 .64472 .51982 -.01573 .03102

Insuranc .07191 .05984 1.20160 .23088 -.04607 .18988

Major_Ps -.11601 .04123 -2.81398 .00536 -.19729 -.03474

Chemo_co -.03653 .02534 -1.44171 .15088 -.08647 .01342

Mastecto .00935 .02391 .39109 .69613 -.03778 .05648

Radiothe .08171 .04340 1.88283 .06111 -.00384 .16726

Standardized coefficients

coeff

Stigma_I -.17348

Emoinfo .32030

Age_cov -.03927

Educatio -.01559

Employme .01900

Social_p .03887

Insuranc .07298

Major_Ps -.17524

Chemo_co -.10980

Mastecto .02657

Radiothe .14150

| **Benjamini-Hochberg Procedure** | | | | |
| --- | --- | --- | --- | --- |
|  |  | **Ascending P-value s** | **I = ranking** | **(I/11)* 0.10** |
|  | **Emotional Informational** | **.0000** | **1** | **.009** |
|  | **Major Psychological issues** | **.005** | **2** | **.018** |
|  | **Stigma** | **.0068** | **3** | **.027** |
|  | **Radiotherapy** | **~~.061~~** | **4** | **.03636** |
|  | **Chemotherapy** | **~~.150~~** | **5** | **.045** |
|  | **Insurance** | **~~.230~~** | **6** | **.054** |
|  | **Social participation frequency** | **~~.519~~** | **7** | **.063** |
|  | **Age** | **~~.521~~** | **8** | **.072** |
|  | **Mastectomy** | **~~.696~~** | **9** | **.081** |
|  | **Employment** | **~~.789~~** | **10** | **.0909** |
|  | **Education** | **~~.825~~** | **11** | **.100** |

************************** TOTAL EFFECT MODEL ****************************

OUTCOME VARIABLE:

SWBwn

Model Summary

R R-sq MSE F df1 df2 p

.42525 .18084 .06888 4.63587 10.00000 210.00000 .00001

Model

coeff se t p LLCI ULCI

constant .56974 .15568 3.65961 .00032 .26284 .87664

Stigma_I -.01386 .00377 -3.67144 .00031 -.02130 -.00642

Age_cov -.00066 .00200 -.32767 .74349 -.00461 .00329

Educatio -.01670 .05373 -.31075 .75630 -.12263 .08923

Employme .00788 .05691 .13850 .88998 -.10432 .12008

Social_p .00883 .01253 .70475 .48175 -.01587 .03353

Insuranc .08589 .06318 1.35953 .17544 -.03865 .21044

Major_Ps -.16142 .04253 -3.79580 .00019 -.24526 -.07759

Chemo_co -.03690 .02678 -1.37795 .16969 -.08968 .01589

Mastecto .00386 .02524 .15305 .87851 -.04589 .05362

Radiothe .09496 .04578 2.07431 .03927 .00471 .18521

Standardized coefficients

coeff

Stigma_I -.24081

Age_cov -.02116

Educatio -.02318

Employme .01041

Social_p .04490

Insuranc .08718

Major_Ps -.24383

Chemo_co -.11091

Mastecto .01098

Radiothe .16446

| **Benjamini-Hochberg Procedure** | | | | |
| --- | --- | --- | --- | --- |
|  |  | **Ascending P-value s** | **I = ranking** | **(I/10)* 0.10** |
|  | **Major Psychological issues** | **.00019** | **1** | **.01** |
|  | **Stigma** | **.00031** | **2** | **.02** |
|  | **Radiotherapy** | **~~.039~~** | **3** | **.03** |
|  | **Insurance** | **~~.175~~** | **4** | **.04** |
|  | **Chemotherapy** | **~~.169~~** | **5** | **.05** |
|  | **Social participation frequency** | **~~.481~~** | **6** | **.06** |
|  | **Age** | **~~.743~~** | **7** | **.07** |
|  | **Education** | **~~.756~~** | **8** | **.08** |
|  | **Mastectomy** | **~~.878~~** | **9** | **.09** |
|  | **Employment** | **~~.889~~** | **10** | **.10** |

************** TOTAL, DIRECT, AND INDIRECT EFFECTS OF X ON Y **************

Total effect of X on Y

Effect se t p LLCI ULCI c_ps c_cs

-.01386 .00377 -3.67144 .00031 -.02130 -.00642 -.04891 -.24081

Direct effect of X on Y

Effect se t p LLCI ULCI c'_ps c'_cs

-.00998 .00365 -2.73313 .00681 -.01718 -.00278 -.03524 -.17348

Indirect effect(s) of X on Y:

Effect BootSE BootLLCI BootULCI

Emoinfo -.00387 .00136 -.00670 -.00142

Partially standardized indirect effect(s) of X on Y:

Effect BootSE BootLLCI BootULCI

Emoinfo -.01368 .00467 -.02328 -.00514

Completely standardized indirect effect(s) of X on Y:

Effect BootSE BootLLCI BootULCI

Emoinfo -.06733 .02350 -.11576 -.02481

*********************** ANALYSIS NOTES AND ERRORS ************************

Level of confidence for all confidence intervals in output:

95.0000

Number of bootstrap samples for percentile bootstrap confidence intervals:

5000

***************** PROCESS Procedure for SPSS Version 3.5 *****************

Written by Andrew F. Hayes, Ph.D. www.afhayes.com

Documentation available in Hayes (2018). www.guilford.com/p/hayes3

**************************************************************************

**Model : 4**

**Y : SWB = Social Well-being**

**X : Stigma_IV**

**M : Affectionate support**

Covariates:

Age_cov Educatio Employme Social_p Insuranc Major_Ps Chemo_co Mastecto Radiothe

Sample

Size: 221

**************************************************************************

OUTCOME VARIABLE:

Affectio

Model Summary

R R-sq MSE F df1 df2 p

.37797 .14286 .05276 3.50013 10.00000 210.00000 .00027

Model

coeff se t p LLCI ULCI

constant .48253 .13625 3.54144 .00049 .21393 .75112

Stigma_I -.00910 .00330 -2.75500 .00638 -.01561 -.00259

Age_cov .00105 .00175 .59605 .55178 -.00241 .00450

Educatio -.01455 .04703 -.30944 .75729 -.10726 .07815

Employme -.02925 .04981 -.58719 .55770 -.12744 .06894

Social_p .00296 .01097 .27031 .78719 -.01865 .02458

Insuranc .07932 .05529 1.43455 .15290 -.02968 .18832

Major_Ps -.11830 .03722 -3.17845 .00170 -.19167 -.04493

Chemo_co -.00828 .02343 -.35349 .72408 -.05448 .03791

Mastecto -.03515 .02209 -1.59143 .11302 -.07870 .00839

Radiothe .05027 .04007 1.25454 .21104 -.02872 .12925

Standardized coefficients

coeff

Stigma_I -.18484

Age_cov .03937

Educatio -.02361

Employme -.04515

Social_p .01762

Insuranc .09410

Major_Ps -.20885

Chemo_co -.02910

Mastecto -.11678

Radiothe .10175

| **Benjamini-Hochberg Procedure** | | | | |
| --- | --- | --- | --- | --- |
|  |  | **Ascending P-value s** | **I = ranking** | **(I/10)* 0.10** |
|  | **Major Psychological issues** | **.0017** | **1** | **.01** |
|  | **Stigma** | **.0063** | **2** | **.02** |
|  | **Mastectomy** | **~~.113~~** | **3** | **.03** |
|  | **Insurance** | **~~.152~~** | **4** | **.04** |
|  | **Radiotherapy** | **~~.211~~** | **5** | **.05** |
|  | **Age** | **~~.551~~** | **6** | **.06** |
|  | **Employment** | **~~.557~~** | **7** | **.07** |
|  | **Chemotherapy** | **~~.724~~** | **8** | **.08** |
|  | **Education** | **~~.757~~** | **9** | **.09** |
|  | **Social participation frequency** | **~~.787~~** | **10** | **.10** |

**************************************************************************

OUTCOME VARIABLE:

SWBwn

Model Summary

R R-sq MSE F df1 df2 p

.52671 .27742 .06105 7.29466 11.00000 209.00000 .00000

Model

coeff se t p LLCI ULCI

constant .38042 .15088 2.52136 .01244 .08298 .67787

Stigma_I -.01029 .00362 -2.84402 .00490 -.01742 -.00316

Affectio .39235 .07423 5.28547 .00000 .24601 .53868

Age_cov -.00107 .00189 -.56497 .57270 -.00479 .00266

Educatio -.01099 .05060 -.21716 .82830 -.11074 .08876

Employme .01936 .05363 .36098 .71848 -.08636 .12508

Social_p .00767 .01180 .64988 .51649 -.01559 .03092

Insuranc .05477 .05977 .91639 .36052 -.06306 .17260

Major_Ps -.11501 .04099 -2.80591 .00549 -.19581 -.03421

Chemo_co -.03365 .02522 -1.33433 .18355 -.08336 .01606

Mastecto .01766 .02390 .73857 .46100 -.02947 .06478

Radiothe .07524 .04326 1.73925 .08346 -.01004 .16053

Standardized coefficients

coeff

Stigma_I -.17876

Affectio .33568

Age_cov -.03437

Educatio -.01525

Employme .02557

Social_p .03899

Insuranc .05559

Major_Ps -.17372

Chemo_co -.10114

Mastecto .05018

Radiothe .13031

| **Benjamini-Hochberg Procedure** | | | | |
| --- | --- | --- | --- | --- |
|  |  | **Ascending P-value s** | **I = ranking** | **(I/11)* 0.10** |
|  | **Affectionate** | **.0000** | **1** | **.009** |
|  | **Stigma** | **.0049** | **2** | **.018** |
|  | **Major Psychological issues** | **.005** | **3** | **.027** |
|  | **Radiotherapy** | **~~.083~~** | **4** | **.03636** |
|  | **Chemotherapy** | **~~.183~~** | **5** | **.045** |
|  | **Insurance** | **~~.360~~** | **6** | **.054** |
|  | **Mastectomy** | **~~.468~~** | **7** | **.063** |
|  | **Social participation frequency** | **~~.516~~** | **8** | **.072** |
|  | **Age** | **~~.572~~** | **9** | **.081** |
|  | **Employment** | **~~.718~~** | **10** | **.0909** |
|  | **Education** | **~~.828~~** | **11** | **.100** |

************************** TOTAL EFFECT MODEL ****************************

OUTCOME VARIABLE:

SWBwn

Model Summary

R R-sq MSE F df1 df2 p

.42525 .18084 .06888 4.63587 10.00000 210.00000 .00001

Model

coeff se t p LLCI ULCI

constant .56974 .15568 3.65961 .00032 .26284 .87664

Stigma_I -.01386 .00377 -3.67144 .00031 -.02130 -.00642

Age_cov -.00066 .00200 -.32767 .74349 -.00461 .00329

Educatio -.01670 .05373 -.31075 .75630 -.12263 .08923

Employme .00788 .05691 .13850 .88998 -.10432 .12008

Social_p .00883 .01253 .70475 .48175 -.01587 .03353

Insuranc .08589 .06318 1.35953 .17544 -.03865 .21044

Major_Ps -.16142 .04253 -3.79580 .00019 -.24526 -.07759

Chemo_co -.03690 .02678 -1.37795 .16969 -.08968 .01589

Mastecto .00386 .02524 .15305 .87851 -.04589 .05362

Radiothe .09496 .04578 2.07431 .03927 .00471 .18521

Standardized coefficients

coeff

Stigma_I -.24081

Age_cov -.02116

Educatio -.02318

Employme .01041

Social_p .04490

Insuranc .08718

Major_Ps -.24383

Chemo_co -.11091

Mastecto .01098

Radiothe .16446

| **Benjamini-Hochberg Procedure** | | | | |
| --- | --- | --- | --- | --- |
|  |  | **Ascending P-value s** | **I = ranking** | **(I/10)* 0.10** |
|  | **Major Psychological issues** | **.00019** | **1** | **.01** |
|  | **Stigma** | **.00031** | **2** | **.02** |
|  | **Radiotherapy** | **~~.039~~** | **3** | **.03** |
|  | **Insurance** | **~~.175~~** | **4** | **.04** |
|  | **Chemotherapy** | **~~.169~~** | **5** | **.05** |
|  | **Social participation frequency** | **~~.481~~** | **6** | **.06** |
|  | **Age** | **~~.743~~** | **7** | **.07** |
|  | **Education** | **~~.756~~** | **8** | **.08** |
|  | **Mastectomy** | **~~.878~~** | **9** | **.09** |
|  | **Employment** | **~~.889~~** | **10** | **.10** |

************** TOTAL, DIRECT, AND INDIRECT EFFECTS OF X ON Y **************

Total effect of X on Y

Effect se t p LLCI ULCI c_ps c_cs

-.01386 .00377 -3.67144 .00031 -.02130 -.00642 -.04891 -.24081

Direct effect of X on Y

Effect se t p LLCI ULCI c'_ps c'_cs

-.01029 .00362 -2.84402 .00490 -.01742 -.00316 -.03631 -.17876

Indirect effect(s) of X on Y:

Effect BootSE BootLLCI BootULCI

Affectio -.00357 .00148 -.00662 -.00077

Partially standardized indirect effect(s) of X on Y:

Effect BootSE BootLLCI BootULCI

Affectio -.01260 .00513 -.02308 -.00276

Completely standardized indirect effect(s) of X on Y:

Effect BootSE BootLLCI BootULCI

Affectio -.06205 .02583 -.11442 -.01323

*********************** ANALYSIS NOTES AND ERRORS ************************

Level of confidence for all confidence intervals in output:

95.0000

Number of bootstrap samples for percentile bootstrap confidence intervals:

5000

***************** PROCESS Procedure for SPSS Version 3.5 *****************

Written by Andrew F. Hayes, Ph.D. www.afhayes.com

Documentation available in Hayes (2018). www.guilford.com/p/hayes3

**************************************************************************

**Model : 4**

**Y : SWB = Social Well-being**

**X : Stigma_IV**

**M : Tangible support**

Covariates:

Age_cov Educatio Employme Social_p Insuranc Major_Ps Chemo_co Mastecto Radiothe

Sample

Size: 221

**************************************************************************

OUTCOME VARIABLE:

Tangible

Model Summary

R R-sq MSE F df1 df2 p

.36445 .13282 .05649 3.21649 10.00000 210.00000 .00071

Model

coeff se t p LLCI ULCI

constant .39310 .14099 2.78817 .00579 .11516 .67103

Stigma_I -.00884 .00342 -2.58739 .01035 -.01558 -.00211

Age_cov .00193 .00182 1.06059 .29010 -.00165 .00550

Educatio .01051 .04866 .21603 .82918 -.08542 .10644

Employme .00466 .05154 .09047 .92800 -.09694 .10627

Social_p -.01229 .01135 -1.08312 .28000 -.03466 .01008

Insuranc .08211 .05722 1.43514 .15274 -.03068 .19490

Major_Ps -.12284 .03851 -3.18964 .00164 -.19876 -.04692

Chemo_co -.00498 .02425 -.20536 .83749 -.05278 .04282

Mastecto -.02154 .02286 -.94221 .34717 -.06659 .02352

Radiothe .05682 .04146 1.37039 .17203 -.02491 .13855

Standardized coefficients

coeff

Stigma_I -.17461

Age_cov .07046

Educatio .01658

Employme .00700

Social_p -.07100

Insuranc .09469

Major_Ps -.21081

Chemo_co -.01701

Mastecto -.06954

Radiothe .11179

| **Benjamini-Hochberg Procedure** | | | | |
| --- | --- | --- | --- | --- |
|  |  | **Ascending P-value s** | **I = ranking** | **(I/10)* 0.10** |
|  | **Major Psychological issues** | **.0016** | **1** | **.01** |
|  | **Stigma** | **.0103** | **2** | **.02** |
|  | **Insurance** | **~~.152~~** | **3** | **.03** |
|  | **Radiotherapy** | **~~.172~~** | **4** | **.04** |
|  | **Social participation frequency** | **~~.280~~** | **5** | **.05** |
|  | **Age** | **~~.290~~** | **6** | **.06** |
|  | **Mastectomy** | **~~.347~~** | **7** | **.07** |
|  | **Education** | **~~.829~~** | **8** | **.08** |
|  | **Chemotherapy** | **~~.837~~** | **9** | **.09** |
|  | **Employment** | **~~.928~~** | **10** | **.10** |

**************************************************************************

OUTCOME VARIABLE:

SWBwn

Model Summary

R R-sq MSE F df1 df2 p

.50623 .25627 .06283 6.54697 11.00000 209.00000 .00000

Model

coeff se t p LLCI ULCI

constant .43802 .15142 2.89265 .00422 .13950 .73653

Stigma_I -.01089 .00366 -2.97484 .00328 -.01811 -.00367

Tangible .33509 .07278 4.60422 .00001 .19162 .47857

Age_cov -.00130 .00192 -.67822 .49838 -.00509 .00248

Educatio -.02022 .05133 -.39394 .69403 -.12141 .08097

Employme .00632 .05436 .11626 .90756 -.10085 .11349

Social_p .01295 .01200 1.07898 .28184 -.01071 .03661

Insuranc .05838 .06064 .96273 .33680 -.06116 .17792

Major_Ps -.12026 .04159 -2.89153 .00424 -.20225 -.03827

Chemo_co -.03523 .02558 -1.37731 .16989 -.08565 .01519

Mastecto .01108 .02416 .45863 .64698 -.03654 .05870

Radiothe .07593 .04392 1.72866 .08535 -.01066 .16251

Standardized coefficients

coeff

Stigma_I -.18931

Tangible .29494

Age_cov -.04194

Educatio -.02807

Employme .00835

Social_p .06584

Insuranc .05925

Major_Ps -.18166

Chemo_co -.10589

Mastecto .03149

Radiothe .13149

| **Benjamini-Hochberg Procedure** | | | | |
| --- | --- | --- | --- | --- |
|  |  | **Ascending P-value s** | **I = ranking** | **(I/11)* 0.10** |
|  | **Tangible support** | **.00001** | **1** | **.009** |
|  | **Stigma** | **.0032** | **2** | **.018** |
|  | **Major Psychological issues** | **.004** | **3** | **.027** |
|  | **Radiotherapy** | **~~.0853~~** | **4** | **.03636** |
|  | **Chemotherapy** | **~~.169~~** | **5** | **.045** |
|  | **Social participation frequency** | **~~.281~~** | **6** | **.054** |
|  | **Insurance** | **~~.336~~** | **7** | **.063** |
|  | **Age** | **~~.498~~** | **8** | **.072** |
|  | **Mastectomy** | **~~.648~~** | **9** | **.081** |
|  | **Education** | **~~.694~~** | **10** | **.0909** |
|  | **Employment** | **~~.907~~** | **11** | **.100** |

************************** TOTAL EFFECT MODEL ****************************

OUTCOME VARIABLE:

SWBwn

Model Summary

R R-sq MSE F df1 df2 p

.42525 .18084 .06888 4.63587 10.00000 210.00000 .00001

Model

coeff se t p LLCI ULCI

constant .56974 .15568 3.65961 .00032 .26284 .87664

Stigma_I -.01386 .00377 -3.67144 .00031 -.02130 -.00642

Age_cov -.00066 .00200 -.32767 .74349 -.00461 .00329

Educatio -.01670 .05373 -.31075 .75630 -.12263 .08923

Employme .00788 .05691 .13850 .88998 -.10432 .12008

Social_p .00883 .01253 .70475 .48175 -.01587 .03353

Insuranc .08589 .06318 1.35953 .17544 -.03865 .21044

Major_Ps -.16142 .04253 -3.79580 .00019 -.24526 -.07759

Chemo_co -.03690 .02678 -1.37795 .16969 -.08968 .01589

Mastecto .00386 .02524 .15305 .87851 -.04589 .05362

Radiothe .09496 .04578 2.07431 .03927 .00471 .18521

Standardized coefficients

coeff

Stigma_I -.24081

Age_cov -.02116

Educatio -.02318

Employme .01041

Social_p .04490

Insuranc .08718

Major_Ps -.24383

Chemo_co -.11091

Mastecto .01098

Radiothe .16446

| **Benjamini-Hochberg Procedure** | | | | |
| --- | --- | --- | --- | --- |
|  |  | **Ascending P-value s** | **I = ranking** | **(I/10)* 0.10** |
|  | **Major Psychological issues** | **.00019** | **1** | **.01** |
|  | **Stigma** | **.00031** | **2** | **.02** |
|  | **Radiotherapy** | **~~.039~~** | **3** | **.03** |
|  | **Insurance** | **~~.175~~** | **4** | **.04** |
|  | **Chemotherapy** | **~~.169~~** | **5** | **.05** |
|  | **Social participation frequency** | **~~.481~~** | **6** | **.06** |
|  | **Age** | **~~.743~~** | **7** | **.07** |
|  | **Education** | **~~.756~~** | **8** | **.08** |
|  | **Mastectomy** | **~~.878~~** | **9** | **.09** |
|  | **Employment** | **~~.889~~** | **10** | **.10** |

************** TOTAL, DIRECT, AND INDIRECT EFFECTS OF X ON Y **************

Total effect of X on Y

Effect se t p LLCI ULCI c_ps c_cs

-.01386 .00377 -3.67144 .00031 -.02130 -.00642 -.04891 -.24081

Direct effect of X on Y

Effect se t p LLCI ULCI c'_ps c'_cs

-.01089 .00366 -2.97484 .00328 -.01811 -.00367 -.03845 -.18931

Indirect effect(s) of X on Y:

Effect BootSE BootLLCI BootULCI

Tangible -.00296 .00134 -.00592 -.00066

Partially standardized indirect effect(s) of X on Y:

Effect BootSE BootLLCI BootULCI

Tangible -.01046 .00463 -.02060 -.00241

Completely standardized indirect effect(s) of X on Y:

Effect BootSE BootLLCI BootULCI

Tangible -.05150 .02311 -.10366 -.01173

*********************** ANALYSIS NOTES AND ERRORS ************************

Level of confidence for all confidence intervals in output:

95.0000

Number of bootstrap samples for percentile bootstrap confidence intervals:

5000

***************** PROCESS Procedure for SPSS Version 3.5 *****************

Written by Andrew F. Hayes, Ph.D. www.afhayes.com

Documentation available in Hayes (2018). www.guilford.com/p/hayes3

**************************************************************************

**Model : 4**

**Y : SWB = Social Well-being**

**X : Stigma**

**M : Positive social Interaction**

Covariates:

Age_cov Educatio Employme Social_p Insuranc Major_Ps Chemo_co Mastecto Radiothe

Sample

Size: 221

**************************************************************************

OUTCOME VARIABLE:

Positive

Model Summary

R R-sq MSE F df1 df2 p

.34064 .11604 .05623 2.75668 10.00000 210.00000 .00325

Model

coeff se t p LLCI ULCI

constant .52551 .14067 3.73583 .00024 .24821 .80281

Stigma_I -.01295 .00341 -3.79812 .00019 -.01968 -.00623

Age_cov .00060 .00181 .33404 .73868 -.00297 .00417

Educatio .01962 .04855 .40417 .68650 -.07609 .11533

Employme .02577 .05143 .50115 .61679 -.07560 .12715

Social_p .00119 .01132 .10550 .91608 -.02112 .02351

Insuranc .05715 .05709 1.00117 .31790 -.05538 .16969

Major_Ps -.08447 .03842 -2.19820 .02903 -.16021 -.00872

Chemo_co -.00020 .02419 -.00842 .99329 -.04790 .04749

Mastecto -.00918 .02281 -.40250 .68773 -.05414 .03578

Radiothe .02742 .04137 .66295 .50809 -.05412 .10897

Standardized coefficients

coeff

Stigma_I -.25878

Age_cov .02241

Educatio .03131

Employme .03914

Social_p .00698

Insuranc .06669

Major_Ps -.14669

Chemo_co -.00070

Mastecto -.02999

Radiothe .05460

| **Benjamini-Hochberg Procedure** | | | | |
| --- | --- | --- | --- | --- |
|  |  | **Ascending P-value s** | **I = ranking** | **(I/10)* 0.10** |
|  | **Stigma** | **.00019** | **1** | **.01** |
|  | **Major Psychological issues** | **~~.029~~** | **2** | **.02** |
|  | **Insurance** | **~~.317~~** | **3** | **.03** |
|  | **Radiotherapy** | **~~.508~~** | **4** | **.04** |
|  | **Employment** | **~~.616~~** | **5** | **.05** |
|  | **Education** | **~~.686~~** | **6** | **.06** |
|  | **Mastectomy** | **~~.687~~** | **7** | **.07** |
|  | **Age** | **~~.738~~** | **8** | **.08** |
|  | **Social participation frequency** | **~~.916~~** | **9** | **.09** |
|  | **Chemotherapy** | **~~.993~~** | **10** | **.10** |

**************************************************************************

OUTCOME VARIABLE:

SWBwn

Model Summary

R R-sq MSE F df1 df2 p

.54631 .29845 .05927 8.08291 11.00000 209.00000 .00000

Model

coeff se t p LLCI ULCI

constant .34936 .14914 2.34249 .02010 .05535 .64337

Stigma_I -.00843 .00362 -2.32776 .02088 -.01556 -.00129

Positive .41937 .07085 5.91937 .00000 .27970 .55903

Age_cov -.00091 .00186 -.48954 .62497 -.00458 .00276

Educatio -.02493 .04987 -.49988 .61768 -.12323 .07338

Employme -.00293 .05283 -.05537 .95589 -.10707 .10122

Social_p .00833 .01162 .71661 .47442 -.01458 .03124

Insuranc .06193 .05875 1.05411 .29305 -.05389 .17774

Major_Ps -.12600 .03990 -3.15785 .00182 -.20466 -.04734

Chemo_co -.03681 .02484 -1.48199 .13985 -.08578 .01216

Mastecto .00771 .02342 .32927 .74228 -.03846 .05389

Radiothe .08346 .04251 1.96326 .05094 -.00035 .16727

Standardized coefficients

coeff

Stigma_I -.14641

Positive .36477

Age_cov -.02933

Educatio -.03460

Employme -.00386

Social_p .04236

Insuranc .06285

Major_Ps -.19033

Chemo_co -.11065

Mastecto .02192

Radiothe .14455

| **Benjamini-Hochberg Procedure** | | | | |
| --- | --- | --- | --- | --- |
|  |  | **Ascending P-value s** | **I = ranking** | **(I/11)* 0.10** |
|  | **Positive Social Interaction** | **.00000** | **1** | **.009** |
|  | **Major Psychological issues** | **.001** | **2** | **.018** |
|  | **Stigma** | **.0208** | **3** | **.027** |
|  | **Radiotherapy** | **~~.050~~** | **4** | **.03636** |
|  | **Chemotherapy** | **~~.139~~** | **5** | **.045** |
|  | **Insurance** | **~~.293~~** | **6** | **.054** |
|  | **Social participation frequency** | **~~.474~~** | **7** | **.063** |
|  | **Education** | **~~.617~~** | **8** | **.072** |
|  | **Age** | **~~.624~~** | **9** | **.081** |
|  | **Mastectomy** | **~~.742~~** | **10** | **.0909** |
|  | **Employment** | **~~.955~~** | **11** | **.100** |

************************** TOTAL EFFECT MODEL ****************************

OUTCOME VARIABLE:

SWBwn

Model Summary

R R-sq MSE F df1 df2 p

.42525 .18084 .06888 4.63587 10.00000 210.00000 .00001

Model

coeff se t p LLCI ULCI

constant .56974 .15568 3.65961 .00032 .26284 .87664

Stigma_I -.01386 .00377 -3.67144 .00031 -.02130 -.00642

Age_cov -.00066 .00200 -.32767 .74349 -.00461 .00329

Educatio -.01670 .05373 -.31075 .75630 -.12263 .08923

Employme .00788 .05691 .13850 .88998 -.10432 .12008

Social_p .00883 .01253 .70475 .48175 -.01587 .03353

Insuranc .08589 .06318 1.35953 .17544 -.03865 .21044

Major_Ps -.16142 .04253 -3.79580 .00019 -.24526 -.07759

Chemo_co -.03690 .02678 -1.37795 .16969 -.08968 .01589

Mastecto .00386 .02524 .15305 .87851 -.04589 .05362

Radiothe .09496 .04578 2.07431 .03927 .00471 .18521

Standardized coefficients

coeff

Stigma_I -.24081

Age_cov -.02116

Educatio -.02318

Employme .01041

Social_p .04490

Insuranc .08718

Major_Ps -.24383

Chemo_co -.11091

Mastecto .01098

Radiothe .16446

| **Benjamini-Hochberg Procedure** | | | | |
| --- | --- | --- | --- | --- |
|  |  | **Ascending P-value s** | **I = ranking** | **(I/10)* 0.10** |
|  | **Major Psychological issues** | **.00019** | **1** | **.01** |
|  | **Stigma** | **.00031** | **2** | **.02** |
|  | **Radiotherapy** | **~~.039~~** | **3** | **.03** |
|  | **Insurance** | **~~.175~~** | **4** | **.04** |
|  | **Chemotherapy** | **~~.169~~** | **5** | **.05** |
|  | **Social participation frequency** | **~~.481~~** | **6** | **.06** |
|  | **Age** | **~~.743~~** | **7** | **.07** |
|  | **Education** | **~~.756~~** | **8** | **.08** |
|  | **Mastectomy** | **~~.878~~** | **9** | **.09** |
|  | **Employment** | **~~.889~~** | **10** | **.10** |

************** TOTAL, DIRECT, AND INDIRECT EFFECTS OF X ON Y **************

Total effect of X on Y

Effect se t p LLCI ULCI c_ps c_cs

-.01386 .00377 -3.67144 .00031 -.02130 -.00642 -.04891 -.24081

Direct effect of X on Y

Effect se t p LLCI ULCI c'_ps c'_cs

-.00843 .00362 -2.32776 .02088 -.01556 -.00129 -.02974 -.14641

Indirect effect(s) of X on Y:

Effect BootSE BootLLCI BootULCI

Positive -.00543 .00161 -.00875 -.00252

Partially standardized indirect effect(s) of X on Y:

Effect BootSE BootLLCI BootULCI

Positive -.01917 .00543 -.03015 -.00908

Completely standardized indirect effect(s) of X on Y:

Effect BootSE BootLLCI BootULCI

Positive -.09440 .02769 -.15192 -.04297

*********************** ANALYSIS NOTES AND ERRORS ************************

Level of confidence for all confidence intervals in output:

95.0000

Number of bootstrap samples for percentile bootstrap confidence intervals:

5000

***************** PROCESS Procedure for SPSS Version 3.5 *****************

Written by Andrew F. Hayes, Ph.D. www.afhayes.com

Documentation available in Hayes (2018). www.guilford.com/p/hayes3

**************************************************************************

**Model : 4**

**Y : SWB = Social Well-being**

**X : Stigma_I**

**M : MOS_SSS**

Covariates:

Age_cov Educatio Employme Social_p Insuranc Major_Ps Chemo_co Mastecto Radiothe

Sample

Size: 221

**************************************************************************

OUTCOME VARIABLE:

MOS_SSS

Model Summary

R R-sq MSE F df1 df2 p

.35287 .12451 .06905 2.98667 10.00000 210.00000 .00153

Model

coeff se t p LLCI ULCI

constant .46456 .15587 2.98037 .00322 .15728 .77183

Stigma_I -.01095 .00378 -2.89783 .00416 -.01840 -.00350

Age_cov .00169 .00201 .84386 .39971 -.00226 .00565

Educatio -.03744 .05380 -.69584 .48730 -.14349 .06862

Employme -.02411 .05698 -.42304 .67270 -.13644 .08823

Social_p -.00090 .01254 -.07168 .94292 -.02563 .02383

Insuranc .07701 .06326 1.21750 .22478 -.04768 .20171

Major_Ps -.12186 .04258 -2.86202 .00464 -.20579 -.03792

Chemo_co -.00198 .02681 -.07371 .94131 -.05483 .05087

Mastecto -.01605 .02527 -.63501 .52612 -.06586 .03377

Radiothe .05665 .04584 1.23597 .21785 -.03371 .14701

Standardized coefficients

coeff

Stigma_I -.19649

Age_cov .05633

Educatio -.05365

Employme -.03288

Social_p -.00472

Insuranc .08071

Major_Ps -.19006

Chemo_co -.00613

Mastecto -.04709

Radiothe .10131

| **Benjamini-Hochberg Procedure** | | | | |
| --- | --- | --- | --- | --- |
|  |  | **Ascending P-value s** | **I = ranking** | **(I/10)* 0.10** |
|  | **Stigma** | **.0041** | **1** | **.01** |
|  | **Major Psychological issues** | **.0046** | **2** | **.02** |
|  | **Radiotherapy** | **~~.217~~** | **3** | **.03** |
|  | **Insurance** | **~~.224~~** | **4** | **.04** |
|  | **Age** | **~~.399~~** | **5** | **.05** |
|  | **Education** | **~~.487~~** | **6** | **.06** |
|  | **Mastectomy** | **~~.526~~** | **7** | **.07** |
|  | **Employment** | **~~.672~~** | **8** | **.08** |
|  | **Chemotherapy** | **~~.941~~** | **9** | **.09** |
|  | **Social participation frequency** | **~~.942~~** | **10** | **.10** |

**************************************************************************

OUTCOME VARIABLE:

SWBwn

Model Summary

R R-sq MSE F df1 df2 p

.53393 .28508 .06040 7.57640 11.00000 209.00000 .00000

Model

coeff se t p LLCI ULCI

constant .40422 .14884 2.71581 .00716 .11080 .69764

Stigma_I -.00996 .00360 -2.76204 .00626 -.01706 -.00285

MOS_SSS .35630 .06454 5.52041 .00000 .22906 .48354

Age_cov -.00126 .00188 -.67024 .50345 -.00497 .00245

Educatio -.00336 .05038 -.06669 .94690 -.10267 .09595

Employme .01647 .05332 .30892 .75769 -.08864 .12159

Social_p .00915 .01173 .77988 .43635 -.01398 .03228

Insuranc .05845 .05937 .98454 .32599 -.05859 .17550

Major_Ps -.11800 .04059 -2.90702 .00404 -.19803 -.03798

Chemo_co -.03619 .02508 -1.44337 .15041 -.08563 .01324

Mastecto .00958 .02366 .40495 .68593 -.03706 .05622

Radiothe .07478 .04303 1.73796 .08369 -.01004 .15960

Standardized coefficients

coeff

Stigma_I -.17301

MOS_SSS .34507

Age_cov -.04060

Educatio -.00466

Employme .02176

Social_p .04653

Insuranc .05933

Major_Ps -.17825

Chemo_co -.10879

Mastecto .02723

Radiothe .12950

| **Benjamini-Hochberg Procedure** | | | | |
| --- | --- | --- | --- | --- |
|  |  | **Ascending P-value s** | **I = ranking** | **(I/11)* 0.10** |
|  | **Total Social Support** | **.00000** | **1** | **.009** |
|  | **Major Psychological issues** | **.004** | **2** | **.018** |
|  | **Stigma** | **.006** | **3** | **.027** |
|  | **Radiotherapy** | **~~.080~~** | **4** | **.03636** |
|  | **Chemotherapy** | **~~.150~~** | **5** | **.045** |
|  | **Insurance** | **~~.325~~** | **6** | **.054** |
|  | **Social participation frequency** | **~~.436~~** | **7** | **.063** |
|  | **Age** | **~~.503~~** | **8** | **.072** |
|  | **Mastectomy** | **~~.685~~** | **9** | **.081** |
|  | **Employment** | **~~.757~~** | **10** | **.0909** |
|  | **Education** | **~~.946~~** | **11** | **.100** |

************************** TOTAL EFFECT MODEL ****************************

OUTCOME VARIABLE:

SWBwn

Model Summary

R R-sq MSE F df1 df2 p

.42525 .18084 .06888 4.63587 10.00000 210.00000 .00001

Model

coeff se t p LLCI ULCI

constant .56974 .15568 3.65961 .00032 .26284 .87664

Stigma_I -.01386 .00377 -3.67144 .00031 -.02130 -.00642

Age_cov -.00066 .00200 -.32767 .74349 -.00461 .00329

Educatio -.01670 .05373 -.31075 .75630 -.12263 .08923

Employme .00788 .05691 .13850 .88998 -.10432 .12008

Social_p .00883 .01253 .70475 .48175 -.01587 .03353

Insuranc .08589 .06318 1.35953 .17544 -.03865 .21044

Major_Ps -.16142 .04253 -3.79580 .00019 -.24526 -.07759

Chemo_co -.03690 .02678 -1.37795 .16969 -.08968 .01589

Mastecto .00386 .02524 .15305 .87851 -.04589 .05362

Radiothe .09496 .04578 2.07431 .03927 .00471 .18521

Standardized coefficients

coeff

Stigma_I -.24081

Age_cov -.02116

Educatio -.02318

Employme .01041

Social_p .04490

Insuranc .08718

Major_Ps -.24383

Chemo_co -.11091

Mastecto .01098

Radiothe .16446

| **Benjamini-Hochberg Procedure** | | | | |
| --- | --- | --- | --- | --- |
|  |  | **Ascending P-value s** | **I = ranking** | **(I/10)* 0.10** |
|  | **Major Psychological issues** | **.00019** | **1** | **.01** |
|  | **Stigma** | **.00031** | **2** | **.02** |
|  | **Radiotherapy** | **~~.039~~** | **3** | **.03** |
|  | **Insurance** | **~~.175~~** | **4** | **.04** |
|  | **Chemotherapy** | **~~.169~~** | **5** | **.05** |
|  | **Social participation frequency** | **~~.481~~** | **6** | **.06** |
|  | **Age** | **~~.743~~** | **7** | **.07** |
|  | **Education** | **~~.756~~** | **8** | **.08** |
|  | **Mastectomy** | **~~.878~~** | **9** | **.09** |
|  | **Employment** | **~~.889~~** | **10** | **.10** |

************** TOTAL, DIRECT, AND INDIRECT EFFECTS OF X ON Y **************

Total effect of X on Y

Effect se t p LLCI ULCI c_ps c_cs

-.01386 .00377 -3.67144 .00031 -.02130 -.00642 -.04891 -.24081

Direct effect of X on Y

Effect se t p LLCI ULCI c'_ps c'_cs

-.00996 .00360 -2.76204 .00626 -.01706 -.00285 -.03514 -.17301

Indirect effect(s) of X on Y:

Effect BootSE BootLLCI BootULCI

MOS_SSS -.00390 .00145 -.00688 -.00125

Partially standardized indirect effect(s) of X on Y:

Effect BootSE BootLLCI BootULCI

MOS_SSS -.01377 .00497 -.02385 -.00449

Completely standardized indirect effect(s) of X on Y:

Effect BootSE BootLLCI BootULCI

MOS_SSS -.06780 .02518 -.11925 -.02145

*********************** ANALYSIS NOTES AND ERRORS ************************

Level of confidence for all confidence intervals in output:

95.0000

Number of bootstrap samples for percentile bootstrap confidence intervals:

5000

***************** PROCESS Procedure for SPSS Version 3.5 *****************

Written by Andrew F. Hayes, Ph.D. www.afhayes.com

Documentation available in Hayes (2018). www.guilford.com/p/hayes3

**************************************************************************

**Model : 4**

**Y : SWB = Social Well-being**

**X : Stigma**

**M1 : Meaningfulness**

**M2 : MOS_SSS = total Social support**

Covariates:

Age_cov Educatio Employme Social_p Insuranc Major_Ps Chemo_co Mastecto Radiothe

Sample

Size: 221

**************************************************************************

OUTCOME VARIABLE:

Meaningf

Model Summary

R R-sq MSE F df1 df2 p

.43435 .18866 20.31616 4.88319 10.00000 210.00000 .00000

Model

coeff se t p LLCI ULCI

constant 22.12763 2.67375 8.27588 .00000 16.85680 27.39846

Stigma_I -.23369 .06482 -3.60503 .00039 -.36147 -.10590

Age_cov .05889 .03442 1.71069 .08861 -.00897 .12674

Educatio -1.59914 .92285 -1.73283 .08459 -3.41839 .22010

Employme .60739 .97747 .62139 .53502 -1.31953 2.53430

Social_p .53412 .21518 2.48215 .01384 .10992 .95831

Insuranc .02574 1.08506 .02372 .98110 -2.11327 2.16475

Major_Ps -1.33952 .73036 -1.83404 .06806 -2.77930 .10026

Chemo_co -.26584 .45987 -.57808 .56383 -1.17240 .64071

Mastecto -.84146 .43347 -1.94122 .05357 -1.69596 .01305

Radiothe .61470 .78626 .78180 .43521 -.93528 2.16468

Standardized coefficients

coeff

Stigma_I -.23532

Age_cov .10993

Educatio -.12862

Employme .04649

Social_p .15739

Insuranc .00151

Major_Ps -.11725

Chemo_co -.04631

Mastecto -.13858

Radiothe .06169

| **Benjamini-Hochberg Procedure** | | | | |
| --- | --- | --- | --- | --- |
|  |  | **Ascending P-value s** | **I = ranking** | **(I/10)* 0.10** |
|  | **Stigma** | **.00039** | **1** | **.01** |
|  | **Social participation frequency** | **.0138** | **2** | **.02** |
|  | **Mastectomy** | **~~.053~~** | **3** | **.03** |
|  | **Major Psychological issues** | **~~.068~~** | **4** | **.04** |
|  | **Education** | **~~.084~~** | **5** | **.05** |
|  | **Age** | **~~.088~~** | **6** | **.06** |
|  | **Radiotherapy** | **~~.435~~** | **7** | **.07** |
|  | **Employment** | **~~.535~~** | **8** | **.08** |
|  | **Chemotherapy** | **~~.563~~** | **9** | **.09** |
|  | **Insurance** | **~~.981~~** | **10** | **.10** |

**************************************************************************

OUTCOME VARIABLE:

MOS_SSS

Model Summary

R R-sq MSE F df1 df2 p

.35287 .12451 .06905 2.98667 10.00000 210.00000 .00153

Model

coeff se t p LLCI ULCI

constant .46456 .15587 2.98037 .00322 .15728 .77183

Stigma_I -.01095 .00378 -2.89783 .00416 -.01840 -.00350

Age_cov .00169 .00201 .84386 .39971 -.00226 .00565

Educatio -.03744 .05380 -.69584 .48730 -.14349 .06862

Employme -.02411 .05698 -.42304 .67270 -.13644 .08823

Social_p -.00090 .01254 -.07168 .94292 -.02563 .02383

Insuranc .07701 .06326 1.21750 .22478 -.04768 .20171

Major_Ps -.12186 .04258 -2.86202 .00464 -.20579 -.03792

Chemo_co -.00198 .02681 -.07371 .94131 -.05483 .05087

Mastecto -.01605 .02527 -.63501 .52612 -.06586 .03377

Radiothe .05665 .04584 1.23597 .21785 -.03371 .14701

Standardized coefficients

coeff

Stigma_I -.19649

Age_cov .05633

Educatio -.05365

Employme -.03288

Social_p -.00472

Insuranc .08071

Major_Ps -.19006

Chemo_co -.00613

Mastecto -.04709

Radiothe .10131

| **Benjamini-Hochberg Procedure** | | | | |
| --- | --- | --- | --- | --- |
|  |  | **Ascending P-value s** | **I = ranking** | **(I/10)* 0.10** |
|  | **Stigma** | **.0041** | **1** | **.01** |
|  | **Major Psychological issues** | **.0046** | **2** | **.02** |
|  | **Radiotherapy** | **~~.217~~** | **3** | **.03** |
|  | **Insurance** | **~~.224~~** | **4** | **.04** |
|  | **Age** | **~~.399~~** | **5** | **.05** |
|  | **Education** | **~~.487~~** | **6** | **.06** |
|  | **Mastectomy** | **~~.526~~** | **7** | **.07** |
|  | **Employment** | **~~.672~~** | **8** | **.08** |
|  | **Chemotherapy** | **~~.941~~** | **9** | **.09** |
|  | **Social participation frequency** | **~~.942~~** | **10** | **.10** |

**************************************************************************

OUTCOME VARIABLE:

SWBwn

Model Summary

R R-sq MSE F df1 df2 p

.55349 .30635 .05889 7.65537 12.00000 208.00000 .00000

Model

coeff se t p LLCI ULCI

constant .20920 .16601 1.26020 .20901 -.11807 .53648

Stigma_I -.00815 .00363 -2.24595 .02576 -.01531 -.00100

Meaningf .00970 .00384 2.52569 .01229 .00213 .01728

MOS_SSS .31389 .06590 4.76305 .00000 .18397 .44382

Age_cov -.00176 .00187 -.94260 .34698 -.00544 .00192

Educatio .01057 .05005 .21121 .83293 -.08809 .10923

Employme .00956 .05272 .18126 .85634 -.09437 .11349

Social_p .00393 .01177 .33392 .73878 -.01927 .02713

Insuranc .06147 .05863 1.04836 .29569 -.05412 .17706

Major_Ps -.11017 .04020 -2.74063 .00667 -.18942 -.03092

Chemo_co -.03370 .02478 -1.35994 .17532 -.08255 .01515

Mastecto .01706 .02355 .72473 .46943 -.02936 .06348

Radiothe .07122 .04251 1.67541 .09536 -.01258 .15502

Standardized coefficients

coeff

Stigma_I -.14167

Meaningf .16745

MOS_SSS .30400

Age_cov -.05669

Educatio .01467

Employme .01262

Social_p .01998

Insuranc .06239

Major_Ps -.16642

Chemo_co -.10129

Mastecto .04850

Radiothe .12334

| **Benjamini-Hochberg Procedure** | | | | |
| --- | --- | --- | --- | --- |
|  |  | **Ascending P-value s** | **I = ranking** | **(I/12)* 0.10** |
|  | **Total Social Support** | **.00000** | **1** | **.0083** |
|  | **Major Psychological issues** | **.006** | **2** | **.016** |
|  | **Meaningfulness** | **.0122** | **3** | **.025** |
|  | **Stigma** | **.0257** | **4** | **.033** |
|  | **Radiotherapy** | **~~.095~~** | **5** | **.0416** |
|  | **Chemotherapy** | **~~.175~~** | **6** | **.050** |
|  | **Insurance** | **~~.295~~** | **7** | **.0583** |
|  | **Age** | **~~.346~~** | **8** | **.066** |
|  | **Mastectomy** | **~~.469~~** | **9** | **.075** |
|  | **Social participation frequency** | **~~.738~~** | **10** | **.083** |
|  | **Education** | **~~.832~~** | **11** | **.091** |
|  | **Employment** | **~~.856~~** | **12** | **.10** |

************************** TOTAL EFFECT MODEL ****************************

OUTCOME VARIABLE:

SWBwn

Model Summary

R R-sq MSE F df1 df2 p

.42525 .18084 .06888 4.63587 10.00000 210.00000 .00001

Model

coeff se t p LLCI ULCI

constant .56974 .15568 3.65961 .00032 .26284 .87664

Stigma_I -.01386 .00377 -3.67144 .00031 -.02130 -.00642

Age_cov -.00066 .00200 -.32767 .74349 -.00461 .00329

Educatio -.01670 .05373 -.31075 .75630 -.12263 .08923

Employme .00788 .05691 .13850 .88998 -.10432 .12008

Social_p .00883 .01253 .70475 .48175 -.01587 .03353

Insuranc .08589 .06318 1.35953 .17544 -.03865 .21044

Major_Ps -.16142 .04253 -3.79580 .00019 -.24526 -.07759

Chemo_co -.03690 .02678 -1.37795 .16969 -.08968 .01589

Mastecto .00386 .02524 .15305 .87851 -.04589 .05362

Radiothe .09496 .04578 2.07431 .03927 .00471 .18521

Standardized coefficients

coeff

Stigma_I -.24081

Age_cov -.02116

Educatio -.02318

Employme .01041

Social_p .04490

Insuranc .08718

Major_Ps -.24383

Chemo_co -.11091

Mastecto .01098

Radiothe .16446

| **Benjamini-Hochberg Procedure** | | | | |
| --- | --- | --- | --- | --- |
|  |  | **Ascending P-value s** | **I = ranking** | **(I/10)* 0.10** |
|  | **Major Psychological issues** | **.00019** | **1** | **.01** |
|  | **Stigma** | **.00031** | **2** | **.02** |
|  | **Radiotherapy** | **~~.039~~** | **3** | **.03** |
|  | **Insurance** | **~~.175~~** | **4** | **.04** |
|  | **Chemotherapy** | **~~.169~~** | **5** | **.05** |
|  | **Social participation frequency** | **~~.481~~** | **6** | **.06** |
|  | **Age** | **~~.743~~** | **7** | **.07** |
|  | **Education** | **~~.756~~** | **8** | **.08** |
|  | **Mastectomy** | **~~.878~~** | **9** | **.09** |
|  | **Employment** | **~~.889~~** | **10** | **.10** |

************** TOTAL, DIRECT, AND INDIRECT EFFECTS OF X ON Y **************

Total effect of X on Y

Effect se t p LLCI ULCI c_ps c_cs

-.01386 .00377 -3.67144 .00031 -.02130 -.00642 -.04891 -.24081

Direct effect of X on Y

Effect se t p LLCI ULCI c'_ps c'_cs

-.00815 .00363 -2.24595 .02576 -.01531 -.00100 -.02878 -.14167

Indirect effect(s) of X on Y:

Effect BootSE BootLLCI BootULCI

TOTAL -.00570 .00170 -.00929 -.00263

Meaningf -.00227 .00108 -.00463 -.00041

MOS_SSS -.00344 .00133 -.00626 -.00111

Partially standardized indirect effect(s) of X on Y:

Effect BootSE BootLLCI BootULCI

TOTAL -.02014 .00578 -.03215 -.00944

Meaningf -.00800 .00380 -.01638 -.00150

MOS_SSS -.01213 .00458 -.02177 -.00410

Completely standardized indirect effect(s) of X on Y:

Effect BootSE BootLLCI BootULCI

TOTAL -.09914 .02927 -.16073 -.04580

Meaningf -.03940 .01869 -.08035 -.00721

MOS_SSS -.05973 .02315 -.10929 -.01952

*********************** ANALYSIS NOTES AND ERRORS ************************

Level of confidence for all confidence intervals in output:

95.0000

Number of bootstrap samples for percentile bootstrap confidence intervals:

5000

***************** PROCESS Procedure for SPSS Version 3.5 *****************

Written by Andrew F. Hayes, Ph.D. www.afhayes.com

Documentation available in Hayes (2018). www.guilford.com/p/hayes3

**************************************************************************

**Model : 4**

**Y : EWB = Emotional Well-being**

**X : Stigma_IV**

**M : Comprehensibility**

Covariates:

Age_cov Educatio Employme Social_p Insuranc Major_Ps Chemo_co Mastecto Radiothe

Sample

Size: 221

**************************************************************************

OUTCOME VARIABLE:

Comprehe

Model Summary

R R-sq MSE F(HC4) df1 df2 p

.38011 .14448 36.53598 3.74051 10.00000 210.00000 .00012

Model

coeff se(HC4) t p LLCI ULCI

constant 28.41039 3.64463 7.79514 .00000 21.22564 35.59514

Stigma_I -.32272 .08970 -3.59785 .00040 -.49954 -.14589

Age_cov .04775 .04451 1.07269 .28464 -.04000 .13550

Educatio -.31523 1.13449 -.27786 .78139 -2.55167 1.92121

Employme 1.82890 1.25331 1.45925 .14599 -.64179 4.29959

Social_p .36516 .34624 1.05464 .29280 -.31739 1.04770

Insuranc -.55790 1.47487 -.37827 .70561 -3.46534 2.34955

Major_Ps -1.89152 1.06151 -1.78193 .07621 -3.98410 .20105

Chemo_co -1.16488 .60020 -1.94081 .05362 -2.34807 .01831

Mastecto -1.47027 .58651 -2.50683 .01294 -2.62647 -.31408

Radiothe -1.08926 1.07527 -1.01301 .31222 -3.20896 1.03045

Standardized coefficients

coeff

Stigma_I -.24884

Age_cov .06826

Educatio -.01941

Employme .10719

Social_p .08239

Insuranc -.02513

Major_Ps -.12678

Chemo_co -.15537

Mastecto -.18542

Radiothe -.08370

| **Benjamini-Hochberg Procedure** | | | | |
| --- | --- | --- | --- | --- |
|  |  | **Ascending P-value s** | **I = ranking** | **(I/10)* 0.10** |
|  | **Stigma** | **.0004** | **1** | **.01** |
|  | **Mastectomy** | **.0129** | **2** | **.02** |
|  | **Chemotherapy** | **~~.053~~** | **3** | **.03** |
|  | **Major Psychological issues** | **~~.076~~** | **4** | **.04** |
|  | **Employment** | **~~.145~~** | **5** | **.05** |
|  | **Age** | **~~.284~~** | **6** | **.06** |
|  | **Social participation frequency** | **~~.292~~** | **7** | **.07** |
|  | **Radiotherapy** | **~~.312~~** | **8** | **.08** |
|  | **Insurance** | **~~.705~~** | **9** | **.09** |
|  | **Education** | **~~.781~~** | **10** | **.10** |

**************************************************************************

OUTCOME VARIABLE:

EWB

Model Summary

R R-sq MSE F(HC4) df1 df2 p

.48328 .23356 21.78663 5.09898 11.00000 209.00000 .00000

Model

coeff se(HC4) t p LLCI ULCI

constant 16.44554 3.11092 5.28639 .00000 10.31273 22.57834

Stigma_I -.33882 .07643 -4.43288 .00002 -.48950 -.18814

Comprehe .16256 .05591 2.90755 .00404 .05234 .27277

Age_cov .01557 .03611 .43124 .66674 -.05562 .08677

Educatio .39795 .80974 .49145 .62362 -1.19836 1.99426

Employme -.19000 .82613 -.22998 .81833 -1.81862 1.43863

Social_p .37893 .17953 2.11072 .03598 .02502 .73285

Insuranc .57062 .99844 .57151 .56827 -1.39769 2.53893

Major_Ps -1.58738 .78398 -2.02477 .04416 -3.13290 -.04186

Chemo_co -.24270 .48345 -.50202 .61618 -1.19577 .71036

Mastecto -.55634 .44830 -1.24100 .21600 -1.44012 .32743

Radiothe -.81976 .87444 -.93747 .34960 -2.54362 .90410

Standardized coefficients

coeff

Stigma_I -.32100

Comprehe .19972

Age_cov .02735

Educatio .03011

Employme -.01368

Social_p .10505

Insuranc .03157

Major_Ps -.13072

Chemo_co -.03977

Mastecto -.08620

Radiothe -.07740

| **Benjamini-Hochberg Procedure** | | | | |
| --- | --- | --- | --- | --- |
|  |  | **Ascending P-value s** | **I = ranking** | **(I/11)* 0.10** |
|  | **Stigma** | **.00002** | **1** | **.009** |
|  | **Comprehensibility** | **.004** | **2** | **.018** |
|  | **Social participation frequency** | **~~.0359~~** | **3** | **.027** |
|  | **Major Psychological issues** | **~~.044~~** | **4** | **.03636** |
|  | **Mastectomy** | **~~.216~~** | **5** | **.045** |
|  | **Radiotherapy** | **~~.349~~** | **6** | **.054** |
|  | **Insurance** | **~~.568~~** | **7** | **.063** |
|  | **Chemotherapy** | **~~.616~~** | **8** | **.072** |
|  | **Education** | **~~.623~~** | **9** | **.081** |
|  | **Age** | **~~.666~~** | **10** | **.0909** |
|  | **Employment** | **~~.818~~** | **11** | **.100** |

************************** TOTAL EFFECT MODEL ****************************

OUTCOME VARIABLE:

EWB

Model Summary

R R-sq MSE F(HC4) df1 df2 p

.44658 .19944 22.64833 5.06267 10.00000 210.00000 .00000

Model

coeff se(HC4) t p LLCI ULCI

constant 21.06384 2.76475 7.61872 .00000 15.61362 26.51405

Stigma_I -.39128 .07569 -5.16978 .00000 -.54048 -.24208

Age_cov .02334 .03691 .63218 .52796 -.04943 .09610

Educatio .34671 .85743 .40436 .68636 -1.34356 2.03698

Employme .10730 .88896 .12071 .90404 -1.64513 1.85974

Social_p .43829 .19024 2.30384 .02221 .06326 .81333

Insuranc .47993 1.03206 .46502 .64240 -1.55459 2.51446

Major_Ps -1.89486 .82516 -2.29635 .02264 -3.52151 -.26820

Chemo_co -.43206 .48456 -.89166 .37360 -1.38729 .52316

Mastecto -.79535 .47907 -1.66017 .09837 -1.73976 .14906

Radiothe -.99683 .93056 -1.07121 .28531 -2.83127 .83762

Standardized coefficients

coeff

Stigma_I -.37070

Age_cov .04099

Educatio .02624

Employme .00773

Social_p .12151

Insuranc .02656

Major_Ps -.15604

Chemo_co -.07080

Mastecto -.12324

Radiothe -.09412

| **Benjamini-Hochberg Procedure** | | | | |
| --- | --- | --- | --- | --- |
|  |  | **Ascending P-value s** | **I = ranking** | **(I/10)* 0.10** |
|  | **Stigma** | **.00000** | **1** | **.01** |
|  | **Social participation frequency** | **~~.0222~~** | **2** | **.02** |
|  | **Major Psychological issues** | **~~.0226~~** | **3** | **.03** |
|  | **Mastectomy** | **~~.098~~** | **4** | **.04** |
|  | **Radiotherapy** | **~~.285~~** | **5** | **.05** |
|  | **Chemotherapy** | **~~.373~~** | **6** | **.06** |
|  | **Age** | **~~.527~~** | **7** | **.07** |
|  | **Insurance** | **~~.642~~** | **8** | **.08** |
|  | **Education** | **~~.686~~** | **9** | **.09** |
|  | **Employment** | **~~.904~~** | **10** | **.10** |

************** TOTAL, DIRECT, AND INDIRECT EFFECTS OF X ON Y **************

Total effect of X on Y

Effect se(HC4) t p LLCI ULCI c_ps c_cs

-.39128 .07569 -5.16978 .00000 -.54048 -.24208 -.07530 -.37070

Direct effect of X on Y

Effect se(HC4) t p LLCI ULCI c'_ps c'_cs

-.33882 .07643 -4.43288 .00002 -.48950 -.18814 -.06520 -.32100

Indirect effect(s) of X on Y:

Effect BootSE BootLLCI BootULCI

Comprehe -.05246 .02175 -.09948 -.01485

Partially standardized indirect effect(s) of X on Y:

Effect BootSE BootLLCI BootULCI

Comprehe -.01010 .00411 -.01901 -.00293

Completely standardized indirect effect(s) of X on Y:

Effect BootSE BootLLCI BootULCI

Comprehe -.04970 .01987 -.09186 -.01449

*********************** ANALYSIS NOTES AND ERRORS ************************

Level of confidence for all confidence intervals in output:

95.0000

Number of bootstrap samples for percentile bootstrap confidence intervals:

5000

NOTE: A heteroscedasticity consistent standard error and covariance matrix estimator was used.

***************** PROCESS Procedure for SPSS Version 3.5 *****************

Written by Andrew F. Hayes, Ph.D. www.afhayes.com

Documentation available in Hayes (2018). www.guilford.com/p/hayes3

**************************************************************************

**Model : 4**

**Y : EWB = Emotional Well-being**

**X : Stigma_IV**

**M : Manageability**

Covariates:

Age_cov Educatio Employme Social_p Insuranc Major_Ps Chemo_co Mastecto Radiothe

Sample

Size: 221

**************************************************************************

OUTCOME VARIABLE:

Manageab

Model Summary

R R-sq MSE F(HC4) df1 df2 p

.35462 .12576 30.37485 2.97038 10.00000 210.00000 .00161

Model

coeff se(HC4) t p LLCI ULCI

constant 17.47806 3.59395 4.86319 .00000 10.39322 24.56291

Stigma_I -.26580 .07642 -3.47821 .00061 -.41644 -.11515

Age_cov .06260 .04121 1.51893 .13028 -.01864 .14384

Educatio 1.48418 1.11507 1.33102 .18463 -.71399 3.68235

Employme .90084 1.15318 .78118 .43557 -1.37244 3.17413

Social_p .51544 .37142 1.38776 .16668 -.21675 1.24763

Insuranc -1.09144 1.42344 -.76676 .44409 -3.89750 1.71463

Major_Ps -1.73020 .92147 -1.87766 .06181 -3.54671 .08631

Chemo_co -.05627 .54364 -.10351 .91766 -1.12797 1.01542

Mastecto -.68241 .51570 -1.32327 .18718 -1.69902 .33420

Radiothe .09180 .96482 .09515 .92429 -1.81018 1.99377

Standardized coefficients

coeff

Stigma_I -.22723

Age_cov .09921

Educatio .10134

Employme .05853

Social_p .12894

Insuranc -.05450

Major_Ps -.12857

Chemo_co -.00832

Mastecto -.09541

Radiothe .00782

| **Benjamini-Hochberg Procedure** | | | | |
| --- | --- | --- | --- | --- |
|  |  | **Ascending P-value s** | **I = ranking** | **(I/10)* 0.10** |
|  | **Stigma** | **.0006** | **1** | **.01** |
|  | **Major Psychological issues** | **~~.061~~** | **2** | **.02** |
|  | **Age** | **~~.130~~** | **3** | **.03** |
|  | **Social participation frequency** | **~~.166~~** | **4** | **.04** |
|  | **Education** | **~~.184~~** | **5** | **.05** |
|  | **Mastectomy** | **~~.187~~** | **6** | **.06** |
|  | **Employment** | **~~.435~~** | **7** | **.07** |
|  | **Insurance** | **~~.444~~** | **8** | **.08** |
|  | **Chemotherapy** | **~~.917~~** | **9** | **.09** |
|  | **Radiotherapy** | **~~.924~~** | **10** | **.10** |

**************************************************************************

OUTCOME VARIABLE:

EWB

Model Summary

R R-sq MSE F(HC4) df1 df2 p

.46280 .21419 22.33737 4.55746 11.00000 209.00000 .00000

Model

coeff se(HC4) t p LLCI ULCI

constant 19.01515 2.87140 6.62225 .00000 13.35452 24.67578

Stigma_I -.36013 .07726 -4.66102 .00001 -.51244 -.20781

Manageab .11721 .05689 2.06028 .04061 .00506 .22937

Age_cov .01600 .03712 .43103 .66689 -.05717 .08917

Educatio .17274 .86957 .19865 .84273 -1.54152 1.88700

Employme .00171 .86015 .00199 .99841 -1.69397 1.69739

Social_p .37788 .20347 1.85717 .06469 -.02324 .77899

Insuranc .60786 .98591 .61655 .53820 -1.33575 2.55148

Major_Ps -1.69205 .79409 -2.13079 .03427 -3.25751 -.12659

Chemo_co -.42546 .47161 -.90215 .36802 -1.35519 .50426

Mastecto -.71536 .46374 -1.54259 .12444 -1.62956 .19885

Radiothe -1.00759 .90571 -1.11248 .26721 -2.79309 .77792

Standardized coefficients

coeff

Stigma_I -.34118

Manageab .12990

Age_cov .02810

Educatio .01307

Employme .00012

Social_p .10476

Insuranc .03364

Major_Ps -.13934

Chemo_co -.06972

Mastecto -.11084

Radiothe -.09513

| **Benjamini-Hochberg Procedure** | | | | |
| --- | --- | --- | --- | --- |
|  |  | **Ascending P-value s** | **I = ranking** | **(I/11)* 0.10** |
|  | **Stigma** | **.00001** | **1** | **.009** |
|  | **Major Psychological issues** | **~~.0342~~** | **2** | **.018** |
|  | **Manageability** | **~~.0406~~** | **3** | **.027** |
|  | **Social participation frequency** | **~~.0646~~** | **4** | **.03636** |
|  | **Mastectomy** | **~~.124~~** | **5** | **.045** |
|  | **Radiotherapy** | **~~.267~~** | **6** | **.054** |
|  | **Chemotherapy** | **~~.368~~** | **7** | **.063** |
|  | **Insurance** | **~~.538~~** | **8** | **.072** |
|  | **Age** | **~~.667~~** | **9** | **.081** |
|  | **Education** | **~~.823~~** | **10** | **.0909** |
|  | **Employment** | **~~.998~~** | **11** | **.100** |

************************** TOTAL EFFECT MODEL ****************************

OUTCOME VARIABLE:

EWB

Model Summary

R R-sq MSE F(HC4) df1 df2 p

.44658 .19944 22.64833 5.06267 10.00000 210.00000 .00000

Model

coeff se(HC4) t p LLCI ULCI

constant 21.06384 2.76475 7.61872 .00000 15.61362 26.51405

Stigma_I -.39128 .07569 -5.16978 .00000 -.54048 -.24208

Age_cov .02334 .03691 .63218 .52796 -.04943 .09610

Educatio .34671 .85743 .40436 .68636 -1.34356 2.03698

Employme .10730 .88896 .12071 .90404 -1.64513 1.85974

Social_p .43829 .19024 2.30384 .02221 .06326 .81333

Insuranc .47993 1.03206 .46502 .64240 -1.55459 2.51446

Major_Ps -1.89486 .82516 -2.29635 .02264 -3.52151 -.26820

Chemo_co -.43206 .48456 -.89166 .37360 -1.38729 .52316

Mastecto -.79535 .47907 -1.66017 .09837 -1.73976 .14906

Radiothe -.99683 .93056 -1.07121 .28531 -2.83127 .83762

Standardized coefficients

coeff

Stigma_I -.37070

Age_cov .04099

Educatio .02624

Employme .00773

Social_p .12151

Insuranc .02656

Major_Ps -.15604

Chemo_co -.07080

Mastecto -.12324

Radiothe -.09412

| **Benjamini-Hochberg Procedure** | | | | |
| --- | --- | --- | --- | --- |
|  |  | **Ascending P-value s** | **I = ranking** | **(I/10)* 0.10** |
|  | **Stigma** | **.00000** | **1** | **.01** |
|  | **Social participation frequency** | **~~.0222~~** | **2** | **.02** |
|  | **Major Psychological issues** | **~~.0226~~** | **3** | **.03** |
|  | **Mastectomy** | **~~.098~~** | **4** | **.04** |
|  | **Radiotherapy** | **~~.287~~** | **5** | **.05** |
|  | **Chemotherapy** | **~~.373~~** | **6** | **.06** |
|  | **Age** | **~~.527~~** | **7** | **.07** |
|  | **Insurance** | **~~.642~~** | **8** | **.08** |
|  | **Education** | **~~.686~~** | **9** | **.09** |
|  | **Employment** | **~~.904~~** | **10** | **.10** |

************** TOTAL, DIRECT, AND INDIRECT EFFECTS OF X ON Y **************

Total effect of X on Y

Effect se(HC4) t p LLCI ULCI c_ps c_cs

-.39128 .07569 -5.16978 .00000 -.54048 -.24208 -.07530 -.37070

Direct effect of X on Y

Effect se(HC4) t p LLCI ULCI c'_ps c'_cs

-.36013 .07726 -4.66102 .00001 -.51244 -.20781 -.06930 -.34118

Indirect effect(s) of X on Y:

Effect BootSE BootLLCI BootULCI

Manageab -.03116 .01690 -.06671 -.00058

Partially standardized indirect effect(s) of X on Y:

Effect BootSE BootLLCI BootULCI

Manageab -.00600 .00323 -.01273 -.00011

Completely standardized indirect effect(s) of X on Y:

Effect BootSE BootLLCI BootULCI

Manageab -.02952 .01589 -.06286 -.00055

*********************** ANALYSIS NOTES AND ERRORS ************************

Level of confidence for all confidence intervals in output:

95.0000

Number of bootstrap samples for percentile bootstrap confidence intervals:

5000

NOTE: A heteroscedasticity consistent standard error and covariance matrix estimator was used.

***************** PROCESS Procedure for SPSS Version 3.5 *****************

Written by Andrew F. Hayes, Ph.D. www.afhayes.com

Documentation available in Hayes (2018). www.guilford.com/p/hayes3

**************************************************************************

**Model : 4**

**Y : EWB = Emotional Well-being**

**X : Stigma_IV**

**M : Meaningfulness**

Covariates:

Age_cov Educatio Employme Social_p Insuranc Major_Ps Chemo_co Mastecto Radiothe

Sample

Size: 221

**************************************************************************

OUTCOME VARIABLE:

Meaningf

Model Summary

R R-sq MSE F(HC4) df1 df2 p

.43435 .18866 20.31616 8.05231 10.00000 210.00000 .00000

Model

coeff se(HC4) t p LLCI ULCI

constant 22.12763 2.53165 8.74041 .00000 17.13693 27.11833

Stigma_I -.23369 .06320 -3.69777 .00028 -.35827 -.10910

Age_cov .05889 .03292 1.78882 .07509 -.00601 .12378

Educatio -1.59914 1.01029 -1.58285 .11496 -3.59076 .39248

Employme .60739 1.21085 .50162 .61646 -1.77959 2.99436

Social_p .53412 .26313 2.02983 .04364 .01539 1.05284

Insuranc .02574 1.05951 .02429 .98064 -2.06290 2.11438

Major_Ps -1.33952 .72667 -1.84337 .06668 -2.77201 .09298

Chemo_co -.26584 .43980 -.60446 .54619 -1.13283 .60115

Mastecto -.84146 .43800 -1.92113 .05607 -1.70490 .02198

Radiothe .61470 .76913 .79922 .42506 -.90150 2.13090

Standardized coefficients

coeff

Stigma_I -.23532

Age_cov .10993

Educatio -.12862

Employme .04649

Social_p .15739

Insuranc .00151

Major_Ps -.11725

Chemo_co -.04631

Mastecto -.13858

Radiothe .06169

| **Benjamini-Hochberg Procedure** | | | | |
| --- | --- | --- | --- | --- |
|  |  | **Ascending P-value s** | **I = ranking** | **(I/10)* 0.10** |
|  | **Stigma** | **.0002** | **1** | **.01** |
|  | **Social participation frequency** | **~~.043~~** | **2** | **.02** |
|  | **Mastectomy** | **~~.056~~** | **3** | **.03** |
|  | **Major Psychological issues** | **~~.066~~** | **4** | **.04** |
|  | **Age** | **~~.075~~** | **5** | **.05** |
|  | **Education** | **~~.114~~** | **6** | **.06** |
|  | **Radiotherapy** | **~~.425~~** | **7** | **.07** |
|  | **Chemotherapy** | **~~.546~~** | **8** | **.08** |
|  | **Employment** | **~~.616~~** | **9** | **.09** |
|  | **Insurance** | **~~.980~~** | **10** | **.10** |

**************************************************************************

OUTCOME VARIABLE:

EWB

Model Summary

R R-sq MSE F(HC4) df1 df2 p

.50941 .25950 21.04925 6.22213 11.00000 209.00000 .00000

Model

coeff se(HC4) t p LLCI ULCI

constant 14.66425 3.12654 4.69024 .00000 8.50065 20.82786

Stigma_I -.32370 .07473 -4.33127 .00002 -.47103 -.17637

Meaningf .28921 .07305 3.95930 .00010 .14521 .43321

Age_cov .00631 .03755 .16792 .86681 -.06772 .08033

Educatio .80920 .77252 1.04749 .29609 -.71372 2.33212

Employme -.06836 .78931 -.08661 .93107 -1.62439 1.48767

Social_p .28382 .21755 1.30462 .19346 -.14505 .71269

Insuranc .47249 .92222 .51234 .60896 -1.34555 2.29053

Major_Ps -1.50745 .77935 -1.93424 .05443 -3.04385 .02895

Chemo_co -.35518 .47553 -.74690 .45596 -1.29263 .58228

Mastecto -.55199 .46771 -1.18019 .23927 -1.47402 .37005

Radiothe -1.17461 .87530 -1.34195 .18107 -2.90016 .55094

Standardized coefficients

coeff

Stigma_I -.30667

Meaningf .27209

Age_cov .01107

Educatio .06123

Employme -.00492

Social_p .07868

Insuranc .02614

Major_Ps -.12414

Chemo_co -.05820

Mastecto -.08553

Radiothe -.11090

| **Benjamini-Hochberg Procedure** | | | | |
| --- | --- | --- | --- | --- |
|  |  | **Ascending P-value s** | **I = ranking** | **(I/11)* 0.10** |
|  | **Stigma** | **.00002** | **1** | **.009** |
|  | **Meaningfulness** | **.0001** | **2** | **.018** |
|  | **Major Psychological issues** | **~~.054~~** | **3** | **.027** |
|  | **Social participation frequency** | **~~.193~~** | **4** | **.03636** |
|  | **Radiotherapy** | **~~.181~~** | **5** | **.045** |
|  | **Mastectomy** | **~~.239~~** | **6** | **.054** |
|  | **Education** | **~~.269~~** | **7** | **.063** |
|  | **Chemotherapy** | **~~.455~~** | **8** | **.072** |
|  | **Insurance** | **~~.608~~** | **9** | **.081** |
|  | **Age** | **~~.866~~** | **10** | **.0909** |
|  | **Employment** | **~~.931~~** | **11** | **.100** |

************************** TOTAL EFFECT MODEL ****************************

OUTCOME VARIABLE:

EWB

Model Summary

R R-sq MSE F(HC4) df1 df2 p

.44658 .19944 22.64833 5.06267 10.00000 210.00000 .00000

Model

coeff se(HC4) t p LLCI ULCI

constant 21.06384 2.76475 7.61872 .00000 15.61362 26.51405

Stigma_I -.39128 .07569 -5.16978 .00000 -.54048 -.24208

Age_cov .02334 .03691 .63218 .52796 -.04943 .09610

Educatio .34671 .85743 .40436 .68636 -1.34356 2.03698

Employme .10730 .88896 .12071 .90404 -1.64513 1.85974

Social_p .43829 .19024 2.30384 .02221 .06326 .81333

Insuranc .47993 1.03206 .46502 .64240 -1.55459 2.51446

Major_Ps -1.89486 .82516 -2.29635 .02264 -3.52151 -.26820

Chemo_co -.43206 .48456 -.89166 .37360 -1.38729 .52316

Mastecto -.79535 .47907 -1.66017 .09837 -1.73976 .14906

Radiothe -.99683 .93056 -1.07121 .28531 -2.83127 .83762

Standardized coefficients

coeff

Stigma_I -.37070

Age_cov .04099

Educatio .02624

Employme .00773

Social_p .12151

Insuranc .02656

Major_Ps -.15604

Chemo_co -.07080

Mastecto -.12324

Radiothe -.09412

| **Benjamini-Hochberg Procedure** | | | | |
| --- | --- | --- | --- | --- |
|  |  | **Ascending P-value s** | **I = ranking** | **(I/10)* 0.10** |
|  | **Stigma** | **.00000** | **1** | **.01** |
|  | **Social participation frequency** | **~~.0222~~** | **2** | **.02** |
|  | **Major Psychological issues** | **~~.0226~~** | **3** | **.03** |
|  | **Mastectomy** | **~~.098~~** | **4** | **.04** |
|  | **Radiotherapy** | **~~.285~~** | **5** | **.05** |
|  | **Chemotherapy** | **~~.373~~** | **6** | **.06** |
|  | **Age** | **~~.527~~** | **7** | **.07** |
|  | **Insurance** | **~~.642~~** | **8** | **.08** |
|  | **Education** | **~~.686~~** | **9** | **.09** |
|  | **Employment** | **~~.904~~** | **10** | **.10** |

************** TOTAL, DIRECT, AND INDIRECT EFFECTS OF X ON Y **************

Total effect of X on Y

Effect se(HC4) t p LLCI ULCI c_ps c_cs

-.39128 .07569 -5.16978 .00000 -.54048 -.24208 -.07530 -.37070

Direct effect of X on Y

Effect se(HC4) t p LLCI ULCI c'_ps c'_cs

-.32370 .07473 -4.33127 .00002 -.47103 -.17637 -.06229 -.30667

Indirect effect(s) of X on Y:

Effect BootSE BootLLCI BootULCI

Meaningf -.06758 .02493 -.12154 -.02503

Partially standardized indirect effect(s) of X on Y:

Effect BootSE BootLLCI BootULCI

Meaningf -.01301 .00471 -.02312 -.00491

Completely standardized indirect effect(s) of X on Y:

Effect BootSE BootLLCI BootULCI

Meaningf -.06403 .02356 -.11510 -.02358

*********************** ANALYSIS NOTES AND ERRORS ************************

Level of confidence for all confidence intervals in output:

95.0000

Number of bootstrap samples for percentile bootstrap confidence intervals:

5000

NOTE: A heteroscedasticity consistent standard error and covariance matrix estimator was used.

***************** PROCESS Procedure for SPSS Version 3.5 *****************

Written by Andrew F. Hayes, Ph.D. www.afhayes.com

Documentation available in Hayes (2018). www.guilford.com/p/hayes3

**************************************************************************

**Model : 4**

**Y : EWB = Emotional Well-being**

**X : Stigma_IV**

**M : SOC = Sense of coherence**

Covariates:

Age_cov Educatio Employme Social_p Insuranc Major_Ps Chemo_co Mastecto Radiothe

Sample

Size: 221

**************************************************************************

OUTCOME VARIABLE:

SOC

Model Summary

R R-sq MSE F(HC4) df1 df2 p

.45548 .20746 151.84085 6.66508 10.00000 210.00000 .00000

Model

coeff se(HC4) t p LLCI ULCI

constant 68.01609 7.67463 8.86246 .00000 52.88690 83.14528

Stigma_I -.82220 .17345 -4.74035 .00000 -1.16412 -.48028

Age_cov .16923 .09181 1.84326 .06670 -.01176 .35022

Educatio -.43019 2.51540 -.17102 .86437 -5.38886 4.52848

Employme 3.33713 2.92422 1.14120 .25508 -2.42746 9.10172

Social_p 1.41471 .66047 2.14199 .03335 .11272 2.71671

Insuranc -1.62359 3.14157 -.51681 .60583 -7.81665 4.56947

Major_Ps -4.96124 2.17111 -2.28512 .02330 -9.24121 -.68127

Chemo_co -1.48699 1.27542 -1.16588 .24498 -4.00126 1.02728

Mastecto -2.99414 1.22241 -2.44938 .01513 -5.40390 -.58437

Radiothe -.38276 2.33173 -.16415 .86977 -4.97936 4.21385

Standardized coefficients

coeff

Stigma_I -.29932

Age_cov .11422

Educatio -.01251

Employme .09234

Social_p .15071

Insuranc -.03452

Major_Ps -.15700

Chemo_co -.09364

Mastecto -.17827

Radiothe -.01389

| **Benjamini-Hochberg Procedure** | | | | |
| --- | --- | --- | --- | --- |
|  |  | **Ascending P-value s** | **I = ranking** | **(I/10)* 0.10** |
|  | **Stigma** | **.00000** | **1** | **.01** |
|  | **Mastectomy** | **.015** | **2** | **.02** |
|  | **Major Psychological issues** | **.023** | **3** | **.03** |
|  | **Social participation frequency** | **.033** | **4** | **.04** |
|  | **Age** | **~~.066~~** | **5** | **.05** |
|  | **Employment** | **~~.255~~** | **6** | **.06** |
|  | **Chemotherapy** | **~~.244~~** | **7** | **.07** |
|  | **Insurance** | **~~.605~~** | **8** | **.08** |
|  | **Education** | **~~.864~~** | **9** | **.09** |
|  | **Radiotherapy** | **~~.869~~** | **10** | **.10** |

**************************************************************************

OUTCOME VARIABLE:

EWB

Model Summary

R R-sq MSE F(HC4) df1 df2 p

.50445 .25447 21.19237 5.34992 11.00000 209.00000 .00000

Model

coeff se(HC4) t p LLCI ULCI

constant 14.17661 3.15034 4.50002 .00001 7.96609 20.38713

Stigma_I -.30803 .07574 -4.06673 .00007 -.45735 -.15871

SOC .10126 .02586 3.91553 .00012 .05028 .15224

Age_cov .00620 .03687 .16813 .86664 -.06649 .07889

Educatio .39027 .79551 .49059 .62423 -1.17797 1.95851

Employme -.23061 .78386 -.29420 .76890 -1.77590 1.31468

Social_p .29504 .20195 1.46093 .14554 -.10309 .69317

Insuranc .64433 .93155 .69168 .48991 -1.19210 2.48077

Major_Ps -1.39249 .76140 -1.82886 .06885 -2.89349 .10852

Chemo_co -.28149 .46634 -.60362 .54675 -1.20082 .63784

Mastecto -.49216 .44715 -1.10066 .27231 -1.37367 .38935

Radiothe -.95807 .85799 -1.11664 .26543 -2.64949 .73335

Standardized coefficients

coeff

Stigma_I -.29182

SOC .26351

Age_cov .01089

Educatio .02953

Employme -.01661

Social_p .08179

Insuranc .03565

Major_Ps -.11467

Chemo_co -.04613

Mastecto -.07626

Radiothe -.09046

| **Benjamini-Hochberg Procedure** | | | | |
| --- | --- | --- | --- | --- |
|  |  | **Ascending P-value s** | **I = ranking** | **(I/11)* 0.10** |
|  | **Stigma** | **0.00007** | **1** | **.009** |
|  | **Sense of coherence** | **0.00012** | **2** | **.018** |
|  | **Major Psychological issues** | **~~.0688~~** | **3** | **.027** |
|  | **Social participation frequency** | **~~.145~~** | **4** | **.03636** |
|  | **Radiotherapy** | **~~.265~~** | **5** | **.045** |
|  | **Mastectomy** | **~~.272~~** | **6** | **.054** |
|  | **Insurance** | **~~.498~~** | **7** | **.063** |
|  | **Chemotherapy** | **~~.546~~** | **8** | **.072** |
|  | **Education** | **~~.624~~** | **9** | **.081** |
|  | **Employment** | **~~.768~~** | **10** | **.0909** |
|  | **Age** | **~~.866~~** | **11** | **.100** |

************************** TOTAL EFFECT MODEL ****************************

OUTCOME VARIABLE:

EWB

Model Summary

R R-sq MSE F(HC4) df1 df2 p

.44658 .19944 22.64833 5.06267 10.00000 210.00000 .00000

Model

coeff se(HC4) t p LLCI ULCI

constant 21.06384 2.76475 7.61872 .00000 15.61362 26.51405

Stigma_I -.39128 .07569 -5.16978 .00000 -.54048 -.24208

Age_cov .02334 .03691 .63218 .52796 -.04943 .09610

Educatio .34671 .85743 .40436 .68636 -1.34356 2.03698

Employme .10730 .88896 .12071 .90404 -1.64513 1.85974

Social_p .43829 .19024 2.30384 .02221 .06326 .81333

Insuranc .47993 1.03206 .46502 .64240 -1.55459 2.51446

Major_Ps -1.89486 .82516 -2.29635 .02264 -3.52151 -.26820

Chemo_co -.43206 .48456 -.89166 .37360 -1.38729 .52316

Mastecto -.79535 .47907 -1.66017 .09837 -1.73976 .14906

Radiothe -.99683 .93056 -1.07121 .28531 -2.83127 .83762

Standardized coefficients

coeff

Stigma_I -.37070

Age_cov .04099

Educatio .02624

Employme .00773

Social_p .12151

Insuranc .02656

Major_Ps -.15604

Chemo_co -.07080

Mastecto -.12324

Radiothe -.09412

| **Benjamini-Hochberg Procedure** | | | | |
| --- | --- | --- | --- | --- |
|  |  | **Ascending P-value s** | **I = ranking** | **(I/10)* 0.10** |
|  | **Stigma** | **.00000** | **1** | **.01** |
|  | **Social participation frequency** | **~~.0222~~** | **2** | **.02** |
|  | **Major Psychological issues** | **~~.0226~~** | **3** | **.03** |
|  | **Mastectomy** | **~~.098~~** | **4** | **.04** |
|  | **Radiotherapy** | **~~.285~~** | **5** | **.05** |
|  | **Chemotherapy** | **~~.373~~** | **6** | **.06** |
|  | **Age** | **~~.527~~** | **7** | **.07** |
|  | **Insurance** | **~~.642~~** | **8** | **.08** |
|  | **Education** | **~~.686~~** | **9** | **.09** |
|  | **Employment** | **~~.904~~** | **10** | **.10** |

************** TOTAL, DIRECT, AND INDIRECT EFFECTS OF X ON Y **************

Total effect of X on Y

Effect se(HC4) t p LLCI ULCI c_ps c_cs

-.39128 .07569 -5.16978 .00000 -.54048 -.24208 -.07530 -.37070

Direct effect of X on Y

Effect se(HC4) t p LLCI ULCI c'_ps c'_cs

-.30803 .07574 -4.06673 .00007 -.45735 -.15871 -.05927 -.29182

Indirect effect(s) of X on Y:

Effect BootSE BootLLCI BootULCI

SOC -.08325 .02499 -.13504 -.03710

Partially standardized indirect effect(s) of X on Y:

Effect BootSE BootLLCI BootULCI

SOC -.01602 .00466 -.02563 -.00732

Completely standardized indirect effect(s) of X on Y:

Effect BootSE BootLLCI BootULCI

SOC -.07887 .02299 -.12504 -.03587

*********************** ANALYSIS NOTES AND ERRORS ************************

Level of confidence for all confidence intervals in output:

95.0000

Number of bootstrap samples for percentile bootstrap confidence intervals:

5000

NOTE: A heteroscedasticity consistent standard error and covariance matrix estimator was used.

***************** PROCESS Procedure for SPSS Version 3.5 *****************

Written by Andrew F. Hayes, Ph.D. www.afhayes.com

Documentation available in Hayes (2018). www.guilford.com/p/hayes3

**************************************************************************

**Model : 4**

**Y : EWB = Emotional Well-being**

**X : Stigma_IV**

**M : CopPosit = Coping Positive Reframing**

Covariates:

Age_cov Educatio Employme Social_p Insuranc Major_Ps Chemo_co Mastecto Radiothe

Sample

Size: 219

**************************************************************************

OUTCOME VARIABLE:

CopPosit

Model Summary

R R-sq MSE F(HC4) df1 df2 p

.33083 .10945 .80153 2.72582 10.00000 208.00000 .00361

Model

coeff se(HC4) t p LLCI ULCI

constant 3.43955 .49862 6.89814 .00000 2.45655 4.42255

Stigma_I -.04736 .01305 -3.62853 .00036 -.07309 -.02163

Age_cov -.00942 .00706 -1.33435 .18355 -.02334 .00450

Educatio -.04722 .21929 -.21534 .82971 -.47955 .38510

Employme .10310 .21406 .48162 .63058 -.31891 .52510

Social_p -.02313 .04172 -.55445 .57987 -.10537 .05911

Insuranc .01560 .20727 .07528 .94007 -.39302 .42422

Major_Ps -.06339 .15590 -.40658 .68473 -.37073 .24396

Chemo_co -.03581 .09497 -.37712 .70647 -.22303 .15141

Mastecto -.01352 .09137 -.14800 .88249 -.19366 .16661

Radiothe .25174 .15921 1.58112 .11537 -.06214 .56562

Standardized coefficients

coeff

Stigma_I -.25100

Age_cov -.09232

Educatio -.02011

Employme .04178

Social_p -.03606

Insuranc .00486

Major_Ps -.02917

Chemo_co -.03285

Mastecto -.01172

Radiothe .13323

| **Benjamini-Hochberg Procedure** | | | | |
| --- | --- | --- | --- | --- |
|  |  | **Ascending P-value s** | **I = ranking** | **(I/10)* 0.10** |
|  | **Stigma** | **.0003** | **1** | **.01** |
|  | **Radiotherapy** | **~~.115~~** | **2** | **.02** |
|  | **Age** | **~~.183~~** | **3** | **.03** |
|  | **Social participation frequency** | **~~.579~~** | **4** | **.04** |
|  | **Employment** | **~~.630~~** | **5** | **.05** |
|  | **Major Psychological issues** | **~~.684~~** | **6** | **.06** |
|  | **Chemotherapy** | **~~.704~~** | **7** | **.07** |
|  | **Education** | **~~.829~~** | **8** | **.08** |
|  | **Mastectomy** | **~~.882~~** | **9** | **.09** |
|  | **Insurance** | **~~.940~~** | **10** | **.10** |

**************************************************************************

OUTCOME VARIABLE:

EWB

Model Summary

R R-sq MSE F(HC4) df1 df2 p

.49435 .24438 21.44165 6.18443 11.00000 207.00000 .00000

Model

coeff se(HC4) t p LLCI ULCI

constant 17.00967 3.19285 5.32743 .00000 10.71500 23.30433

Stigma_I -.33351 .07986 -4.17646 .00004 -.49095 -.17608

CopPosit 1.23627 .37234 3.32031 .00106 .50221 1.97033

Age_cov .02815 .03611 .77946 .43660 -.04305 .09934

Educatio .35807 .78097 .45850 .64708 -1.18160 1.89775

Employme -.00673 .86363 -.00780 .99379 -1.70936 1.69590

Social_p .49010 .17273 2.83743 .00500 .14957 .83063

Insuranc .49662 1.03725 .47879 .63259 -1.54831 2.54156

Major_Ps -1.87756 .78045 -2.40575 .01702 -3.41620 -.33891

Chemo_co -.37528 .46679 -.80395 .42235 -1.29555 .54500

Mastecto -.76102 .46483 -1.63720 .10311 -1.67744 .15539

Radiothe -1.29033 .91314 -1.41308 .15914 -3.09058 .50991

Standardized coefficients

coeff

Stigma_I -.31558

CopPosit .22071

Age_cov .04925

Educatio .02722

Employme -.00049

Social_p .13640

Insuranc .02762

Major_Ps -.15427

Chemo_co -.06146

Mastecto -.11775

Radiothe -.12192

| **Benjamini-Hochberg Procedure** | | | | |
| --- | --- | --- | --- | --- |
|  |  | **Ascending P-value s** | **I = ranking** | **(I/11)* 0.10** |
|  | **Stigma** | **0.00004** | **1** | **.009** |
|  | **Positive reframing coping** | **.00106** | **2** | **.018** |
|  | **Social participation frequency** | **.005** | **3** | **.027** |
|  | **Major Psychological issues** | **.017** | **4** | **.03636** |
|  | **Mastectomy** | **~~.103~~** | **5** | **.045** |
|  | **Radiotherapy** | **~~.159~~** | **6** | **.054** |
|  | **Chemotherapy** | **~~.422~~** | **7** | **.063** |
|  | **Age** | **~~.436~~** | **8** | **.072** |
|  | **Insurance** | **~~.632~~** | **9** | **.081** |
|  | **Education** | **~~.647~~** | **10** | **.0909** |
|  | **Employment** | **~~.993~~** | **11** | **.100** |

************************** TOTAL EFFECT MODEL ****************************

OUTCOME VARIABLE:

EWB

Model Summary

R R-sq MSE F(HC4) df1 df2 p

.44833 .20100 22.56360 5.12883 10.00000 208.00000 .00000

Model

coeff se(HC4) t p LLCI ULCI

constant 21.26190 2.77396 7.66481 .00000 15.79321 26.73059

Stigma_I -.39206 .07628 -5.13953 .00000 -.54245 -.24167

Age_cov .01650 .03704 .44557 .65637 -.05651 .08952

Educatio .29969 .86033 .34834 .72793 -1.39640 1.99579

Employme .12072 .88988 .13566 .89222 -1.63362 1.87507

Social_p .46151 .18804 2.45425 .01494 .09079 .83222

Insuranc .51591 1.03883 .49663 .61997 -1.53207 2.56390

Major_Ps -1.95592 .82956 -2.35777 .01931 -3.59135 -.32049

Chemo_co -.41955 .48302 -.86860 .38607 -1.37179 .53269

Mastecto -.77774 .48024 -1.61948 .10686 -1.72451 .16903

Radiothe -.97912 .92930 -1.05361 .29328 -2.81118 .85294

Standardized coefficients

coeff

Stigma_I -.37097

Age_cov .02887

Educatio .02278

Employme .00873

Social_p .12844

Insuranc .02870

Major_Ps -.16070

Chemo_co -.06871

Mastecto -.12034

Radiothe -.09251

| **Benjamini-Hochberg Procedure** | | | | |
| --- | --- | --- | --- | --- |
|  |  | **Ascending P-value s** | **I = ranking** | **(I/10)* 0.10** |
|  | **Stigma** | **.00000** | **1** | **.01** |
|  | **Social participation frequency** | **.0149** | **2** | **.02** |
|  | **Major Psychological issues** | **.0193** | **3** | **.03** |
|  | **Mastectomy** | **~~.106~~** | **4** | **.04** |
|  | **Radiotherapy** | **~~.293~~** | **5** | **.05** |
|  | **Chemotherapy** | **~~.386~~** | **6** | **.06** |
|  | **Insurance** | **~~.619~~** | **7** | **.07** |
|  | **Age** | **~~.656~~** | **8** | **.08** |
|  | **Education** | **~~.727~~** | **9** | **.09** |
|  | **Employment** | **~~.892~~** | **10** | **.10** |

************** TOTAL, DIRECT, AND INDIRECT EFFECTS OF X ON Y **************

Total effect of X on Y

Effect se(HC4) t p LLCI ULCI c_ps c_cs

-.39206 .07628 -5.13953 .00000 -.54245 -.24167 -.07553 -.37097

Direct effect of X on Y

Effect se(HC4) t p LLCI ULCI c'_ps c'_cs

-.33351 .07986 -4.17646 .00004 -.49095 -.17608 -.06425 -.31558

Indirect effect(s) of X on Y:

Effect BootSE BootLLCI BootULCI

CopPosit -.05855 .02414 -.11099 -.01728

Partially standardized indirect effect(s) of X on Y:

Effect BootSE BootLLCI BootULCI

CopPosit -.01128 .00458 -.02093 -.00334

Completely standardized indirect effect(s) of X on Y:

Effect BootSE BootLLCI BootULCI

CopPosit -.05540 .02284 -.10469 -.01622

*********************** ANALYSIS NOTES AND ERRORS ************************

Level of confidence for all confidence intervals in output:

95.0000

Number of bootstrap samples for percentile bootstrap confidence intervals:

5000

NOTE: A heteroscedasticity consistent standard error and covariance matrix estimator was used.

***************** PROCESS Procedure for SPSS Version 3.5 *****************

Written by Andrew F. Hayes, Ph.D. www.afhayes.com

Documentation available in Hayes (2018). www.guilford.com/p/hayes3

**************************************************************************

**Model : 4**

**Y : EWB**

**X : Stigma_I**

**M1 : Comprehe**

**M2 : Meaningf**

**M3 : CopPosit**

Covariates:

Age_cov Educatio Employme Social_p Insuranc Major_Ps Chemo_co Mastecto Radiothe

Sample

Size: 219

**************************************************************************

OUTCOME VARIABLE:

Comprehe

Model Summary

R R-sq MSE F(HC4) df1 df2 p

.37350 .13950 36.80129 3.53201 10.00000 208.00000 .00025

Model

coeff se(HC4) t p LLCI ULCI

constant 28.40618 3.65373 7.77456 .00000 21.20308 35.60928

Stigma_I -.31516 .09120 -3.45579 .00067 -.49496 -.13537

Age_cov .04333 .04522 .95825 .33905 -.04581 .13247

Educatio -.32797 1.13554 -.28882 .77301 -2.56661 1.91068

Employme 1.81374 1.25488 1.44535 .14986 -.66017 4.28766

Social_p .37360 .34730 1.07574 .28329 -.31107 1.05828

Insuranc -.56031 1.47296 -.38039 .70404 -3.46415 2.34354

Major_Ps -1.83801 1.07382 -1.71166 .08845 -3.95498 .27895

Chemo_co -1.13661 .60285 -1.88541 .06077 -2.32508 .05186

Mastecto -1.49233 .58860 -2.53537 .01197 -2.65272 -.33193

Radiothe -1.04589 1.07799 -.97022 .33306 -3.17109 1.07930

Standardized coefficients

coeff

Stigma_I -.24233

Age_cov .06161

Educatio -.02026

Employme .10664

Social_p .08449

Insuranc -.02533

Major_Ps -.12272

Chemo_co -.15126

Mastecto -.18764

Radiothe -.08030

| **Benjamini-Hochberg Procedure** | | | | |
| --- | --- | --- | --- | --- |
|  |  | **Ascending P-value s** | **I = ranking** | **(I/10)* 0.10** |
|  | **Stigma** | **.0006** | **1** | **.01** |
|  | **Mastectomy** | **.011** | **2** | **.02** |
|  | **Chemotherapy** | **~~.060~~** | **3** | **.03** |
|  | **Major Psychological issues** | **~~.088~~** | **4** | **.04** |
|  | **Employment** | **~~.149~~** | **5** | **.05** |
|  | **Social participation frequency** | **~~.283~~** | **6** | **.06** |
|  | **Radiotherapy** | **~~.333~~** | **7** | **.07** |
|  | **Age** | **~~.339~~** | **8** | **.08** |
|  | **Insurance** | **~~.704~~** | **9** | **.09** |
|  | **Education** | **~~.773~~** | **10** | **.10** |

**************************************************************************

OUTCOME VARIABLE:

Meaningf

Model Summary

R R-sq MSE F(HC4) df1 df2 p

.43827 .19208 20.04765 8.29543 10.00000 208.00000 .00000

Model

coeff se(HC4) t p LLCI ULCI

constant 21.99116 2.52608 8.70564 .00000 17.01115 26.97116

Stigma_I -.24362 .06341 -3.84175 .00016 -.36864 -.11860

Age_cov .06993 .03162 2.21159 .02808 .00759 .13228

Educatio -1.54766 1.00758 -1.53602 .12605 -3.53403 .43872

Employme .61881 1.21019 .51133 .60966 -1.76700 3.00462

Social_p .50570 .26189 1.93097 .05485 -.01060 1.02199

Insuranc .00323 1.05806 .00305 .99757 -2.08267 2.08913

Major_Ps -1.36998 .73432 -1.86564 .06350 -2.81765 .07769

Chemo_co -.31410 .43908 -.71536 .47519 -1.17972 .55152

Mastecto -.82346 .44136 -1.86575 .06348 -1.69357 .04664

Radiothe .54173 .77332 .70052 .48439 -.98283 2.06628

Standardized coefficients

coeff

Stigma_I -.24592

Age_cov .13054

Educatio -.12551

Employme .04776

Social_p .15015

Insuranc .00019

Major_Ps -.12008

Chemo_co -.05488

Mastecto -.13593

Radiothe .05460

| **Benjamini-Hochberg Procedure** | | | | |
| --- | --- | --- | --- | --- |
|  |  | **Ascending P-value s** | **I = ranking** | **(I/10)* 0.10** |
|  | **Stigma** | **.0001** | **1** | **.01** |
|  | **Age** | **~~.028~~** | **2** | **.02** |
|  | **Social participation frequency** | **~~.054~~** | **3** | **.03** |
|  | **Mastectomy** | **~~.0634~~** | **4** | **.04** |
|  | **Major Psychological issues** | **~~.0635~~** | **5** | **.05** |
|  | **Education** | **~~.126~~** | **6** | **.06** |
|  | **Chemotherapy** | **~~.475~~** | **7** | **.07** |
|  | **Radiotherapy** | **~~.484~~** | **8** | **.08** |
|  | **Employment** | **~~.609~~** | **9** | **.09** |
|  | **Insurance** | **~~.997~~** | **10** | **.10** |

**************************************************************************

OUTCOME VARIABLE:

CopPosit

Model Summary

R R-sq MSE F(HC4) df1 df2 p

.33083 .10945 .80153 2.72582 10.00000 208.00000 .00361

Model

coeff se(HC4) t p LLCI ULCI

constant 3.43955 .49862 6.89814 .00000 2.45655 4.42255

Stigma_I -.04736 .01305 -3.62853 .00036 -.07309 -.02163

Age_cov -.00942 .00706 -1.33435 .18355 -.02334 .00450

Educatio -.04722 .21929 -.21534 .82971 -.47955 .38510

Employme .10310 .21406 .48162 .63058 -.31891 .52510

Social_p -.02313 .04172 -.55445 .57987 -.10537 .05911

Insuranc .01560 .20727 .07528 .94007 -.39302 .42422

Major_Ps -.06339 .15590 -.40658 .68473 -.37073 .24396

Chemo_co -.03581 .09497 -.37712 .70647 -.22303 .15141

Mastecto -.01352 .09137 -.14800 .88249 -.19366 .16661

Radiothe .25174 .15921 1.58112 .11537 -.06214 .56562

Standardized coefficients

coeff

Stigma_I -.25100

Age_cov -.09232

Educatio -.02011

Employme .04178

Social_p -.03606

Insuranc .00486

Major_Ps -.02917

Chemo_co -.03285

Mastecto -.01172

Radiothe .13323

| **Benjamini-Hochberg Procedure** | | | | |
| --- | --- | --- | --- | --- |
|  |  | **Ascending P-value s** | **I = ranking** | **(I/10)* 0.10** |
|  | **Stigma** | **.0003** | **1** | **.01** |
|  | **Radiotherapy** | **~~.115~~** | **2** | **.02** |
|  | **Age** | **~~.183~~** | **3** | **.03** |
|  | **Social participation frequency** | **~~.579~~** | **4** | **.04** |
|  | **Employment** | **~~.630~~** | **5** | **.05** |
|  | **Major Psychological issues** | **~~.684~~** | **6** | **.06** |
|  | **Chemotherapy** | **~~.706~~** | **7** | **.07** |
|  | **Education** | **~~.829~~** | **8** | **.08** |
|  | **Mastectomy** | **~~.886~~** | **9** | **.09** |
|  | **Insurance** | **~~.940~~** | **10** | **.10** |

**************************************************************************

OUTCOME VARIABLE:

EWB

Model Summary

R R-sq MSE F(HC4) df1 df2 p

.55214 .30486 19.91800 6.78123 13.00000 205.00000 .00000

Model

coeff se(HC4) t p LLCI ULCI

constant 10.19323 3.37665 3.01874 .00286 3.53581 16.85065

Stigma_I -.26118 .07607 -3.43343 .00072 -.41115 -.11120

Comprehe .10444 .05433 1.92237 .05595 -.00267 .21156

Meaningf .22959 .07528 3.04998 .00259 .08118 .37801

CopPosit .88756 .36664 2.42080 .01636 .16469 1.61043

Age_cov .00428 .03618 .11831 .90594 -.06705 .07562

Educatio .73119 .72079 1.01443 .31157 -.68992 2.15231

Employme -.30229 .77411 -.39050 .69657 -1.82852 1.22394

Social_p .32691 .18320 1.78443 .07583 -.03429 .68811

Insuranc .55984 .93443 .59913 .54975 -1.28249 2.40218

Major_Ps -1.39316 .74274 -1.87569 .06212 -2.85755 .07124

Chemo_co -.19694 .45880 -.42924 .66820 -1.10151 .70764

Mastecto -.42081 .44740 -.94058 .34803 -1.30291 .46128

Radiothe -1.21769 .83770 -1.45361 .14758 -2.86931 .43393

Standardized coefficients

coeff

Stigma_I -.24713

Comprehe .12853

Meaningf .21522

CopPosit .15845

Age_cov .00749

Educatio .05558

Employme -.02187

Social_p .09098

Insuranc .03114

Major_Ps -.11447

Chemo_co -.03225

Mastecto -.06511

Radiothe -.11505

| **Benjamini-Hochberg Procedure** | | | | |
| --- | --- | --- | --- | --- |
|  |  | **Ascending P-value s** | **I = ranking** | **(I/13) * 0.10** |
|  | **Stigma** | **.0007** | **1** | **.0076** |
|  | **Meaningfulness** | **.002** | **2** | **.0153** |
|  | **Positive reframing** | **.016** | **3** | **.023** |
|  | **Comprehensibility** | **~~.055~~** | **4** | **.030** |
|  | **Major Psychological issues** | **~~.062~~** | **5** | **.038** |
|  | **Social participation frequency** | **~~.075~~** | **6** | **.046** |
|  | **Radiotherapy** | **~~.147~~** | **7** | **.053** |
|  | **Education** | **~~.311~~** | **8** | **.061** |
|  | **Mastectomy** | **~~.348~~** | **9** | **.069** |
|  | **Insurance** | **~~.549~~** | **10** | **.076** |
|  | **Chemotherapy** | **~~.668~~** | **11** | **.084** |
|  | **Employment** | **~~.696~~** | **12** | **.092** |
|  | **Age** | **~~.905~~** | **13** | **.10** |

************************** TOTAL EFFECT MODEL ****************************

OUTCOME VARIABLE:

EWB

Model Summary

R R-sq MSE F(HC4) df1 df2 p

.44833 .20100 22.56360 5.12883 10.00000 208.00000 .00000

Model

coeff se(HC4) t p LLCI ULCI

constant 21.26190 2.77396 7.66481 .00000 15.79321 26.73059

Stigma_I -.39206 .07628 -5.13953 .00000 -.54245 -.24167

Age_cov .01650 .03704 .44557 .65637 -.05651 .08952

Educatio .29969 .86033 .34834 .72793 -1.39640 1.99579

Employme .12072 .88988 .13566 .89222 -1.63362 1.87507

Social_p .46151 .18804 2.45425 .01494 .09079 .83222

Insuranc .51591 1.03883 .49663 .61997 -1.53207 2.56390

Major_Ps -1.95592 .82956 -2.35777 .01931 -3.59135 -.32049

Chemo_co -.41955 .48302 -.86860 .38607 -1.37179 .53269

Mastecto -.77774 .48024 -1.61948 .10686 -1.72451 .16903

Radiothe -.97912 .92930 -1.05361 .29328 -2.81118 .85294

Standardized coefficients

coeff

Stigma_I -.37097

Age_cov .02887

Educatio .02278

Employme .00873

Social_p .12844

Insuranc .02870

Major_Ps -.16070

Chemo_co -.06871

Mastecto -.12034

Radiothe -.09251

| **Benjamini-Hochberg Procedure** | | | | |
| --- | --- | --- | --- | --- |
|  |  | **Ascending P-value s** | **I = ranking** | **(I/10)* 0.10** |
|  | **Stigma** | **.00000** | **1** | **.01** |
|  | **Social participation frequency** | **.0149** | **2** | **.02** |
|  | **Major Psychological issues** | **.0193** | **3** | **.03** |
|  | **Mastectomy** | **~~.106~~** | **4** | **.04** |
|  | **Radiotherapy** | **~~.293~~** | **5** | **.05** |
|  | **Chemotherapy** | **~~.386~~** | **6** | **.06** |
|  | **Insurance** | **~~.619~~** | **7** | **.07** |
|  | **Age** | **~~.656~~** | **8** | **.08** |
|  | **Education** | **~~.727~~** | **9** | **.09** |
|  | **Employment** | **~~.892~~** | **10** | **.10** |

************** TOTAL, DIRECT, AND INDIRECT EFFECTS OF X ON Y **************

Total effect of X on Y

Effect se(HC4) t p LLCI ULCI c_ps c_cs

-.39206 .07628 -5.13953 .00000 -.54245 -.24167 -.07553 -.37097

Direct effect of X on Y

Effect se(HC4) t p LLCI ULCI c'_ps c'_cs

-.26118 .07607 -3.43343 .00072 -.41115 -.11120 -.05032 -.24713

Indirect effect(s) of X on Y:

Effect BootSE BootLLCI BootULCI

TOTAL -.13088 .03332 -.19823 -.06866

Comprehe -.03292 .01927 -.07351 .00248

Meaningf -.05593 .02368 -.10692 -.01558

CopPosit -.04203 .02133 -.08886 -.00569

Partially standardized indirect effect(s) of X on Y:

Effect BootSE BootLLCI BootULCI

TOTAL -.02521 .00618 -.03780 -.01353

Comprehe -.00634 .00369 -.01409 .00048

Meaningf -.01078 .00452 -.02040 -.00307

CopPosit -.00810 .00408 -.01693 -.00113

Completely standardized indirect effect(s) of X on Y:

Effect BootSE BootLLCI BootULCI

TOTAL -.12384 .03079 -.18637 -.06542

Comprehe -.03115 .01785 -.06825 .00233

Meaningf -.05293 .02254 -.10138 -.01488

CopPosit -.03977 .02018 -.08418 -.00544

*********************** ANALYSIS NOTES AND ERRORS ************************

Level of confidence for all confidence intervals in output:

95.0000

Number of bootstrap samples for percentile bootstrap confidence intervals:

5000

NOTE: A heteroscedasticity consistent standard error and covariance matrix estimator was used.

***************** PROCESS Procedure for SPSS Version 3.5 *****************

Written by Andrew F. Hayes, Ph.D. www.afhayes.com

Documentation available in Hayes (2018). www.guilford.com/p/hayes3

**************************************************************************

**Model : 4**

**Y : FWB = Functional Well-being**

**X : Stigma**

**M : Meaningfulness**

Covariates:

Age_cov Educatio Employme Social_p Insuranc Major_Ps Chemo_co Mastecto Radiothe

Sample

Size: 221

**************************************************************************

OUTCOME VARIABLE:

Meaningf

Model Summary

R R-sq MSE F df1 df2 p

.43435 .18866 20.31616 4.88319 10.00000 210.00000 .00000

Model

coeff se t p LLCI ULCI

constant 22.12763 2.67375 8.27588 .00000 16.85680 27.39846

Stigma_I -.23369 .06482 -3.60503 .00039 -.36147 -.10590

Age_cov .05889 .03442 1.71069 .08861 -.00897 .12674

Educatio -1.59914 .92285 -1.73283 .08459 -3.41839 .22010

Employme .60739 .97747 .62139 .53502 -1.31953 2.53430

Social_p .53412 .21518 2.48215 .01384 .10992 .95831

Insuranc .02574 1.08506 .02372 .98110 -2.11327 2.16475

Major_Ps -1.33952 .73036 -1.83404 .06806 -2.77930 .10026

Chemo_co -.26584 .45987 -.57808 .56383 -1.17240 .64071

Mastecto -.84146 .43347 -1.94122 .05357 -1.69596 .01305

Radiothe .61470 .78626 .78180 .43521 -.93528 2.16468

Standardized coefficients

coeff

Stigma_I -.23532

Age_cov .10993

Educatio -.12862

Employme .04649

Social_p .15739

Insuranc .00151

Major_Ps -.11725

Chemo_co -.04631

Mastecto -.13858

Radiothe .06169

| **Benjamini-Hochberg Procedure** | | | | |
| --- | --- | --- | --- | --- |
|  |  | **Ascending P-value s** | **I = ranking** | **(I/10)* 0.10** |
|  | **Stigma** | **.00039** | **1** | **.01** |
|  | **Social participation frequency** | **.0138** | **2** | **.02** |
|  | **Mastectomy** | **~~.053~~** | **3** | **.03** |
|  | **Major Psychological issues** | **~~.068~~** | **4** | **.04** |
|  | **Education** | **~~.084~~** | **5** | **.05** |
|  | **Age** | **~~.088~~** | **6** | **.06** |
|  | **Radiotherapy** | **~~.435~~** | **7** | **.07** |
|  | **Employment** | **~~.535~~** | **8** | **.08** |
|  | **Chemotherapy** | **~~.563~~** | **9** | **.09** |
|  | **Insurance** | **~~.981~~** | **10** | **.10** |

**************************************************************************

OUTCOME VARIABLE:

FWB

Model Summary

R R-sq MSE F df1 df2 p

.47922 .22965 20.98686 5.66419 11.00000 209.00000 .00000

Model

coeff se t p LLCI ULCI

constant 20.43562 3.12946 6.53009 .00000 14.26627 26.60497

Stigma_I -.27503 .06789 -4.05107 .00007 -.40887 -.14119

Meaningf .21809 .07014 3.10948 .00214 .07982 .35635

Age_cov -.04857 .03523 -1.37875 .16945 -.11802 .02088

Educatio -.84757 .94464 -.89724 .37062 -2.70983 1.01468

Employme -.45063 .99439 -.45317 .65089 -2.41095 1.50969

Social_p .38879 .22189 1.75215 .08121 -.04865 .82622

Insuranc 1.23761 1.10283 1.12222 .26306 -.93648 3.41171

Major_Ps -.41795 .74824 -.55857 .57705 -1.89302 1.05712

Chemo_co -.62688 .46777 -1.34014 .18166 -1.54904 .29528

Mastecto -.83542 .44450 -1.87945 .06157 -1.71170 .04086

Radiothe .20142 .80030 .25168 .80154 -1.37627 1.77911

Standardized coefficients

coeff

Stigma_I -.26616

Meaningf .20958

Age_cov -.08714

Educatio -.06551

Employme -.03315

Social_p .11010

Insuranc .06995

Major_Ps -.03516

Chemo_co -.10494

Mastecto -.13222

Radiothe .01942

| **Benjamini-Hochberg Procedure** | | | | |
| --- | --- | --- | --- | --- |
|  |  | **Ascending P-value s** | **I = ranking** | **(I/11)* 0.10** |
|  | **Stigma** | **.00007** | **1** | **.009** |
|  | **Meaningfulness** | **.002** | **2** | **.018** |
|  | **Mastectomy** | **~~.061~~** | **3** | **.027** |
|  | **Social participation frequency** | **~~.081~~** | **4** | **.03636** |
|  | **Age** | **~~.169~~** | **5** | **.045** |
|  | **Chemotherapy** | **~~.181~~** | **6** | **.054** |
|  | **Insurance** | **~~.263~~** | **7** | **.063** |
|  | **Education** | **~~.370~~** | **8** | **.072** |
|  | **Major Psychological issues** | **~~.577~~** | **9** | **.081** |
|  | **Employment** | **~~.650~~** | **10** | **.0909** |
|  | **Radiotherapy** | **~~.801~~** | **11** | **.100** |

************************** TOTAL EFFECT MODEL ****************************

OUTCOME VARIABLE:

FWB

Model Summary

R R-sq MSE F df1 df2 p

.44047 .19401 21.85321 5.05505 10.00000 210.00000 .00000

Model

coeff se t p LLCI ULCI

constant 25.26138 2.77305 9.10961 .00000 19.79480 30.72796

Stigma_I -.32600 .06723 -4.84902 .00000 -.45853 -.19347

Age_cov -.03573 .03570 -1.00081 .31807 -.10611 .03465

Educatio -1.19633 .95713 -1.24992 .21272 -3.08313 .69048

Employme -.31817 1.01377 -.31384 .75395 -2.31664 1.68031

Social_p .50527 .22317 2.26402 .02459 .06532 .94522

Insuranc 1.24323 1.12536 1.10474 .27054 -.97522 3.46168

Major_Ps -.71008 .75749 -.93742 .34962 -2.20333 .78317

Chemo_co -.68486 .47695 -1.43591 .15252 -1.62508 .25537

Mastecto -1.01893 .44957 -2.26647 .02444 -1.90517 -.13269

Radiothe .33547 .81546 .41139 .68120 -1.27207 1.94302

Standardized coefficients

coeff

Stigma_I -.31548

Age_cov -.06410

Educatio -.09247

Employme -.02340

Social_p .14308

Insuranc .07027

Major_Ps -.05973

Chemo_co -.11464

Mastecto -.16127

Radiothe .03235

| **Benjamini-Hochberg Procedure** | | | | |
| --- | --- | --- | --- | --- |
|  |  | **Ascending P-value s** | **I = ranking** | **(I/10)* 0.10** |
|  | **Stigma** | **.00000** | **1** | **.01** |
|  | **Mastectomy** | **.0244** | **2** | **.02** |
|  | **Social participation frequency** | **.0245** | **3** | **.03** |
|  | **Chemotherapy** | **~~.152~~** | **4** | **.04** |
|  | **Education** | **~~.212~~** | **5** | **.05** |
|  | **Insurance** | **~~.270~~** | **6** | **.06** |
|  | **Age** | **~~.318~~** | **7** | **.07** |
|  | **Major Psychological issues** | **~~.349~~** | **8** | **.08** |
|  | **Radiotherapy** | **~~.681~~** | **9** | **.09** |
|  | **Employment** | **~~.753~~** | **10** | **.10** |

************** TOTAL, DIRECT, AND INDIRECT EFFECTS OF X ON Y **************

Total effect of X on Y

Effect se t p LLCI ULCI c_ps c_cs

-.32600 .06723 -4.84902 .00000 -.45853 -.19347 -.06408 -.31548

Direct effect of X on Y

Effect se t p LLCI ULCI c'_ps c'_cs

-.27503 .06789 -4.05107 .00007 -.40887 -.14119 -.05406 -.26616

Indirect effect(s) of X on Y:

Effect BootSE BootLLCI BootULCI

Meaningf -.05096 .02214 -.10083 -.01416

Partially standardized indirect effect(s) of X on Y:

Effect BootSE BootLLCI BootULCI

Meaningf -.01002 .00427 -.01946 -.00286

Completely standardized indirect effect(s) of X on Y:

Effect BootSE BootLLCI BootULCI

Meaningf -.04932 .02114 -.09730 -.01388

*********************** ANALYSIS NOTES AND ERRORS ************************

Level of confidence for all confidence intervals in output:

95.0000

Number of bootstrap samples for percentile bootstrap confidence intervals:

5000

***************** PROCESS Procedure for SPSS Version 3.5 *****************

Written by Andrew F. Hayes, Ph.D. www.afhayes.com

Documentation available in Hayes (2018). www.guilford.com/p/hayes3

**************************************************************************

**Model : 4**

**Y : FWB = Functional Well-being**

**X : Stigma**

**M : SOC = sense of coherence**

Covariates:

Age_cov Educatio Employme Social_p Insuranc Major_Ps Chemo_co Mastecto Radiothe

Sample

Size: 221

**************************************************************************

OUTCOME VARIABLE:

SOC

Model Summary

R R-sq MSE F df1 df2 p

.45548 .20746 151.84085 5.49712 10.00000 210.00000 .00000

Model

coeff se t p LLCI ULCI

constant 68.01609 7.30961 9.30502 .00000 53.60647 82.42570

Stigma_I -.82220 .17721 -4.63961 .00001 -1.17154 -.47285

Age_cov .16923 .09410 1.79834 .07356 -.01628 .35474

Educatio -.43019 2.52293 -.17051 .86477 -5.40371 4.54333

Employme 3.33713 2.67225 1.24881 .21313 -1.93075 8.60501

Social_p 1.41471 .58828 2.40485 .01705 .25503 2.57440

Insuranc -1.62359 2.96639 -.54733 .58473 -7.47131 4.22413

Major_Ps -4.96124 1.99669 -2.48473 .01375 -8.89738 -1.02511

Chemo_co -1.48699 1.25722 -1.18276 .23824 -3.96537 .99139

Mastecto -2.99414 1.18503 -2.52663 .01225 -5.33022 -.65805

Radiothe -.38276 2.14952 -.17807 .85884 -4.62015 3.85464

Standardized coefficients

coeff

Stigma_I -.29932

Age_cov .11422

Educatio -.01251

Employme .09234

Social_p .15071

Insuranc -.03452

Major_Ps -.15700

Chemo_co -.09364

Mastecto -.17827

Radiothe -.01389

| **Benjamini-Hochberg Procedure** | | | | |
| --- | --- | --- | --- | --- |
|  |  | **Ascending P-value s** | **I = ranking** | **(I/10)* 0.10** |
|  | **Stigma** | **.00001** | **1** | **.01** |
|  | **Mastectomy** | **.012** | **2** | **.02** |
|  | **Major Psychological issues** | **.013** | **3** | **.03** |
|  | **Social participation frequency** | **.017** | **4** | **.04** |
|  | **Age** | **~~.073~~** | **5** | **.05** |
|  | **Employment** | **~~.213~~** | **6** | **.06** |
|  | **Chemotherapy** | **~~.238~~** | **7** | **.07** |
|  | **Insurance** | **~~.584~~** | **8** | **.08** |
|  | **Radiotherapy** | **~~.858~~** | **9** | **.09** |
|  | **Education** | **~~.864~~** | **10** | **.10** |

**************************************************************************

OUTCOME VARIABLE:

FWB

Model Summary

R R-sq MSE F df1 df2 p

.47423 .22489 21.11660 5.51266 11.00000 209.00000 .00000

Model

coeff se t p LLCI ULCI

constant 20.21101 3.23948 6.23896 .00000 13.82476 26.59726

Stigma_I -.26495 .06939 -3.81816 .00018 -.40174 -.12815

SOC .07425 .02573 2.88538 .00432 .02352 .12498

Age_cov -.04829 .03536 -1.36570 .17350 -.11801 .02142

Educatio -1.16438 .94092 -1.23749 .21729 -3.01930 .69053

Employme -.56596 1.00023 -.56583 .57212 -2.53780 1.40588

Social_p .40022 .22238 1.79972 .07335 -.03817 .83862

Insuranc 1.36378 1.10702 1.23194 .21936 -.81857 3.54614

Major_Ps -.34169 .75548 -.45229 .65153 -1.83103 1.14764

Chemo_co -.57444 .47040 -1.22117 .22340 -1.50179 .35290

Mastecto -.79660 .44859 -1.77579 .07722 -1.68095 .08774

Radiothe .36390 .80166 .45393 .65035 -1.21648 1.94428

Standardized coefficients

coeff

Stigma_I -.25640

SOC .19738

Age_cov -.08665

Educatio -.09000

Employme -.04163

Social_p .11334

Insuranc .07708

Major_Ps -.02874

Chemo_co -.09616

Mastecto -.12608

Radiothe .03509

| **Benjamini-Hochberg Procedure** | | | | |
| --- | --- | --- | --- | --- |
|  |  | **Ascending P-value s** | **I = ranking** | **(I/11)* 0.10** |
|  | **Stigma** | **.00018** | **1** | **.009** |
|  | **Sense of Coherence** | **.004** | **2** | **.018** |
|  | **Mastectomy** | **~~.077~~** | **3** | **.027** |
|  | **Social participation frequency** | **~~.073~~** | **4** | **.03636** |
|  | **Age** | **~~.173~~** | **5** | **.045** |
|  | **Education** | **~~.217~~** | **6** | **.054** |
|  | **Insurance** | **~~.219~~** | **7** | **.063** |
|  | **Chemotherapy** | **~~.223~~** | **8** | **.072** |
|  | **Employment** | **~~.572~~** | **9** | **.081** |
|  | **Radiotherapy** | **~~.650~~** | **10** | **.0909** |
|  | **Major Psychological issues** | **~~.651~~** | **11** | **.100** |

************************** TOTAL EFFECT MODEL ****************************

OUTCOME VARIABLE:

FWB

Model Summary

R R-sq MSE F df1 df2 p

.44047 .19401 21.85321 5.05505 10.00000 210.00000 .00000

Model

coeff se t p LLCI ULCI

constant 25.26138 2.77305 9.10961 .00000 19.79480 30.72796

Stigma_I -.32600 .06723 -4.84902 .00000 -.45853 -.19347

Age_cov -.03573 .03570 -1.00081 .31807 -.10611 .03465

Educatio -1.19633 .95713 -1.24992 .21272 -3.08313 .69048

Employme -.31817 1.01377 -.31384 .75395 -2.31664 1.68031

Social_p .50527 .22317 2.26402 .02459 .06532 .94522

Insuranc 1.24323 1.12536 1.10474 .27054 -.97522 3.46168

Major_Ps -.71008 .75749 -.93742 .34962 -2.20333 .78317

Chemo_co -.68486 .47695 -1.43591 .15252 -1.62508 .25537

Mastecto -1.01893 .44957 -2.26647 .02444 -1.90517 -.13269

Radiothe .33547 .81546 .41139 .68120 -1.27207 1.94302

Standardized coefficients

coeff

Stigma_I -.31548

Age_cov -.06410

Educatio -.09247

Employme -.02340

Social_p .14308

Insuranc .07027

Major_Ps -.05973

Chemo_co -.11464

Mastecto -.16127

Radiothe .03235

| **Benjamini-Hochberg Procedure** | | | | |
| --- | --- | --- | --- | --- |
|  |  | **Ascending P-value s** | **I = ranking** | **(I/10)* 0.10** |
|  | **Stigma** | **.00000** | **1** | **.01** |
|  | **Mastectomy** | **.0244** | **2** | **.02** |
|  | **Social participation frequency** | **.0245** | **3** | **.03** |
|  | **Chemotherapy** | **~~.152~~** | **4** | **.04** |
|  | **Education** | **~~.212~~** | **5** | **.05** |
|  | **Insurance** | **~~.270~~** | **6** | **.06** |
|  | **Age** | **~~.318~~** | **7** | **.07** |
|  | **Major Psychological issues** | **~~.349~~** | **8** | **.08** |
|  | **Radiotherapy** | **~~.681~~** | **9** | **.09** |
|  | **Employment** | **~~.753~~** | **10** | **.10** |

************** TOTAL, DIRECT, AND INDIRECT EFFECTS OF X ON Y **************

Total effect of X on Y

Effect se t p LLCI ULCI c_ps c_cs

-.32600 .06723 -4.84902 .00000 -.45853 -.19347 -.06408 -.31548

Direct effect of X on Y

Effect se t p LLCI ULCI c'_ps c'_cs

-.26495 .06939 -3.81816 .00018 -.40174 -.12815 -.05208 -.25640

Indirect effect(s) of X on Y:

Effect BootSE BootLLCI BootULCI

SOC -.06105 .02335 -.10862 -.01688

Partially standardized indirect effect(s) of X on Y:

Effect BootSE BootLLCI BootULCI

SOC -.01200 .00456 -.02138 -.00334

Completely standardized indirect effect(s) of X on Y:

Effect BootSE BootLLCI BootULCI

SOC -.05908 .02230 -.10379 -.01654

*********************** ANALYSIS NOTES AND ERRORS ************************

Level of confidence for all confidence intervals in output:

95.0000

Number of bootstrap samples for percentile bootstrap confidence intervals:

5000

***************** PROCESS Procedure for SPSS Version 3.5 *****************

Written by Andrew F. Hayes, Ph.D. www.afhayes.com

Documentation available in Hayes (2018). www.guilford.com/p/hayes3

**************************************************************************

**Model : 4**

**Y : FWB = Functional Well-being**

**X : Stigma**

**M : Emotional Informational**

Covariates:

Age_cov Educatio Employme Social_p Insuranc Major_Ps Chemo_co Mastecto Radiothe

Sample

Size: 221

**************************************************************************

OUTCOME VARIABLE:

Emoinfo

Model Summary

R R-sq MSE F df1 df2 p

.36001 .12960 .06415 3.12695 10.00000 210.00000 .00096

Model

coeff se t p LLCI ULCI

constant .51430 .15025 3.42300 .00074 .21811 .81049

Stigma_I -.01133 .00364 -3.10925 .00214 -.01851 -.00414

Age_cov .00164 .00193 .84938 .39664 -.00217 .00546

Educatio -.01597 .05186 -.30795 .75843 -.11820 .08626

Employme -.01900 .05493 -.34599 .72969 -.12728 .08928

Social_p .00346 .01209 .28655 .77474 -.02037 .02730

Insuranc .04089 .06097 .67054 .50325 -.07931 .16108

Major_Ps -.13273 .04104 -3.23408 .00142 -.21364 -.05183

Chemo_co -.00108 .02584 -.04187 .96664 -.05202 .04986

Mastecto -.01604 .02436 -.65839 .51101 -.06406 .03198

Radiothe .03875 .04418 .87701 .38148 -.04835 .12585

Standardized coefficients

coeff

Stigma_I -.21022

Age_cov .05653

Educatio -.02368

Employme -.02681

Social_p .01882

Insuranc .04432

Major_Ps -.21415

Chemo_co -.00347

Mastecto -.04868

Radiothe .07168

| **Benjamini-Hochberg Procedure** | | | | |
| --- | --- | --- | --- | --- |
|  |  | **Ascending P-value s** | **I = ranking** | **(I/10)* 0.10** |
|  | **Major Psychological issues** | **.001** | **1** | **.01** |
|  | **Stigma** | **.002** | **2** | **.02** |
|  | **Radiotherapy** | **~~.381~~** | **3** | **.03** |
|  | **Age** | **~~.396~~** | **4** | **.04** |
|  | **Insurance** | **~~.503~~** | **5** | **.05** |
|  | **Mastectomy** | **~~.511~~** | **6** | **.06** |
|  | **Employment** | **~~.729~~** | **7** | **.07** |
|  | **Education** | **~~.758~~** | **8** | **.08** |
|  | **Social participation frequency** | **~~.774~~** | **9** | **.09** |
|  | **Chemotherapy** | **~~.966~~** | **10** | **.10** |

**************************************************************************

OUTCOME VARIABLE:

FWB

Model Summary

R R-sq MSE F df1 df2 p

.50356 .25357 20.33514 6.45466 11.00000 209.00000 .00000

Model

coeff se t p LLCI ULCI

constant 22.68102 2.74861 8.25181 .00000 17.26247 28.09958

Stigma_I -.26917 .06633 -4.05820 .00007 -.39993 -.13841

Emoinfo 5.01723 1.22859 4.08374 .00006 2.59522 7.43924

Age_cov -.04397 .03450 -1.27466 .20384 -.11198 .02403

Educatio -1.11620 .92349 -1.20868 .22815 -2.93676 .70435

Employme -.22282 .97821 -.22778 .82004 -2.15123 1.70560

Social_p .48789 .21533 2.26581 .02449 .06340 .91238

Insuranc 1.03810 1.08673 .95525 .34056 -1.10426 3.18045

Major_Ps -.04413 .74868 -.05895 .95305 -1.52006 1.43180

Chemo_co -.67943 .46009 -1.47673 .14125 -1.58644 .22758

Mastecto -.93846 .43412 -2.16177 .03177 -1.79428 -.08265

Radiothe .14106 .78807 .17900 .85811 -1.41252 1.69464

Standardized coefficients

coeff

Stigma_I -.26049

Emoinfo .26159

Age_cov -.07889

Educatio -.08628

Employme -.01639

Social_p .13816

Insuranc .05867

Major_Ps -.00371

Chemo_co -.11373

Mastecto -.14853

Radiothe .01360

| **Benjamini-Hochberg Procedure** | | | | |
| --- | --- | --- | --- | --- |
|  |  | **Ascending P-value s** | **I = ranking** | **(I/11)* 0.10** |
|  | **Stigma** | **.00007** | **1** | **.009** |
|  | **Emotional informational support** | **.00006** | **2** | **.018** |
|  | **Social participation frequency** | **.024** | **3** | **.027** |
|  | **Mastectomy** | **.031** | **4** | **.03636** |
|  | **Chemotherapy** | **~~.141~~** | **5** | **.045** |
|  | **Age** | **~~.203~~** | **6** | **.054** |
|  | **Education** | **~~.227~~** | **7** | **.063** |
|  | **Insurance** | **~~.340~~** | **8** | **.072** |
|  | **Employment** | **~~.820~~** | **9** | **.081** |
|  | **Radiotherapy** | **~~.858~~** | **10** | **.0909** |
|  | **Major Psychological issues** | **~~.953~~** | **11** | **.100** |

************************** TOTAL EFFECT MODEL ****************************

OUTCOME VARIABLE:

FWB

Model Summary

R R-sq MSE F df1 df2 p

.44047 .19401 21.85321 5.05505 10.00000 210.00000 .00000

Model

coeff se t p LLCI ULCI

constant 25.26138 2.77305 9.10961 .00000 19.79480 30.72796

Stigma_I -.32600 .06723 -4.84902 .00000 -.45853 -.19347

Age_cov -.03573 .03570 -1.00081 .31807 -.10611 .03465

Educatio -1.19633 .95713 -1.24992 .21272 -3.08313 .69048

Employme -.31817 1.01377 -.31384 .75395 -2.31664 1.68031

Social_p .50527 .22317 2.26402 .02459 .06532 .94522

Insuranc 1.24323 1.12536 1.10474 .27054 -.97522 3.46168

Major_Ps -.71008 .75749 -.93742 .34962 -2.20333 .78317

Chemo_co -.68486 .47695 -1.43591 .15252 -1.62508 .25537

Mastecto -1.01893 .44957 -2.26647 .02444 -1.90517 -.13269

Radiothe .33547 .81546 .41139 .68120 -1.27207 1.94302

Standardized coefficients

coeff

Stigma_I -.31548

Age_cov -.06410

Educatio -.09247

Employme -.02340

Social_p .14308

Insuranc .07027

Major_Ps -.05973

Chemo_co -.11464

Mastecto -.16127

Radiothe .03235

| **Benjamini-Hochberg Procedure** | | | | |
| --- | --- | --- | --- | --- |
|  |  | **Ascending P-value s** | **I = ranking** | **(I/10)* 0.10** |
|  | **Stigma** | **.00000** | **1** | **.01** |
|  | **Mastectomy** | **.0244** | **2** | **.02** |
|  | **Social participation frequency** | **.0245** | **3** | **.03** |
|  | **Chemotherapy** | **~~.152~~** | **4** | **.04** |
|  | **Education** | **~~.212~~** | **5** | **.05** |
|  | **Insurance** | **~~.270~~** | **6** | **.06** |
|  | **Age** | **~~.318~~** | **7** | **.07** |
|  | **Major Psychological issues** | **~~.349~~** | **8** | **.08** |
|  | **Radiotherapy** | **~~.681~~** | **9** | **.09** |
|  | **Employment** | **~~.753~~** | **10** | **.10** |

************** TOTAL, DIRECT, AND INDIRECT EFFECTS OF X ON Y **************

Total effect of X on Y

Effect se t p LLCI ULCI c_ps c_cs

-.32600 .06723 -4.84902 .00000 -.45853 -.19347 -.06408 -.31548

Direct effect of X on Y

Effect se t p LLCI ULCI c'_ps c'_cs

-.26917 .06633 -4.05820 .00007 -.39993 -.13841 -.05291 -.26049

Indirect effect(s) of X on Y:

Effect BootSE BootLLCI BootULCI

Emoinfo -.05682 .02157 -.10298 -.01969

Partially standardized indirect effect(s) of X on Y:

Effect BootSE BootLLCI BootULCI

Emoinfo -.01117 .00412 -.01972 -.00398

Completely standardized indirect effect(s) of X on Y:

Effect BootSE BootLLCI BootULCI

Emoinfo -.05499 .02052 -.09823 -.01874

*********************** ANALYSIS NOTES AND ERRORS ************************

Level of confidence for all confidence intervals in output:

95.0000

Number of bootstrap samples for percentile bootstrap confidence intervals:

5000

***************** PROCESS Procedure for SPSS Version 3.5 *****************

Written by Andrew F. Hayes, Ph.D. www.afhayes.com

Documentation available in Hayes (2018). www.guilford.com/p/hayes3

**************************************************************************

**Model : 4**

**Y : FWB = Functional Well-being**

**X : Stigma**

**M : Affectionate**

Covariates:

Age_cov Educatio Employme Social_p Insuranc Major_Ps Chemo_co Mastecto Radiothe

Sample

Size: 221

**************************************************************************

OUTCOME VARIABLE:

Affectio

Model Summary

R R-sq MSE F df1 df2 p

.37797 .14286 .05276 3.50013 10.00000 210.00000 .00027

Model

coeff se t p LLCI ULCI

constant .48253 .13625 3.54144 .00049 .21393 .75112

Stigma_I -.00910 .00330 -2.75500 .00638 -.01561 -.00259

Age_cov .00105 .00175 .59605 .55178 -.00241 .00450

Educatio -.01455 .04703 -.30944 .75729 -.10726 .07815

Employme -.02925 .04981 -.58719 .55770 -.12744 .06894

Social_p .00296 .01097 .27031 .78719 -.01865 .02458

Insuranc .07932 .05529 1.43455 .15290 -.02968 .18832

Major_Ps -.11830 .03722 -3.17845 .00170 -.19167 -.04493

Chemo_co -.00828 .02343 -.35349 .72408 -.05448 .03791

Mastecto -.03515 .02209 -1.59143 .11302 -.07870 .00839

Radiothe .05027 .04007 1.25454 .21104 -.02872 .12925

Standardized coefficients

coeff

Stigma_I -.18484

Age_cov .03937

Educatio -.02361

Employme -.04515

Social_p .01762

Insuranc .09410

Major_Ps -.20885

Chemo_co -.02910

Mastecto -.11678

Radiothe .10175

| **Benjamini-Hochberg Procedure** | | | | |
| --- | --- | --- | --- | --- |
|  |  | **Ascending P-value s** | **I = ranking** | **(I/10)* 0.10** |
|  | **Major Psychological issues** | **.001** | **1** | **.01** |
|  | **Stigma** | **.006** | **2** | **.02** |
|  | **Mastectomy** | **~~.113~~** | **3** | **.03** |
|  | **Insurance** | **~~.152~~** | **4** | **.04** |
|  | **Radiotherapy** | **~~.211~~** | **5** | **.05** |
|  | **Age** | **~~.551~~** | **6** | **.06** |
|  | **Employment** | **~~.557~~** | **7** | **.07** |
|  | **Education** | **~~.757~~** | **8** | **.08** |
|  | **Chemotherapy** | **~~.724~~** | **9** | **.09** |
|  | **Social participation frequency** | **~~.787~~** | **10** | **.10** |

**************************************************************************

OUTCOME VARIABLE:

FWB

Model Summary

R R-sq MSE F df1 df2 p

.50126 .25126 20.39810 6.37609 11.00000 209.00000 .00000

Model

coeff se t p LLCI ULCI

constant 22.64405 2.75798 8.21038 .00000 17.20702 28.08107

Stigma_I -.27663 .06612 -4.18407 .00004 -.40697 -.14629

Affectio 5.42424 1.35689 3.99755 .00009 2.74929 8.09918

Age_cov -.04140 .03452 -1.19930 .23177 -.10945 .02665

Educatio -1.11739 .92492 -1.20809 .22838 -2.94077 .70598

Employme -.15952 .98024 -.16273 .87089 -2.09195 1.77292

Social_p .48919 .21565 2.26842 .02433 .06406 .91433

Insuranc .81297 1.09256 .74409 .45766 -1.34089 2.96683

Major_Ps -.06841 .74923 -.09131 .92734 -1.54543 1.40861

Chemo_co -.63992 .46094 -1.38831 .16652 -1.54860 .26876

Mastecto -.82825 .43695 -1.89551 .05940 -1.68965 .03315

Radiothe .06282 .79079 .07944 .93676 -1.49613 1.62177

Standardized coefficients

coeff

Stigma_I -.26771

Affectio .25844

Age_cov -.07428

Educatio -.08637

Employme -.01173

Social_p .13853

Insuranc .04595

Major_Ps -.00575

Chemo_co -.10712

Mastecto -.13109

Radiothe .00606

| **Benjamini-Hochberg Procedure** | | | | |
| --- | --- | --- | --- | --- |
|  |  | **Ascending P-value s** | **I = ranking** | **(I/11)* 0.10** |
|  | **Stigma** | **.00004** | **1** | **.009** |
|  | **Affectionate support** | **.00009** | **2** | **.018** |
|  | **Social participation frequency** | **.024** | **3** | **.027** |
|  | **Mastectomy** | **~~.059~~** | **4** | **.03636** |
|  | **Chemotherapy** | **~~.166~~** | **5** | **.045** |
|  | **Education** | **~~.228~~** | **6** | **.054** |
|  | **Age** | **~~.231~~** | **7** | **.063** |
|  | **Insurance** | **~~.457~~** | **8** | **.072** |
|  | **Employment** | **~~.870~~** | **9** | **.081** |
|  | **Major Psychological issues** | **~~.927~~** | **10** | **.0909** |
|  | **Radiotherapy** | **~~.936~~** | **11** | **.100** |

************************** TOTAL EFFECT MODEL ****************************

OUTCOME VARIABLE:

FWB

Model Summary

R R-sq MSE F df1 df2 p

.44047 .19401 21.85321 5.05505 10.00000 210.00000 .00000

Model

coeff se t p LLCI ULCI

constant 25.26138 2.77305 9.10961 .00000 19.79480 30.72796

Stigma_I -.32600 .06723 -4.84902 .00000 -.45853 -.19347

Age_cov -.03573 .03570 -1.00081 .31807 -.10611 .03465

Educatio -1.19633 .95713 -1.24992 .21272 -3.08313 .69048

Employme -.31817 1.01377 -.31384 .75395 -2.31664 1.68031

Social_p .50527 .22317 2.26402 .02459 .06532 .94522

Insuranc 1.24323 1.12536 1.10474 .27054 -.97522 3.46168

Major_Ps -.71008 .75749 -.93742 .34962 -2.20333 .78317

Chemo_co -.68486 .47695 -1.43591 .15252 -1.62508 .25537

Mastecto -1.01893 .44957 -2.26647 .02444 -1.90517 -.13269

Radiothe .33547 .81546 .41139 .68120 -1.27207 1.94302

Standardized coefficients

coeff

Stigma_I -.31548

Age_cov -.06410

Educatio -.09247

Employme -.02340

Social_p .14308

Insuranc .07027

Major_Ps -.05973

Chemo_co -.11464

Mastecto -.16127

Radiothe .03235

| **Benjamini-Hochberg Procedure** | | | | |
| --- | --- | --- | --- | --- |
|  |  | **Ascending P-value s** | **I = ranking** | **(I/10)* 0.10** |
|  | **Stigma** | **.00000** | **1** | **.01** |
|  | **Mastectomy** | **.0244** | **2** | **.02** |
|  | **Social participation frequency** | **.0245** | **3** | **.03** |
|  | **Chemotherapy** | **~~.152~~** | **4** | **.04** |
|  | **Education** | **~~.212~~** | **5** | **.05** |
|  | **Insurance** | **~~.270~~** | **6** | **.06** |
|  | **Age** | **~~.318~~** | **7** | **.07** |
|  | **Major Psychological issues** | **~~.349~~** | **8** | **.08** |
|  | **Radiotherapy** | **~~.681~~** | **9** | **.09** |
|  | **Employment** | **~~.753~~** | **10** | **.10** |

************** TOTAL, DIRECT, AND INDIRECT EFFECTS OF X ON Y **************

Total effect of X on Y

Effect se t p LLCI ULCI c_ps c_cs

-.32600 .06723 -4.84902 .00000 -.45853 -.19347 -.06408 -.31548

Direct effect of X on Y

Effect se t p LLCI ULCI c'_ps c'_cs

-.27663 .06612 -4.18407 .00004 -.40697 -.14629 -.05438 -.26771

Indirect effect(s) of X on Y:

Effect BootSE BootLLCI BootULCI

Affectio -.04936 .02157 -.09537 -.01108

Partially standardized indirect effect(s) of X on Y:

Effect BootSE BootLLCI BootULCI

Affectio -.00970 .00415 -.01837 -.00221

Completely standardized indirect effect(s) of X on Y:

Effect BootSE BootLLCI BootULCI

Affectio -.04777 .02099 -.09270 -.01039

*********************** ANALYSIS NOTES AND ERRORS ************************

Level of confidence for all confidence intervals in output:

95.0000

Number of bootstrap samples for percentile bootstrap confidence intervals:

5000

***************** PROCESS Procedure for SPSS Version 3.5 *****************

Written by Andrew F. Hayes, Ph.D. www.afhayes.com

Documentation available in Hayes (2018). www.guilford.com/p/hayes3

**************************************************************************

**Model : 4**

**Y : FWB = Functional Well-being**

**X : Stigma**

**M : Positive social interaction**

Covariates:

Age_cov Educatio Employme Social_p Insuranc Major_Ps Chemo_co Mastecto Radiothe

Sample

Size: 221

**************************************************************************

OUTCOME VARIABLE:

Positive

Model Summary

R R-sq MSE F df1 df2 p

.34064 .11604 .05623 2.75668 10.00000 210.00000 .00325

Model

coeff se t p LLCI ULCI

constant .52551 .14067 3.73583 .00024 .24821 .80281

Stigma_I -.01295 .00341 -3.79812 .00019 -.01968 -.00623

Age_cov .00060 .00181 .33404 .73868 -.00297 .00417

Educatio .01962 .04855 .40417 .68650 -.07609 .11533

Employme .02577 .05143 .50115 .61679 -.07560 .12715

Social_p .00119 .01132 .10550 .91608 -.02112 .02351

Insuranc .05715 .05709 1.00117 .31790 -.05538 .16969

Major_Ps -.08447 .03842 -2.19820 .02903 -.16021 -.00872

Chemo_co -.00020 .02419 -.00842 .99329 -.04790 .04749

Mastecto -.00918 .02281 -.40250 .68773 -.05414 .03578

Radiothe .02742 .04137 .66295 .50809 -.05412 .10897

Standardized coefficients

coeff

Stigma_I -.25878

Age_cov .02241

Educatio .03131

Employme .03914

Social_p .00698

Insuranc .06669

Major_Ps -.14669

Chemo_co -.00070

Mastecto -.02999

Radiothe .05460

| **Benjamini-Hochberg Procedure** | | | | |
| --- | --- | --- | --- | --- |
|  |  | **Ascending P-value s** | **I = ranking** | **(I/10)* 0.10** |
|  | **Stigma** | **.0001** | **1** | **.01** |
|  | **Major Psychological issues** | **~~.029~~** | **2** | **.02** |
|  | **Insurance** | **~~.317~~** | **3** | **.03** |
|  | **Radiotherapy** | **~~.508~~** | **4** | **.04** |
|  | **Employment** | **~~.616~~** | **5** | **.05** |
|  | **Education** | **~~.686~~** | **6** | **.06** |
|  | **Mastectomy** | **~~.687~~** | **7** | **.07** |
|  | **Age** | **~~.738~~** | **8** | **.08** |
|  | **Social participation frequency** | **~~.916~~** | **9** | **.09** |
|  | **Chemotherapy** | **~~.993~~** | **10** | **.10** |

**************************************************************************

OUTCOME VARIABLE:

FWB

Model Summary

R R-sq MSE F df1 df2 p

.51535 .26558 20.00804 6.87079 11.00000 209.00000 .00000

Model

coeff se t p LLCI ULCI

constant 22.17438 2.74015 8.09240 .00000 16.77250 27.57625

Stigma_I -.24991 .06650 -3.75794 .00022 -.38101 -.11881

Positive 5.87429 1.30166 4.51291 .00001 3.30822 8.44036

Age_cov -.03928 .03417 -1.14966 .25160 -.10664 .02808

Educatio -1.31160 .91618 -1.43159 .15375 -3.11775 .49455

Employme -.46956 .97061 -.48378 .62905 -2.38300 1.44388

Social_p .49826 .21355 2.33319 .02059 .07727 .91924

Insuranc .90749 1.07937 .84076 .40144 -1.22035 3.03534

Major_Ps -.21391 .73309 -.29179 .77074 -1.65911 1.23130

Chemo_co -.68366 .45637 -1.49803 .13563 -1.58334 .21602

Mastecto -.96501 .43033 -2.24246 .02598 -1.81336 -.11665

Radiothe .17438 .78109 .22325 .82356 -1.36545 1.71421

Standardized coefficients

coeff

Stigma_I -.24184

Positive .28454

Age_cov -.07048

Educatio -.10138

Employme -.03454

Social_p .14110

Insuranc .05129

Major_Ps -.01799

Chemo_co -.11444

Mastecto -.15273

Radiothe .01682

| **Benjamini-Hochberg Procedure** | | | | |
| --- | --- | --- | --- | --- |
|  |  | **Ascending P-value s** | **I = ranking** | **(I/11)* 0.10** |
|  | **Psitive social interaction** | **.00001** | **1** | **.009** |
|  | **Stigma** | **.00022** | **2** | **.018** |
|  | **Social participation frequency** | **.020** | **3** | **.027** |
|  | **Mastectomy** | **.025** | **4** | **.03636** |
|  | **Chemotherapy** | **~~.135~~** | **5** | **.045** |
|  | **Education** | **~~.153~~** | **6** | **.054** |
|  | **Age** | **~~.251~~** | **7** | **.063** |
|  | **Insurance** | **~~.401~~** | **8** | **.072** |
|  | **Employment** | **~~.629~~** | **9** | **.081** |
|  | **Major Psychological issues** | **~~.770~~** | **10** | **.0909** |
|  | **Radiotherapy** | **~~.823~~** | **11** | **.100** |

************************** TOTAL EFFECT MODEL ****************************

OUTCOME VARIABLE:

FWB

Model Summary

R R-sq MSE F df1 df2 p

.44047 .19401 21.85321 5.05505 10.00000 210.00000 .00000

Model

coeff se t p LLCI ULCI

constant 25.26138 2.77305 9.10961 .00000 19.79480 30.72796

Stigma_I -.32600 .06723 -4.84902 .00000 -.45853 -.19347

Age_cov -.03573 .03570 -1.00081 .31807 -.10611 .03465

Educatio -1.19633 .95713 -1.24992 .21272 -3.08313 .69048

Employme -.31817 1.01377 -.31384 .75395 -2.31664 1.68031

Social_p .50527 .22317 2.26402 .02459 .06532 .94522

Insuranc 1.24323 1.12536 1.10474 .27054 -.97522 3.46168

Major_Ps -.71008 .75749 -.93742 .34962 -2.20333 .78317

Chemo_co -.68486 .47695 -1.43591 .15252 -1.62508 .25537

Mastecto -1.01893 .44957 -2.26647 .02444 -1.90517 -.13269

Radiothe .33547 .81546 .41139 .68120 -1.27207 1.94302

Standardized coefficients

coeff

Stigma_I -.31548

Age_cov -.06410

Educatio -.09247

Employme -.02340

Social_p .14308

Insuranc .07027

Major_Ps -.05973

Chemo_co -.11464

Mastecto -.16127

Radiothe .03235

| **Benjamini-Hochberg Procedure** | | | | |
| --- | --- | --- | --- | --- |
|  |  | **Ascending P-value s** | **I = ranking** | **(I/10)* 0.10** |
|  | **Stigma** | **.00000** | **1** | **.01** |
|  | **Mastectomy** | **.0244** | **2** | **.02** |
|  | **Social participation frequency** | **.0245** | **3** | **.03** |
|  | **Chemotherapy** | **~~.152~~** | **4** | **.04** |
|  | **Education** | **~~.212~~** | **5** | **.05** |
|  | **Insurance** | **~~.270~~** | **6** | **.06** |
|  | **Age** | **~~.318~~** | **7** | **.07** |
|  | **Major Psychological issues** | **~~.349~~** | **8** | **.08** |
|  | **Radiotherapy** | **~~.681~~** | **9** | **.09** |
|  | **Employment** | **~~.753~~** | **10** | **.10** |

************** TOTAL, DIRECT, AND INDIRECT EFFECTS OF X ON Y **************

Total effect of X on Y

Effect se t p LLCI ULCI c_ps c_cs

-.32600 .06723 -4.84902 .00000 -.45853 -.19347 -.06408 -.31548

Direct effect of X on Y

Effect se t p LLCI ULCI c'_ps c'_cs

-.24991 .06650 -3.75794 .00022 -.38101 -.11881 -.04912 -.24184

Indirect effect(s) of X on Y:

Effect BootSE BootLLCI BootULCI

Positive -.07609 .02556 -.13070 -.02965

Partially standardized indirect effect(s) of X on Y:

Effect BootSE BootLLCI BootULCI

Positive -.01496 .00484 -.02517 -.00605

Completely standardized indirect effect(s) of X on Y:

Effect BootSE BootLLCI BootULCI

Positive -.07363 .02451 -.12510 -.02874

*********************** ANALYSIS NOTES AND ERRORS ************************

Level of confidence for all confidence intervals in output:

95.0000

Number of bootstrap samples for percentile bootstrap confidence intervals:

5000

***************** PROCESS Procedure for SPSS Version 3.5 *****************

Written by Andrew F. Hayes, Ph.D. www.afhayes.com

Documentation available in Hayes (2018). www.guilford.com/p/hayes3

**************************************************************************

**Model : 4**

**Y : FWB = Functional well-being**

**X : Stigma**

**M : MOS_SSS = total Social support**

Covariates:

Age_cov Educatio Employme Social_p Insuranc Major_Ps Chemo_co Mastecto Radiothe

Sample

Size: 221

**************************************************************************

OUTCOME VARIABLE:

MOS_SSS

Model Summary

R R-sq MSE F df1 df2 p

.35287 .12451 .06905 2.98667 10.00000 210.00000 .00153

Model

coeff se t p LLCI ULCI

constant .46456 .15587 2.98037 .00322 .15728 .77183

Stigma_I -.01095 .00378 -2.89783 .00416 -.01840 -.00350

Age_cov .00169 .00201 .84386 .39971 -.00226 .00565

Educatio -.03744 .05380 -.69584 .48730 -.14349 .06862

Employme -.02411 .05698 -.42304 .67270 -.13644 .08823

Social_p -.00090 .01254 -.07168 .94292 -.02563 .02383

Insuranc .07701 .06326 1.21750 .22478 -.04768 .20171

Major_Ps -.12186 .04258 -2.86202 .00464 -.20579 -.03792

Chemo_co -.00198 .02681 -.07371 .94131 -.05483 .05087

Mastecto -.01605 .02527 -.63501 .52612 -.06586 .03377

Radiothe .05665 .04584 1.23597 .21785 -.03371 .14701

Standardized coefficients

coeff

Stigma_I -.19649

Age_cov .05633

Educatio -.05365

Employme -.03288

Social_p -.00472

Insuranc .08071

Major_Ps -.19006

Chemo_co -.00613

Mastecto -.04709

Radiothe .10131

| **Benjamini-Hochberg Procedure** | | | | |
| --- | --- | --- | --- | --- |
|  |  | **Ascending P-value s** | **I = ranking** | **(I/10)* 0.10** |
|  | **Stigma** | **.0041** | **1** | **.01** |
|  | **Major Psychological issues** | **.0046** | **2** | **.02** |
|  | **Radiotherapy** | **~~.217~~** | **3** | **.03** |
|  | **Insurance** | **~~.224~~** | **4** | **.04** |
|  | **Age** | **~~.399~~** | **5** | **.05** |
|  | **Education** | **~~.487~~** | **6** | **.06** |
|  | **Mastectomy** | **~~.526~~** | **7** | **.07** |
|  | **Employment** | **~~.672~~** | **8** | **.08** |
|  | **Chemotherapy** | **~~.941~~** | **9** | **.09** |
|  | **Social participation frequency** | **~~.942~~** | **10** | **.10** |

**************************************************************************

OUTCOME VARIABLE:

FWB

Model Summary

R R-sq MSE F df1 df2 p

.50269 .25270 20.35902 6.42480 11.00000 209.00000 .00000

Model

coeff se t p LLCI ULCI

constant 23.03129 2.73259 8.42838 .00000 17.64432 28.41826

Stigma_I -.27343 .06617 -4.13189 .00005 -.40388 -.14297

MOS_SSS 4.80048 1.18495 4.05120 .00007 2.46449 7.13647

Age_cov -.04386 .03452 -1.27064 .20527 -.11190 .02419

Educatio -1.01662 .92489 -1.09918 .27296 -2.83993 .80669

Employme -.20244 .97892 -.20680 .83636 -2.13227 1.72738

Social_p .50959 .21541 2.36564 .01891 .08493 .93425

Insuranc .87352 1.09003 .80137 .42383 -1.27535 3.02239

Major_Ps -.12510 .74525 -.16786 .86686 -1.59428 1.34408

Chemo_co -.67537 .46036 -1.46704 .14387 -1.58292 .23218

Mastecto -.94190 .43434 -2.16856 .03125 -1.79815 -.08564

Radiothe .06351 .78995 .08040 .93599 -1.49377 1.62080

Standardized coefficients

coeff

Stigma_I -.26461

MOS_SSS .25890

Age_cov -.07869

Educatio -.07858

Employme -.01489

Social_p .14431

Insuranc .04937

Major_Ps -.01052

Chemo_co -.11305

Mastecto -.14908

Radiothe .00613

| **Benjamini-Hochberg Procedure** | | | | |
| --- | --- | --- | --- | --- |
|  |  | **Ascending P-value s** | **I = ranking** | **(I/11)* 0.10** |
|  | **Stigma** | **.00005** | **1** | **.009** |
|  | **Total social support** | **.00007** | **2** | **.018** |
|  | **Social participation frequency** | **.018** | **3** | **.027** |
|  | **Mastectomy** | **.031** | **4** | **.03636** |
|  | **Chemotherapy** | **~~.143~~** | **5** | **.045** |
|  | **Age** | **~~.205~~** | **6** | **.054** |
|  | **Education** | **~~.272~~** | **7** | **.063** |
|  | **Insurance** | **~~.423~~** | **8** | **.072** |
|  | **Employment** | **~~.836~~** | **9** | **.081** |
|  | **Major Psychological issues** | **~~.866~~** | **10** | **.0909** |
|  | **Radiotherapy** | **~~.935~~** | **11** | **.100** |

************************** TOTAL EFFECT MODEL ****************************

OUTCOME VARIABLE:

FWB

Model Summary

R R-sq MSE F df1 df2 p

.44047 .19401 21.85321 5.05505 10.00000 210.00000 .00000

Model

coeff se t p LLCI ULCI

constant 25.26138 2.77305 9.10961 .00000 19.79480 30.72796

Stigma_I -.32600 .06723 -4.84902 .00000 -.45853 -.19347

Age_cov -.03573 .03570 -1.00081 .31807 -.10611 .03465

Educatio -1.19633 .95713 -1.24992 .21272 -3.08313 .69048

Employme -.31817 1.01377 -.31384 .75395 -2.31664 1.68031

Social_p .50527 .22317 2.26402 .02459 .06532 .94522

Insuranc 1.24323 1.12536 1.10474 .27054 -.97522 3.46168

Major_Ps -.71008 .75749 -.93742 .34962 -2.20333 .78317

Chemo_co -.68486 .47695 -1.43591 .15252 -1.62508 .25537

Mastecto -1.01893 .44957 -2.26647 .02444 -1.90517 -.13269

Radiothe .33547 .81546 .41139 .68120 -1.27207 1.94302

Standardized coefficients

coeff

Stigma_I -.31548

Age_cov -.06410

Educatio -.09247

Employme -.02340

Social_p .14308

Insuranc .07027

Major_Ps -.05973

Chemo_co -.11464

Mastecto -.16127

Radiothe .03235

| **Benjamini-Hochberg Procedure** | | | | |
| --- | --- | --- | --- | --- |
|  |  | **Ascending P-value s** | **I = ranking** | **(I/10)* 0.10** |
|  | **Stigma** | **.00000** | **1** | **.01** |
|  | **Mastectomy** | **.0244** | **2** | **.02** |
|  | **Social participation frequency** | **.0245** | **3** | **.03** |
|  | **Chemotherapy** | **~~.152~~** | **4** | **.04** |
|  | **Education** | **~~.212~~** | **5** | **.05** |
|  | **Insurance** | **~~.270~~** | **6** | **.06** |
|  | **Age** | **~~.318~~** | **7** | **.07** |
|  | **Major Psychological issues** | **~~.349~~** | **8** | **.08** |
|  | **Radiotherapy** | **~~.681~~** | **9** | **.09** |
|  | **Employment** | **~~.753~~** | **10** | **.10** |

************** TOTAL, DIRECT, AND INDIRECT EFFECTS OF X ON Y **************

Total effect of X on Y

Effect se t p LLCI ULCI c_ps c_cs

-.32600 .06723 -4.84902 .00000 -.45853 -.19347 -.06408 -.31548

Direct effect of X on Y

Effect se t p LLCI ULCI c'_ps c'_cs

-.27343 .06617 -4.13189 .00005 -.40388 -.14297 -.05375 -.26461

Indirect effect(s) of X on Y:

Effect BootSE BootLLCI BootULCI

MOS_SSS -.05257 .02037 -.09540 -.01620

Partially standardized indirect effect(s) of X on Y:

Effect BootSE BootLLCI BootULCI

MOS_SSS -.01033 .00388 -.01850 -.00326

Completely standardized indirect effect(s) of X on Y:

Effect BootSE BootLLCI BootULCI

MOS_SSS -.05087 .01960 -.09233 -.01568

*********************** ANALYSIS NOTES AND ERRORS ************************

Level of confidence for all confidence intervals in output:

95.0000

Number of bootstrap samples for percentile bootstrap confidence intervals:

5000

***************** PROCESS Procedure for SPSS Version 3.5 *****************

Written by Andrew F. Hayes, Ph.D. www.afhayes.com

Documentation available in Hayes (2018). www.guilford.com/p/hayes3

**************************************************************************

**Model : 4**

**Y : FWB = Functional Well-being**

**X : Stigma**

**M : CopPosit = Positive reframing coping**

Covariates:

Age_cov Educatio Employme Social_p Insuranc Major_Ps Chemo_co Mastecto Radiothe

Sample

Size: 219

**************************************************************************

OUTCOME VARIABLE:

CopPosit

Model Summary

R R-sq MSE F df1 df2 p

.33083 .10945 .80153 2.55639 10.00000 208.00000 .00623

Model

coeff se t p LLCI ULCI

constant 3.43955 .53168 6.46918 .00000 2.39137 4.48773

Stigma_I -.04736 .01300 -3.64170 .00034 -.07299 -.02172

Age_cov -.00942 .00691 -1.36268 .17446 -.02305 .00421

Educatio -.04722 .18338 -.25752 .79703 -.40875 .31430

Employme .10310 .19421 .53086 .59608 -.27977 .48596

Social_p -.02313 .04283 -.54008 .58972 -.10756 .06130

Insuranc .01560 .21558 .07238 .94237 -.40939 .44060

Major_Ps -.06339 .14611 -.43382 .66487 -.35143 .22466

Chemo_co -.03581 .09155 -.39120 .69605 -.21630 .14467

Mastecto -.01352 .08634 -.15663 .87569 -.18374 .15669

Radiothe .25174 .15646 1.60896 .10914 -.05671 .56018

Standardized coefficients

coeff

Stigma_I -.25100

Age_cov -.09232

Educatio -.02011

Employme .04178

Social_p -.03606

Insuranc .00486

Major_Ps -.02917

Chemo_co -.03285

Mastecto -.01172

Radiothe .13323

| **Benjamini-Hochberg Procedure** | | | | |
| --- | --- | --- | --- | --- |
|  |  | **Ascending P-value s** | **I = ranking** | **(I/10)* 0.10** |
|  | **Stigma** | **.0003** | **1** | **.01** |
|  | **Radiotherapy** | **~~.109~~** | **2** | **.02** |
|  | **Age** | **~~.174~~** | **3** | **.03** |
|  | **Social participation frequency** | **~~.589~~** | **4** | **.04** |
|  | **Employment** | **~~.596~~** | **5** | **.05** |
|  | **Major Psychological issues** | **~~.664~~** | **6** | **.06** |
|  | **Chemotherapy** | **~~.696~~** | **7** | **.07** |
|  | **Education** | **~~.797~~** | **8** | **.08** |
|  | **Mastectomy** | **~~.875~~** | **9** | **.09** |
|  | **Insurance** | **~~.942~~** | **10** | **.10** |

**************************************************************************

OUTCOME VARIABLE:

FWB

Model Summary

R R-sq MSE F df1 df2 p

.48355 .23382 21.01883 5.74277 11.00000 207.00000 .00000

Model

coeff se t p LLCI ULCI

constant 21.39455 2.98405 7.16963 .00000 15.51152 27.27758

Stigma_I -.26913 .06868 -3.91840 .00012 -.40453 -.13372

CopPosit 1.14818 .35507 3.23368 .00142 .44816 1.84820

Age_cov -.02946 .03556 -.82865 .40826 -.09956 .04063

Educatio -1.16680 .93922 -1.24231 .21553 -3.01847 .68486

Employme -.43652 .99518 -.43863 .66138 -2.39851 1.52547

Social_p .54483 .21947 2.48251 .01384 .11215 .97751

Insuranc 1.23967 1.10395 1.12293 .26277 -.93677 3.41610

Major_Ps -.64318 .74855 -.85923 .39121 -2.11895 .83258

Chemo_co -.62786 .46898 -1.33878 .18211 -1.55246 .29673

Mastecto -1.00418 .44217 -2.27101 .02418 -1.87593 -.13244

Radiothe .07016 .80618 .08703 .93074 -1.51922 1.65953

Standardized coefficients

coeff

Stigma_I -.25899

CopPosit .20847

Age_cov -.05243

Educatio -.09021

Employme -.03212

Social_p .15422

Insuranc .07013

Major_Ps -.05375

Chemo_co -.10458

Mastecto -.15803

Radiothe .00674

| **Benjamini-Hochberg Procedure** | | | | |
| --- | --- | --- | --- | --- |
|  |  | **Ascending P-value s** | **I = ranking** | **(I/11)* 0.10** |
|  | **Stigma** | **.00012** | **1** | **.009** |
|  | **Positive reframing** | **.0014** | **2** | **.018** |
|  | **Social participation frequency** | **.013** | **3** | **.027** |
|  | **Mastectomy** | **.024** | **4** | **.03636** |
|  | **Chemotherapy** | **~~.182~~** | **5** | **.045** |
|  | **Education** | **~~.215~~** | **6** | **.054** |
|  | **Insurance** | **~~.262~~** | **7** | **.063** |
|  | **Major Psychological issues** | **~~.391~~** | **8** | **.072** |
|  | **Age** | **~~.408~~** | **9** | **.081** |
|  | **Employment** | **~~.661~~** | **10** | **.0909** |
|  | **Radiotherapy** | **~~.930~~** | **11** | **.100** |

************************** TOTAL EFFECT MODEL ****************************

OUTCOME VARIABLE:

FWB

Model Summary

R R-sq MSE F df1 df2 p

.44172 .19511 21.97445 5.04214 10.00000 208.00000 .00000

Model

coeff se t p LLCI ULCI

constant 25.34378 2.78389 9.10371 .00000 19.85551 30.83204

Stigma_I -.32350 .06809 -4.75111 .00000 -.45774 -.18927

Age_cov -.04028 .03619 -1.11286 .26705 -.11163 .03107

Educatio -1.22102 .96018 -1.27166 .20491 -3.11396 .67191

Employme -.31815 1.01686 -.31287 .75469 -2.32283 1.68653

Social_p .51827 .22424 2.31120 .02180 .07619 .96035

Insuranc 1.25758 1.12876 1.11413 .26651 -.96769 3.48285

Major_Ps -.71596 .76503 -.93585 .35043 -2.22418 .79226

Chemo_co -.66898 .47935 -1.39561 .16432 -1.61399 .27602

Mastecto -1.01971 .45209 -2.25556 .02514 -1.91097 -.12845

Radiothe .35920 .81922 .43846 .66151 -1.25584 1.97423

Standardized coefficients

coeff

Stigma_I -.31132

Age_cov -.07168

Educatio -.09440

Employme -.02341

Social_p .14670

Insuranc .07114

Major_Ps -.05983

Chemo_co -.11143

Mastecto -.16047

Radiothe .03452

| **Benjamini-Hochberg Procedure** | | | | |
| --- | --- | --- | --- | --- |
|  |  | **Ascending P-value s** | **I = ranking** | **(I/10)* 0.10** |
|  | **Stigma** | **.00000** | **1** | **.01** |
|  | **Social participation frequency** | **.0218** | **2** | **.02** |
|  | **Mastectomy** | **.0251** | **3** | **.03** |
|  | **Chemotherapy** | **~~.164~~** | **4** | **.04** |
|  | **Education** | **~~.204~~** | **5** | **.05** |
|  | **Insurance** | **~~.266~~** | **6** | **.06** |
|  | **Age** | **~~.267~~** | **7** | **.07** |
|  | **Major Psychological issues** | **~~.350~~** | **8** | **.08** |
|  | **Radiotherapy** | **~~.661~~** | **9** | **.09** |
|  | **Employment** | **~~.754~~** | **10** | **.10** |

************** TOTAL, DIRECT, AND INDIRECT EFFECTS OF X ON Y **************

Total effect of X on Y

Effect se t p LLCI ULCI c_ps c_cs

-.32350 .06809 -4.75111 .00000 -.45774 -.18927 -.06338 -.31132

Direct effect of X on Y

Effect se t p LLCI ULCI c'_ps c'_cs

-.26913 .06868 -3.91840 .00012 -.40453 -.13372 -.05273 -.25899

Indirect effect(s) of X on Y:

Effect BootSE BootLLCI BootULCI

CopPosit -.05437 .02142 -.09916 -.01580

Partially standardized indirect effect(s) of X on Y:

Effect BootSE BootLLCI BootULCI

CopPosit -.01065 .00408 -.01918 -.00314

Completely standardized indirect effect(s) of X on Y:

Effect BootSE BootLLCI BootULCI

CopPosit -.05233 .02053 -.09547 -.01525

*********************** ANALYSIS NOTES AND ERRORS ************************

Level of confidence for all confidence intervals in output:

95.0000

Number of bootstrap samples for percentile bootstrap confidence intervals:

5000

***************** PROCESS Procedure for SPSS Version 3.5 *****************

Written by Andrew F. Hayes, Ph.D. www.afhayes.com

Documentation available in Hayes (2018). www.guilford.com/p/hayes3

**************************************************************************

**Model : 4**

**Y : FWB**

**X : Stigma_I**

**M1 : Meaningfulness**

**M2 : MOS_SSS = total social support**

**M3 : CopPosit = Positive reframing coping**

Covariates:

Age_cov Educatio Employme Social_p Insuranc Major_Ps Chemo_co Mastecto Radiothe

Sample

Size: 219

**************************************************************************

OUTCOME VARIABLE:

Meaningf

Model Summary

R R-sq MSE F df1 df2 p

.45240 .20467 152.58618 5.35250 10.00000 208.00000 .00000

Model

coeff se t p LLCI ULCI

constant 67.70659 7.33587 9.22953 .00000 53.24440 82.16878

Stigma_I -.82021 .17942 -4.57135 .00001 -1.17393 -.46649

Age_cov .17944 .09537 1.88142 .06131 -.00858 .36746

Educatio -.35814 2.53018 -.14155 .88758 -5.34623 4.62996

Employme 3.31463 2.67955 1.23701 .21748 -1.96792 8.59719

Social_p 1.37936 .59091 2.33431 .02053 .21443 2.54429

Insuranc -1.67999 2.97440 -.56482 .57281 -7.54382 4.18384

Major_Ps -4.86044 2.01595 -2.41099 .01678 -8.83476 -.88613

Chemo_co -1.50360 1.26314 -1.19037 .23526 -3.99380 .98659

Mastecto -3.02388 1.19130 -2.53830 .01187 -5.37245 -.67530

Radiothe -.40592 2.15873 -.18804 .85103 -4.66172 3.84988

Standardized coefficients

coeff

Stigma_I -.29776

Age_cov .12046

Educatio -.01045

Employme .09201

Social_p .14729

Insuranc -.03585

Major_Ps -.15321

Chemo_co -.09448

Mastecto -.17951

Radiothe -.01471

| **Benjamini-Hochberg Procedure** | | | | |
| --- | --- | --- | --- | --- |
|  |  | **Ascending P-value s** | **I = ranking** | **(I/10)* 0.10** |
|  | **Stigma** | **.00001** | **1** | **.01** |
|  | **Mastectomy** | **.011** | **2** | **.02** |
|  | **Major Psychological issues** | **.016** | **3** | **.03** |
|  | **Social participation frequency** | **.020** | **4** | **.04** |
|  | **Age** | **~~.061~~** | **5** | **.05** |
|  | **Employment** | **~~.217~~** | **6** | **.06** |
|  | **Chemotherapy** | **~~.235~~** | **7** | **.07** |
|  | **Insurance** | **~~.572~~** | **8** | **.08** |
|  | **Radiotherapy** | **~~.851~~** | **9** | **.09** |
|  | **Education** | **~~.887~~** | **10** | **.10** |

**************************************************************************

OUTCOME VARIABLE:

MOS_SSS

Model Summary

R R-sq MSE F df1 df2 p

.34356 .11803 .06736 2.78361 10.00000 208.00000 .00299

Model

coeff se t p LLCI ULCI

constant .45133 .15413 2.92819 .00379 .14747 .75519

Stigma_I -.00955 .00377 -2.53373 .01202 -.01698 -.00212

Age_cov .00133 .00200 .66567 .50636 -.00262 .00528

Educatio -.03675 .05316 -.69133 .49013 -.14156 .06805

Employme -.02766 .05630 -.49132 .62372 -.13865 .08333

Social_p -.00085 .01242 -.06857 .94540 -.02533 .02362

Insuranc .07432 .06249 1.18918 .23572 -.04889 .19752

Major_Ps -.10845 .04236 -2.56037 .01117 -.19195 -.02495

Chemo_co .00229 .02654 .08621 .93138 -.05003 .05461

Mastecto -.02110 .02503 -.84289 .40026 -.07044 .02825

Radiothe .06329 .04536 1.39536 .16440 -.02613 .15271

Standardized coefficients

coeff

Stigma_I -.17379

Age_cov .04488

Educatio -.05372

Employme -.03848

Social_p -.00456

Insuranc .07949

Major_Ps -.17134

Chemo_co .00721

Mastecto -.06277

Radiothe .11499

| **Benjamini-Hochberg Procedure** | | | | |
| --- | --- | --- | --- | --- |
|  |  | **Ascending P-value s** | **I = ranking** | **(I/10)* 0.10** |
|  | **Stigma** | **.012** | **1** | **.01** |
|  | **Major Psychological issues** | **.011** | **2** | **.02** |
|  | **Radiotherapy** | **~~.164~~** | **3** | **.03** |
|  | **Insurance** | **~~.235~~** | **4** | **.04** |
|  | **Mastectomy** | **~~.400~~** | **5** | **.05** |
|  | **Education** | **~~.490~~** | **6** | **.06** |
|  | **Age** | **~~.506~~** | **7** | **.07** |
|  | **Employment** | **~~.623~~** | **8** | **.08** |
|  | **Chemotherapy** | **~~.931~~** | **9** | **.09** |
|  | **Social participation frequency** | **~~.945~~** | **10** | **.10** |

**************************************************************************

OUTCOME VARIABLE:

CopPosit

Model Summary

R R-sq MSE F df1 df2 p

.33083 .10945 .80153 2.55639 10.00000 208.00000 .00623

Model

coeff se t p LLCI ULCI

constant 3.43955 .53168 6.46918 .00000 2.39137 4.48773

Stigma_I -.04736 .01300 -3.64170 .00034 -.07299 -.02172

Age_cov -.00942 .00691 -1.36268 .17446 -.02305 .00421

Educatio -.04722 .18338 -.25752 .79703 -.40875 .31430

Employme .10310 .19421 .53086 .59608 -.27977 .48596

Social_p -.02313 .04283 -.54008 .58972 -.10756 .06130

Insuranc .01560 .21558 .07238 .94237 -.40939 .44060

Major_Ps -.06339 .14611 -.43382 .66487 -.35143 .22466

Chemo_co -.03581 .09155 -.39120 .69605 -.21630 .14467

Mastecto -.01352 .08634 -.15663 .87569 -.18374 .15669

Radiothe .25174 .15646 1.60896 .10914 -.05671 .56018

Standardized coefficients

coeff

Stigma_I -.25100

Age_cov -.09232

Educatio -.02011

Employme .04178

Social_p -.03606

Insuranc .00486

Major_Ps -.02917

Chemo_co -.03285

Mastecto -.01172

Radiothe .13323

| **Benjamini-Hochberg Procedure** | | | | |
| --- | --- | --- | --- | --- |
|  |  | **Ascending P-value s** | **I = ranking** | **(I/10)* 0.10** |
|  | **Stigma** | **.0003** | **1** | **.01** |
|  | **Radiotherapy** | **~~.109~~** | **2** | **.02** |
|  | **Age** | **~~.174~~** | **3** | **.03** |
|  | **Social participation frequency** | **~~.589~~** | **4** | **.04** |
|  | **Employment** | **~~.596~~** | **5** | **.05** |
|  | **Major Psychological issues** | **~~.664~~** | **6** | **.06** |
|  | **Chemotherapy** | **~~.696~~** | **7** | **.07** |
|  | **Education** | **~~.797~~** | **8** | **.08** |
|  | **Mastectomy** | **~~.875~~** | **9** | **.09** |
|  | **Insurance** | **~~.942~~** | **10** | **.10** |

**************************************************************************

OUTCOME VARIABLE:

FWB

Model Summary

R R-sq MSE F df1 df2 p

.53455 .28574 19.78562 6.30845 13.00000 205.00000 .00000

Model

coeff se t p LLCI ULCI

constant 17.95914 3.27565 5.48261 .00000 11.50084 24.41743

Stigma_I -.21443 .06912 -3.10216 .00219 -.35072 -.07815

Meaningf .04350 .02632 1.65246 .09997 -.00840 .09540

MOS_SSS 3.67506 1.27166 2.88996 .00427 1.16784 6.18228

CopPosit .80851 .35559 2.27372 .02402 .10743 1.50960

Age_cov -.04537 .03488 -1.30076 .19480 -.11414 .02340

Educatio -1.03220 .91219 -1.13156 .25914 -2.83068 .76628

Employme -.44402 .97099 -.45729 .64795 -2.35844 1.47039

Social_p .48010 .21619 2.22076 .02746 .05386 .90634

Insuranc 1.04492 1.07716 .97007 .33315 -1.07881 3.16865

Major_Ps -.05474 .74249 -.07373 .94130 -1.51864 1.40916

Chemo_co -.58303 .45674 -1.27652 .20322 -1.48354 .31747

Mastecto -.79971 .43565 -1.83567 .06786 -1.65864 .05922

Radiothe -.05927 .78532 -.07547 .93991 -1.60761 1.48907

Standardized coefficients

coeff

Stigma_I -.20636

Meaningf .11531

MOS_SSS .19438

CopPosit .14680

Age_cov -.08074

Educatio -.07980

Employme -.03267

Social_p .13590

Insuranc .05911

Major_Ps -.00457

Chemo_co -.09711

Mastecto -.12585

Radiothe -.00570

| **Benjamini-Hochberg Procedure** | | | | |
| --- | --- | --- | --- | --- |
|  |  | **Ascending P-value s** | **I = ranking** | **(I/13) * 0.10** |
|  | **Stigma** | **.002** | **1** | **.0076** |
|  | **Total social support** | **.004** | **2** | **.0153** |
|  | **Positive reframing** | **.024** | **3** | **.023** |
|  | **Social participation frequency** | **.027** | **4** | **.030** |
|  | **Mastectomy** | **~~.067~~** | **5** | **.038** |
|  | **Meaningfulness** | **~~.099~~** | **6** | **.046** |
|  | **Age** | **~~.194~~** | **7** | **.053** |
|  | **Chemotherapy** | **~~.203~~** | **8** | **.061** |
|  | **Education** | **~~.259~~** | **9** | **.069** |
|  | **Insurance** | **~~.333~~** | **10** | **.076** |
|  | **Employment** | **~~.647~~** | **11** | **.084** |
|  | **Major Psychological issues** | **~~.941~~** | **12** | **.092** |
|  | **Radiotherapy** | **~~.939~~** | **13** | **.10** |

************************** TOTAL EFFECT MODEL ****************************

OUTCOME VARIABLE:

FWB

Model Summary

R R-sq MSE F df1 df2 p

.44172 .19511 21.97445 5.04214 10.00000 208.00000 .00000

Model

coeff se t p LLCI ULCI

constant 25.34378 2.78389 9.10371 .00000 19.85551 30.83204

Stigma_I -.32350 .06809 -4.75111 .00000 -.45774 -.18927

Age_cov -.04028 .03619 -1.11286 .26705 -.11163 .03107

Educatio -1.22102 .96018 -1.27166 .20491 -3.11396 .67191

Employme -.31815 1.01686 -.31287 .75469 -2.32283 1.68653

Social_p .51827 .22424 2.31120 .02180 .07619 .96035

Insuranc 1.25758 1.12876 1.11413 .26651 -.96769 3.48285

Major_Ps -.71596 .76503 -.93585 .35043 -2.22418 .79226

Chemo_co -.66898 .47935 -1.39561 .16432 -1.61399 .27602

Mastecto -1.01971 .45209 -2.25556 .02514 -1.91097 -.12845

Radiothe .35920 .81922 .43846 .66151 -1.25584 1.97423

Standardized coefficients

coeff

Stigma_I -.31132

Age_cov -.07168

Educatio -.09440

Employme -.02341

Social_p .14670

Insuranc .07114

Major_Ps -.05983

Chemo_co -.11143

Mastecto -.16047

Radiothe .03452

| **Benjamini-Hochberg Procedure** | | | | |
| --- | --- | --- | --- | --- |
|  |  | **Ascending P-value s** | **I = ranking** | **(I/10)* 0.10** |
|  | **Stigma** | **.00000** | **1** | **.01** |
|  | **Social participation frequency** | **.0218** | **2** | **.02** |
|  | **Mastectomy** | **.0251** | **3** | **.03** |
|  | **Chemotherapy** | **~~.164~~** | **4** | **.04** |
|  | **Education** | **~~.204~~** | **5** | **.05** |
|  | **Insurance** | **~~.266~~** | **6** | **.06** |
|  | **Age** | **~~.267~~** | **7** | **.07** |
|  | **Major Psychological issues** | **~~.350~~** | **8** | **.08** |
|  | **Radiotherapy** | **~~.661~~** | **9** | **.09** |
|  | **Employment** | **~~.754~~** | **10** | **.10** |

************** TOTAL, DIRECT, AND INDIRECT EFFECTS OF X ON Y **************

Total effect of X on Y

Effect se t p LLCI ULCI c_ps c_cs

-.32350 .06809 -4.75111 .00000 -.45774 -.18927 -.06338 -.31132

Direct effect of X on Y

Effect se t p LLCI ULCI c'_ps c'_cs

-.21443 .06912 -3.10216 .00219 -.35072 -.07815 -.04201 -.20636

Indirect effect(s) of X on Y:

Effect BootSE BootLLCI BootULCI

TOTAL -.10907 .03408 -.17793 -.04562

Meaningf -.03568 .02253 -.08094 .00849

MOS_SSS -.03510 .01773 -.07406 -.00638

CopPosit -.03829 .01928 -.07893 -.00348

Partially standardized indirect effect(s) of X on Y:

Effect BootSE BootLLCI BootULCI

TOTAL -.02137 .00644 -.03420 -.00911

Meaningf -.00699 .00443 -.01593 .00163

MOS_SSS -.00688 .00341 -.01444 -.00126

CopPosit -.00750 .00370 -.01510 -.00072

Completely standardized indirect effect(s) of X on Y:

Effect BootSE BootLLCI BootULCI

TOTAL -.10496 .03241 -.16948 -.04385

Meaningf -.03433 .02158 -.07771 .00828

MOS_SSS -.03378 .01705 -.07162 -.00603

CopPosit -.03685 .01853 -.07595 -.00345

*********************** ANALYSIS NOTES AND ERRORS ************************

Level of confidence for all confidence intervals in output:

95.0000

Number of bootstrap samples for percentile bootstrap confidence intervals:

5000

***************** PROCESS Procedure for SPSS Version 3.5 *****************

Written by Andrew F. Hayes, Ph.D. www.afhayes.com

Documentation available in Hayes (2018). www.guilford.com/p/hayes3

**************************************************************************

**Model : 4**

**Y : BCS = Breast Cancer Specific subscale**

**X : Stigma**

**M : Comprehensibility**

Covariates:

Age_cov Educatio Employme Social_p Insuranc Major_Ps Chemo_co Mastecto Radiothe

Sample

Size: 221

**************************************************************************

OUTCOME VARIABLE:

Comprehe

Model Summary

R R-sq MSE F df1 df2 p

.38011 .14448 36.53598 3.54648 10.00000 210.00000 .00023

Model

coeff se t p LLCI ULCI

constant 28.41039 3.58559 7.92350 .00000 21.34203 35.47875

Stigma_I -.32272 .08693 -3.71244 .00026 -.49408 -.15135

Age_cov .04775 .04616 1.03439 .30214 -.04325 .13875

Educatio -.31523 1.23758 -.25471 .79919 -2.75489 2.12444

Employme 1.82890 1.31082 1.39523 .16442 -.75515 4.41296

Social_p .36516 .28857 1.26541 .20713 -.20371 .93402

Insuranc -.55790 1.45510 -.38341 .70181 -3.42638 2.31059

Major_Ps -1.89152 .97944 -1.93123 .05480 -3.82232 .03927

Chemo_co -1.16488 .61670 -1.88888 .06029 -2.38060 .05085

Mastecto -1.47027 .58130 -2.52930 .01216 -2.61619 -.32435

Radiothe -1.08926 1.05440 -1.03306 .30277 -3.16783 .98931

Standardized coefficients

coeff

Stigma_I -.24884

Age_cov .06826

Educatio -.01941

Employme .10719

Social_p .08239

Insuranc -.02513

Major_Ps -.12678

Chemo_co -.15537

Mastecto -.18542

Radiothe -.08370

| **Benjamini-Hochberg Procedure** | | | | |
| --- | --- | --- | --- | --- |
|  |  | **Ascending P-value s** | **I = ranking** | **(I/10)* 0.10** |
|  | **Stigma** | **.0002** | **1** | **.01** |
|  | **Mastectomy** | **.0121** | **2** | **.02** |
|  | **Major Psychological issues** | **~~.054~~** | **3** | **.03** |
|  | **Chemotherapy** | **~~.060~~** | **4** | **.04** |
|  | **Employment** | **~~.164~~** | **5** | **.05** |
|  | **Social participation frequency** | **~~.207~~** | **6** | **.06** |
|  | **Age** | **~~.3021~~** | **7** | **.07** |
|  | **Radiotherapy** | **~~.3027~~** | **8** | **.08** |
|  | **Insurance** | **~~.701~~** | **9** | **.09** |
|  | **Education** | **~~.799~~** | **10** | **.10** |

**************************************************************************

OUTCOME VARIABLE:

BCS

Model Summary

R R-sq MSE F df1 df2 p

.47793 .22842 25.33746 5.62484 11.00000 209.00000 .00000

Model

coeff se t p LLCI ULCI

constant 20.92953 3.40314 6.15007 .00000 14.22065 27.63841

Stigma_I -.37379 .07473 -5.00202 .00000 -.52111 -.22647

Comprehe .17913 .05747 3.11714 .00208 .06584 .29242

Age_cov .03699 .03854 .95989 .33822 -.03898 .11297

Educatio -1.52078 1.03077 -1.47539 .14161 -3.55282 .51125

Employme -1.37449 1.09665 -1.25335 .21148 -3.53640 .78743

Social_p .32528 .24122 1.34844 .17898 -.15027 .80082

Insuranc 1.53423 1.21218 1.26568 .20704 -.85544 3.92390

Major_Ps -.63300 .82285 -.76928 .44260 -2.25515 .98915

Chemo_co -.47615 .51791 -.91937 .35896 -1.49715 .54485

Mastecto .08755 .49140 .17817 .85876 -.88118 1.05629

Radiothe .12826 .88030 .14570 .88430 -1.60714 1.86366

Standardized coefficients

coeff

Stigma_I -.32948

Comprehe .20477

Age_cov .06045

Educatio -.10707

Employme -.09209

Social_p .08390

Insuranc .07898

Major_Ps -.04850

Chemo_co -.07260

Mastecto .01262

Radiothe .01127

| **Benjamini-Hochberg Procedure** | | | | |
| --- | --- | --- | --- | --- |
|  |  | **Ascending P-value s** | **I = ranking** | **(I/11)* 0.10** |
|  | **Stigma** | **.00000** | **1** | **.009** |
|  | **Comprehensibility** | **.002** | **2** | **.018** |
|  | **Social participation frequency** | **~~.178~~** | **3** | **.027** |
|  | **Education** | **~~.141~~** | **4** | **.03636** |
|  | **Employment** | **~~.211~~** | **5** | **.045** |
|  | **Insurance** | **~~.207~~** | **6** | **.054** |
|  | **Age** | **~~.338~~** | **7** | **.063** |
|  | **Chemotherapy** | **~~.358~~** | **8** | **.072** |
|  | **Major Psychological issues** | **~~.442~~** | **9** | **.081** |
|  | **Mastectomy** | **~~.858~~** | **10** | **.0909** |
|  | **Radiotherapy** | **~~.884~~** | **11** | **.100** |

************************** TOTAL EFFECT MODEL ****************************

OUTCOME VARIABLE:

BCS

Model Summary

R R-sq MSE F df1 df2 p

.43881 .19255 26.38915 5.00781 10.00000 210.00000 .00000

Model

coeff se t p LLCI ULCI

constant 26.01867 3.04728 8.53832 .00000 20.01149 32.02586

Stigma_I -.43160 .07388 -5.84209 .00000 -.57724 -.28596

Age_cov .04555 .03923 1.16098 .24697 -.03179 .12288

Educatio -1.57725 1.05178 -1.49960 .13522 -3.65065 .49615

Employme -1.04688 1.11403 -.93972 .34844 -3.24299 1.14924

Social_p .39069 .24524 1.59304 .11265 -.09277 .87414

Insuranc 1.43430 1.23665 1.15982 .24744 -1.00354 3.87213

Major_Ps -.97183 .83240 -1.16751 .24433 -2.61275 .66910

Chemo_co -.68481 .52412 -1.30661 .19278 -1.71802 .34839

Mastecto -.17582 .49403 -.35589 .72228 -1.14970 .79807

Radiothe -.06686 .89611 -.07461 .94060 -1.83337 1.69966

Standardized coefficients

coeff

Stigma_I -.38043

Age_cov .07443

Educatio -.11104

Employme -.07014

Social_p .10077

Insuranc .07384

Major_Ps -.07446

Chemo_co -.10441

Mastecto -.02535

Radiothe -.00587

| **Benjamini-Hochberg Procedure** | | | | |
| --- | --- | --- | --- | --- |
|  |  | **Ascending P-value s** | **I = ranking** | **(I/10)* 0.10** |
|  | **Stigma** | **.00000** | **1** | **.01** |
|  | **Social participation frequency** | **~~.112~~** | **2** | **.02** |
|  | **Education** | **~~.135~~** | **3** | **.03** |
|  | **Chemotherapy** | **~~.192~~** | **4** | **.04** |
|  | **Major Psychological issues** | **~~.244~~** | **5** | **.05** |
|  | **Age** | **~~.246~~** | **6** | **.06** |
|  | **Insurance** | **~~.247~~** | **7** | **.07** |
|  | **Employment** | **~~.348~~** | **8** | **.08** |
|  | **Mastectomy** | **~~.722~~** | **9** | **.09** |
|  | **Radiotherapy** | **~~.940~~** | **10** | **.10** |

************** TOTAL, DIRECT, AND INDIRECT EFFECTS OF X ON Y **************

Total effect of X on Y

Effect se t p LLCI ULCI c_ps c_cs

-.43160 .07388 -5.84209 .00000 -.57724 -.28596 -.07727 -.38043

Direct effect of X on Y

Effect se t p LLCI ULCI c'_ps c'_cs

-.37379 .07473 -5.00202 .00000 -.52111 -.22647 -.06692 -.32948

Indirect effect(s) of X on Y:

Effect BootSE BootLLCI BootULCI

Comprehe -.05781 .02394 -.11187 -.01714

Partially standardized indirect effect(s) of X on Y:

Effect BootSE BootLLCI BootULCI

Comprehe -.01035 .00428 -.01986 -.00312

Completely standardized indirect effect(s) of X on Y:

Effect BootSE BootLLCI BootULCI

Comprehe -.05095 .02060 -.09603 -.01539

*********************** ANALYSIS NOTES AND ERRORS ************************

Level of confidence for all confidence intervals in output:

95.0000

Number of bootstrap samples for percentile bootstrap confidence intervals:

5000

***************** PROCESS Procedure for SPSS Version 3.5 *****************

Written by Andrew F. Hayes, Ph.D. www.afhayes.com

Documentation available in Hayes (2018). www.guilford.com/p/hayes3

**************************************************************************

**Model : 4**

**Y : BCS = Breast Cancer Specific subscale**

**X : Stigma**

**M : Manageability**

Covariates:

Age_cov Educatio Employme Social_p Insuranc Major_Ps Chemo_co Mastecto Radiothe

Sample

Size: 221

**************************************************************************

OUTCOME VARIABLE:

Manageab

Model Summary

R R-sq MSE F df1 df2 p

.35462 .12576 30.37485 3.02083 10.00000 210.00000 .00137

Model

coeff se t p LLCI ULCI

constant 17.47806 3.26932 5.34609 .00000 11.03318 23.92295

Stigma_I -.26580 .07926 -3.35345 .00095 -.42205 -.10955

Age_cov .06260 .04209 1.48725 .13845 -.02037 .14557

Educatio 1.48418 1.12841 1.31528 .18985 -.74029 3.70865

Employme .90084 1.19520 .75372 .45186 -1.45528 3.25697

Social_p .51544 .26311 1.95900 .05144 -.00324 1.03413

Insuranc -1.09144 1.32676 -.82264 .41165 -3.70690 1.52403

Major_Ps -1.73020 .89305 -1.93742 .05404 -3.49069 .03028

Chemo_co -.05627 .56231 -.10007 .92038 -1.16476 1.05222

Mastecto -.68241 .53002 -1.28751 .19933 -1.72725 .36244

Radiothe .09180 .96140 .09548 .92402 -1.80343 1.98703

Standardized coefficients

coeff

Stigma_I -.22723

Age_cov .09921

Educatio .10134

Employme .05853

Social_p .12894

Insuranc -.05450

Major_Ps -.12857

Chemo_co -.00832

Mastecto -.09541

Radiothe .00782

| **Benjamini-Hochberg Procedure** | | | | |
| --- | --- | --- | --- | --- |
|  |  | **Ascending P-value s** | **I = ranking** | **(I/10)* 0.10** |
|  | **Stigma** | **.0009** | **1** | **.01** |
|  | **Social participation frequency** | **~~.051~~** | **2** | **.02** |
|  | **Major Psychological issues** | **~~.054~~** | **3** | **.03** |
|  | **Age** | **~~.138~~** | **4** | **.04** |
|  | **Education** | **~~.189~~** | **5** | **.05** |
|  | **Mastectomy** | **~~.199~~** | **6** | **.06** |
|  | **Insurance** | **~~.411~~** | **7** | **.07** |
|  | **Employment** | **~~.451~~** | **8** | **.08** |
|  | **Chemotherapy** | **~~.920~~** | **9** | **.09** |
|  | **Radiotherapy** | **~~.924~~** | **10** | **.10** |

**************************************************************************

OUTCOME VARIABLE:

BCS

Model Summary

R R-sq MSE F df1 df2 p

.46107 .21259 25.85745 5.12964 11.00000 209.00000 .00000

Model

coeff se t p LLCI ULCI

constant 23.45240 3.21515 7.29435 .00000 17.11413 29.79068

Stigma_I -.39257 .07506 -5.22998 .00000 -.54055 -.24460

Manageab .14683 .06367 2.30613 .02208 .02131 .27234

Age_cov .03635 .03904 .93128 .35278 -.04060 .11331

Educatio -1.79517 1.04541 -1.71719 .08742 -3.85607 .26573

Employme -1.17914 1.10424 -1.06784 .28683 -3.35602 .99773

Social_p .31500 .24497 1.28589 .19990 -.16792 .79793

Insuranc 1.59455 1.22610 1.30051 .19486 -.82256 4.01165

Major_Ps -.71779 .83130 -.86345 .38888 -2.35659 .92102

Chemo_co -.67655 .51882 -1.30401 .19366 -1.69935 .34624

Mastecto -.07562 .49095 -.15403 .87773 -1.04347 .89223

Radiothe -.08034 .88705 -.09057 .92792 -1.82905 1.66838

Standardized coefficients

coeff

Stigma_I -.34603

Manageab .15139

Age_cov .05941

Educatio -.12639

Employme -.07900

Social_p .08125

Insuranc .08209

Major_Ps -.05500

Chemo_co -.10315

Mastecto -.01090

Radiothe -.00706

| **Benjamini-Hochberg Procedure** | | | | |
| --- | --- | --- | --- | --- |
|  |  | **Ascending P-value s** | **I = ranking** | **(I/11)* 0.10** |
|  | **Stigma** | **.00000** | **1** | **.009** |
|  | **Manageability** | **~~.022~~** | **2** | **.018** |
|  | **Education** | **~~.087~~** | **3** | **.027** |
|  | **Chemotherapy** | **~~.193~~** | **4** | **.03636** |
|  | **Insurance** | **~~.194~~** | **5** | **.045** |
|  | **Social participation frequency** | **~~.199~~** | **6** | **.054** |
|  | **Employment** | **~~.286~~** | **7** | **.063** |
|  | **Age** | **~~.352~~** | **8** | **.072** |
|  | **Major Psychological issues** | **~~.388~~** | **9** | **.081** |
|  | **Mastectomy** | **~~.877~~** | **10** | **.0909** |
|  | **Radiotherapy** | **~~.927~~** | **11** | **.100** |

************************** TOTAL EFFECT MODEL ****************************

OUTCOME VARIABLE:

BCS

Model Summary

R R-sq MSE F df1 df2 p

.43881 .19255 26.38915 5.00781 10.00000 210.00000 .00000

Model

coeff se t p LLCI ULCI

constant 26.01867 3.04728 8.53832 .00000 20.01149 32.02586

Stigma_I -.43160 .07388 -5.84209 .00000 -.57724 -.28596

Age_cov .04555 .03923 1.16098 .24697 -.03179 .12288

Educatio -1.57725 1.05178 -1.49960 .13522 -3.65065 .49615

Employme -1.04688 1.11403 -.93972 .34844 -3.24299 1.14924

Social_p .39069 .24524 1.59304 .11265 -.09277 .87414

Insuranc 1.43430 1.23665 1.15982 .24744 -1.00354 3.87213

Major_Ps -.97183 .83240 -1.16751 .24433 -2.61275 .66910

Chemo_co -.68481 .52412 -1.30661 .19278 -1.71802 .34839

Mastecto -.17582 .49403 -.35589 .72228 -1.14970 .79807

Radiothe -.06686 .89611 -.07461 .94060 -1.83337 1.69966

Standardized coefficients

coeff

Stigma_I -.38043

Age_cov .07443

Educatio -.11104

Employme -.07014

Social_p .10077

Insuranc .07384

Major_Ps -.07446

Chemo_co -.10441

Mastecto -.02535

Radiothe -.00587

| **Benjamini-Hochberg Procedure** | | | | |
| --- | --- | --- | --- | --- |
|  |  | **Ascending P-value s** | **I = ranking** | **(I/10)* 0.10** |
|  | **Stigma** | **.00000** | **1** | **.01** |
|  | **Social participation frequency** | **~~.112~~** | **2** | **.02** |
|  | **Education** | **~~.135~~** | **3** | **.03** |
|  | **Chemotherapy** | **~~.192~~** | **4** | **.04** |
|  | **Major Psychological issues** | **~~.244~~** | **5** | **.05** |
|  | **Age** | **~~.246~~** | **6** | **.06** |
|  | **Insurance** | **~~.247~~** | **7** | **.07** |
|  | **Employment** | **~~.348~~** | **8** | **.08** |
|  | **Mastectomy** | **~~.722~~** | **9** | **.09** |
|  | **Radiotherapy** | **~~.940~~** | **10** | **.10** |

************** TOTAL, DIRECT, AND INDIRECT EFFECTS OF X ON Y **************

Total effect of X on Y

Effect se t p LLCI ULCI c_ps c_cs

-.43160 .07388 -5.84209 .00000 -.57724 -.28596 -.07727 -.38043

Direct effect of X on Y

Effect se t p LLCI ULCI c'_ps c'_cs

-.39257 .07506 -5.22998 .00000 -.54055 -.24460 -.07029 -.34603

Indirect effect(s) of X on Y:

Effect BootSE BootLLCI BootULCI

Manageab -.03903 .02127 -.08692 -.00393

Partially standardized indirect effect(s) of X on Y:

Effect BootSE BootLLCI BootULCI

Manageab -.00699 .00378 -.01543 -.00072

Completely standardized indirect effect(s) of X on Y:

Effect BootSE BootLLCI BootULCI

Manageab -.03440 .01868 -.07634 -.00348

*********************** ANALYSIS NOTES AND ERRORS ************************

Level of confidence for all confidence intervals in output:

95.0000

Number of bootstrap samples for percentile bootstrap confidence intervals:

5000

***************** PROCESS Procedure for SPSS Version 3.5 *****************

Written by Andrew F. Hayes, Ph.D. www.afhayes.com

Documentation available in Hayes (2018). www.guilford.com/p/hayes3

**************************************************************************

**Model : 4**

**Y : BCS = Breast Cancer Specific subscale**

**X : Stigma**

**M : Meaningfulness**

Covariates:

Age_cov Educatio Employme Social_p Insuranc Major_Ps Chemo_co Mastecto Radiothe

Sample

Size: 221

**************************************************************************

OUTCOME VARIABLE:

Meaningf

Model Summary

R R-sq MSE F df1 df2 p

.43435 .18866 20.31616 4.88319 10.00000 210.00000 .00000

Model

coeff se t p LLCI ULCI

constant 22.12763 2.67375 8.27588 .00000 16.85680 27.39846

Stigma_I -.23369 .06482 -3.60503 .00039 -.36147 -.10590

Age_cov .05889 .03442 1.71069 .08861 -.00897 .12674

Educatio -1.59914 .92285 -1.73283 .08459 -3.41839 .22010

Employme .60739 .97747 .62139 .53502 -1.31953 2.53430

Social_p .53412 .21518 2.48215 .01384 .10992 .95831

Insuranc .02574 1.08506 .02372 .98110 -2.11327 2.16475

Major_Ps -1.33952 .73036 -1.83404 .06806 -2.77930 .10026

Chemo_co -.26584 .45987 -.57808 .56383 -1.17240 .64071

Mastecto -.84146 .43347 -1.94122 .05357 -1.69596 .01305

Radiothe .61470 .78626 .78180 .43521 -.93528 2.16468

Standardized coefficients

coeff

Stigma_I -.23532

Age_cov .10993

Educatio -.12862

Employme .04649

Social_p .15739

Insuranc .00151

Major_Ps -.11725

Chemo_co -.04631

Mastecto -.13858

Radiothe .06169

| **Benjamini-Hochberg Procedure** | | | | |
| --- | --- | --- | --- | --- |
|  |  | **Ascending P-value s** | **I = ranking** | **(I/10)* 0.10** |
|  | **Stigma** | **.0003** | **1** | **.01** |
|  | **Social participation frequency** | **.0138** | **2** | **.02** |
|  | **Mastectomy** | **~~.053~~** | **3** | **.03** |
|  | **Major Psychological issues** | **~~.068~~** | **4** | **.04** |
|  | **Education** | **~~.084~~** | **5** | **.05** |
|  | **Age** | **~~.088~~** | **6** | **.06** |
|  | **Radiotherapy** | **~~.435~~** | **7** | **.07** |
|  | **Employment** | **~~.535~~** | **8** | **.08** |
|  | **Chemotherapy** | **~~.563~~** | **9** | **.09** |
|  | **Insurance** | **~~.981~~** | **10** | **.10** |

**************************************************************************

OUTCOME VARIABLE:

BCS

Model Summary

R R-sq MSE F df1 df2 p

.46378 .21509 25.77526 5.20658 11.00000 209.00000 .00000

Model

coeff se t p LLCI ULCI

constant 21.80520 3.46814 6.28729 .00000 14.96818 28.64222

Stigma_I -.38710 .07524 -5.14500 .00000 -.53543 -.23878

Meaningf .19042 .07773 2.44982 .01512 .03719 .34365

Age_cov .03433 .03904 .87942 .38018 -.04263 .11130

Educatio -1.27275 1.04688 -1.21575 .22545 -3.33654 .79105

Employme -1.16253 1.10200 -1.05492 .29268 -3.33500 1.00994

Social_p .28898 .24591 1.17517 .24126 -.19579 .77375

Insuranc 1.42939 1.22218 1.16954 .24352 -.97999 3.83878

Major_Ps -.71676 .82922 -.86438 .38837 -2.35147 .91794

Chemo_co -.63419 .51840 -1.22337 .22257 -1.65615 .38776

Mastecto -.01559 .49261 -.03165 .97478 -.98670 .95552

Radiothe -.18391 .88691 -.20736 .83593 -1.93234 1.56453

Standardized coefficients

coeff

Stigma_I -.34121

Meaningf .16668

Age_cov .05610

Educatio -.08961

Employme -.07789

Social_p .07454

Insuranc .07359

Major_Ps -.05492

Chemo_co -.09669

Mastecto -.00225

Radiothe -.01615

| **Benjamini-Hochberg Procedure** | | | | |
| --- | --- | --- | --- | --- |
|  |  | **Ascending P-value s** | **I = ranking** | **(I/11)* 0.10** |
|  | **Stigma** | **.00000** | **1** | **.009** |
|  | **Meaningfulness** | **.015** | **2** | **.018** |
|  | **Chemotherapy** | **~~.222~~** | **3** | **.027** |
|  | **Education** | **~~.225~~** | **4** | **.03636** |
|  | **Social participation frequency** | **~~.241~~** | **5** | **.045** |
|  | **Insurance** | **~~.243~~** | **6** | **.054** |
|  | **Employment** | **~~.292~~** | **7** | **.063** |
|  | **Age** | **~~.380~~** | **8** | **.072** |
|  | **Major Psychological issues** | **~~.388~~** | **9** | **.081** |
|  | **Radiotherapy** | **~~.835~~** | **10** | **.0909** |
|  | **Mastectomy** | **~~.974~~** | **11** | **.100** |

************************** TOTAL EFFECT MODEL ****************************

OUTCOME VARIABLE:

BCS

Model Summary

R R-sq MSE F df1 df2 p

.43881 .19255 26.38915 5.00781 10.00000 210.00000 .00000

Model

coeff se t p LLCI ULCI

constant 26.01867 3.04728 8.53832 .00000 20.01149 32.02586

Stigma_I -.43160 .07388 -5.84209 .00000 -.57724 -.28596

Age_cov .04555 .03923 1.16098 .24697 -.03179 .12288

Educatio -1.57725 1.05178 -1.49960 .13522 -3.65065 .49615

Employme -1.04688 1.11403 -.93972 .34844 -3.24299 1.14924

Social_p .39069 .24524 1.59304 .11265 -.09277 .87414

Insuranc 1.43430 1.23665 1.15982 .24744 -1.00354 3.87213

Major_Ps -.97183 .83240 -1.16751 .24433 -2.61275 .66910

Chemo_co -.68481 .52412 -1.30661 .19278 -1.71802 .34839

Mastecto -.17582 .49403 -.35589 .72228 -1.14970 .79807

Radiothe -.06686 .89611 -.07461 .94060 -1.83337 1.69966

Standardized coefficients

coeff

Stigma_I -.38043

Age_cov .07443

Educatio -.11104

Employme -.07014

Social_p .10077

Insuranc .07384

Major_Ps -.07446

Chemo_co -.10441

Mastecto -.02535

Radiothe -.00587

| **Benjamini-Hochberg Procedure** | | | | |
| --- | --- | --- | --- | --- |
|  |  | **Ascending P-value s** | **I = ranking** | **(I/10)* 0.10** |
|  | **Stigma** | **.00000** | **1** | **.01** |
|  | **Social participation frequency** | **~~.112~~** | **2** | **.02** |
|  | **Education** | **~~.135~~** | **3** | **.03** |
|  | **Chemotherapy** | **~~.192~~** | **4** | **.04** |
|  | **Major Psychological issues** | **~~.244~~** | **5** | **.05** |
|  | **Age** | **~~.246~~** | **6** | **.06** |
|  | **Insurance** | **~~.247~~** | **7** | **.07** |
|  | **Employment** | **~~.348~~** | **8** | **.08** |
|  | **Mastectomy** | **~~.722~~** | **9** | **.09** |
|  | **Radiotherapy** | **~~.940~~** | **10** | **.10** |

************** TOTAL, DIRECT, AND INDIRECT EFFECTS OF X ON Y **************

Total effect of X on Y

Effect se t p LLCI ULCI c_ps c_cs

-.43160 .07388 -5.84209 .00000 -.57724 -.28596 -.07727 -.38043

Direct effect of X on Y

Effect se t p LLCI ULCI c'_ps c'_cs

-.38710 .07524 -5.14500 .00000 -.53543 -.23878 -.06931 -.34121

Indirect effect(s) of X on Y:

Effect BootSE BootLLCI BootULCI

Meaningf -.04450 .02075 -.08907 -.00828

Partially standardized indirect effect(s) of X on Y:

Effect BootSE BootLLCI BootULCI

Meaningf -.00797 .00373 -.01612 -.00150

Completely standardized indirect effect(s) of X on Y:

Effect BootSE BootLLCI BootULCI

Meaningf -.03922 .01851 -.08030 -.00712

*********************** ANALYSIS NOTES AND ERRORS ************************

Level of confidence for all confidence intervals in output:

95.0000

Number of bootstrap samples for percentile bootstrap confidence intervals:

5000

***************** PROCESS Procedure for SPSS Version 3.5 *****************

Written by Andrew F. Hayes, Ph.D. www.afhayes.com

Documentation available in Hayes (2018). www.guilford.com/p/hayes3

**************************************************************************

**Model : 4**

**Y : BCS = Breast Cancer Specific subscale**

**X : Stigma**

**M : SOC = Sense of Coherence**

Covariates:

Age_cov Educatio Employme Social_p Insuranc Major_Ps Chemo_co Mastecto Radiothe

Sample

Size: 221

**************************************************************************

OUTCOME VARIABLE:

SOC

Model Summary

R R-sq MSE F df1 df2 p

.45548 .20746 151.84085 5.49712 10.00000 210.00000 .00000

Model

coeff se t p LLCI ULCI

constant 68.01609 7.30961 9.30502 .00000 53.60647 82.42570

Stigma_I -.82220 .17721 -4.63961 .00001 -1.17154 -.47285

Age_cov .16923 .09410 1.79834 .07356 -.01628 .35474

Educatio -.43019 2.52293 -.17051 .86477 -5.40371 4.54333

Employme 3.33713 2.67225 1.24881 .21313 -1.93075 8.60501

Social_p 1.41471 .58828 2.40485 .01705 .25503 2.57440

Insuranc -1.62359 2.96639 -.54733 .58473 -7.47131 4.22413

Major_Ps -4.96124 1.99669 -2.48473 .01375 -8.89738 -1.02511

Chemo_co -1.48699 1.25722 -1.18276 .23824 -3.96537 .99139

Mastecto -2.99414 1.18503 -2.52663 .01225 -5.33022 -.65805

Radiothe -.38276 2.14952 -.17807 .85884 -4.62015 3.85464

Standardized coefficients

coeff

Stigma_I -.29932

Age_cov .11422

Educatio -.01251

Employme .09234

Social_p .15071

Insuranc -.03452

Major_Ps -.15700

Chemo_co -.09364

Mastecto -.17827

Radiothe -.01389

| **Benjamini-Hochberg Procedure** | | | | |
| --- | --- | --- | --- | --- |
|  |  | **Ascending P-value s** | **I = ranking** | **(I/10)* 0.10** |
|  | **Stigma** | **.00001** | **1** | **.01** |
|  | **Social participation frequency** | **.017** | **2** | **.02** |
|  | **Mastectomy** | **.012** | **3** | **.03** |
|  | **Major Psychological issues** | **.013** | **4** | **.04** |
|  | **Age** | **~~.073~~** | **5** | **.05** |
|  | **Employment** | **~~.213~~** | **6** | **.06** |
|  | **Chemotherapy** | **~~.238~~** | **7** | **.07** |
|  | **Insurance** | **~~.584~~** | **8** | **.08** |
|  | **Radiotherapy** | **~~.858~~** | **9** | **.09** |
|  | **Education** | **~~.864~~** | **10** | **.10** |

**************************************************************************

OUTCOME VARIABLE:

BCS

Model Summary

R R-sq MSE F df1 df2 p

.48696 .23713 25.05160 5.90583 11.00000 209.00000 .00000

Model

coeff se t p LLCI ULCI

constant 19.35637 3.52843 5.48583 .00000 12.40049 26.31224

Stigma_I -.35107 .07558 -4.64492 .00001 -.50006 -.20207

SOC .09795 .02803 3.49461 .00058 .04270 .15321

Age_cov .02897 .03852 .75213 .45282 -.04696 .10490

Educatio -1.53511 1.02485 -1.49789 .13567 -3.55548 .48525

Employme -1.37375 1.08945 -1.26096 .20873 -3.52147 .77397

Social_p .25211 .24222 1.04085 .29915 -.22539 .72961

Insuranc 1.59333 1.20576 1.32143 .18780 -.78368 3.97034

Major_Ps -.48586 .82286 -.59046 .55552 -2.10804 1.13631

Chemo_co -.53916 .51236 -1.05231 .29387 -1.54922 .47090

Mastecto .11746 .48860 .24041 .81025 -.84576 1.08069

Radiothe -.02937 .87317 -.03363 .97320 -1.75071 1.69198

Standardized coefficients

coeff

Stigma_I -.30944

SOC .23716

Age_cov .04734

Educatio -.10808

Employme -.09204

Social_p .06503

Insuranc .08203

Major_Ps -.03723

Chemo_co -.08221

Mastecto .01693

Radiothe -.00258

| **Benjamini-Hochberg Procedure** | | | | |
| --- | --- | --- | --- | --- |
|  |  | **Ascending P-value s** | **I = ranking** | **(I/11)* 0.10** |
|  | **Stigma** | **.00000** | **1** | **.009** |
|  | **Sense of Coherence** | **.0005** | **2** | **.018** |
|  | **Education** | **~~.135~~** | **3** | **.027** |
|  | **Insurance** | **~~.187~~** | **4** | **.03636** |
|  | **Employment** | **~~.208~~** | **5** | **.045** |
|  | **Chemotherapy** | **~~.293~~** | **6** | **.054** |
|  | **Social participation frequency** | **~~.299~~** | **7** | **.063** |
|  | **Age** | **~~.452~~** | **8** | **.072** |
|  | **Major Psychological issues** | **~~.555~~** | **9** | **.081** |
|  | **Mastectomy** | **~~.810~~** | **10** | **.0909** |
|  | **Radiotherapy** | **~~.973~~** | **11** | **.100** |

************************** TOTAL EFFECT MODEL ****************************

OUTCOME VARIABLE:

BCS

Model Summary

R R-sq MSE F df1 df2 p

.43881 .19255 26.38915 5.00781 10.00000 210.00000 .00000

Model

coeff se t p LLCI ULCI

constant 26.01867 3.04728 8.53832 .00000 20.01149 32.02586

Stigma_I -.43160 .07388 -5.84209 .00000 -.57724 -.28596

Age_cov .04555 .03923 1.16098 .24697 -.03179 .12288

Educatio -1.57725 1.05178 -1.49960 .13522 -3.65065 .49615

Employme -1.04688 1.11403 -.93972 .34844 -3.24299 1.14924

Social_p .39069 .24524 1.59304 .11265 -.09277 .87414

Insuranc 1.43430 1.23665 1.15982 .24744 -1.00354 3.87213

Major_Ps -.97183 .83240 -1.16751 .24433 -2.61275 .66910

Chemo_co -.68481 .52412 -1.30661 .19278 -1.71802 .34839

Mastecto -.17582 .49403 -.35589 .72228 -1.14970 .79807

Radiothe -.06686 .89611 -.07461 .94060 -1.83337 1.69966

Standardized coefficients

coeff

Stigma_I -.38043

Age_cov .07443

Educatio -.11104

Employme -.07014

Social_p .10077

Insuranc .07384

Major_Ps -.07446

Chemo_co -.10441

Mastecto -.02535

Radiothe -.00587

| **Benjamini-Hochberg Procedure** | | | | |
| --- | --- | --- | --- | --- |
|  |  | **Ascending P-value s** | **I = ranking** | **(I/10)* 0.10** |
|  | **Stigma** | **.00000** | **1** | **.01** |
|  | **Social participation frequency** | **~~.112~~** | **2** | **.02** |
|  | **Education** | **~~.135~~** | **3** | **.03** |
|  | **Chemotherapy** | **~~.192~~** | **4** | **.04** |
|  | **Major Psychological issues** | **~~.244~~** | **5** | **.05** |
|  | **Age** | **~~.246~~** | **6** | **.06** |
|  | **Insurance** | **~~.247~~** | **7** | **.07** |
|  | **Employment** | **~~.348~~** | **8** | **.08** |
|  | **Mastectomy** | **~~.722~~** | **9** | **.09** |
|  | **Radiotherapy** | **~~.940~~** | **10** | **.10** |

************** TOTAL, DIRECT, AND INDIRECT EFFECTS OF X ON Y **************

Total effect of X on Y

Effect se t p LLCI ULCI c_ps c_cs

-.43160 .07388 -5.84209 .00000 -.57724 -.28596 -.07727 -.38043

Direct effect of X on Y

Effect se t p LLCI ULCI c'_ps c'_cs

-.35107 .07558 -4.64492 .00001 -.50006 -.20207 -.06285 -.30944

Indirect effect(s) of X on Y:

Effect BootSE BootLLCI BootULCI

SOC -.08054 .02836 -.14298 -.03126

Partially standardized indirect effect(s) of X on Y:

Effect BootSE BootLLCI BootULCI

SOC -.01442 .00504 -.02544 -.00563

Completely standardized indirect effect(s) of X on Y:

Effect BootSE BootLLCI BootULCI

SOC -.07099 .02482 -.12546 -.02737

*********************** ANALYSIS NOTES AND ERRORS ************************

Level of confidence for all confidence intervals in output:

95.0000

Number of bootstrap samples for percentile bootstrap confidence intervals:

5000

***************** PROCESS Procedure for SPSS Version 3.5 *****************

Written by Andrew F. Hayes, Ph.D. www.afhayes.com

Documentation available in Hayes (2018). www.guilford.com/p/hayes3

**************************************************************************

**Model : 4**

**Y : BCS**

**X : Stigma_I**

**M1 : Comprehensibility**

**M2 : Meaningfulness**

Covariates:

Age_cov Educatio Employme Social_p Insuranc Major_Ps Chemo_co Mastecto Radiothe

Sample

Size: 221

**************************************************************************

OUTCOME VARIABLE:

Comprehe

Model Summary

R R-sq MSE F df1 df2 p

.38011 .14448 36.53598 3.54648 10.00000 210.00000 .00023

Model

coeff se t p LLCI ULCI

constant 28.41039 3.58559 7.92350 .00000 21.34203 35.47875

Stigma_I -.32272 .08693 -3.71244 .00026 -.49408 -.15135

Age_cov .04775 .04616 1.03439 .30214 -.04325 .13875

Educatio -.31523 1.23758 -.25471 .79919 -2.75489 2.12444

Employme 1.82890 1.31082 1.39523 .16442 -.75515 4.41296

Social_p .36516 .28857 1.26541 .20713 -.20371 .93402

Insuranc -.55790 1.45510 -.38341 .70181 -3.42638 2.31059

Major_Ps -1.89152 .97944 -1.93123 .05480 -3.82232 .03927

Chemo_co -1.16488 .61670 -1.88888 .06029 -2.38060 .05085

Mastecto -1.47027 .58130 -2.52930 .01216 -2.61619 -.32435

Radiothe -1.08926 1.05440 -1.03306 .30277 -3.16783 .98931

Standardized coefficients

coeff

Stigma_I -.24884

Age_cov .06826

Educatio -.01941

Employme .10719

Social_p .08239

Insuranc -.02513

Major_Ps -.12678

Chemo_co -.15537

Mastecto -.18542

Radiothe -.08370

| **Benjamini-Hochberg Procedure** | | | | |
| --- | --- | --- | --- | --- |
|  |  | **Ascending P-value s** | **I = ranking** | **(I/10)* 0.10** |
|  | **Stigma** | **.0002** | **1** | **.01** |
|  | **Mastectomy** | **.0121** | **2** | **.02** |
|  | **Major Psychological issues** | **~~.054~~** | **3** | **.03** |
|  | **Chemotherapy** | **~~.060~~** | **4** | **.04** |
|  | **Employment** | **~~.164~~** | **5** | **.05** |
|  | **Social participation frequency** | **~~.207~~** | **6** | **.06** |
|  | **Age** | **~~.3021~~** | **7** | **.07** |
|  | **Radiotherapy** | **~~.3027~~** | **8** | **.08** |
|  | **Insurance** | **~~.701~~** | **9** | **.09** |
|  | **Education** | **~~.799~~** | **10** | **.10** |

**************************************************************************

OUTCOME VARIABLE:

Meaningf

Model Summary

R R-sq MSE F df1 df2 p

.43435 .18866 20.31616 4.88319 10.00000 210.00000 .00000

Model

coeff se t p LLCI ULCI

constant 22.12763 2.67375 8.27588 .00000 16.85680 27.39846

Stigma_I -.23369 .06482 -3.60503 .00039 -.36147 -.10590

Age_cov .05889 .03442 1.71069 .08861 -.00897 .12674

Educatio -1.59914 .92285 -1.73283 .08459 -3.41839 .22010

Employme .60739 .97747 .62139 .53502 -1.31953 2.53430

Social_p .53412 .21518 2.48215 .01384 .10992 .95831

Insuranc .02574 1.08506 .02372 .98110 -2.11327 2.16475

Major_Ps -1.33952 .73036 -1.83404 .06806 -2.77930 .10026

Chemo_co -.26584 .45987 -.57808 .56383 -1.17240 .64071

Mastecto -.84146 .43347 -1.94122 .05357 -1.69596 .01305

Radiothe .61470 .78626 .78180 .43521 -.93528 2.16468

Standardized coefficients

coeff

Stigma_I -.23532

Age_cov .10993

Educatio -.12862

Employme .04649

Social_p .15739

Insuranc .00151

Major_Ps -.11725

Chemo_co -.04631

Mastecto -.13858

Radiothe .06169

| **Benjamini-Hochberg Procedure** | | | | |
| --- | --- | --- | --- | --- |
|  |  | **Ascending P-value s** | **I = ranking** | **(I/10)* 0.10** |
|  | **Stigma** | **.0003** | **1** | **.01** |
|  | **Social participation frequency** | **.0138** | **2** | **.02** |
|  | **Mastectomy** | **~~.053~~** | **3** | **.03** |
|  | **Major Psychological issues** | **~~.068~~** | **4** | **.04** |
|  | **Education** | **~~.084~~** | **5** | **.05** |
|  | **Age** | **~~.088~~** | **6** | **.06** |
|  | **Radiotherapy** | **~~.435~~** | **7** | **.07** |
|  | **Employment** | **~~.535~~** | **8** | **.08** |
|  | **Chemotherapy** | **~~.563~~** | **9** | **.09** |
|  | **Insurance** | **~~.981~~** | **10** | **.10** |

**************************************************************************

OUTCOME VARIABLE:

BCS

Model Summary

R R-sq MSE F df1 df2 p

.48933 .23945 25.09551 5.45706 12.00000 208.00000 .00000

Model

coeff se t p LLCI ULCI

constant 18.63043 3.63647 5.12321 .00000 11.46135 25.79950

Stigma_I -.35011 .07561 -4.63043 .00001 -.49917 -.20105

Comprehe .15273 .05918 2.58090 .01054 .03607 .26940

Meaningf .13780 .07936 1.73637 .08398 -.01865 .29425

Age_cov .03014 .03856 .78168 .43529 -.04587 .10615

Educatio -1.30875 1.03308 -1.26685 .20663 -3.34539 .72789

Employme -1.40990 1.09159 -1.29160 .19793 -3.56191 .74210

Social_p .26132 .24288 1.07591 .28321 -.21750 .74013

Insuranc 1.51596 1.20642 1.25657 .21032 -.86243 3.89434

Major_Ps -.49835 .82258 -.60584 .54528 -2.12001 1.12330

Chemo_co -.47027 .51544 -.91236 .36264 -1.48643 .54589

Mastecto .16469 .49106 .33537 .73768 -.80341 1.13278

Radiothe .01480 .87852 .01685 .98657 -1.71714 1.74674

Standardized coefficients

coeff

Stigma_I -.30860

Comprehe .17459

Meaningf .12062

Age_cov .04925

Educatio -.09214

Employme -.09446

Social_p .06740

Insuranc .07804

Major_Ps -.03818

Chemo_co -.07170

Mastecto .02374

Radiothe .00130

| **Benjamini-Hochberg Procedure** | | | | |
| --- | --- | --- | --- | --- |
|  |  | **Ascending P-value s** | **I = ranking** | **(I/12)* 0.10** |
|  | **Stigma** | **.00000** | **1** | **.0083** |
|  | **Comprehensibility** | **.0105** | **2** | **.016** |
|  | **Meaningfulness** | **~~.083~~** | **3** | **.025** |
|  | **Employment** | **~~.197~~** | **4** | **.033** |
|  | **Education** | **~~.206~~** | **5** | **.0416** |
|  | **Insurance** | **~~.210~~** | **6** | **.050** |
|  | **Social participation frequency** | **~~.283~~** | **7** | **.0583** |
|  | **Chemotherapy** | **~~.362~~** | **8** | **.066** |
|  | **Age** | **~~.435~~** | **9** | **.075** |
|  | **Major Psychological issues** | **~~.545~~** | **10** | **.083** |
|  | **Mastectomy** | **~~.737~~** | **11** | **.091** |
|  | **Radiotherapy** | **~~.986~~** | **12** | **.10** |

************************** TOTAL EFFECT MODEL ****************************

OUTCOME VARIABLE:

BCS

Model Summary

R R-sq MSE F df1 df2 p

.43881 .19255 26.38915 5.00781 10.00000 210.00000 .00000

Model

coeff se t p LLCI ULCI

constant 26.01867 3.04728 8.53832 .00000 20.01149 32.02586

Stigma_I -.43160 .07388 -5.84209 .00000 -.57724 -.28596

Age_cov .04555 .03923 1.16098 .24697 -.03179 .12288

Educatio -1.57725 1.05178 -1.49960 .13522 -3.65065 .49615

Employme -1.04688 1.11403 -.93972 .34844 -3.24299 1.14924

Social_p .39069 .24524 1.59304 .11265 -.09277 .87414

Insuranc 1.43430 1.23665 1.15982 .24744 -1.00354 3.87213

Major_Ps -.97183 .83240 -1.16751 .24433 -2.61275 .66910

Chemo_co -.68481 .52412 -1.30661 .19278 -1.71802 .34839

Mastecto -.17582 .49403 -.35589 .72228 -1.14970 .79807

Radiothe -.06686 .89611 -.07461 .94060 -1.83337 1.69966

Standardized coefficients

coeff

Stigma_I -.38043

Age_cov .07443

Educatio -.11104

Employme -.07014

Social_p .10077

Insuranc .07384

Major_Ps -.07446

Chemo_co -.10441

Mastecto -.02535

Radiothe -.00587

| **Benjamini-Hochberg Procedure** | | | | |
| --- | --- | --- | --- | --- |
|  |  | **Ascending P-value s** | **I = ranking** | **(I/10)* 0.10** |
|  | **Stigma** | **.00000** | **1** | **.01** |
|  | **Social participation frequency** | **~~.112~~** | **2** | **.02** |
|  | **Education** | **~~.135~~** | **3** | **.03** |
|  | **Chemotherapy** | **~~.192~~** | **4** | **.04** |
|  | **Major Psychological issues** | **~~.244~~** | **5** | **.05** |
|  | **Age** | **~~.246~~** | **6** | **.06** |
|  | **Insurance** | **~~.247~~** | **7** | **.07** |
|  | **Employment** | **~~.348~~** | **8** | **.08** |
|  | **Mastectomy** | **~~.722~~** | **9** | **.09** |
|  | **Radiotherapy** | **~~.940~~** | **10** | **.10** |

************** TOTAL, DIRECT, AND INDIRECT EFFECTS OF X ON Y **************

Total effect of X on Y

Effect se t p LLCI ULCI c_ps c_cs

-.43160 .07388 -5.84209 .00000 -.57724 -.28596 -.07727 -.38043

Direct effect of X on Y

Effect se t p LLCI ULCI c'_ps c'_cs

-.35011 .07561 -4.63043 .00001 -.49917 -.20105 -.06268 -.30860

Indirect effect(s) of X on Y:

Effect BootSE BootLLCI BootULCI

TOTAL -.08149 .02829 -.14407 -.03320

Comprehe -.04929 .02338 -.10194 -.01040

Meaningf -.03220 .02016 -.07668 .00463

Partially standardized indirect effect(s) of X on Y:

Effect BootSE BootLLCI BootULCI

TOTAL -.01459 .00505 -.02569 -.00605

Comprehe -.00882 .00418 -.01813 -.00186

Meaningf -.00577 .00363 -.01370 .00081

Completely standardized indirect effect(s) of X on Y:

Effect BootSE BootLLCI BootULCI

TOTAL -.07183 .02462 -.12459 -.02991

Comprehe -.04345 .02015 -.08686 -.00922

Meaningf -.02838 .01797 -.06813 .00410

*********************** ANALYSIS NOTES AND ERRORS ************************

Level of confidence for all confidence intervals in output:

95.0000

Number of bootstrap samples for percentile bootstrap confidence intervals:

5000

***************** PROCESS Procedure for SPSS Version 3.5 *****************

Written by Andrew F. Hayes, Ph.D. www.afhayes.com

Documentation available in Hayes (2018). www.guilford.com/p/hayes3

**************************************************************************

**Model : 4**

**Y : Arm subscale**

**X : Stigma**

**M : Manageability**

Covariates:

Age_cov Educatio Employme Social_p Insuranc Major_Ps Chemo_co Mastecto Radiothe

Sample

Size: 136

**************************************************************************

OUTCOME VARIABLE:

Manageab

Model Summary

R R-sq MSE F df1 df2 p

.43694 .19091 28.78249 2.94952 10.00000 125.00000 .00233

Model

coeff se t p LLCI ULCI

constant 25.67631 4.03706 6.36016 .00000 17.68646 33.66615

Stigma_I -.28030 .09195 -3.04842 .00281 -.46228 -.09832

Age_cov .01067 .05324 .20038 .84151 -.09469 .11603

Educatio -.22900 1.41672 -.16164 .87185 -3.03286 2.57486

Employme .01304 1.60324 .00813 .99352 -3.15997 3.18604

Social_p .38654 .29030 1.33150 .18545 -.18801 .96108

Insuranc -2.77293 1.54515 -1.79460 .07513 -5.83097 .28512

Major_Ps -2.79103 1.13434 -2.46049 .01524 -5.03603 -.54603

Chemo_co -.26704 1.03338 -.25841 .79652 -2.31223 1.77816

Mastecto -1.02723 .73596 -1.39576 .16526 -2.48379 .42934

Radiothe -.69189 1.06871 -.64741 .51855 -2.80701 1.42322

Standardized coefficients

coeff

Stigma_I -.26195

Age_cov .01685

Educatio -.01527

Employme .00075

Social_p .11109

Insuranc -.15191

Major_Ps -.20924

Chemo_co -.02323

Mastecto -.11873

Radiothe -.05970

| **Benjamini-Hochberg Procedure** | | | | |
| --- | --- | --- | --- | --- |
|  |  | **Ascending P-value s** | **I = ranking** | **(I/10)* 0.10** |
|  | **Stigma** | **.0028** | **1** | **.001** |
|  | **Major Psychological issues** | **.015** | **2** | **.02** |
|  | **Insurance** | **~~.075~~** | **3** | **.03** |
|  | **Mastectomy** | **~~.165~~** | **4** | **.04** |
|  | **Social participation frequency** | **~~.185~~** | **5** | **.05** |
|  | **Radiotherapy** | **~~.518~~** | **6** | **.06** |
|  | **Chemotherapy** | **~~.796~~** | **7** | **.07** |
|  | **Age** | **~~.841~~** | **8** | **.08** |
|  | **Education** | **~~.871~~** | **9** | **.09** |
|  | **Employment** | **~~.993~~** | **10** | **.10** |

**************************************************************************

OUTCOME VARIABLE:

Arm subscale

Model Summary

R R-sq MSE F df1 df2 p

.51911 .26948 .06094 4.15834 11.00000 124.00000 .00003

Model

coeff se t p LLCI ULCI

constant .45722 .21372 2.13932 .03437 .03420 .88023

Stigma_I -.00537 .00439 -1.22382 .22334 -.01405 .00331

Manageab .00913 .00412 2.21758 .02841 .00098 .01727

Age_cov -.00249 .00245 -1.01792 .31070 -.00734 .00236

Educatio -.09536 .06520 -1.46266 .14609 -.22441 .03368

Employme -.03943 .07377 -.53448 .59397 -.18545 .10659

Social_p -.00454 .01345 -.33766 .73619 -.03117 .02208

Insuranc .08979 .07201 1.24686 .21480 -.05274 .23232

Major_Ps .03060 .05345 .57249 .56802 -.07519 .13638

Chemo_co .00387 .04756 .08144 .93523 -.09027 .09802

Mastecto -.10820 .03413 -3.17050 .00192 -.17575 -.04065

Radiothe .12255 .04926 2.48792 .01418 .02506 .22005

Standardized coefficients

coeff

Stigma_I -.10399

Manageab .18923

Age_cov -.08166

Educatio -.13181

Employme -.04728

Social_p -.02707

Insuranc .10198

Major_Ps .04756

Chemo_co .00698

Mastecto -.25929

Radiothe .21925

| **Benjamini-Hochberg Procedure** | | | | |
| --- | --- | --- | --- | --- |
|  |  | **Ascending P-value s** | **I = ranking** | **(I/11)* 0.10** |
|  | **Mastectomy** | **.001** | **1** | **.009** |
|  | **Radiotherapy** | **.014** | **2** | **.018** |
|  | **Manageability** | **~~.028~~** | **3** | **.027** |
|  | **Education** | **~~.146~~** | **4** | **.03636** |
|  | **Insurance** | **~~.214~~** | **5** | **.045** |
|  | **Stigma** | **~~.223~~** | **6** | **.054** |
|  | **Age** | **~~.310~~** | **7** | **.063** |
|  | **Major Psychological issues** | **~~.568~~** | **8** | **.072** |
|  | **Employment** | **~~.593~~** | **9** | **.081** |
|  | **Social participation frequency** | **~~.736~~** | **10** | **.0909** |
|  | **Chemotherapy** | **~~.935~~** | **11** | **.100** |

************************** TOTAL EFFECT MODEL ****************************

OUTCOME VARIABLE:

Arm subscale

Model Summary

R R-sq MSE F df1 df2 p

.49042 .24051 .06285 3.95835 10.00000 125.00000 .00010

Model

coeff se t p LLCI ULCI

constant .69157 .18866 3.66576 .00036 .31819 1.06494

Stigma_I -.00793 .00430 -1.84445 .06748 -.01643 .00058

Age_cov -.00240 .00249 -.96336 .33723 -.00732 .00253

Educatio -.09745 .06620 -1.47198 .14354 -.22848 .03358

Employme -.03931 .07492 -.52471 .60072 -.18759 .10897

Social_p -.00101 .01357 -.07478 .94051 -.02786 .02583

Insuranc .06448 .07221 .89299 .37358 -.07843 .20738

Major_Ps .00512 .05301 .09666 .92315 -.09979 .11003

Chemo_co .00144 .04829 .02974 .97632 -.09414 .09701

Mastecto -.11758 .03439 -3.41879 .00085 -.18565 -.04951

Radiothe .11624 .04994 2.32748 .02155 .01740 .21508

Standardized coefficients

coeff

Stigma_I -.15356

Age_cov -.07847

Educatio -.13470

Employme -.04714

Social_p -.00604

Insuranc .07324

Major_Ps .00796

Chemo_co .00259

Mastecto -.28176

Radiothe .20796

| **Benjamini-Hochberg Procedure** | | | | |
| --- | --- | --- | --- | --- |
|  |  | **Ascending P-value s** | **I = ranking** | **(I/10)* 0.10** |
|  | **Mastectomy** | **.000** | **1** | **.01** |
|  | **Radiotherapy** | **~~.021~~** | **2** | **.02** |
|  | **Stigma** | **~~.067~~** | **3** | **.03** |
|  | **Education** | **~~.143~~** | **4** | **.04** |
|  | **Age** | **~~.337~~** | **5** | **.05** |
|  | **Insurance** | **~~.373~~** | **6** | **.06** |
|  | **Employment** | **~~.600~~** | **7** | **.07** |
|  | **Major Psychological issues** | **~~.923~~** | **8** | **.08** |
|  | **Social participation frequency** | **~~.940~~** | **9** | **.09** |
|  | **Chemotherapy** | **~~.976~~** | **10** | **.10** |

************** TOTAL, DIRECT, AND INDIRECT EFFECTS OF X ON Y **************

Total effect of X on Y

Effect se t p LLCI ULCI c_ps c_cs

-.00793 .00430 -1.84445 .06748 -.01643 .00058 -.02863 -.15356

Direct effect of X on Y

Effect se t p LLCI ULCI c'_ps c'_cs

-.00537 .00439 -1.22382 .22334 -.01405 .00331 -.01939 -.10399

Indirect effect(s) of X on Y:

Effect BootSE BootLLCI BootULCI

Manageab -.00256 .00146 -.00599 -.00031

Partially standardized indirect effect(s) of X on Y:

Effect BootSE BootLLCI BootULCI

Manageab -.00924 .00531 -.02159 -.00112

Completely standardized indirect effect(s) of X on Y:

Effect BootSE BootLLCI BootULCI

Manageab -.04957 .02858 -.11666 -.00609

*********************** ANALYSIS NOTES AND ERRORS ************************

Level of confidence for all confidence intervals in output:

95.0000

Number of bootstrap samples for percentile bootstrap confidence intervals:

5000

***************** PROCESS Procedure for SPSS Version 3.5 *****************

Written by Andrew F. Hayes, Ph.D. www.afhayes.com

Documentation available in Hayes (2018). www.guilford.com/p/hayes3

**************************************************************************

**Model : 4**

**Y : FACT_G**

**X : Stigma_I**

**M : Manageability**

Covariates:

Age_cov Educatio Employme Social_p Insuranc Major_Ps Chemo_co Mastecto Radiothe

Sample

Size: 221

**************************************************************************

OUTCOME VARIABLE:

Manageab

Model Summary

R R-sq MSE F df1 df2 p

.35462 .12576 30.37485 3.02083 10.00000 210.00000 .00137

Model

coeff se t p LLCI ULCI

constant 17.47806 3.26932 5.34609 .00000 11.03318 23.92295

Stigma_I -.26580 .07926 -3.35345 .00095 -.42205 -.10955

Age_cov .06260 .04209 1.48725 .13845 -.02037 .14557

Educatio 1.48418 1.12841 1.31528 .18985 -.74029 3.70865

Employme .90084 1.19520 .75372 .45186 -1.45528 3.25697

Social_p .51544 .26311 1.95900 .05144 -.00324 1.03413

Insuranc -1.09144 1.32676 -.82264 .41165 -3.70690 1.52403

Major_Ps -1.73020 .89305 -1.93742 .05404 -3.49069 .03028

Chemo_co -.05627 .56231 -.10007 .92038 -1.16476 1.05222

Mastecto -.68241 .53002 -1.28751 .19933 -1.72725 .36244

Radiothe .09180 .96140 .09548 .92402 -1.80343 1.98703

Standardized coefficients

coeff

Stigma_I -.22723

Age_cov .09921

Educatio .10134

Employme .05853

Social_p .12894

Insuranc -.05450

Major_Ps -.12857

Chemo_co -.00832

Mastecto -.09541

Radiothe .00782

| **Benjamini-Hochberg Procedure** | | | | |
| --- | --- | --- | --- | --- |
|  |  | **Ascending P-value s** | **I = ranking** | **(I/10)* 0.10** |
|  | **Stigma** | **.0009** | **1** | **.001** |
|  | **Social participation frequency** | **~~.051~~** | **2** | **.02** |
|  | **Major Psychological issues** | **~~.054~~** | **3** | **.03** |
|  | **Age** | **~~.138~~** | **4** | **.04** |
|  | **Education** | **~~.189~~** | **5** | **.05** |
|  | **Mastectomy** | **~~.199~~** | **6** | **.06** |
|  | **Insurance** | **~~.411~~** | **7** | **.07** |
|  | **Employment** | **~~.451~~** | **8** | **.08** |
|  | **Chemotherapy** | **~~.920~~** | **9** | **.09** |
|  | **Radiotherapy** | **~~.924~~** | **10** | **.10** |

**************************************************************************

OUTCOME VARIABLE:

FACT_G

Model Summary

R R-sq MSE F df1 df2 p

.55777 .31111 195.41015 8.58061 11.00000 209.00000 .00000

Model

coeff se t p LLCI ULCI

constant 90.72987 8.83856 10.26524 .00000 73.30571 108.15403

Stigma_I -1.39890 .20635 -6.77928 .00000 -1.80569 -.99211

Manageab .41557 .17503 2.37430 .01849 .07052 .76061

Age_cov -.13217 .10732 -1.23163 .21947 -.34373 .07939

Educatio -4.69054 2.87387 -1.63214 .10416 -10.35603 .97494

Employme -3.68844 3.03559 -1.21506 .22571 -9.67274 2.29587

Social_p 1.28776 .67343 1.91224 .05721 -.03983 2.61535

Insuranc 3.88963 3.37059 1.15399 .24982 -2.75508 10.53435

Major_Ps -4.77872 2.28527 -2.09110 .03773 -9.28386 -.27358

Chemo_co -1.76836 1.42626 -1.23985 .21642 -4.58006 1.04335

Mastecto -1.14419 1.34964 -.84777 .39753 -3.80483 1.51646

Radiothe -.35760 2.43854 -.14665 .88355 -5.16488 4.44968

Standardized coefficients

coeff

Stigma_I -.41954

Manageab .14579

Age_cov -.07349

Educatio -.11236

Employme -.08408

Social_p .11301

Insuranc .06813

Major_Ps -.12458

Chemo_co -.09174

Mastecto -.05612

Radiothe -.01069

| **Benjamini-Hochberg Procedure** | | | | |
| --- | --- | --- | --- | --- |
|  |  | **Ascending P-value s** | **I = ranking** | **(I/11)* 0.10** |
|  | **Stigma** | **.00000** | **1** | **.009** |
|  | **Manageability** | **~~.01849~~** | **2** | **.01818** |
|  | **Major Psychological issues** | **~~.037~~** | **3** | **.027** |
|  | **Social participation frequency** | **~~.057~~** | **4** | **.03636** |
|  | **Education** | **~~.104~~** | **5** | **.045** |
|  | **Chemotherapy** | **~~.216~~** | **6** | **.054** |
|  | **Age** | **~~.219~~** | **7** | **.063** |
|  | **Employment** | **~~.225~~** | **8** | **.072** |
|  | **Insurance** | **~~.249~~** | **9** | **.081** |
|  | **Mastectomy** | **~~.379~~** | **10** | **.0909** |
|  | **Radiotherapy** | **~~.883~~** | **11** | **.100** |

************************** TOTAL EFFECT MODEL ****************************

OUTCOME VARIABLE:

FACT_G

Model Summary

R R-sq MSE F df1 df2 p

.54086 .29253 199.72527 8.68320 10.00000 210.00000 .00000

Model

coeff se t p LLCI ULCI

constant 97.99319 8.38332 11.68906 .00000 81.46693 114.51946

Stigma_I -1.50936 .20324 -7.42632 .00000 -1.91002 -1.10870

Age_cov -.10616 .10793 -.98363 .32643 -.31892 .10660

Educatio -4.07377 2.89353 -1.40789 .16064 -9.77786 1.63033

Employme -3.31407 3.06478 -1.08134 .28079 -9.35576 2.72761

Social_p 1.50196 .67469 2.22616 .02707 .17193 2.83200

Insuranc 3.43607 3.40212 1.00998 .31367 -3.27063 10.14276

Major_Ps -5.49774 2.28999 -2.40077 .01723 -10.01205 -.98342

Chemo_co -1.79174 1.44189 -1.24263 .21539 -4.63418 1.05069

Mastecto -1.42777 1.35910 -1.05052 .29468 -4.10701 1.25146

Radiothe -.31945 2.46526 -.12958 .89702 -5.17928 4.54038

Standardized coefficients

coeff

Stigma_I -.45267

Age_cov -.05903

Educatio -.09758

Employme -.07554

Social_p .13181

Insuranc .06019

Major_Ps -.14332

Chemo_co -.09295

Mastecto -.07003

Radiothe -.00955

| **Benjamini-Hochberg Procedure** | | | | |
| --- | --- | --- | --- | --- |
|  |  | **Ascending P-value s** | **I = ranking** | **(I/10)* 0.10** |
|  | **Stigma** | **.00000** | **1** | **.01** |
|  | **Major Psychological issues** | **.017** | **2** | **.02** |
|  | **Social participation frequency** | **.027** | **3** | **.03** |
|  | **Education** | **~~.160~~** | **4** | **.04** |
|  | **Chemotherapy** | **~~.215~~** | **5** | **.05** |
|  | **Mastectomy** | **~~.294~~** | **6** | **.06** |
|  | **Employment** | **~~.280~~** | **7** | **.07** |
|  | **Insurance** | **~~.313~~** | **8** | **.08** |
|  | **Age** | **~~.326~~** | **9** | **.09** |
|  | **Radiotherapy** | **~~.897~~** | **10** | **.10** |

************** TOTAL, DIRECT, AND INDIRECT EFFECTS OF X ON Y **************

Total effect of X on Y

Effect se t p LLCI ULCI c_ps c_cs

-1.50936 .20324 -7.42632 .00000 -1.91002 -1.10870 -.09195 -.45267

Direct effect of X on Y

Effect se t p LLCI ULCI c'_ps c'_cs

-1.39890 .20635 -6.77928 .00000 -1.80569 -.99211 -.08522 -.41954

Indirect effect(s) of X on Y:

Effect BootSE BootLLCI BootULCI

Manageab -.11046 .05201 -.22344 -.01918

Partially standardized indirect effect(s) of X on Y:

Effect BootSE BootLLCI BootULCI

Manageab -.00673 .00316 -.01363 -.00120

Completely standardized indirect effect(s) of X on Y:

Effect BootSE BootLLCI BootULCI

Manageab -.03313 .01558 -.06654 -.00584

*********************** ANALYSIS NOTES AND ERRORS ************************

Level of confidence for all confidence intervals in output:

95.0000

Number of bootstrap samples for percentile bootstrap confidence intervals:

5000

***************** PROCESS Procedure for SPSS Version 3.5 *****************

Written by Andrew F. Hayes, Ph.D. www.afhayes.com

Documentation available in Hayes (2018). www.guilford.com/p/hayes3

**************************************************************************

**Model : 4**

**Y : FACT_G**

**X : Stigma_I**

**M : Meaningfulness**

Covariates:

Age_cov Educatio Employme Social_p Insuranc Major_Ps Chemo_co Mastecto Radiothe

Sample

Size: 221

**************************************************************************

OUTCOME VARIABLE:

Meaningf

Model Summary

R R-sq MSE F(HC4) df1 df2 p

.43435 .18866 20.31616 8.05231 10.00000 210.00000 .00000

Model

coeff se(HC4) t p LLCI ULCI

constant 22.12763 2.53165 8.74041 .00000 17.13693 27.11833

Stigma_I -.23369 .06320 -3.69777 .00028 -.35827 -.10910

Age_cov .05889 .03292 1.78882 .07509 -.00601 .12378

Educatio -1.59914 1.01029 -1.58285 .11496 -3.59076 .39248

Employme .60739 1.21085 .50162 .61646 -1.77959 2.99436

Social_p .53412 .26313 2.02983 .04364 .01539 1.05284

Insuranc .02574 1.05951 .02429 .98064 -2.06290 2.11438

Major_Ps -1.33952 .72667 -1.84337 .06668 -2.77201 .09298

Chemo_co -.26584 .43980 -.60446 .54619 -1.13283 .60115

Mastecto -.84146 .43800 -1.92113 .05607 -1.70490 .02198

Radiothe .61470 .76913 .79922 .42506 -.90150 2.13090

Standardized coefficients

coeff

Stigma_I -.23532

Age_cov .10993

Educatio -.12862

Employme .04649

Social_p .15739

Insuranc .00151

Major_Ps -.11725

Chemo_co -.04631

Mastecto -.13858

Radiothe .06169

| **Benjamini-Hochberg Procedure** | | | | |
| --- | --- | --- | --- | --- |
|  |  | **Ascending P-value s** | **I = ranking** | **(I/10)* 0.10** |
|  | **Stigma** | **.0002** | **1** | **.01** |
|  | **Social participation frequency** | **~~.043~~** | **2** | **.02** |
|  | **Mastectomy** | **~~.056~~** | **3** | **.03** |
|  | **Major Psychological issues** | **~~.066~~** | **4** | **.04** |
|  | **Age** | **~~.075~~** | **5** | **.05** |
|  | **Education** | **~~.114~~** | **6** | **.06** |
|  | **Radiotherapy** | **~~.425~~** | **7** | **.07** |
|  | **Chemotherapy** | **~~.546~~** | **8** | **.08** |
|  | **Employment** | **~~.616~~** | **9** | **.09** |
|  | **Insurance** | **~~.980~~** | **10** | **.10** |

**************************************************************************

OUTCOME VARIABLE:

FACT_G

Model Summary

R R-sq MSE F(HC4) df1 df2 p

.60855 .37034 178.60988 10.15929 11.00000 209.00000 .00000

Model

coeff se(HC4) t p LLCI ULCI

constant 74.98467 9.24982 8.10661 .00000 56.74975 93.21959

Stigma_I -1.26637 .23152 -5.46976 .00000 -1.72278 -.80995

Meaningf 1.03981 .21706 4.79048 .00000 .61191 1.46771

Age_cov -.16739 .10135 -1.65152 .10013 -.36720 .03242

Educatio -2.41096 2.36710 -1.01853 .30960 -7.07741 2.25549

Employme -3.94564 2.76489 -1.42705 .15506 -9.39629 1.50501

Social_p .94658 .94144 1.00546 .31584 -.90935 2.80252

Insuranc 3.40930 3.11509 1.09445 .27502 -2.73173 9.55034

Major_Ps -4.10490 2.57267 -1.59558 .11209 -9.17662 .96682

Chemo_co -1.51532 1.40277 -1.08023 .28128 -4.28071 1.25008

Mastecto -.55282 1.49045 -.37091 .71108 -3.49106 2.38542

Radiothe -.95863 2.48612 -.38559 .70019 -5.85971 3.94245

Standardized coefficients

coeff

Stigma_I -.37979

Meaningf .30968

Age_cov -.09307

Educatio -.05775

Employme -.08994

Social_p .08307

Insuranc .05972

Major_Ps -.10701

Chemo_co -.07861

Mastecto -.02712

Radiothe -.02865

| **Benjamini-Hochberg Procedure** | | | | |
| --- | --- | --- | --- | --- |
|  |  | **Ascending P-value s** | **I = ranking** | **(I/11)* 0.10** |
|  | **Stigma** | **.00000** | **1** | **.009** |
|  | **Meaningfulness** | **.00000** | **2** | **.01818** |
|  | **Age** | **~~.100~~** | **3** | **.027** |
|  | **Major Psychological issues** | **~~.112~~** | **4** | **.03636** |
|  | **Employment** | **~~.155~~** | **5** | **.045** |
|  | **Insurance** | **~~.275~~** | **6** | **.054** |
|  | **Chemotherapy** | **~~.281~~** | **7** | **.063** |
|  | **Education** | **~~.309~~** | **8** | **.072** |
|  | **Social participation frequency** | **~~.315~~** | **9** | **.081** |
|  | **Radiotherapy** | **~~.700~~** | **10** | **.0909** |
|  | **Mastectomy** | **~~.711~~** | **11** | **.100** |

************************** TOTAL EFFECT MODEL ****************************

OUTCOME VARIABLE:

FACT_G

Model Summary

R R-sq MSE F(HC4) df1 df2 p

.54086 .29253 199.72527 8.08476 10.00000 210.00000 .00000

Model

coeff se(HC4) t p LLCI ULCI

constant 97.99319 8.46450 11.57696 .00000 81.30691 114.67948

Stigma_I -1.50936 .23571 -6.40348 .00000 -1.97401 -1.04470

Age_cov -.10616 .10653 -.99649 .32016 -.31617 .10385

Educatio -4.07377 2.63209 -1.54773 .12319 -9.26248 1.11495

Employme -3.31407 3.03693 -1.09126 .27641 -9.30086 2.67271

Social_p 1.50196 .78438 1.91484 .05687 -.04430 3.04823

Insuranc 3.43607 3.55197 .96737 .33447 -3.56602 10.43815

Major_Ps -5.49774 2.70660 -2.03124 .04349 -10.83332 -.16216

Chemo_co -1.79174 1.46240 -1.22521 .22187 -4.67460 1.09112

Mastecto -1.42777 1.48589 -.96089 .33771 -4.35695 1.50141

Radiothe -.31945 2.66992 -.11965 .90488 -5.58274 4.94384

Standardized coefficients

coeff

Stigma_I -.45267

Age_cov -.05903

Educatio -.09758

Employme -.07554

Social_p .13181

Insuranc .06019

Major_Ps -.14332

Chemo_co -.09295

Mastecto -.07003

Radiothe -.00955

| **Benjamini-Hochberg Procedure** | | | | |
| --- | --- | --- | --- | --- |
|  |  | **Ascending P-value s** | **I = ranking** | **(I/10)* 0.10** |
|  | **Stigma** | **.00000** | **1** | **.01** |
|  | **Major Psychological issues** | **~~.043~~** | **2** | **.02** |
|  | **Social participation frequency** | **~~.056~~** | **3** | **.03** |
|  | **Education** | **~~.123~~** | **4** | **.04** |
|  | **Chemotherapy** | **~~.221~~** | **5** | **.05** |
|  | **Employment** | **~~.276~~** | **6** | **.06** |
|  | **Age** | **~~.320~~** | **7** | **.07** |
|  | **Insurance** | **~~.334~~** | **8** | **.08** |
|  | **Mastectomy** | **~~.337~~** | **9** | **.09** |
|  | **Radiotherapy** | **~~.904~~** | **10** | **.10** |

************** TOTAL, DIRECT, AND INDIRECT EFFECTS OF X ON Y **************

Total effect of X on Y

Effect se(HC4) t p LLCI ULCI c_ps c_cs

-1.50936 .23571 -6.40348 .00000 -1.97401 -1.04470 -.09195 -.45267

Direct effect of X on Y

Effect se(HC4) t p LLCI ULCI c'_ps c'_cs

-1.26637 .23152 -5.46976 .00000 -1.72278 -.80995 -.07714 -.37979

Indirect effect(s) of X on Y:

Effect BootSE BootLLCI BootULCI

Meaningf -.24299 .07831 -.40882 -.10225

Partially standardized indirect effect(s) of X on Y:

Effect BootSE BootLLCI BootULCI

Meaningf -.01480 .00467 -.02465 -.00642

Completely standardized indirect effect(s) of X on Y:

Effect BootSE BootLLCI BootULCI

Meaningf -.07287 .02335 -.12253 -.03076

*********************** ANALYSIS NOTES AND ERRORS ************************

Level of confidence for all confidence intervals in output:

95.0000

Number of bootstrap samples for percentile bootstrap confidence intervals:

5000

NOTE: A heteroscedasticity consistent standard error and covariance matrix estimator was used.

***************** PROCESS Procedure for SPSS Version 3.5 *****************

Written by Andrew F. Hayes, Ph.D. www.afhayes.com

Documentation available in Hayes (2018). www.guilford.com/p/hayes3

**************************************************************************

**Model : 4**

**Y : FACT_G**

**X : Stigma_I**

**M : SOC = Sense of Coherence**

Covariates:

Age_cov Educatio Employme Social_p Insuranc Major_Ps Chemo_co Mastecto Radiothe

Sample

Size: 221

**************************************************************************

OUTCOME VARIABLE:

SOC

Model Summary

R R-sq MSE F(HC4) df1 df2 p

.45548 .20746 151.84085 6.66508 10.00000 210.00000 .00000

Model

coeff se(HC4) t p LLCI ULCI

constant 68.01609 7.67463 8.86246 .00000 52.88690 83.14528

Stigma_I -.82220 .17345 -4.74035 .00000 -1.16412 -.48028

Age_cov .16923 .09181 1.84326 .06670 -.01176 .35022

Educatio -.43019 2.51540 -.17102 .86437 -5.38886 4.52848

Employme 3.33713 2.92422 1.14120 .25508 -2.42746 9.10172

Social_p 1.41471 .66047 2.14199 .03335 .11272 2.71671

Insuranc -1.62359 3.14157 -.51681 .60583 -7.81665 4.56947

Major_Ps -4.96124 2.17111 -2.28512 .02330 -9.24121 -.68127

Chemo_co -1.48699 1.27542 -1.16588 .24498 -4.00126 1.02728

Mastecto -2.99414 1.22241 -2.44938 .01513 -5.40390 -.58437

Radiothe -.38276 2.33173 -.16415 .86977 -4.97936 4.21385

Standardized coefficients

coeff

Stigma_I -.29932

Age_cov .11422

Educatio -.01251

Employme .09234

Social_p .15071

Insuranc -.03452

Major_Ps -.15700

Chemo_co -.09364

Mastecto -.17827

Radiothe -.01389

| **Benjamini-Hochberg Procedure** | | | | |
| --- | --- | --- | --- | --- |
|  |  | **Ascending P-value s** | **I = ranking** | **(I/10)* 0.10** |
|  | **Stigma** | **.0002** | **1** | **.01** |
|  | **Mastectomy** | **.015** | **2** | **.02** |
|  | **Major Psychological issues** | **.023** | **3** | **.03** |
|  | **Social participation frequency** | **.033** | **4** | **.04** |
|  | **Age** | **~~.066~~** | **5** | **.05** |
|  | **Chemotherapy** | **~~.244~~** | **6** | **.06** |
|  | **Employment** | **~~.255~~** | **7** | **.07** |
|  | **Insurance** | **~~.605~~** | **8** | **.08** |
|  | **Education** | **~~.864~~** | **9** | **.09** |
|  | **Radiotherapy** | **~~.869~~** | **10** | **.10** |

**************************************************************************

OUTCOME VARIABLE:

FACT_G

Model Summary

R R-sq MSE F(HC4) df1 df2 p

.58515 .34240 186.53379 8.94069 11.00000 209.00000 .00000

Model

coeff se(HC4) t p LLCI ULCI

constant 77.28155 9.79668 7.88855 .00000 57.96857 96.59453

Stigma_I -1.25899 .24295 -5.18217 .00000 -1.73793 -.78005

SOC .30451 .07800 3.90400 .00013 .15074 .45828

Age_cov -.15769 .10167 -1.55099 .12242 -.35813 .04274

Educatio -3.94277 2.53631 -1.55453 .12157 -8.94281 1.05727

Employme -4.33027 2.78875 -1.55276 .12199 -9.82795 1.16741

Social_p 1.07117 .83488 1.28301 .20091 -.57471 2.71704

Insuranc 3.93047 3.30060 1.19084 .23507 -2.57626 10.43720

Major_Ps -3.98698 2.54692 -1.56541 .11900 -9.00794 1.03397

Chemo_co -1.33894 1.44413 -.92716 .35491 -4.18587 1.50799

Mastecto -.51603 1.46179 -.35301 .72444 -3.39777 2.36572

Radiothe -.20290 2.50445 -.08101 .93551 -5.14012 4.73433

Standardized coefficients

coeff

Stigma_I -.37758

SOC .25086

Age_cov -.08768

Educatio -.09445

Employme -.09871

Social_p .09401

Insuranc .06885

Major_Ps -.10394

Chemo_co -.06946

Mastecto -.02531

Radiothe -.00606

| **Benjamini-Hochberg Procedure** | | | | |
| --- | --- | --- | --- | --- |
|  |  | **Ascending P-value s** | **I = ranking** | **(I/11)* 0.10** |
|  | **Stigma** | **.00000** | **1** | **.009** |
|  | **Sense of Coherence** | **.00013** | **2** | **.01818** |
|  | **Major Psychological issues** | **~~.119~~** | **3** | **.027** |
|  | **Education** | **~~.1215~~** | **4** | **.03636** |
|  | **Employment** | **~~.1219~~** | **5** | **.045** |
|  | **Age** | **~~.122~~** | **6** | **.054** |
|  | **Social participation frequency** | **~~.200~~** | **7** | **.063** |
|  | **Insurance** | **~~.235~~** | **8** | **.072** |
|  | **Chemotherapy** | **~~.354~~** | **9** | **.081** |
|  | **Mastectomy** | **~~.724~~** | **10** | **.0909** |
|  | **Radiotherapy** | **~~.935~~** | **11** | **.100** |

************************** TOTAL EFFECT MODEL ****************************

OUTCOME VARIABLE:

FACT_G

Model Summary

R R-sq MSE F(HC4) df1 df2 p

.54086 .29253 199.72527 8.08476 10.00000 210.00000 .00000

Model

coeff se(HC4) t p LLCI ULCI

constant 97.99319 8.46450 11.57696 .00000 81.30691 114.67948

Stigma_I -1.50936 .23571 -6.40348 .00000 -1.97401 -1.04470

Age_cov -.10616 .10653 -.99649 .32016 -.31617 .10385

Educatio -4.07377 2.63209 -1.54773 .12319 -9.26248 1.11495

Employme -3.31407 3.03693 -1.09126 .27641 -9.30086 2.67271

Social_p 1.50196 .78438 1.91484 .05687 -.04430 3.04823

Insuranc 3.43607 3.55197 .96737 .33447 -3.56602 10.43815

Major_Ps -5.49774 2.70660 -2.03124 .04349 -10.83332 -.16216

Chemo_co -1.79174 1.46240 -1.22521 .22187 -4.67460 1.09112

Mastecto -1.42777 1.48589 -.96089 .33771 -4.35695 1.50141

Radiothe -.31945 2.66992 -.11965 .90488 -5.58274 4.94384

Standardized coefficients

coeff

Stigma_I -.45267

Age_cov -.05903

Educatio -.09758

Employme -.07554

Social_p .13181

Insuranc .06019

Major_Ps -.14332

Chemo_co -.09295

Mastecto -.07003

Radiothe -.00955

| **Benjamini-Hochberg Procedure** | | | | |
| --- | --- | --- | --- | --- |
|  |  | **Ascending P-value s** | **I = ranking** | **(I/10)* 0.10** |
|  | **Stigma** | **.00000** | **1** | **.01** |
|  | **Major Psychological issues** | **~~.043~~** | **2** | **.02** |
|  | **Social participation frequency** | **~~.056~~** | **3** | **.03** |
|  | **Education** | **~~.123~~** | **4** | **.04** |
|  | **Chemotherapy** | **~~.221~~** | **5** | **.05** |
|  | **Employment** | **~~.276~~** | **6** | **.06** |
|  | **Age** | **~~.320~~** | **7** | **.07** |
|  | **Insurance** | **~~.334~~** | **8** | **.08** |
|  | **Mastectomy** | **~~.337~~** | **9** | **.09** |
|  | **Radiotherapy** | **~~.904~~** | **10** | **.10** |

************** TOTAL, DIRECT, AND INDIRECT EFFECTS OF X ON Y **************

Total effect of X on Y

Effect se(HC4) t p LLCI ULCI c_ps c_cs

-1.50936 .23571 -6.40348 .00000 -1.97401 -1.04470 -.09195 -.45267

Direct effect of X on Y

Effect se(HC4) t p LLCI ULCI c'_ps c'_cs

-1.25899 .24295 -5.18217 .00000 -1.73793 -.78005 -.07669 -.37758

Indirect effect(s) of X on Y:

Effect BootSE BootLLCI BootULCI

SOC -.25037 .07479 -.40882 -.11397

Partially standardized indirect effect(s) of X on Y:

Effect BootSE BootLLCI BootULCI

SOC -.01525 .00449 -.02457 -.00715

Completely standardized indirect effect(s) of X on Y:

Effect BootSE BootLLCI BootULCI

SOC -.07509 .02191 -.12098 -.03461

*********************** ANALYSIS NOTES AND ERRORS ************************

Level of confidence for all confidence intervals in output:

95.0000

Number of bootstrap samples for percentile bootstrap confidence intervals:

5000

NOTE: A heteroscedasticity consistent standard error and covariance matrix estimator was used.

***************** PROCESS Procedure for SPSS Version 3.5 *****************

Written by Andrew F. Hayes, Ph.D. www.afhayes.com

Documentation available in Hayes (2018). www.guilford.com/p/hayes3

**************************************************************************

**Model : 4**

**Y : FACT_G**

**X : Stigma_I**

**M : Emotional informational**

Covariates:

Age_cov Educatio Employme Social_p Insuranc Major_Ps Chemo_co Mastecto Radiothe

Sample

Size: 221

**************************************************************************

OUTCOME VARIABLE:

Emoinfo

Model Summary

R R-sq MSE F df1 df2 p

.36001 .12960 .06415 3.12695 10.00000 210.00000 .00096

Model

coeff se t p LLCI ULCI

constant .51430 .15025 3.42300 .00074 .21811 .81049

Stigma_I -.01133 .00364 -3.10925 .00214 -.01851 -.00414

Age_cov .00164 .00193 .84938 .39664 -.00217 .00546

Educatio -.01597 .05186 -.30795 .75843 -.11820 .08626

Employme -.01900 .05493 -.34599 .72969 -.12728 .08928

Social_p .00346 .01209 .28655 .77474 -.02037 .02730

Insuranc .04089 .06097 .67054 .50325 -.07931 .16108

Major_Ps -.13273 .04104 -3.23408 .00142 -.21364 -.05183

Chemo_co -.00108 .02584 -.04187 .96664 -.05202 .04986

Mastecto -.01604 .02436 -.65839 .51101 -.06406 .03198

Radiothe .03875 .04418 .87701 .38148 -.04835 .12585

Standardized coefficients

coeff

Stigma_I -.21022

Age_cov .05653

Educatio -.02368

Employme -.02681

Social_p .01882

Insuranc .04432

Major_Ps -.21415

Chemo_co -.00347

Mastecto -.04868

Radiothe .07168

| **Benjamini-Hochberg Procedure** | | | | |
| --- | --- | --- | --- | --- |
|  |  | **Ascending P-value s** | **I = ranking** | **(I/10)* 0.10** |
|  | **Major Psychological issues** | **.001** | **1** | **.01** |
|  | **Stigma** | **.002** | **2** | **.02** |
|  | **Radiotherapy** | **~~.381~~** | **3** | **.03** |
|  | **Age** | **~~.396~~** | **4** | **.04** |
|  | **Insurance** | **~~.503~~** | **5** | **.05** |
|  | **Mastectomy** | **~~.511~~** | **6** | **.06** |
|  | **Employment** | **~~.729~~** | **7** | **.07** |
|  | **Education** | **~~.758~~** | **8** | **.08** |
|  | **Social participation frequency** | **~~.774~~** | **9** | **.09** |
|  | **Chemotherapy** | **~~.966~~** | **10** | **.10** |

**************************************************************************

OUTCOME VARIABLE:

FACT_G

Model Summary

R R-sq MSE F df1 df2 p

.57913 .33540 188.52145 9.58842 11.00000 209.00000 .00000

Model

coeff se t p LLCI ULCI

constant 90.92958 8.36893 10.86514 .00000 74.43123 107.42792

Stigma_I -1.35380 .20196 -6.70349 .00000 -1.75193 -.95567

Emoinfo 13.73447 3.74078 3.67155 .00031 6.35997 21.10898

Age_cov -.12872 .10504 -1.22553 .22175 -.33579 .07834

Educatio -3.85443 2.81184 -1.37079 .17191 -9.39763 1.68877

Employme -3.05306 2.97843 -1.02506 .30652 -8.92467 2.81856

Social_p 1.45437 .65562 2.21832 .02761 .16190 2.74685

Insuranc 2.87453 3.30886 .86874 .38599 -3.64849 9.39756

Major_Ps -3.67473 2.27957 -1.61203 .10846 -8.16862 .81916

Chemo_co -1.77688 1.40087 -1.26841 .20606 -4.53853 .98477

Mastecto -1.20751 1.32180 -.91354 .36201 -3.81327 1.39825

Radiothe -.85165 2.39950 -.35493 .72300 -5.58197 3.87868

Standardized coefficients

coeff

Stigma_I -.40602

Emoinfo .22192

Age_cov -.07157

Educatio -.09233

Employme -.06959

Social_p .12764

Insuranc .05035

Major_Ps -.09580

Chemo_co -.09218

Mastecto -.05923

Radiothe -.02545

| **Benjamini-Hochberg Procedure** | | | | |
| --- | --- | --- | --- | --- |
|  |  | **Ascending P-value s** | **I = ranking** | **(I/11)* 0.10** |
|  | **Stigma** | **.00000** | **1** | **.009** |
|  | **Emotional informational support** | **.00031** | **2** | **.01818** |
|  | **Social participation frequency** | **~~.02761~~** | **3** | **.02727** |
|  | **Major Psychological issues** | **~~.108~~** | **4** | **.03636** |
|  | **Education** | **~~.171~~** | **5** | **.045** |
|  | **Chemotherapy** | **~~.206~~** | **6** | **.054** |
|  | **Age** | **~~.221~~** | **7** | **.063** |
|  | **Employment** | **~~.306~~** | **8** | **.072** |
|  | **Mastectomy** | **~~.362~~** | **9** | **.081** |
|  | **Insurance** | **~~.385~~** | **10** | **.0909** |
|  | **Radiotherapy** | **~~.723~~** | **11** | **.100** |

************************** TOTAL EFFECT MODEL ****************************

OUTCOME VARIABLE:

FACT_G

Model Summary

R R-sq MSE F df1 df2 p

.54086 .29253 199.72527 8.68320 10.00000 210.00000 .00000

Model

coeff se t p LLCI ULCI

constant 97.99319 8.38332 11.68906 .00000 81.46693 114.51946

Stigma_I -1.50936 .20324 -7.42632 .00000 -1.91002 -1.10870

Age_cov -.10616 .10793 -.98363 .32643 -.31892 .10660

Educatio -4.07377 2.89353 -1.40789 .16064 -9.77786 1.63033

Employme -3.31407 3.06478 -1.08134 .28079 -9.35576 2.72761

Social_p 1.50196 .67469 2.22616 .02707 .17193 2.83200

Insuranc 3.43607 3.40212 1.00998 .31367 -3.27063 10.14276

Major_Ps -5.49774 2.28999 -2.40077 .01723 -10.01205 -.98342

Chemo_co -1.79174 1.44189 -1.24263 .21539 -4.63418 1.05069

Mastecto -1.42777 1.35910 -1.05052 .29468 -4.10701 1.25146

Radiothe -.31945 2.46526 -.12958 .89702 -5.17928 4.54038

Standardized coefficients

coeff

Stigma_I -.45267

Age_cov -.05903

Educatio -.09758

Employme -.07554

Social_p .13181

Insuranc .06019

Major_Ps -.14332

Chemo_co -.09295

Mastecto -.07003

Radiothe -.00955

| **Benjamini-Hochberg Procedure** | | | | |
| --- | --- | --- | --- | --- |
|  |  | **Ascending P-value s** | **I = ranking** | **(I/10)* 0.10** |
|  | **Stigma** | **.00000** | **1** | **.01** |
|  | **Major Psychological issues** | **.017** | **2** | **.02** |
|  | **Social participation frequency** | **.027** | **3** | **.03** |
|  | **Education** | **~~.160~~** | **4** | **.04** |
|  | **Chemotherapy** | **~~.215~~** | **5** | **.05** |
|  | **Mastectomy** | **~~.294~~** | **6** | **.06** |
|  | **Employment** | **~~.280~~** | **7** | **.07** |
|  | **Insurance** | **~~.313~~** | **8** | **.08** |
|  | **Age** | **~~.326~~** | **9** | **.09** |
|  | **Radiotherapy** | **~~.897~~** | **10** | **.10** |

************** TOTAL, DIRECT, AND INDIRECT EFFECTS OF X ON Y **************

Total effect of X on Y

Effect se t p LLCI ULCI c_ps c_cs

-1.50936 .20324 -7.42632 .00000 -1.91002 -1.10870 -.09195 -.45267

Direct effect of X on Y

Effect se t p LLCI ULCI c'_ps c'_cs

-1.35380 .20196 -6.70349 .00000 -1.75193 -.95567 -.08247 -.40602

Indirect effect(s) of X on Y:

Effect BootSE BootLLCI BootULCI

Emoinfo -.15555 .05935 -.28458 -.05226

Partially standardized indirect effect(s) of X on Y:

Effect BootSE BootLLCI BootULCI

Emoinfo -.00948 .00356 -.01708 -.00326

Completely standardized indirect effect(s) of X on Y:

Effect BootSE BootLLCI BootULCI

Emoinfo -.04665 .01783 -.08542 -.01558

*********************** ANALYSIS NOTES AND ERRORS ************************

Level of confidence for all confidence intervals in output:

95.0000

Number of bootstrap samples for percentile bootstrap confidence intervals:

5000

***************** PROCESS Procedure for SPSS Version 3.5 *****************

Written by Andrew F. Hayes, Ph.D. www.afhayes.com

Documentation available in Hayes (2018). www.guilford.com/p/hayes3

**************************************************************************

**Model : 4**

**Y : FACT_G**

**X : Stigma_I**

**M : Affectionate**

Covariates:

Age_cov Educatio Employme Social_p Insuranc Major_Ps Chemo_co Mastecto Radiothe

Sample

Size: 221

**************************************************************************

OUTCOME VARIABLE:

Affectio

Model Summary

R R-sq MSE F df1 df2 p

.37797 .14286 .05276 3.50013 10.00000 210.00000 .00027

Model

coeff se t p LLCI ULCI

constant .48253 .13625 3.54144 .00049 .21393 .75112

Stigma_I -.00910 .00330 -2.75500 .00638 -.01561 -.00259

Age_cov .00105 .00175 .59605 .55178 -.00241 .00450

Educatio -.01455 .04703 -.30944 .75729 -.10726 .07815

Employme -.02925 .04981 -.58719 .55770 -.12744 .06894

Social_p .00296 .01097 .27031 .78719 -.01865 .02458

Insuranc .07932 .05529 1.43455 .15290 -.02968 .18832

Major_Ps -.11830 .03722 -3.17845 .00170 -.19167 -.04493

Chemo_co -.00828 .02343 -.35349 .72408 -.05448 .03791

Mastecto -.03515 .02209 -1.59143 .11302 -.07870 .00839

Radiothe .05027 .04007 1.25454 .21104 -.02872 .12925

Standardized coefficients

coeff

Stigma_I -.18484

Age_cov .03937

Educatio -.02361

Employme -.04515

Social_p .01762

Insuranc .09410

Major_Ps -.20885

Chemo_co -.02910

Mastecto -.11678

Radiothe .10175

| **Benjamini-Hochberg Procedure** | | | | |
| --- | --- | --- | --- | --- |
|  |  | **Ascending P-value s** | **I = ranking** | **(I/10)* 0.10** |
|  | **Major Psychological issues** | **.001** | **1** | **.01** |
|  | **Stigma** | **.006** | **2** | **.02** |
|  | **Mastectomy** | **~~.113~~** | **3** | **.03** |
|  | **Insurance** | **~~.152~~** | **4** | **.04** |
|  | **Radiotherapy** | **~~.211~~** | **5** | **.05** |
|  | **Age** | **~~.551~~** | **6** | **.06** |
|  | **Employment** | **~~.557~~** | **7** | **.07** |
|  | **Chemotherapy** | **~~.724~~** | **8** | **.08** |
|  | **Education** | **~~.757~~** | **9** | **.09** |
|  | **Social participation frequency** | **~~.787~~** | **10** | **.10** |

**************************************************************************

OUTCOME VARIABLE:

FACT_G

Model Summary

R R-sq MSE F df1 df2 p

.58532 .34260 186.47852 9.90162 11.00000 209.00000 .00000

Model

coeff se t p LLCI ULCI

constant 90.09508 8.33893 10.80415 .00000 73.65587 106.53428

Stigma_I -1.36040 .19991 -6.80519 .00000 -1.75449 -.96631

Affectio 16.36830 4.10265 3.98969 .00009 8.28042 24.45617

Age_cov -.12327 .10437 -1.18107 .23892 -.32903 .08249

Educatio -3.83557 2.79657 -1.37153 .17168 -9.34866 1.67753

Employme -2.83532 2.96383 -.95664 .33985 -8.67817 3.00752

Social_p 1.45345 .65204 2.22906 .02687 .16802 2.73887

Insuranc 2.13772 3.30343 .64712 .51826 -4.37461 8.65004

Major_Ps -3.56142 2.26535 -1.57213 .11743 -8.02728 .90444

Chemo_co -1.65615 1.39367 -1.18834 .23605 -4.40360 1.09130

Mastecto -.85238 1.32116 -.64517 .51952 -3.45687 1.75212

Radiothe -1.14222 2.39101 -.47771 .63335 -5.85582 3.57138

Standardized coefficients

coeff

Stigma_I -.40799

Affectio .24169

Age_cov -.06854

Educatio -.09188

Employme -.06463

Social_p .12756

Insuranc .03745

Major_Ps -.09284

Chemo_co -.08592

Mastecto -.04181

Radiothe -.03414

| **Benjamini-Hochberg Procedure** | | | | |
| --- | --- | --- | --- | --- |
|  |  | **Ascending P-value s** | **I = ranking** | **(I/11)* 0.10** |
|  | **Stigma** | **.00000** | **1** | **.009** |
|  | **Affectionate support** | **.00009** | **2** | **.01818** |
|  | **Social participation frequency** | **~~.02687~~** | **3** | **.02727** |
|  | **Major Psychological issues** | **~~.117~~** | **4** | **.03636** |
|  | **Education** | **~~.171~~** | **5** | **.045** |
|  | **Chemotherapy** | **~~.236~~** | **6** | **.054** |
|  | **Age** | **~~.238~~** | **7** | **.063** |
|  | **Employment** | **~~.339~~** | **8** | **.072** |
|  | **Insurance** | **~~.518~~** | **9** | **.081** |
|  | **Mastectomy** | **~~.519~~** | **10** | **.0909** |
|  | **Radiotherapy** | **~~.633~~** | **11** | **.100** |

************************** TOTAL EFFECT MODEL ****************************

OUTCOME VARIABLE:

FACT_G

Model Summary

R R-sq MSE F df1 df2 p

.54086 .29253 199.72527 8.68320 10.00000 210.00000 .00000

Model

coeff se t p LLCI ULCI

constant 97.99319 8.38332 11.68906 .00000 81.46693 114.51946

Stigma_I -1.50936 .20324 -7.42632 .00000 -1.91002 -1.10870

Age_cov -.10616 .10793 -.98363 .32643 -.31892 .10660

Educatio -4.07377 2.89353 -1.40789 .16064 -9.77786 1.63033

Employme -3.31407 3.06478 -1.08134 .28079 -9.35576 2.72761

Social_p 1.50196 .67469 2.22616 .02707 .17193 2.83200

Insuranc 3.43607 3.40212 1.00998 .31367 -3.27063 10.14276

Major_Ps -5.49774 2.28999 -2.40077 .01723 -10.01205 -.98342

Chemo_co -1.79174 1.44189 -1.24263 .21539 -4.63418 1.05069

Mastecto -1.42777 1.35910 -1.05052 .29468 -4.10701 1.25146

Radiothe -.31945 2.46526 -.12958 .89702 -5.17928 4.54038

Standardized coefficients

coeff

Stigma_I -.45267

Age_cov -.05903

Educatio -.09758

Employme -.07554

Social_p .13181

Insuranc .06019

Major_Ps -.14332

Chemo_co -.09295

Mastecto -.07003

Radiothe -.00955

| **Benjamini-Hochberg Procedure** | | | | |
| --- | --- | --- | --- | --- |
|  |  | **Ascending P-value s** | **I = ranking** | **(I/10)* 0.10** |
|  | **Stigma** | **.00000** | **1** | **.01** |
|  | **Major Psychological issues** | **.017** | **2** | **.02** |
|  | **Social participation frequency** | **.027** | **3** | **.03** |
|  | **Education** | **~~.160~~** | **4** | **.04** |
|  | **Chemotherapy** | **~~.215~~** | **5** | **.05** |
|  | **Mastectomy** | **~~.294~~** | **6** | **.06** |
|  | **Employment** | **~~.280~~** | **7** | **.07** |
|  | **Insurance** | **~~.313~~** | **8** | **.08** |
|  | **Age** | **~~.326~~** | **9** | **.09** |
|  | **Radiotherapy** | **~~.897~~** | **10** | **.10** |

************** TOTAL, DIRECT, AND INDIRECT EFFECTS OF X ON Y **************

Total effect of X on Y

Effect se t p LLCI ULCI c_ps c_cs

-1.50936 .20324 -7.42632 .00000 -1.91002 -1.10870 -.09195 -.45267

Direct effect of X on Y

Effect se t p LLCI ULCI c'_ps c'_cs

-1.36040 .19991 -6.80519 .00000 -1.75449 -.96631 -.08287 -.40799

Indirect effect(s) of X on Y:

Effect BootSE BootLLCI BootULCI

Affectio -.14896 .06496 -.28711 -.03102

Partially standardized indirect effect(s) of X on Y:

Effect BootSE BootLLCI BootULCI

Affectio -.00907 .00390 -.01721 -.00192

Completely standardized indirect effect(s) of X on Y:

Effect BootSE BootLLCI BootULCI

Affectio -.04467 .01962 -.08681 -.00909

*********************** ANALYSIS NOTES AND ERRORS ************************

Level of confidence for all confidence intervals in output:

95.0000

Number of bootstrap samples for percentile bootstrap confidence intervals:

5000

***************** PROCESS Procedure for SPSS Version 3.5 *****************

Written by Andrew F. Hayes, Ph.D. www.afhayes.com

Documentation available in Hayes (2018). www.guilford.com/p/hayes3

**************************************************************************

**Model : 4**

**Y : FACT_G**

**X : Stigma_I**

**M : Tangible**

Covariates:

Age_cov Educatio Employme Social_p Insuranc Major_Ps Chemo_co Mastecto Radiothe

Sample

Size: 221

**************************************************************************

OUTCOME VARIABLE:

Tangible

Model Summary

R R-sq MSE F df1 df2 p

.36445 .13282 .05649 3.21649 10.00000 210.00000 .00071

Model

coeff se t p LLCI ULCI

constant .39310 .14099 2.78817 .00579 .11516 .67103

Stigma_I -.00884 .00342 -2.58739 .01035 -.01558 -.00211

Age_cov .00193 .00182 1.06059 .29010 -.00165 .00550

Educatio .01051 .04866 .21603 .82918 -.08542 .10644

Employme .00466 .05154 .09047 .92800 -.09694 .10627

Social_p -.01229 .01135 -1.08312 .28000 -.03466 .01008

Insuranc .08211 .05722 1.43514 .15274 -.03068 .19490

Major_Ps -.12284 .03851 -3.18964 .00164 -.19876 -.04692

Chemo_co -.00498 .02425 -.20536 .83749 -.05278 .04282

Mastecto -.02154 .02286 -.94221 .34717 -.06659 .02352

Radiothe .05682 .04146 1.37039 .17203 -.02491 .13855

Standardized coefficients

coeff

Stigma_I -.17461

Age_cov .07046

Educatio .01658

Employme .00700

Social_p -.07100

Insuranc .09469

Major_Ps -.21081

Chemo_co -.01701

Mastecto -.06954

Radiothe .11179

| **Benjamini-Hochberg Procedure** | | | | |
| --- | --- | --- | --- | --- |
|  |  | **Ascending P-value s** | **I = ranking** | **(I/10)* 0.10** |
|  | **Major Psychological issues** | **.001** | **1** | **.01** |
|  | **Stigma** | **.01** | **2** | **.02** |
|  | **Insurance** | **~~.152~~** | **3** | **.03** |
|  | **Radiotherapy** | **~~.172~~** | **4** | **.04** |
|  | **Social participation frequency** | **~~.280~~** | **5** | **.05** |
|  | **Age** | **~~.290~~** | **6** | **.06** |
|  | **Mastectomy** | **~~.347~~** | **7** | **.07** |
|  | **Education** | **~~.829~~** | **8** | **.08** |
|  | **Chemotherapy** | **~~.837~~** | **9** | **.09** |
|  | **Employment** | **~~.928~~** | **10** | **.10** |

**************************************************************************

OUTCOME VARIABLE:

FACT_G

Model Summary

R R-sq MSE F df1 df2 p

.55531 .30837 196.18884 8.47114 11.00000 209.00000 .00000

Model

coeff se t p LLCI ULCI

constant 94.49613 8.46117 11.16822 .00000 77.81595 111.17630

Stigma_I -1.43068 .20462 -6.99181 .00000 -1.83407 -1.02729

Tangible 8.89623 4.06676 2.18755 .02981 .87911 16.91335

Age_cov -.12329 .10725 -1.14948 .25167 -.33472 .08815

Educatio -4.16729 2.86812 -1.45297 .14773 -9.82144 1.48687

Employme -3.35556 3.03759 -1.10468 .27057 -9.34380 2.63268

Social_p 1.61130 .67055 2.40293 .01714 .28938 2.93321

Insuranc 2.70558 3.38837 .79849 .42549 -3.97418 9.38534

Major_Ps -4.40493 2.32395 -1.89545 .05941 -8.98633 .17647

Chemo_co -1.74744 1.42921 -1.22266 .22283 -4.56496 1.07008

Mastecto -1.23618 1.34986 -.91579 .36083 -3.89728 1.42491

Radiothe -.82490 2.45424 -.33611 .73712 -5.66313 4.01334

Standardized coefficients

coeff

Stigma_I -.42907

Tangible .13514

Age_cov -.06855

Educatio -.09982

Employme -.07649

Social_p .14141

Insuranc .04739

Major_Ps -.11483

Chemo_co -.09065

Mastecto -.06063

Radiothe -.02465

| **Benjamini-Hochberg Procedure** | | | | |
| --- | --- | --- | --- | --- |
|  |  | **Ascending P-values** | **I = ranking** | **(I/11)* 0.10** |
|  | **Stigma** | **.00000** | **1** | **.009** |
|  | **Social participation frequency** | **.017** | **2** | **.018** |
|  | **Tangible support** | **~~.029~~** | **3** | **.027** |
|  | **Major Psychological issues** | **~~.059~~** | **4** | **.03636** |
|  | **Education** | **~~.147~~** | **5** | **.045** |
|  | **Chemotherapy** | **~~.222~~** | **6** | **.054** |
|  | **Age** | **~~.251~~** | **7** | **.063** |
|  | **Employment** | **~~.270~~** | **8** | **.072** |
|  | **Mastectomy** | **~~.360~~** | **9** | **.081** |
|  | **Insurance** | **~~.425~~** | **10** | **.0909** |
|  | **Radiotherapy** | **~~.737~~** | **11** | **.100** |

************************** TOTAL EFFECT MODEL ****************************

OUTCOME VARIABLE:

FACT_G

Model Summary

R R-sq MSE F df1 df2 p

.54086 .29253 199.72527 8.68320 10.00000 210.00000 .00000

Model

coeff se t p LLCI ULCI

constant 97.99319 8.38332 11.68906 .00000 81.46693 114.51946

Stigma_I -1.50936 .20324 -7.42632 .00000 -1.91002 -1.10870

Age_cov -.10616 .10793 -.98363 .32643 -.31892 .10660

Educatio -4.07377 2.89353 -1.40789 .16064 -9.77786 1.63033

Employme -3.31407 3.06478 -1.08134 .28079 -9.35576 2.72761

Social_p 1.50196 .67469 2.22616 .02707 .17193 2.83200

Insuranc 3.43607 3.40212 1.00998 .31367 -3.27063 10.14276

Major_Ps -5.49774 2.28999 -2.40077 .01723 -10.01205 -.98342

Chemo_co -1.79174 1.44189 -1.24263 .21539 -4.63418 1.05069

Mastecto -1.42777 1.35910 -1.05052 .29468 -4.10701 1.25146

Radiothe -.31945 2.46526 -.12958 .89702 -5.17928 4.54038

Standardized coefficients

coeff

Stigma_I -.45267

Age_cov -.05903

Educatio -.09758

Employme -.07554

Social_p .13181

Insuranc .06019

Major_Ps -.14332

Chemo_co -.09295

Mastecto -.07003

Radiothe -.00955

| **Benjamini-Hochberg Procedure** | | | | |
| --- | --- | --- | --- | --- |
|  |  | **Ascending P-value s** | **I = ranking** | **(I/10)* 0.10** |
|  | **Stigma** | **.00000** | **1** | **.01** |
|  | **Major Psychological issues** | **.017** | **2** | **.02** |
|  | **Social participation frequency** | **.027** | **3** | **.03** |
|  | **Education** | **~~.160~~** | **4** | **.04** |
|  | **Chemotherapy** | **~~.215~~** | **5** | **.05** |
|  | **Mastectomy** | **~~.294~~** | **6** | **.06** |
|  | **Employment** | **~~.280~~** | **7** | **.07** |
|  | **Insurance** | **~~.313~~** | **8** | **.08** |
|  | **Age** | **~~.326~~** | **9** | **.09** |
|  | **Radiotherapy** | **~~.897~~** | **10** | **.10** |

************** TOTAL, DIRECT, AND INDIRECT EFFECTS OF X ON Y **************

Total effect of X on Y

Effect se t p LLCI ULCI c_ps c_cs

-1.50936 .20324 -7.42632 .00000 -1.91002 -1.10870 -.09195 -.45267

Direct effect of X on Y

Effect se t p LLCI ULCI c'_ps c'_cs

-1.43068 .20462 -6.99181 .00000 -1.83407 -1.02729 -.08715 -.42907

Indirect effect(s) of X on Y:

Effect BootSE BootLLCI BootULCI

Tangible -.07868 .04757 -.18795 -.00214

Partially standardized indirect effect(s) of X on Y:

Effect BootSE BootLLCI BootULCI

Tangible -.00479 .00289 -.01141 -.00013

Completely standardized indirect effect(s) of X on Y:

Effect BootSE BootLLCI BootULCI

Tangible -.02360 .01435 -.05655 -.00062

*********************** ANALYSIS NOTES AND ERRORS ************************

Level of confidence for all confidence intervals in output:

95.0000

Number of bootstrap samples for percentile bootstrap confidence intervals:

5000

***************** PROCESS Procedure for SPSS Version 3.5 *****************

Written by Andrew F. Hayes, Ph.D. www.afhayes.com

Documentation available in Hayes (2018). www.guilford.com/p/hayes3

**************************************************************************

**Model : 4**

**Y : FACT_G**

**X : Stigma_I**

**M : Positive social interaction**

Covariates:

Age_cov Educatio Employme Social_p Insuranc Major_Ps Chemo_co Mastecto Radiothe

Sample

Size: 221

**************************************************************************

OUTCOME VARIABLE:

Positive

Model Summary

R R-sq MSE F df1 df2 p

.34064 .11604 .05623 2.75668 10.00000 210.00000 .00325

Model

coeff se t p LLCI ULCI

constant .52551 .14067 3.73583 .00024 .24821 .80281

Stigma_I -.01295 .00341 -3.79812 .00019 -.01968 -.00623

Age_cov .00060 .00181 .33404 .73868 -.00297 .00417

Educatio .01962 .04855 .40417 .68650 -.07609 .11533

Employme .02577 .05143 .50115 .61679 -.07560 .12715

Social_p .00119 .01132 .10550 .91608 -.02112 .02351

Insuranc .05715 .05709 1.00117 .31790 -.05538 .16969

Major_Ps -.08447 .03842 -2.19820 .02903 -.16021 -.00872

Chemo_co -.00020 .02419 -.00842 .99329 -.04790 .04749

Mastecto -.00918 .02281 -.40250 .68773 -.05414 .03578

Radiothe .02742 .04137 .66295 .50809 -.05412 .10897

Standardized coefficients

coeff

Stigma_I -.25878

Age_cov .02241

Educatio .03131

Employme .03914

Social_p .00698

Insuranc .06669

Major_Ps -.14669

Chemo_co -.00070

Mastecto -.02999

Radiothe .05460

| **Benjamini-Hochberg Procedure** | | | | |
| --- | --- | --- | --- | --- |
|  |  | **Ascending P-value s** | **I = ranking** | **(I/10)* 0.10** |
|  | **Stigma** | **.0001** | **1** | **.01** |
|  | **Major Psychological issues** | **~~.029~~** | **2** | **.02** |
|  | **Insurance** | **~~.317~~** | **3** | **.03** |
|  | **Radiotherapy** | **~~.508~~** | **4** | **.04** |
|  | **Employment** | **~~.616~~** | **5** | **.05** |
|  | **Education** | **~~.686~~** | **6** | **.06** |
|  | **Mastectomy** | **~~.687~~** | **7** | **.07** |
|  | **Age** | **~~.738~~** | **8** | **.08** |
|  | **Social participation frequency** | **~~.916~~** | **9** | **.09** |
|  | **Chemotherapy** | **~~.993~~** | **10** | **.10** |

**************************************************************************

OUTCOME VARIABLE:

FACT_G

Model Summary

R R-sq MSE F df1 df2 p

.60170 .36205 180.96115 10.78281 11.00000 209.00000 .00000

Model

coeff se t p LLCI ULCI

constant 88.17569 8.24071 10.70001 .00000 71.93012 104.42126

Stigma_I -1.26737 .20000 -6.33700 .00000 -1.66164 -.87311

Positive 18.68183 3.91461 4.77234 .00000 10.96464 26.39901

Age_cov -.11746 .10276 -1.14307 .25432 -.32004 .08512

Educatio -4.44036 2.75533 -1.61156 .10857 -9.87216 .99143

Employme -3.79554 2.91901 -1.30028 .19494 -9.55001 1.95894

Social_p 1.47965 .64223 2.30392 .02221 .21357 2.74573

Insuranc 2.36835 3.24609 .72960 .46645 -4.03092 8.76763

Major_Ps -3.91977 2.20470 -1.77792 .07687 -8.26608 .42653

Chemo_co -1.78793 1.37249 -1.30270 .19411 -4.49363 .91776

Mastecto -1.25629 1.29419 -.97072 .33281 -3.80762 1.29504

Radiothe -.83177 2.34905 -.35409 .72363 -5.46265 3.79911

Standardized coefficients

coeff

Stigma_I -.38009

Positive .28044

Age_cov -.06531

Educatio -.10637

Employme -.08652

Social_p .12985

Insuranc .04149

Major_Ps -.10218

Chemo_co -.09275

Mastecto -.06162

Radiothe -.02486

| **Benjamini-Hochberg Procedure** | | | | |
| --- | --- | --- | --- | --- |
|  |  | **Ascending P-value s** | **I = ranking** | **(I/11)* 0.10** |
|  | **Stigma** | **.00000** | **1** | **.009** |
|  | **Positive social interaction** | **.00000** | **2** | **.01818** |
|  | **Social participation frequency** | **.022** | **3** | **.02727** |
|  | **Major Psychological issues** | **~~.076~~** | **4** | **.03636** |
|  | **Education** | **~~.108~~** | **5** | **.045** |
|  | **Chemotherapy** | **~~.1941~~** | **6** | **.054** |
|  | **Employment** | **~~.1949~~** | **7** | **.063** |
|  | **Age** | **~~.254~~** | **8** | **.072** |
|  | **Mastectomy** | **~~.332~~** | **9** | **.081** |
|  | **Insurance** | **~~.466~~** | **10** | **.0909** |
|  | **Radiotherapy** | **~~.723~~** | **11** | **.100** |

************************** TOTAL EFFECT MODEL ****************************

OUTCOME VARIABLE:

FACT_G

Model Summary

R R-sq MSE F df1 df2 p

.54086 .29253 199.72527 8.68320 10.00000 210.00000 .00000

Model

coeff se t p LLCI ULCI

constant 97.99319 8.38332 11.68906 .00000 81.46693 114.51946

Stigma_I -1.50936 .20324 -7.42632 .00000 -1.91002 -1.10870

Age_cov -.10616 .10793 -.98363 .32643 -.31892 .10660

Educatio -4.07377 2.89353 -1.40789 .16064 -9.77786 1.63033

Employme -3.31407 3.06478 -1.08134 .28079 -9.35576 2.72761

Social_p 1.50196 .67469 2.22616 .02707 .17193 2.83200

Insuranc 3.43607 3.40212 1.00998 .31367 -3.27063 10.14276

Major_Ps -5.49774 2.28999 -2.40077 .01723 -10.01205 -.98342

Chemo_co -1.79174 1.44189 -1.24263 .21539 -4.63418 1.05069

Mastecto -1.42777 1.35910 -1.05052 .29468 -4.10701 1.25146

Radiothe -.31945 2.46526 -.12958 .89702 -5.17928 4.54038

Standardized coefficients

coeff

Stigma_I -.45267

Age_cov -.05903

Educatio -.09758

Employme -.07554

Social_p .13181

Insuranc .06019

Major_Ps -.14332

Chemo_co -.09295

Mastecto -.07003

Radiothe -.00955

| **Benjamini-Hochberg Procedure** | | | | |
| --- | --- | --- | --- | --- |
|  |  | **Ascending P-value s** | **I = ranking** | **(I/10)* 0.10** |
|  | **Stigma** | **.00000** | **1** | **.01** |
|  | **Major Psychological issues** | **.017** | **2** | **.02** |
|  | **Social participation frequency** | **.027** | **3** | **.03** |
|  | **Education** | **~~.160~~** | **4** | **.04** |
|  | **Chemotherapy** | **~~.215~~** | **5** | **.05** |
|  | **Mastectomy** | **~~.294~~** | **6** | **.06** |
|  | **Employment** | **~~.280~~** | **7** | **.07** |
|  | **Insurance** | **~~.313~~** | **8** | **.08** |
|  | **Age** | **~~.326~~** | **9** | **.09** |
|  | **Radiotherapy** | **~~.897~~** | **10** | **.10** |

************** TOTAL, DIRECT, AND INDIRECT EFFECTS OF X ON Y **************

Total effect of X on Y

Effect se t p LLCI ULCI c_ps c_cs

-1.50936 .20324 -7.42632 .00000 -1.91002 -1.10870 -.09195 -.45267

Direct effect of X on Y

Effect se t p LLCI ULCI c'_ps c'_cs

-1.26737 .20000 -6.33700 .00000 -1.66164 -.87311 -.07720 -.38009

Indirect effect(s) of X on Y:

Effect BootSE BootLLCI BootULCI

Positive -.24198 .07600 -.39875 -.10081

Partially standardized indirect effect(s) of X on Y:

Effect BootSE BootLLCI BootULCI

Positive -.01474 .00452 -.02422 -.00625

Completely standardized indirect effect(s) of X on Y:

Effect BootSE BootLLCI BootULCI

Positive -.07257 .02300 -.12053 -.02965

*********************** ANALYSIS NOTES AND ERRORS ************************

Level of confidence for all confidence intervals in output:

95.0000

Number of bootstrap samples for percentile bootstrap confidence intervals:

5000

***************** PROCESS Procedure for SPSS Version 3.5 *****************

Written by Andrew F. Hayes, Ph.D. www.afhayes.com

Documentation available in Hayes (2018). www.guilford.com/p/hayes3

**************************************************************************

**Model : 4**

**Y : FACT_G**

**X : Stigma_I**

**M : MOS_SSS = Total social support**

Covariates:

Age_cov Educatio Employme Social_p Insuranc Major_Ps Chemo_co Mastecto Radiothe

Sample

Size: 221

**************************************************************************

OUTCOME VARIABLE:

MOS_SSS

Model Summary

R R-sq MSE F df1 df2 p

.35287 .12451 .06905 2.98667 10.00000 210.00000 .00153

Model

coeff se t p LLCI ULCI

constant .46456 .15587 2.98037 .00322 .15728 .77183

Stigma_I -.01095 .00378 -2.89783 .00416 -.01840 -.00350

Age_cov .00169 .00201 .84386 .39971 -.00226 .00565

Educatio -.03744 .05380 -.69584 .48730 -.14349 .06862

Employme -.02411 .05698 -.42304 .67270 -.13644 .08823

Social_p -.00090 .01254 -.07168 .94292 -.02563 .02383

Insuranc .07701 .06326 1.21750 .22478 -.04768 .20171

Major_Ps -.12186 .04258 -2.86202 .00464 -.20579 -.03792

Chemo_co -.00198 .02681 -.07371 .94131 -.05483 .05087

Mastecto -.01605 .02527 -.63501 .52612 -.06586 .03377

Radiothe .05665 .04584 1.23597 .21785 -.03371 .14701

Standardized coefficients

coeff

Stigma_I -.19649

Age_cov .05633

Educatio -.05365

Employme -.03288

Social_p -.00472

Insuranc .08071

Major_Ps -.19006

Chemo_co -.00613

Mastecto -.04709

Radiothe .10131

| **Benjamini-Hochberg Procedure** | | | | |
| --- | --- | --- | --- | --- |
|  |  | **Ascending P-value s** | **I = ranking** | **(I/10)* 0.10** |
|  | **Stigma** | **.0041** | **1** | **.01** |
|  | **Major Psychological issues** | **.0046** | **2** | **.02** |
|  | **Radiotherapy** | **~~.217~~** | **3** | **.03** |
|  | **Insurance** | **~~.224~~** | **4** | **.04** |
|  | **Age** | **~~.399~~** | **5** | **.05** |
|  | **Education** | **~~.487~~** | **6** | **.06** |
|  | **Mastectomy** | **~~.526~~** | **7** | **.07** |
|  | **Employment** | **~~.672~~** | **8** | **.08** |
|  | **Chemotherapy** | **~~.941~~** | **9** | **.09** |
|  | **Social participation frequency** | **~~.942~~** | **10** | **.10** |

**************************************************************************

OUTCOME VARIABLE:

FACT_G

Model Summary

R R-sq MSE F df1 df2 p

.58314 .34005 187.20161 9.78998 11.00000 209.00000 .00000

Model

coeff se t p LLCI ULCI

constant 91.51780 8.28611 11.04473 .00000 75.18273 107.85287

Stigma_I -1.35671 .20066 -6.76112 .00000 -1.75230 -.96113

MOS_SSS 13.93889 3.59316 3.87928 .00014 6.85541 21.02238

Age_cov -.12976 .10467 -1.23980 .21644 -.33610 .07657

Educatio -3.55195 2.80457 -1.26649 .20675 -9.08083 1.97693

Employme -2.97806 2.96840 -1.00325 .31690 -8.82991 2.87380

Social_p 1.51450 .65320 2.31858 .02138 .22679 2.80221

Insuranc 2.36257 3.30534 .71478 .47555 -4.15350 8.87865

Major_Ps -3.79916 2.25986 -1.68115 .09423 -8.25419 .65588

Chemo_co -1.76420 1.39597 -1.26378 .20772 -4.51618 .98779

Mastecto -1.20410 1.31707 -.91423 .36165 -3.80054 1.39234

Radiothe -1.10913 2.39538 -.46303 .64383 -5.83134 3.61308

Standardized coefficients

coeff

Stigma_I -.40689

MOS_SSS .23298

Age_cov -.07215

Educatio -.08508

Employme -.06789

Social_p .13291

Insuranc .04138

Major_Ps -.09904

Chemo_co -.09152

Mastecto -.05906

Radiothe -.03315

| **Benjamini-Hochberg Procedure** | | | | |
| --- | --- | --- | --- | --- |
|  |  | **Ascending P-value s** | **I = ranking** | **(I/11)* 0.10** |
|  | **Stigma** | **.00000** | **1** | **.009** |
|  | **Total social support** | **.00000** | **2** | **.01818** |
|  | **Social participation frequency** | **.021** | **3** | **.02727** |
|  | **Major Psychological issues** | **~~.094~~** | **4** | **.03636** |
|  | **Education** | **~~.206~~** | **5** | **.045** |
|  | **Chemotherapy** | **~~.207~~** | **6** | **.054** |
|  | **Age** | **~~.216~~** | **7** | **.063** |
|  | **Employment** | **~~.316~~** | **8** | **.072** |
|  | **Mastectomy** | **~~.361~~** | **9** | **.081** |
|  | **Insurance** | **~~.475~~** | **10** | **.0909** |
|  | **Radiotherapy** | **~~.643~~** | **11** | **.100** |

************************** TOTAL EFFECT MODEL ****************************

OUTCOME VARIABLE:

FACT_G

Model Summary

R R-sq MSE F df1 df2 p

.54086 .29253 199.72527 8.68320 10.00000 210.00000 .00000

Model

coeff se t p LLCI ULCI

constant 97.99319 8.38332 11.68906 .00000 81.46693 114.51946

Stigma_I -1.50936 .20324 -7.42632 .00000 -1.91002 -1.10870

Age_cov -.10616 .10793 -.98363 .32643 -.31892 .10660

Educatio -4.07377 2.89353 -1.40789 .16064 -9.77786 1.63033

Employme -3.31407 3.06478 -1.08134 .28079 -9.35576 2.72761

Social_p 1.50196 .67469 2.22616 .02707 .17193 2.83200

Insuranc 3.43607 3.40212 1.00998 .31367 -3.27063 10.14276

Major_Ps -5.49774 2.28999 -2.40077 .01723 -10.01205 -.98342

Chemo_co -1.79174 1.44189 -1.24263 .21539 -4.63418 1.05069

Mastecto -1.42777 1.35910 -1.05052 .29468 -4.10701 1.25146

Radiothe -.31945 2.46526 -.12958 .89702 -5.17928 4.54038

Standardized coefficients

coeff

Stigma_I -.45267

Age_cov -.05903

Educatio -.09758

Employme -.07554

Social_p .13181

Insuranc .06019

Major_Ps -.14332

Chemo_co -.09295

Mastecto -.07003

Radiothe -.00955

| **Benjamini-Hochberg Procedure** | | | | |
| --- | --- | --- | --- | --- |
|  |  | **Ascending P-value s** | **I = ranking** | **(I/10)* 0.10** |
|  | **Stigma** | **.00000** | **1** | **.01** |
|  | **Major Psychological issues** | **.017** | **2** | **.02** |
|  | **Social participation frequency** | **.027** | **3** | **.03** |
|  | **Education** | **~~.160~~** | **4** | **.04** |
|  | **Chemotherapy** | **~~.215~~** | **5** | **.05** |
|  | **Mastectomy** | **~~.294~~** | **6** | **.06** |
|  | **Employment** | **~~.280~~** | **7** | **.07** |
|  | **Insurance** | **~~.313~~** | **8** | **.08** |
|  | **Age** | **~~.326~~** | **9** | **.09** |
|  | **Radiotherapy** | **~~.897~~** | **10** | **.10** |

************** TOTAL, DIRECT, AND INDIRECT EFFECTS OF X ON Y **************

Total effect of X on Y

Effect se t p LLCI ULCI c_ps c_cs

-1.50936 .20324 -7.42632 .00000 -1.91002 -1.10870 -.09195 -.45267

Direct effect of X on Y

Effect se t p LLCI ULCI c'_ps c'_cs

-1.35671 .20066 -6.76112 .00000 -1.75230 -.96113 -.08265 -.40689

Indirect effect(s) of X on Y:

Effect BootSE BootLLCI BootULCI

MOS_SSS -.15264 .06070 -.28086 -.04360

Partially standardized indirect effect(s) of X on Y:

Effect BootSE BootLLCI BootULCI

MOS_SSS -.00930 .00363 -.01698 -.00273

Completely standardized indirect effect(s) of X on Y:

Effect BootSE BootLLCI BootULCI

MOS_SSS -.04578 .01820 -.08439 -.01285

*********************** ANALYSIS NOTES AND ERRORS ************************

Level of confidence for all confidence intervals in output:

95.0000

Number of bootstrap samples for percentile bootstrap confidence intervals:

5000

***************** PROCESS Procedure for SPSS Version 3.5 *****************

Written by Andrew F. Hayes, Ph.D. www.afhayes.com

Documentation available in Hayes (2018). www.guilford.com/p/hayes3

**************************************************************************

**Model : 4**

**Y : FACT_G**

**X : Stigma_I**

**M : Positive reframing**

Covariates:

Age_cov Educatio Employme Social_p Insuranc Major_Ps Chemo_co Mastecto Radiothe

Sample

Size: 219

**************************************************************************

OUTCOME VARIABLE:

CopPosit

Model Summary

R R-sq MSE F df1 df2 p

.33083 .10945 .80153 2.55639 10.00000 208.00000 .00623

Model

coeff se t p LLCI ULCI

constant 3.43955 .53168 6.46918 .00000 2.39137 4.48773

Stigma_I -.04736 .01300 -3.64170 .00034 -.07299 -.02172

Age_cov -.00942 .00691 -1.36268 .17446 -.02305 .00421

Educatio -.04722 .18338 -.25752 .79703 -.40875 .31430

Employme .10310 .19421 .53086 .59608 -.27977 .48596

Social_p -.02313 .04283 -.54008 .58972 -.10756 .06130

Insuranc .01560 .21558 .07238 .94237 -.40939 .44060

Major_Ps -.06339 .14611 -.43382 .66487 -.35143 .22466

Chemo_co -.03581 .09155 -.39120 .69605 -.21630 .14467

Mastecto -.01352 .08634 -.15663 .87569 -.18374 .15669

Radiothe .25174 .15646 1.60896 .10914 -.05671 .56018

Standardized coefficients

coeff

Stigma_I -.25100

Age_cov -.09232

Educatio -.02011

Employme .04178

Social_p -.03606

Insuranc .00486

Major_Ps -.02917

Chemo_co -.03285

Mastecto -.01172

Radiothe .13323

| **Benjamini-Hochberg Procedure** | | | | |
| --- | --- | --- | --- | --- |
|  |  | **Ascending P-value s** | **I = ranking** | **(I/10)* 0.10** |
|  | **Stigma** | **.0003** | **1** | **.01** |
|  | **Radiotherapy** | **~~.109~~** | **2** | **.02** |
|  | **Age** | **~~.174~~** | **3** | **.03** |
|  | **Social participation frequency** | **~~.589~~** | **4** | **.04** |
|  | **Employment** | **~~.596~~** | **5** | **.05** |
|  | **Major Psychological issues** | **~~.664~~** | **6** | **.06** |
|  | **Chemotherapy** | **~~.696~~** | **7** | **.07** |
|  | **Education** | **~~.797~~** | **8** | **.08** |
|  | **Mastectomy** | **~~.875~~** | **9** | **.09** |
|  | **Insurance** | **~~.942~~** | **10** | **.10** |

**************************************************************************

OUTCOME VARIABLE:

FACT_G

Model Summary

R R-sq MSE F df1 df2 p

.58001 .33641 189.31629 9.54000 11.00000 207.00000 .00000

Model

coeff se t p LLCI ULCI

constant 84.16628 8.95563 9.39815 .00000 66.51034 101.82222

Stigma_I -1.33200 .20613 -6.46197 .00000 -1.73838 -.92562

CopPosit 4.02570 1.06562 3.77779 .00021 1.92483 6.12656

Age_cov -.06092 .10671 -.57091 .56868 -.27129 .14945

Educatio -3.86425 2.81876 -1.37091 .17189 -9.42141 1.69291

Employme -3.70171 2.98670 -1.23940 .21660 -9.58996 2.18654

Social_p 1.58171 .65866 2.40142 .01722 .28318 2.88025

Insuranc 3.37971 3.31315 1.02009 .30888 -3.15213 9.91156

Major_Ps -5.34014 2.24653 -2.37706 .01836 -9.76916 -.91112

Chemo_co -1.69633 1.40750 -1.20521 .22950 -4.47119 1.07854

Mastecto -1.33358 1.32704 -1.00493 .31610 -3.94983 1.28266

Radiothe -1.40777 2.41947 -.58185 .56130 -6.17774 3.36221

Standardized coefficients

coeff

Stigma_I -.39749

CopPosit .22666

Age_cov -.03362

Educatio -.09265

Employme -.08447

Social_p .13883

Insuranc .05929

Major_Ps -.13838

Chemo_co -.08762

Mastecto -.06508

Radiothe -.04195

| **Benjamini-Hochberg Procedure** | | | | |
| --- | --- | --- | --- | --- |
|  |  | **Ascending P-values** | **I = ranking** | **(I/11)* 0.10** |
|  | **Stigma** | **.00000** | **1** | **.009** |
|  | **Positive reframing** | **.00021** | **2** | **.01818** |
|  | **Social participation frequency** | **.017** | **3** | **.02727** |
|  | **Major Psychological issues** | **.018** | **4** | **.03636** |
|  | **Education** | **~~.171~~** | **5** | **.045** |
|  | **Employment** | **~~.216~~** | **6** | **.054** |
|  | **Chemotherapy** | **~~.229~~** | **7** | **.063** |
|  | **Mastectomy** | **~~.316~~** | **8** | **.072** |
|  | **Insurance** | **~~.308~~** | **9** | **.081** |
|  | **Radiotherapy** | **~~.561~~** | **10** | **.0909** |
|  | **Age** | **~~.568~~** | **11** | **.100** |

************************** TOTAL EFFECT MODEL ****************************

OUTCOME VARIABLE:

FACT_G

Model Summary

R R-sq MSE F df1 df2 p

.53913 .29066 201.39584 8.52301 10.00000 208.00000 .00000

Model

coeff se t p LLCI ULCI

constant 98.01288 8.42789 11.62958 .00000 81.39783 114.62792

Stigma_I -1.52264 .20613 -7.38669 .00000 -1.92902 -1.11626

Age_cov -.09884 .10957 -.90206 .36807 -.31485 .11717

Educatio -4.05436 2.90683 -1.39477 .16457 -9.78499 1.67627

Employme -3.28667 3.07843 -1.06765 .28692 -9.35559 2.78225

Social_p 1.48860 .67887 2.19276 .02943 .15025 2.82695

Insuranc 3.44253 3.41717 1.00742 .31490 -3.29421 10.17926

Major_Ps -5.59531 2.31605 -2.41589 .01656 -10.16125 -1.02937

Chemo_co -1.84050 1.45117 -1.26829 .20611 -4.70139 1.02039

Mastecto -1.38803 1.36864 -1.01416 .31168 -4.08621 1.31016

Radiothe -.39435 2.48008 -.15901 .87382 -5.28368 4.49497

Standardized coefficients

coeff

Stigma_I -.45439

Age_cov -.05454

Educatio -.09720

Employme -.07500

Social_p .13066

Insuranc .06039

Major_Ps -.14499

Chemo_co -.09506

Mastecto -.06773

Radiothe -.01175

| **Benjamini-Hochberg Procedure** | | | | |
| --- | --- | --- | --- | --- |
|  |  | **Ascending P-value s** | **I = ranking** | **(I/10)* 0.10** |
|  | **Stigma** | **.00000** | **1** | **.01** |
|  | **Major Psychological issues** | **.016** | **2** | **.02** |
|  | **Social participation frequency** | **.029** | **3** | **.03** |
|  | **Education** | **~~.164~~** | **4** | **.04** |
|  | **Chemotherapy** | **~~.206~~** | **5** | **.05** |
|  | **Employment** | **~~.286~~** | **6** | **.06** |
|  | **Mastectomy** | **~~.311~~** | **7** | **.07** |
|  | **Insurance** | **~~.314~~** | **8** | **.08** |
|  | **Age** | **~~.368~~** | **9** | **.09** |
|  | **Radiotherapy** | **~~.873~~** | **10** | **.10** |

************** TOTAL, DIRECT, AND INDIRECT EFFECTS OF X ON Y **************

Total effect of X on Y

Effect se t p LLCI ULCI c_ps c_cs

-1.52264 .20613 -7.38669 .00000 -1.92902 -1.11626 -.09251 -.45439

Direct effect of X on Y

Effect se t p LLCI ULCI c'_ps c'_cs

-1.33200 .20613 -6.46197 .00000 -1.73838 -.92562 -.08093 -.39749

Indirect effect(s) of X on Y:

Effect BootSE BootLLCI BootULCI

CopPosit -.19065 .07071 -.33739 -.06206

Partially standardized indirect effect(s) of X on Y:

Effect BootSE BootLLCI BootULCI

CopPosit -.01158 .00424 -.02035 -.00381

Completely standardized indirect effect(s) of X on Y:

Effect BootSE BootLLCI BootULCI

CopPosit -.05689 .02120 -.10075 -.01827

*********************** ANALYSIS NOTES AND ERRORS ************************

Level of confidence for all confidence intervals in output:

95.0000

Number of bootstrap samples for percentile bootstrap confidence intervals:

5000

***************** PROCESS Procedure for SPSS Version 3.5 *****************

Written by Andrew F. Hayes, Ph.D. www.afhayes.com

Documentation available in Hayes (2018). www.guilford.com/p/hayes3

**************************************************************************

**Model : 4**

**Y : FACT_G**

**X : Stigma_I**

**M : Active coping**

Covariates:

Age_cov Educatio Employme Social_p Insuranc Major_Ps Chemo_co Mastecto Radiothe

Sample

Size: 221

**************************************************************************

OUTCOME VARIABLE:

Activeco

Model Summary

R R-sq MSE F df1 df2 p

.26036 .06779 .67205 1.52706 10.00000 210.00000 .13138

Model

coeff se t p LLCI ULCI

constant 2.93761 .48630 6.04078 .00000 1.97896 3.89626

Stigma_I -.03310 .01179 -2.80723 .00547 -.05634 -.00986

Age_cov .00261 .00626 .41677 .67727 -.00973 .01495

Educatio -.12851 .16785 -.76565 .44474 -.45939 .20237

Employme .10211 .17778 .57436 .56634 -.24835 .45257

Social_p .03567 .03914 .91135 .36316 -.04148 .11282

Insuranc .14093 .19735 .71410 .47596 -.24811 .52997

Major_Ps -.10039 .13284 -.75575 .45064 -.36226 .16147

Chemo_co .02878 .08364 .34409 .73112 -.13610 .19366

Mastecto .13340 .07884 1.69201 .09213 -.02202 .28881

Radiothe -.07485 .14300 -.52343 .60123 -.35676 .20706

Standardized coefficients

coeff

Stigma_I -.19642

Age_cov .02871

Educatio -.06092

Employme .04606

Social_p .06194

Insuranc .04885

Major_Ps -.05179

Chemo_co .02954

Mastecto .12948

Radiothe -.04427

| **Benjamini-Hochberg Procedure** | | | | |
| --- | --- | --- | --- | --- |
|  |  | **Ascending P-value s** | **I = ranking** | **(I/10)* 0.10** |
|  | **Stigma** | **.005** | **1** | **.01** |
|  | **Mastectomy** | **~~.092~~** | **2** | **.02** |
|  | **Social participation frequency** | **~~.363~~** | **3** | **.03** |
|  | **Education** | **~~.444~~** | **4** | **.04** |
|  | **Major Psychological issues** | **~~.450~~** | **5** | **.05** |
|  | **Insurance** | **~~.475~~** | **6** | **.06** |
|  | **Employment** | **~~.566~~** | **7** | **.07** |
|  | **Radiotherapy** | **~~.601~~** | **8** | **.08** |
|  | **Age** | **~~.677~~** | **9** | **.09** |
|  | **Chemotherapy** | **~~.731~~** | **10** | **.10** |

**************************************************************************

OUTCOME VARIABLE:

FACT_G

Model Summary

R R-sq MSE F df1 df2 p

.55750 .31081 195.49598 8.56850 11.00000 209.00000 .00000

Model

coeff se t p LLCI ULCI

constant 89.85315 8.98586 9.99939 .00000 72.13860 107.56769

Stigma_I -1.41765 .20482 -6.92147 .00000 -1.82142 -1.01387

Activeco 2.77098 1.17695 2.35437 .01948 .45076 5.09119

Age_cov -.11339 .10682 -1.06148 .28970 -.32398 .09720

Educatio -3.71766 2.86672 -1.29683 .19612 -9.36906 1.93374

Employme -3.59702 3.03454 -1.18536 .23722 -9.57925 2.38521

Social_p 1.40313 .66883 2.09790 .03712 .08462 2.72164

Insuranc 3.04556 3.37000 .90373 .36718 -3.59798 9.68911

Major_Ps -5.21955 2.26869 -2.30069 .02240 -9.69201 -.74710

Chemo_co -1.87149 1.42694 -1.31154 .19112 -4.68454 .94156

Mastecto -1.79741 1.35377 -1.32770 .18572 -4.46621 .87139

Radiothe -.11204 2.44061 -.04591 .96343 -4.92341 4.69933

Standardized coefficients

coeff

Stigma_I -.42516

Activeco .14003

Age_cov -.06305

Educatio -.08905

Employme -.08199

Social_p .12314

Insuranc .05335

Major_Ps -.13607

Chemo_co -.09709

Mastecto -.08816

Radiothe -.00335

| **Benjamini-Hochberg Procedure** | | | | |
| --- | --- | --- | --- | --- |
|  |  | **Ascending P-values** | **I = ranking** | **(I/11)* 0.10** |
|  | **Stigma** | **.00000** | **1** | **.009** |
|  | **Ative coping** | **.019** | **2** | **.01818** |
|  | **Major Psychological issues** | **.022** | **3** | **.02727** |
|  | **Social participation frequency** | **~~.037~~** | **4** | **.03636** |
|  | **Mastectomy** | **~~.185~~** | **5** | **.045** |
|  | **Chemotherapy** | **~~.191~~** | **6** | **.054** |
|  | **Education** | **~~.196~~** | **7** | **.063** |
|  | **Employment** | **~~.237~~** | **8** | **.072** |
|  | **Age** | **~~.289~~** | **9** | **.081** |
|  | **Insurance** | **~~.367~~** | **10** | **.0909** |
|  | **Radiotherapy** | **~~.963~~** | **11** | **.100** |

************************** TOTAL EFFECT MODEL ****************************

OUTCOME VARIABLE:

FACT_G

Model Summary

R R-sq MSE F df1 df2 p

.54086 .29253 199.72527 8.68320 10.00000 210.00000 .00000

Model

coeff se t p LLCI ULCI

constant 97.99319 8.38332 11.68906 .00000 81.46693 114.51946

Stigma_I -1.50936 .20324 -7.42632 .00000 -1.91002 -1.10870

Age_cov -.10616 .10793 -.98363 .32643 -.31892 .10660

Educatio -4.07377 2.89353 -1.40789 .16064 -9.77786 1.63033

Employme -3.31407 3.06478 -1.08134 .28079 -9.35576 2.72761

Social_p 1.50196 .67469 2.22616 .02707 .17193 2.83200

Insuranc 3.43607 3.40212 1.00998 .31367 -3.27063 10.14276

Major_Ps -5.49774 2.28999 -2.40077 .01723 -10.01205 -.98342

Chemo_co -1.79174 1.44189 -1.24263 .21539 -4.63418 1.05069

Mastecto -1.42777 1.35910 -1.05052 .29468 -4.10701 1.25146

Radiothe -.31945 2.46526 -.12958 .89702 -5.17928 4.54038

Standardized coefficients

coeff

Stigma_I -.45267

Age_cov -.05903

Educatio -.09758

Employme -.07554

Social_p .13181

Insuranc .06019

Major_Ps -.14332

Chemo_co -.09295

Mastecto -.07003

Radiothe -.00955

| **Benjamini-Hochberg Procedure** | | | | |
| --- | --- | --- | --- | --- |
|  |  | **Ascending P-value s** | **I = ranking** | **(I/10)* 0.10** |
|  | **Stigma** | **.00000** | **1** | **.01** |
|  | **Major Psychological issues** | **.017** | **2** | **.02** |
|  | **Social participation frequency** | **.027** | **3** | **.03** |
|  | **Education** | **~~.160~~** | **4** | **.04** |
|  | **Chemotherapy** | **~~.215~~** | **5** | **.05** |
|  | **Mastectomy** | **~~.294~~** | **6** | **.06** |
|  | **Employment** | **~~.280~~** | **7** | **.07** |
|  | **Insurance** | **~~.313~~** | **8** | **.08** |
|  | **Age** | **~~.326~~** | **9** | **.09** |
|  | **Radiotherapy** | **~~.897~~** | **10** | **.10** |

************** TOTAL, DIRECT, AND INDIRECT EFFECTS OF X ON Y **************

Total effect of X on Y

Effect se t p LLCI ULCI c_ps c_cs

-1.50936 .20324 -7.42632 .00000 -1.91002 -1.10870 -.09195 -.45267

Direct effect of X on Y

Effect se t p LLCI ULCI c'_ps c'_cs

-1.41765 .20482 -6.92147 .00000 -1.82142 -1.01387 -.08636 -.42516

Indirect effect(s) of X on Y:

Effect BootSE BootLLCI BootULCI

Activeco -.09171 .05321 -.21097 -.00965

Partially standardized indirect effect(s) of X on Y:

Effect BootSE BootLLCI BootULCI

Activeco -.00559 .00322 -.01270 -.00060

Completely standardized indirect effect(s) of X on Y:

Effect BootSE BootLLCI BootULCI

Activeco -.02750 .01586 -.06297 -.00282

*********************** ANALYSIS NOTES AND ERRORS ************************

Level of confidence for all confidence intervals in output:

95.0000

Number of bootstrap samples for percentile bootstrap confidence intervals:

5000

***************** PROCESS Procedure for SPSS Version 3.5 *****************

Written by Andrew F. Hayes, Ph.D. www.afhayes.com

Documentation available in Hayes (2018). www.guilford.com/p/hayes3

**************************************************************************

**Model : 4**

**Y : FACT_G**

**X : Stigma_I**

**M : copAccep**

Covariates:

Age_cov Educatio Employme Social_p Insuranc Major_Ps Chemo_co Mastecto Radiothe

Sample

Size: 220

**************************************************************************

OUTCOME VARIABLE:

copAccep

Model Summary

R R-sq MSE F df1 df2 p

.32195 .10365 .05680 2.41687 10.00000 209.00000 .00968

Model

coeff se t p LLCI ULCI

constant .51797 .14152 3.65996 .00032 .23897 .79696

Stigma_I -.00740 .00345 -2.14869 .03281 -.01420 -.00061

Age_cov .00317 .00182 1.74118 .08312 -.00042 .00676

Educatio -.03797 .04880 -.77799 .43745 -.13418 .05824

Employme .08544 .05170 1.65274 .09988 -.01647 .18735

Social_p -.01322 .01138 -1.16155 .24675 -.03566 .00922

Insuranc .01042 .05739 .18149 .85616 -.10271 .12354

Major_Ps .00045 .03885 .01156 .99079 -.07615 .07705

Chemo_co -.04830 .02433 -1.98533 .04842 -.09627 -.00034

Mastecto -.04955 .02297 -2.15714 .03214 -.09483 -.00427

Radiothe .05223 .04159 1.25566 .21064 -.02977 .13423

Standardized coefficients

coeff

Stigma_I -.14785

Age_cov .11782

Educatio -.06082

Employme .13025

Social_p -.07757

Insuranc .01220

Major_Ps .00078

Chemo_co -.16717

Mastecto -.16199

Radiothe .10428

| **Benjamini-Hochberg Procedure** | | | | |
| --- | --- | --- | --- | --- |
|  |  | **Ascending P-value s** | **I = ranking** | **(I/10)* 0.10** |
|  | **Mastectomy** | **~~.0321~~** | **1** | **.01** |
|  | **Stigma** | **~~.0328~~** | **2** | **.02** |
|  | **Chemotherapy** | **~~.048~~** | **3** | **.03** |
|  | **Age** | **~~.083~~** | **4** | **.04** |
|  | **Employment** | **~~.099~~** | **5** | **.05** |
|  | **Radiotherapy** | **~~.210~~** | **6** | **.06** |
|  | **Social participation frequency** | **~~.246~~** | **7** | **.07** |
|  | **Education** | **~~.437~~** | **8** | **.08** |
|  | **Insurance** | **~~.856~~** | **9** | **.09** |
|  | **Major Psychological issues** | **~~.990~~** | **10** | **.10** |

**************************************************************************

OUTCOME VARIABLE:

FACT_G

Model Summary

R R-sq MSE F df1 df2 p

.56362 .31766 193.73354 8.80312 11.00000 208.00000 .00000

Model

coeff se t p LLCI ULCI

constant 91.97925 8.52606 10.78801 .00000 75.17067 108.78784

Stigma_I -1.42629 .20345 -7.01055 .00000 -1.82738 -1.02520

copAccep 11.74937 4.03979 2.90841 .00403 3.78518 19.71356

Age_cov -.14367 .10707 -1.34179 .18113 -.35476 .06742

Educatio -3.63806 2.85445 -1.27452 .20390 -9.26543 1.98931

Employme -4.30587 3.03880 -1.41697 .15799 -10.29666 1.68492

Social_p 1.66142 .66699 2.49094 .01352 .34650 2.97634

Insuranc 3.32755 3.35176 .99277 .32197 -3.28024 9.93533

Major_Ps -5.55040 2.26918 -2.44600 .01528 -10.02394 -1.07687

Chemo_co -1.23346 1.43427 -.85999 .39078 -4.06103 1.59411

Mastecto -.82873 1.35636 -.61099 .54187 -3.50271 1.84526

Radiothe -.94782 2.43839 -.38871 .69789 -5.75494 3.85930

Standardized coefficients

coeff

Stigma_I -.42654

copAccep .17595

Age_cov -.07998

Educatio -.08727

Employme -.09830

Social_p .14596

Insuranc .05839

Major_Ps -.14392

Chemo_co -.06393

Mastecto -.04057

Radiothe -.02834

| **Benjamini-Hochberg Procedure** | | | | |
| --- | --- | --- | --- | --- |
|  |  | **Ascending P-values** | **I = ranking** | **(I/11)* 0.10** |
|  | **Stigma** | **.00000** | **1** | **.009** |
|  | **Acceptance** | **.004** | **2** | **.01818** |
|  | **Social participation frequency** | **.013** | **3** | **.02727** |
|  | **Major Psychological issues** | **.015** | **4** | **.03636** |
|  | **Age** | **~~.181~~** | **5** | **.045** |
|  | **Employment** | **~~.157~~** | **6** | **.054** |
|  | **Education** | **~~.203~~** | **7** | **.063** |
|  | **Insurance** | **~~.321~~** | **8** | **.072** |
|  | **Chemotherapy** | **~~.390~~** | **9** | **.081** |
|  | **Mastectomy** | **~~.541~~** | **10** | **.0909** |
|  | **Radiotherapy** | **~~.697~~** | **11** | **.100** |

************************** TOTAL EFFECT MODEL ****************************

OUTCOME VARIABLE:

FACT_G

Model Summary

R R-sq MSE F df1 df2 p

.53844 .28991 200.64754 8.53302 10.00000 209.00000 .00000

Model

coeff se t p LLCI ULCI

constant 98.06505 8.41150 11.65845 .00000 81.48279 114.64732

Stigma_I -1.51328 .20480 -7.38914 .00000 -1.91701 -1.10955

Age_cov -.10643 .10819 -.98380 .32635 -.31971 .10684

Educatio -4.08418 2.90074 -1.40798 .16062 -9.80265 1.63428

Employme -3.30202 3.07253 -1.07469 .28375 -9.35915 2.75511

Social_p 1.50606 .67660 2.22592 .02709 .17222 2.83991

Insuranc 3.44992 3.41078 1.01147 .31296 -3.27403 10.17386

Major_Ps -5.54513 2.30931 -2.40120 .01722 -10.09766 -.99259

Chemo_co -1.80099 1.44607 -1.24544 .21436 -4.65174 1.04976

Mastecto -1.41091 1.36524 -1.03345 .30259 -4.10232 1.28050

Radiothe -.33417 2.47221 -.13517 .89261 -5.20783 4.53949

Standardized coefficients

coeff

Stigma_I -.45255

Age_cov -.05925

Educatio -.09797

Employme -.07538

Social_p .13231

Insuranc .06053

Major_Ps -.14379

Chemo_co -.09334

Mastecto -.06907

Radiothe -.00999

| **Benjamini-Hochberg Procedure** | | | | |
| --- | --- | --- | --- | --- |
|  |  | **Ascending P-value s** | **I = ranking** | **(I/10)* 0.10** |
|  | **Stigma** | **.00000** | **1** | **.01** |
|  | **Major Psychological issues** | **.017** | **2** | **.02** |
|  | **Social participation frequency** | **.027** | **3** | **.03** |
|  | **Education** | **~~.160~~** | **4** | **.04** |
|  | **Chemotherapy** | **~~.214~~** | **5** | **.05** |
|  | **Employment** | **~~.283~~** | **6** | **.06** |
|  | **Mastectomy** | **~~.302~~** | **7** | **.07** |
|  | **Insurance** | **~~.312~~** | **8** | **.08** |
|  | **Age** | **~~.318~~** | **9** | **.09** |
|  | **Radiotherapy** | **~~.892~~** | **10** | **.10** |

************** TOTAL, DIRECT, AND INDIRECT EFFECTS OF X ON Y **************

Total effect of X on Y

Effect se t p LLCI ULCI c_ps c_cs

-1.51328 .20480 -7.38914 .00000 -1.91701 -1.10955 -.09215 -.45255

Direct effect of X on Y

Effect se t p LLCI ULCI c'_ps c'_cs

-1.42629 .20345 -7.01055 .00000 -1.82738 -1.02520 -.08686 -.42654

Indirect effect(s) of X on Y:

Effect BootSE BootLLCI BootULCI

copAccep -.08699 .04879 -.19252 -.00356

Partially standardized indirect effect(s) of X on Y:

Effect BootSE BootLLCI BootULCI

copAccep -.00530 .00295 -.01169 -.00024

Completely standardized indirect effect(s) of X on Y:

Effect BootSE BootLLCI BootULCI

copAccep -.02601 .01444 -.05749 -.00107

*********************** ANALYSIS NOTES AND ERRORS ************************

Level of confidence for all confidence intervals in output:

95.0000

Number of bootstrap samples for percentile bootstrap confidence intervals:

5000

***************** PROCESS Procedure for SPSS Version 3.5 *****************

Written by Andrew F. Hayes, Ph.D. www.afhayes.com

Documentation available in Hayes (2018). www.guilford.com/p/hayes3

**************************************************************************

**Model : 4**

**Y : FACT_G**

**X : Stigma_I**

**M1 : Meaningf**

**M2 : MOS_SSS**

**M3 : CopPosit**

Covariates:

Age_cov Educatio Employme Social_p Insuranc Major_Ps Chemo_co Mastecto Radiothe

Sample

Size: 219

**************************************************************************

OUTCOME VARIABLE:

Meaningf

Model Summary

R R-sq MSE F df1 df2 p

.43827 .19208 20.04765 4.94515 10.00000 208.00000 .00000

Model

coeff se t p LLCI ULCI

constant 21.99116 2.65904 8.27033 .00000 16.74902 27.23329

Stigma_I -.24362 .06504 -3.74594 .00023 -.37184 -.11541

Age_cov .06993 .03457 2.02298 .04436 .00178 .13809

Educatio -1.54766 .91712 -1.68752 .09300 -3.35570 .26039

Employme .61881 .97126 .63712 .52475 -1.29597 2.53358

Social_p .50570 .21419 2.36102 .01915 .08344 .92795

Insuranc .00323 1.07813 .00300 .99761 -2.12224 2.12870

Major_Ps -1.36998 .73073 -1.87482 .06222 -2.81056 .07060

Chemo_co -.31410 .45785 -.68604 .49345 -1.21673 .58852

Mastecto -.82346 .43181 -1.90699 .05790 -1.67475 .02783

Radiothe .54173 .78248 .69232 .48951 -1.00088 2.08433

Standardized coefficients

coeff

Stigma_I -.24592

Age_cov .13054

Educatio -.12551

Employme .04776

Social_p .15015

Insuranc .00019

Major_Ps -.12008

Chemo_co -.05488

Mastecto -.13593

Radiothe .05460

| **Benjamini-Hochberg Procedure** | | | | |
| --- | --- | --- | --- | --- |
|  |  | **Ascending P-value s** | **I = ranking** | **(I/10)* 0.10** |
|  | **Stigma** | **.0002** | **1** | **.01** |
|  | **Social participation frequency** | **.019** | **2** | **.02** |
|  | **Age** | **~~.044~~** | **3** | **.03** |
|  | **Mastectomy** | **~~.057~~** | **4** | **.04** |
|  | **Major Psychological issues** | **~~.062~~** | **5** | **.05** |
|  | **Education** | **~~.093~~** | **6** | **.06** |
|  | **Radiotherapy** | **~~.489~~** | **7** | **.07** |
|  | **Chemotherapy** | **~~.493~~** | **8** | **.08** |
|  | **Employment** | **~~.524~~** | **9** | **.09** |
|  | **Insurance** | **~~.997~~** | **10** | **.10** |

**************************************************************************

OUTCOME VARIABLE:

MOS_SSS

Model Summary

R R-sq MSE F df1 df2 p

.34356 .11803 .06736 2.78361 10.00000 208.00000 .00299

Model

coeff se t p LLCI ULCI

constant .45133 .15413 2.92819 .00379 .14747 .75519

Stigma_I -.00955 .00377 -2.53373 .01202 -.01698 -.00212

Age_cov .00133 .00200 .66567 .50636 -.00262 .00528

Educatio -.03675 .05316 -.69133 .49013 -.14156 .06805

Employme -.02766 .05630 -.49132 .62372 -.13865 .08333

Social_p -.00085 .01242 -.06857 .94540 -.02533 .02362

Insuranc .07432 .06249 1.18918 .23572 -.04889 .19752

Major_Ps -.10845 .04236 -2.56037 .01117 -.19195 -.02495

Chemo_co .00229 .02654 .08621 .93138 -.05003 .05461

Mastecto -.02110 .02503 -.84289 .40026 -.07044 .02825

Radiothe .06329 .04536 1.39536 .16440 -.02613 .15271

Standardized coefficients

coeff

Stigma_I -.17379

Age_cov .04488

Educatio -.05372

Employme -.03848

Social_p -.00456

Insuranc .07949

Major_Ps -.17134

Chemo_co .00721

Mastecto -.06277

Radiothe .11499

| **Benjamini-Hochberg Procedure** | | | | |
| --- | --- | --- | --- | --- |
|  |  | **Ascending P-value s** | **I = ranking** | **(I/10)* 0.10** |
|  | **Stigma** | **.012** | **1** | **.01** |
|  | **Major Psychological issues** | **.011** | **2** | **.02** |
|  | **Radiotherapy** | **~~.164~~** | **3** | **.03** |
|  | **Insurance** | **~~.235~~** | **4** | **.04** |
|  | **Mastectomy** | **~~.400~~** | **5** | **.05** |
|  | **Education** | **~~.490~~** | **6** | **.06** |
|  | **Age** | **~~.506~~** | **7** | **.07** |
|  | **Employment** | **~~.623~~** | **8** | **.08** |
|  | **Chemotherapy** | **~~.931~~** | **9** | **.09** |
|  | **Social participation frequency** | **~~.945~~** | **10** | **.10** |

**************************************************************************

OUTCOME VARIABLE:

CopPosit

Model Summary

R R-sq MSE F df1 df2 p

.33083 .10945 .80153 2.55639 10.00000 208.00000 .00623

Model

coeff se t p LLCI ULCI

constant 3.43955 .53168 6.46918 .00000 2.39137 4.48773

Stigma_I -.04736 .01300 -3.64170 .00034 -.07299 -.02172

Age_cov -.00942 .00691 -1.36268 .17446 -.02305 .00421

Educatio -.04722 .18338 -.25752 .79703 -.40875 .31430

Employme .10310 .19421 .53086 .59608 -.27977 .48596

Social_p -.02313 .04283 -.54008 .58972 -.10756 .06130

Insuranc .01560 .21558 .07238 .94237 -.40939 .44060

Major_Ps -.06339 .14611 -.43382 .66487 -.35143 .22466

Chemo_co -.03581 .09155 -.39120 .69605 -.21630 .14467

Mastecto -.01352 .08634 -.15663 .87569 -.18374 .15669

Radiothe .25174 .15646 1.60896 .10914 -.05671 .56018

Standardized coefficients

coeff

Stigma_I -.25100

Age_cov -.09232

Educatio -.02011

Employme .04178

Social_p -.03606

Insuranc .00486

Major_Ps -.02917

Chemo_co -.03285

Mastecto -.01172

Radiothe .13323

| **Benjamini-Hochberg Procedure** | | | | |
| --- | --- | --- | --- | --- |
|  |  | **Ascending P-value s** | **I = ranking** | **(I/10)* 0.10** |
|  | **Stigma** | **.0003** | **1** | **.01** |
|  | **Radiotherapy** | **~~.109~~** | **2** | **.02** |
|  | **Age** | **~~.174~~** | **3** | **.03** |
|  | **Social participation frequency** | **~~.589~~** | **4** | **.04** |
|  | **Employment** | **~~.596~~** | **5** | **.05** |
|  | **Major Psychological issues** | **~~.664~~** | **6** | **.06** |
|  | **Chemotherapy** | **~~.696~~** | **7** | **.07** |
|  | **Education** | **~~.797~~** | **8** | **.08** |
|  | **Mastectomy** | **~~.875~~** | **9** | **.09** |
|  | **Insurance** | **~~.942~~** | **10** | **.10** |

**************************************************************************

OUTCOME VARIABLE:

FACT_G

Model Summary

R R-sq MSE F df1 df2 p

.63900 .40832 170.44747 10.88248 13.00000 205.00000 .00000

Model

coeff se t p LLCI ULCI

constant 68.26252 9.26591 7.36706 .00000 49.99382 86.53123

Stigma_I -1.12909 .19987 -5.64910 .00000 -1.52315 -.73502

Meaningf .78507 .21439 3.66191 .00032 .36238 1.20776

MOS_SSS 9.10342 3.68037 2.47351 .01419 1.84719 16.35966

CopPosit 2.43551 1.06060 2.29636 .02267 .34443 4.52658

Age_cov -.14295 .10273 -1.39148 .16559 -.34549 .05960

Educatio -2.38976 2.69309 -.88737 .37592 -7.69947 2.91996

Employme -3.77176 2.83982 -1.32817 .18560 -9.37076 1.82723

Social_p 1.15567 .63530 1.81911 .07035 -.09688 2.40822

Insuranc 2.72545 3.15532 .86376 .38873 -3.49560 8.94650

Major_Ps -3.37815 2.17243 -1.55501 .12149 -7.66131 .90502

Chemo_co -1.52751 1.33704 -1.14246 .25460 -4.16363 1.10860

Mastecto -.51655 1.27087 -.40645 .68483 -3.02220 1.98910

Radiothe -2.00890 2.30179 -.87275 .38382 -6.54712 2.52932

Standardized coefficients

coeff

Stigma_I -.33694

Meaningf .23209

MOS_SSS .14931

CopPosit .13713

Age_cov -.07888

Educatio -.05729

Employme -.08607

Social_p .10144

Insuranc .04781

Major_Ps -.08754

Chemo_co -.07890

Mastecto -.02521

Radiothe -.05986

| **Benjamini-Hochberg Procedure** | | | | |
| --- | --- | --- | --- | --- |
|  |  | **Ascending P-value s** | **I = ranking** | **(I/13) * 0.10** |
|  | **Stigma** | **.00000** | **1** | **.0076** |
|  | **Meaningfulness** | **.0003** | **2** | **.0153** |
|  | **Total social support** | **.014** | **3** | **.023** |
|  | **Positive reframing** | **.022** | **4** | **.030** |
|  | **Social participation frequency** | **~~.07~~** | **5** | **.038** |
|  | **Major Psychological issues** | **~~.121~~** | **6** | **.046** |
|  | **Age** | **~~.165~~** | **7** | **.053** |
|  | **Employment** | **~~.185~~** | **8** | **.061** |
|  | **Chemotherapy** | **~~.254~~** | **9** | **.069** |
|  | **Education** | **~~.375~~** | **10** | **.076** |
|  | **Radiotherapy** | **~~.383~~** | **11** | **.084** |
|  | **Insurance** | **~~.388~~** | **12** | **.092** |
|  | **Mastectomy** | **~~.684~~** | **13** | **.10** |

************************** TOTAL EFFECT MODEL ****************************

OUTCOME VARIABLE:

FACT_G

Model Summary

R R-sq MSE F df1 df2 p

.53913 .29066 201.39584 8.52301 10.00000 208.00000 .00000

Model

coeff se t p LLCI ULCI

constant 98.01288 8.42789 11.62958 .00000 81.39783 114.62792

Stigma_I -1.52264 .20613 -7.38669 .00000 -1.92902 -1.11626

Age_cov -.09884 .10957 -.90206 .36807 -.31485 .11717

Educatio -4.05436 2.90683 -1.39477 .16457 -9.78499 1.67627

Employme -3.28667 3.07843 -1.06765 .28692 -9.35559 2.78225

Social_p 1.48860 .67887 2.19276 .02943 .15025 2.82695

Insuranc 3.44253 3.41717 1.00742 .31490 -3.29421 10.17926

Major_Ps -5.59531 2.31605 -2.41589 .01656 -10.16125 -1.02937

Chemo_co -1.84050 1.45117 -1.26829 .20611 -4.70139 1.02039

Mastecto -1.38803 1.36864 -1.01416 .31168 -4.08621 1.31016

Radiothe -.39435 2.48008 -.15901 .87382 -5.28368 4.49497

Standardized coefficients

coeff

Stigma_I -.45439

Age_cov -.05454

Educatio -.09720

Employme -.07500

Social_p .13066

Insuranc .06039

Major_Ps -.14499

Chemo_co -.09506

Mastecto -.06773

Radiothe -.01175

| **Benjamini-Hochberg Procedure** | | | | |
| --- | --- | --- | --- | --- |
|  |  | **Ascending P-value s** | **I = ranking** | **(I/10)* 0.10** |
|  | **Stigma** | **.00000** | **1** | **.01** |
|  | **Major Psychological issues** | **.016** | **2** | **.02** |
|  | **Social participation frequency** | **.029** | **3** | **.03** |
|  | **Education** | **~~.164~~** | **4** | **.04** |
|  | **Chemotherapy** | **~~.206~~** | **5** | **.05** |
|  | **Employment** | **~~.286~~** | **6** | **.06** |
|  | **Mastectomy** | **~~.311~~** | **7** | **.07** |
|  | **Insurance** | **~~.314~~** | **8** | **.08** |
|  | **Age** | **~~.368~~** | **9** | **.09** |
|  | **Radiotherapy** | **~~.873~~** | **10** | **.10** |

************** TOTAL, DIRECT, AND INDIRECT EFFECTS OF X ON Y **************

Total effect of X on Y

Effect se t p LLCI ULCI c_ps c_cs

-1.52264 .20613 -7.38669 .00000 -1.92902 -1.11626 -.09251 -.45439

Direct effect of X on Y

Effect se t p LLCI ULCI c'_ps c'_cs

-1.12909 .19987 -5.64910 .00000 -1.52315 -.73502 -.06860 -.33694

Indirect effect(s) of X on Y:

Effect BootSE BootLLCI BootULCI

TOTAL -.39355 .10667 -.61178 -.19195

Meaningf -.19126 .06951 -.34556 -.07050

MOS_SSS -.08695 .04761 -.19272 -.00896

CopPosit -.11534 .05750 -.23572 -.01160

Partially standardized indirect effect(s) of X on Y:

Effect BootSE BootLLCI BootULCI

TOTAL -.02391 .00628 -.03657 -.01189

Meaningf -.01162 .00417 -.02084 -.00439

MOS_SSS -.00528 .00286 -.01156 -.00055

CopPosit -.00701 .00347 -.01424 -.00071

Completely standardized indirect effect(s) of X on Y:

Effect BootSE BootLLCI BootULCI

TOTAL -.11744 .03175 -.18223 -.05725

Meaningf -.05708 .02057 -.10190 -.02142

MOS_SSS -.02595 .01424 -.05745 -.00262

CopPosit -.03442 .01719 -.07020 -.00350

*********************** ANALYSIS NOTES AND ERRORS ************************

Level of confidence for all confidence intervals in output:

95.0000

Number of bootstrap samples for percentile bootstrap confidence intervals:

5000

************** PROCESS Procedure for SPSS Version 3.5 *****************

Written by Andrew F. Hayes, Ph.D. www.afhayes.com

Documentation available in Hayes (2018). www.guilford.com/p/hayes3

**************************************************************************

**Model : 4**

**Y : FACT_B**

**X : Stigma_I**

**M : Comprehensibility**

Covariates:

Age_cov Educatio Employme Social_p Insuranc Major_Ps Chemo_co Mastecto Radiothe

Sample

Size: 221

**************************************************************************

OUTCOME VARIABLE:

Comprehe

Model Summary

R R-sq MSE F df1 df2 p

.38011 .14448 36.53598 3.54648 10.00000 210.00000 .00023

Model

coeff se t p LLCI ULCI

constant 28.41039 3.58559 7.92350 .00000 21.34203 35.47875

Stigma_I -.32272 .08693 -3.71244 .00026 -.49408 -.15135

Age_cov .04775 .04616 1.03439 .30214 -.04325 .13875

Educatio -.31523 1.23758 -.25471 .79919 -2.75489 2.12444

Employme 1.82890 1.31082 1.39523 .16442 -.75515 4.41296

Social_p .36516 .28857 1.26541 .20713 -.20371 .93402

Insuranc -.55790 1.45510 -.38341 .70181 -3.42638 2.31059

Major_Ps -1.89152 .97944 -1.93123 .05480 -3.82232 .03927

Chemo_co -1.16488 .61670 -1.88888 .06029 -2.38060 .05085

Mastecto -1.47027 .58130 -2.52930 .01216 -2.61619 -.32435

Radiothe -1.08926 1.05440 -1.03306 .30277 -3.16783 .98931

Standardized coefficients

coeff

Stigma_I -.24884

Age_cov .06826

Educatio -.01941

Employme .10719

Social_p .08239

Insuranc -.02513

Major_Ps -.12678

Chemo_co -.15537

Mastecto -.18542

Radiothe -.08370

| **Benjamini-Hochberg Procedure** | | | | |
| --- | --- | --- | --- | --- |
|  |  | **Ascending P-value s** | **I = ranking** | **(I/10)* 0.10** |
|  | **Stigma** | **.00026** | **1** | **.01** |
|  | **Mastectomy** | **.012** | **2** | **.02** |
|  | **Major Psychological issues** | **~~.054~~** | **3** | **.03** |
|  | **Chemotherapy** | **~~.060~~** | **4** | **.04** |
|  | **Employment** | **~~.164~~** | **5** | **.05** |
|  | **Social participation frequency** | **~~.207~~** | **6** | **.06** |
|  | **Age** | **~~.3021~~** | **7** | **.07** |
|  | **Radiotherapy** | **~~.3027~~** | **8** | **.08** |
|  | **Insurance** | **~~.701~~** | **9** | **.09** |
|  | **Education** | **~~.799~~** | **10** | **.10** |

**************************************************************************

OUTCOME VARIABLE:

FACT_B

Model Summary

R R-sq MSE F df1 df2 p

.58321 .34013 277.03075 9.79361 11.00000 209.00000 .00000

Model

coeff se t p LLCI ULCI

constant 109.21092 11.25283 9.70519 .00000 87.02731 131.39454

Stigma_I -1.77283 .24710 -7.17462 .00000 -2.25995 -1.28571

Comprehe .52097 .19002 2.74169 .00664 .14637 .89557

Age_cov -.08549 .12743 -.67086 .50305 -.33671 .16573

Educatio -5.48679 3.40834 -1.60981 .10895 -12.20592 1.23234

Employme -5.31375 3.62619 -1.46538 .14432 -12.46235 1.83485

Social_p 1.70241 .79763 2.13434 .03398 .12998 3.27484

Insuranc 5.16101 4.00820 1.28761 .19930 -2.74068 13.06270

Major_Ps -5.48414 2.72085 -2.01560 .04512 -10.84796 -.12032

Chemo_co -1.86969 1.71253 -1.09177 .27619 -5.24574 1.50636

Mastecto -.83762 1.62486 -.51550 .60675 -4.04085 2.36560

Radiothe .18116 2.91079 .06224 .95043 -5.55712 5.91944

Standardized coefficients

coeff

Stigma_I -.43704

Comprehe .16656

Age_cov -.03907

Educatio -.10804

Employme -.09956

Social_p .12281

Insuranc .07431

Major_Ps -.11752

Chemo_co -.07973

Mastecto -.03377

Radiothe .00445

| **Benjamini-Hochberg Procedure** | | | | |
| --- | --- | --- | --- | --- |
|  |  | **Ascending P-values** | **I = ranking** | **(I/11)* 0.10** |
|  | **Stigma** | **.00000** | **1** | **.009** |
|  | **Comprehensibility** | **.006** | **2** | **.01818** |
|  | **Social participation frequency** | **~~.033~~** | **3** | **.02727** |
|  | **Major Psychological issues** | **~~.045~~** | **4** | **.03636** |
|  | **Education** | **~~.108~~** | **5** | **.045** |
|  | **Employment** | **~~.144~~** | **6** | **.054** |
|  | **Insurance** | **~~.199~~** | **7** | **.063** |
|  | **Chemotherapy** | **~~.276~~** | **8** | **.072** |
|  | **Age** | **~~.503~~** | **9** | **.081** |
|  | **Mastectomy** | **~~.606~~** | **10** | **.0909** |
|  | **Radiotherapy** | **~~.950~~** | **11** | **.100** |

************************** TOTAL EFFECT MODEL ****************************

OUTCOME VARIABLE:

FACT_B

Model Summary

R R-sq MSE F df1 df2 p

.56249 .31640 285.62775 9.71966 10.00000 210.00000 .00000

Model

coeff se t p LLCI ULCI

constant 124.01187 10.02536 12.36982 .00000 104.24862 143.77512

Stigma_I -1.94096 .24305 -7.98573 .00000 -2.42009 -1.46182

Age_cov -.06061 .12907 -.46963 .63910 -.31504 .19382

Educatio -5.65102 3.46028 -1.63311 .10394 -12.47236 1.17033

Employme -4.36095 3.66508 -1.18986 .23544 -11.58601 2.86412

Social_p 1.89265 .80684 2.34575 .01992 .30210 3.48319

Insuranc 4.87036 4.06850 1.19709 .23262 -3.14997 12.89070

Major_Ps -6.46957 2.73853 -2.36242 .01907 -11.86810 -1.07103

Chemo_co -2.47656 1.72431 -1.43626 .15242 -5.87574 .92263

Mastecto -1.60359 1.62531 -.98664 .32496 -4.80761 1.60043

Radiothe -.38631 2.94813 -.13104 .89587 -6.19803 5.42541

Standardized coefficients

coeff

Stigma_I -.47848

Age_cov -.02770

Educatio -.11127

Employme -.08171

Social_p .13653

Insuranc .07012

Major_Ps -.13863

Chemo_co -.10561

Mastecto -.06465

Radiothe -.00949

| **Benjamini-Hochberg Procedure** | | | | |
| --- | --- | --- | --- | --- |
|  |  | **Ascending P-value s** | **I = ranking** | **(I/10)* 0.10** |
|  | **Stigma** | **.00000** | **1** | **.01** |
|  | **Major Psychological issues** | **.0190** | **2** | **.02** |
|  | **Social participation frequency** | **.0199** | **3** | **.03** |
|  | **Education** | **~~.103~~** | **4** | **.04** |
|  | **Chemotherapy** | **~~.152~~** | **5** | **.05** |
|  | **Insurance** | **~~.232~~** | **6** | **.06** |
|  | **Employment** | **~~.235~~** | **7** | **.07** |
|  | **Mastectomy** | **~~.324~~** | **8** | **.08** |
|  | **Age** | **~~.639~~** | **9** | **.09** |
|  | **Radiotherapy** | **~~.898~~** | **10** | **.10** |

************** TOTAL, DIRECT, AND INDIRECT EFFECTS OF X ON Y **************

Total effect of X on Y

Effect se t p LLCI ULCI c_ps c_cs

-1.94096 .24305 -7.98573 .00000 -2.42009 -1.46182 -.09719 -.47848

Direct effect of X on Y

Effect se t p LLCI ULCI c'_ps c'_cs

-1.77283 .24710 -7.17462 .00000 -2.25995 -1.28571 -.08877 -.43704

Indirect effect(s) of X on Y:

Effect BootSE BootLLCI BootULCI

Comprehe -.16813 .07502 -.33431 -.04136

Partially standardized indirect effect(s) of X on Y:

Effect BootSE BootLLCI BootULCI

Comprehe -.00842 .00378 -.01670 -.00203

Completely standardized indirect effect(s) of X on Y:

Effect BootSE BootLLCI BootULCI

Comprehe -.04145 .01814 -.08074 -.01015

*********************** ANALYSIS NOTES AND ERRORS ************************

Level of confidence for all confidence intervals in output:

95.0000

Number of bootstrap samples for percentile bootstrap confidence intervals:

5000

***************** PROCESS Procedure for SPSS Version 3.5 *****************

Written by Andrew F. Hayes, Ph.D. www.afhayes.com

Documentation available in Hayes (2018). www.guilford.com/p/hayes3

**************************************************************************

**Model : 4**

**Y : FACT_B**

**X : Stigma_I**

**M : Manageability**

Covariates:

Age_cov Educatio Employme Social_p Insuranc Major_Ps Chemo_co Mastecto Radiothe

Sample

Size: 221

**************************************************************************

OUTCOME VARIABLE:

Manageab

Model Summary

R R-sq MSE F df1 df2 p

.35462 .12576 30.37485 3.02083 10.00000 210.00000 .00137

Model

coeff se t p LLCI ULCI

constant 17.47806 3.26932 5.34609 .00000 11.03318 23.92295

Stigma_I -.26580 .07926 -3.35345 .00095 -.42205 -.10955

Age_cov .06260 .04209 1.48725 .13845 -.02037 .14557

Educatio 1.48418 1.12841 1.31528 .18985 -.74029 3.70865

Employme .90084 1.19520 .75372 .45186 -1.45528 3.25697

Social_p .51544 .26311 1.95900 .05144 -.00324 1.03413

Insuranc -1.09144 1.32676 -.82264 .41165 -3.70690 1.52403

Major_Ps -1.73020 .89305 -1.93742 .05404 -3.49069 .03028

Chemo_co -.05627 .56231 -.10007 .92038 -1.16476 1.05222

Mastecto -.68241 .53002 -1.28751 .19933 -1.72725 .36244

Radiothe .09180 .96140 .09548 .92402 -1.80343 1.98703

Standardized coefficients

coeff

Stigma_I -.22723

Age_cov .09921

Educatio .10134

Employme .05853

Social_p .12894

Insuranc -.05450

Major_Ps -.12857

Chemo_co -.00832

Mastecto -.09541

Radiothe .00782

| **Benjamini-Hochberg Procedure** | | | | |
| --- | --- | --- | --- | --- |
|  |  | **Ascending P-value s** | **I = ranking** | **(I/10)* 0.10** |
|  | **Stigma** | **.0009** | **1** | **.01** |
|  | **Social participation frequency** | **~~.051~~** | **2** | **.02** |
|  | **Major Psychological issues** | **~~.054~~** | **3** | **.03** |
|  | **Age** | **~~.138~~** | **4** | **.04** |
|  | **Education** | **~~.189~~** | **5** | **.05** |
|  | **Mastectomy** | **~~.199~~** | **6** | **.06** |
|  | **Insurance** | **~~.411~~** | **7** | **.07** |
|  | **Employment** | **~~.451~~** | **8** | **.08** |
|  | **Chemotherapy** | **~~.920~~** | **9** | **.09** |
|  | **Radiotherapy** | **~~.924~~** | **10** | **.10** |

**************************************************************************

OUTCOME VARIABLE:

FACT_B

Model Summary

R R-sq MSE F df1 df2 p

.58257 .33939 277.34118 9.76138 11.00000 209.00000 .00000

Model

coeff se t p LLCI ULCI

constant 114.18227 10.52968 10.84385 .00000 93.42428 134.94027

Stigma_I -1.79147 .24583 -7.28743 .00000 -2.27610 -1.30685

Manageab .56240 .20852 2.69713 .00756 .15133 .97346

Age_cov -.09582 .12785 -.74947 .45442 -.34786 .15622

Educatio -6.48571 3.42374 -1.89434 .05956 -13.23520 .26377

Employme -4.86758 3.61640 -1.34597 .17977 -11.99689 2.26173

Social_p 1.60277 .80228 1.99776 .04704 .02116 3.18437

Insuranc 5.48418 4.01550 1.36575 .17348 -2.43189 13.40026

Major_Ps -5.49651 2.72252 -2.01890 .04477 -10.86363 -.12938

Chemo_co -2.44491 1.69916 -1.43890 .15168 -5.79459 .90477

Mastecto -1.21981 1.60787 -.75865 .44892 -4.38953 1.94992

Radiothe -.43794 2.90511 -.15075 .88032 -6.16502 5.28914

Standardized coefficients

coeff

Stigma_I -.44163

Manageab .16218

Age_cov -.04379

Educatio -.12770

Employme -.09120

Social_p .11562

Insuranc .07896

Major_Ps -.11778

Chemo_co -.10426

Mastecto -.04918

Radiothe -.01076

| **Benjamini-Hochberg Procedure** | | | | |
| --- | --- | --- | --- | --- |
|  |  | **Ascending P-values** | **I = ranking** | **(I/11)* 0.10** |
|  | **Stigma** | **.00000** | **1** | **.009** |
|  | **Manageability** | **.007** | **2** | **.01818** |
|  | **Major Psychological issues** | **~~.044~~** | **3** | **.02727** |
|  | **Social participation frequency** | **~~.047~~** | **4** | **.03636** |
|  | **Education** | **~~.059~~** | **5** | **.045** |
|  | **Chemotherapy** | **~~.151~~** | **6** | **.054** |
|  | **Insurance** | **~~.173~~** | **7** | **.063** |
|  | **Employment** | **~~.179~~** | **8** | **.072** |
|  | **Mastectomy** | **~~.448~~** | **9** | **.081** |
|  | **Age** | **~~.454~~** | **10** | **.0909** |
|  | **Radiotherapy** | **~~.880~~** | **11** | **.100** |

************************** TOTAL EFFECT MODEL ****************************

OUTCOME VARIABLE:

FACT_B

Model Summary

R R-sq MSE F df1 df2 p

.56249 .31640 285.62775 9.71966 10.00000 210.00000 .00000

Model

coeff se t p LLCI ULCI

constant 124.01187 10.02536 12.36982 .00000 104.24862 143.77512

Stigma_I -1.94096 .24305 -7.98573 .00000 -2.42009 -1.46182

Age_cov -.06061 .12907 -.46963 .63910 -.31504 .19382

Educatio -5.65102 3.46028 -1.63311 .10394 -12.47236 1.17033

Employme -4.36095 3.66508 -1.18986 .23544 -11.58601 2.86412

Social_p 1.89265 .80684 2.34575 .01992 .30210 3.48319

Insuranc 4.87036 4.06850 1.19709 .23262 -3.14997 12.89070

Major_Ps -6.46957 2.73853 -2.36242 .01907 -11.86810 -1.07103

Chemo_co -2.47656 1.72431 -1.43626 .15242 -5.87574 .92263

Mastecto -1.60359 1.62531 -.98664 .32496 -4.80761 1.60043

Radiothe -.38631 2.94813 -.13104 .89587 -6.19803 5.42541

Standardized coefficients

coeff

Stigma_I -.47848

Age_cov -.02770

Educatio -.11127

Employme -.08171

Social_p .13653

Insuranc .07012

Major_Ps -.13863

Chemo_co -.10561

Mastecto -.06465

Radiothe -.00949

| **Benjamini-Hochberg Procedure** | | | | |
| --- | --- | --- | --- | --- |
|  |  | **Ascending P-value s** | **I = ranking** | **(I/10)* 0.10** |
|  | **Stigma** | **.00000** | **1** | **.01** |
|  | **Major Psychological issues** | **.0190** | **2** | **.02** |
|  | **Social participation frequency** | **.0199** | **3** | **.03** |
|  | **Education** | **~~.103~~** | **4** | **.04** |
|  | **Chemotherapy** | **~~.152~~** | **5** | **.05** |
|  | **Insurance** | **~~.232~~** | **6** | **.06** |
|  | **Employment** | **~~.235~~** | **7** | **.07** |
|  | **Mastectomy** | **~~.324~~** | **8** | **.08** |
|  | **Age** | **~~.639~~** | **9** | **.09** |
|  | **Radiotherapy** | **~~.898~~** | **10** | **.10** |

************** TOTAL, DIRECT, AND INDIRECT EFFECTS OF X ON Y **************

Total effect of X on Y

Effect se t p LLCI ULCI c_ps c_cs

-1.94096 .24305 -7.98573 .00000 -2.42009 -1.46182 -.09719 -.47848

Direct effect of X on Y

Effect se t p LLCI ULCI c'_ps c'_cs

-1.79147 .24583 -7.28743 .00000 -2.27610 -1.30685 -.08970 -.44163

Indirect effect(s) of X on Y:

Effect BootSE BootLLCI BootULCI

Manageab -.14948 .06750 -.29341 -.03175

Partially standardized indirect effect(s) of X on Y:

Effect BootSE BootLLCI BootULCI

Manageab -.00749 .00335 -.01472 -.00158

Completely standardized indirect effect(s) of X on Y:

Effect BootSE BootLLCI BootULCI

Manageab -.03685 .01658 -.07225 -.00759

*********************** ANALYSIS NOTES AND ERRORS ************************

Level of confidence for all confidence intervals in output:

95.0000

Number of bootstrap samples for percentile bootstrap confidence intervals:

5000

***************** PROCESS Procedure for SPSS Version 3.5 *****************

Written by Andrew F. Hayes, Ph.D. www.afhayes.com

Documentation available in Hayes (2018). www.guilford.com/p/hayes3

**************************************************************************

**Model : 4**

**Y : FACT_B**

**X : Stigma_I**

**M : Meaningfulness**

Covariates:

Age_cov Educatio Employme Social_p Insuranc Major_Ps Chemo_co Mastecto Radiothe

Sample

Size: 221

**************************************************************************

OUTCOME VARIABLE:

Meaningf

Model Summary

R R-sq MSE F df1 df2 p

.43435 .18866 20.31616 4.88319 10.00000 210.00000 .00000

Model

coeff se t p LLCI ULCI

constant 22.12763 2.67375 8.27588 .00000 16.85680 27.39846

Stigma_I -.23369 .06482 -3.60503 .00039 -.36147 -.10590

Age_cov .05889 .03442 1.71069 .08861 -.00897 .12674

Educatio -1.59914 .92285 -1.73283 .08459 -3.41839 .22010

Employme .60739 .97747 .62139 .53502 -1.31953 2.53430

Social_p .53412 .21518 2.48215 .01384 .10992 .95831

Insuranc .02574 1.08506 .02372 .98110 -2.11327 2.16475

Major_Ps -1.33952 .73036 -1.83404 .06806 -2.77930 .10026

Chemo_co -.26584 .45987 -.57808 .56383 -1.17240 .64071

Mastecto -.84146 .43347 -1.94122 .05357 -1.69596 .01305

Radiothe .61470 .78626 .78180 .43521 -.93528 2.16468

Standardized coefficients

coeff

Stigma_I -.23532

Age_cov .10993

Educatio -.12862

Employme .04649

Social_p .15739

Insuranc .00151

Major_Ps -.11725

Chemo_co -.04631

Mastecto -.13858

Radiothe .06169

| **Benjamini-Hochberg Procedure** | | | | |
| --- | --- | --- | --- | --- |
|  |  | **Ascending P-value s** | **I = ranking** | **(I/10)* 0.10** |
|  | **Stigma** | **.0009** | **1** | **.01** |
|  | **Social participation frequency** | **.013** | **2** | **.02** |
|  | **Mastectomy** | **~~.053~~** | **3** | **.03** |
|  | **Major Psychological issues** | **~~.068~~** | **4** | **.04** |
|  | **Education** | **~~.084~~** | **5** | **.05** |
|  | **Age** | **~~.088~~** | **6** | **.06** |
|  | **Radiotherapy** | **~~.435~~** | **7** | **.07** |
|  | **Employment** | **~~.535~~** | **8** | **.08** |
|  | **Chemotherapy** | **~~.563~~** | **9** | **.09** |
|  | **Insurance** | **~~.981~~** | **10** | **.10** |

**************************************************************************

OUTCOME VARIABLE:

FACT_B

Model Summary

R R-sq MSE F df1 df2 p

.62449 .38999 256.09964 12.14692 11.00000 209.00000 .00000

Model

coeff se t p LLCI ULCI

constant 96.78987 10.93200 8.85381 .00000 75.23874 118.34100

Stigma_I -1.65347 .23716 -6.97191 .00000 -2.12101 -1.18593

Meaningf 1.23023 .24500 5.02124 .00000 .74723 1.71322

Age_cov -.13306 .12306 -1.08121 .28085 -.37566 .10955

Educatio -3.68371 3.29989 -1.11631 .26557 -10.18904 2.82162

Employme -5.10817 3.47365 -1.47055 .14292 -11.95606 1.73972

Social_p 1.23556 .77512 1.59402 .11244 -.29250 2.76363

Insuranc 4.83870 3.85247 1.25600 .21052 -2.75598 12.43337

Major_Ps -4.82166 2.61380 -1.84469 .06650 -9.97445 .33114

Chemo_co -2.14951 1.63405 -1.31545 .18980 -5.37085 1.07183

Mastecto -.56841 1.55275 -.36607 .71469 -3.62948 2.49266

Radiothe -1.14253 2.79564 -.40868 .68319 -6.65381 4.36874

Standardized coefficients

coeff

Stigma_I -.40761

Meaningf .30117

Age_cov -.06081

Educatio -.07253

Employme -.09571

Social_p .08913

Insuranc .06967

Major_Ps -.10332

Chemo_co -.09166

Mastecto -.02292

Radiothe -.02807

| **Benjamini-Hochberg Procedure** | | | | |
| --- | --- | --- | --- | --- |
|  |  | **Ascending P-value s** | **I = ranking** | **(I/11)* 0.10** |
|  | **Stigma** | **.00000** | **1** | **.009** |
|  | **Meaningfulness** | **.00000** | **2** | **.01818** |
|  | **Major Psychological issues** | **~~.066~~** | **3** | **.027** |
|  | **Social participation frequency** | **~~.112~~** | **4** | **.03636** |
|  | **Employment** | **~~.142~~** | **5** | **.045** |
|  | **Chemotherapy** | **~~.189~~** | **6** | **.054** |
|  | **Insurance** | **~~.210~~** | **7** | **.063** |
|  | **Education** | **~~.265~~** | **8** | **.072** |
|  | **Age** | **~~.280~~** | **9** | **.081** |
|  | **Radiotherapy** | **~~.683~~** | **10** | **.0909** |
|  | **Mastectomy** | **~~.714~~** | **11** | **.100** |

************************** TOTAL EFFECT MODEL ****************************

OUTCOME VARIABLE:

FACT_B

Model Summary

R R-sq MSE F df1 df2 p

.56249 .31640 285.62775 9.71966 10.00000 210.00000 .00000

Model

coeff se t p LLCI ULCI

constant 124.01187 10.02536 12.36982 .00000 104.24862 143.77512

Stigma_I -1.94096 .24305 -7.98573 .00000 -2.42009 -1.46182

Age_cov -.06061 .12907 -.46963 .63910 -.31504 .19382

Educatio -5.65102 3.46028 -1.63311 .10394 -12.47236 1.17033

Employme -4.36095 3.66508 -1.18986 .23544 -11.58601 2.86412

Social_p 1.89265 .80684 2.34575 .01992 .30210 3.48319

Insuranc 4.87036 4.06850 1.19709 .23262 -3.14997 12.89070

Major_Ps -6.46957 2.73853 -2.36242 .01907 -11.86810 -1.07103

Chemo_co -2.47656 1.72431 -1.43626 .15242 -5.87574 .92263

Mastecto -1.60359 1.62531 -.98664 .32496 -4.80761 1.60043

Radiothe -.38631 2.94813 -.13104 .89587 -6.19803 5.42541

Standardized coefficients

coeff

Stigma_I -.47848

Age_cov -.02770

Educatio -.11127

Employme -.08171

Social_p .13653

Insuranc .07012

Major_Ps -.13863

Chemo_co -.10561

Mastecto -.06465

Radiothe -.00949

| **Benjamini-Hochberg Procedure** | | | | |
| --- | --- | --- | --- | --- |
|  |  | **Ascending P-value s** | **I = ranking** | **(I/10)* 0.10** |
|  | **Stigma** | **.00000** | **1** | **.01** |
|  | **Major Psychological issues** | **.0190** | **2** | **.02** |
|  | **Social participation frequency** | **.0199** | **3** | **.03** |
|  | **Education** | **~~.103~~** | **4** | **.04** |
|  | **Chemotherapy** | **~~.152~~** | **5** | **.05** |
|  | **Insurance** | **~~.232~~** | **6** | **.06** |
|  | **Employment** | **~~.235~~** | **7** | **.07** |
|  | **Mastectomy** | **~~.324~~** | **8** | **.08** |
|  | **Age** | **~~.639~~** | **9** | **.09** |
|  | **Radiotherapy** | **~~.898~~** | **10** | **.10** |

************** TOTAL, DIRECT, AND INDIRECT EFFECTS OF X ON Y **************

Total effect of X on Y

Effect se t p LLCI ULCI c_ps c_cs

-1.94096 .24305 -7.98573 .00000 -2.42009 -1.46182 -.09719 -.47848

Direct effect of X on Y

Effect se t p LLCI ULCI c'_ps c'_cs

-1.65347 .23716 -6.97191 .00000 -2.12101 -1.18593 -.08279 -.40761

Indirect effect(s) of X on Y:

Effect BootSE BootLLCI BootULCI

Meaningf -.28749 .09264 -.48785 -.12302

Partially standardized indirect effect(s) of X on Y:

Effect BootSE BootLLCI BootULCI

Meaningf -.01440 .00458 -.02422 -.00628

Completely standardized indirect effect(s) of X on Y:

Effect BootSE BootLLCI BootULCI

Meaningf -.07087 .02280 -.11972 -.03077

*********************** ANALYSIS NOTES AND ERRORS ************************

Level of confidence for all confidence intervals in output:

95.0000

Number of bootstrap samples for percentile bootstrap confidence intervals:

5000

***************** PROCESS Procedure for SPSS Version 3.5 *****************

Written by Andrew F. Hayes, Ph.D. www.afhayes.com

Documentation available in Hayes (2018). www.guilford.com/p/hayes3

**************************************************************************

**Model : 4**

**Y : FACT_B**

**X : Stigma_I**

**M : SOC = sense of coherence**

Covariates:

Age_cov Educatio Employme Social_p Insuranc Major_Ps Chemo_co Mastecto Radiothe

Sample

Size: 221

**************************************************************************

OUTCOME VARIABLE:

SOC

Model Summary

R R-sq MSE F df1 df2 p

.45548 .20746 151.84085 5.49712 10.00000 210.00000 .00000

Model

coeff se t p LLCI ULCI

constant 68.01609 7.30961 9.30502 .00000 53.60647 82.42570

Stigma_I -.82220 .17721 -4.63961 .00001 -1.17154 -.47285

Age_cov .16923 .09410 1.79834 .07356 -.01628 .35474

Educatio -.43019 2.52293 -.17051 .86477 -5.40371 4.54333

Employme 3.33713 2.67225 1.24881 .21313 -1.93075 8.60501

Social_p 1.41471 .58828 2.40485 .01705 .25503 2.57440

Insuranc -1.62359 2.96639 -.54733 .58473 -7.47131 4.22413

Major_Ps -4.96124 1.99669 -2.48473 .01375 -8.89738 -1.02511

Chemo_co -1.48699 1.25722 -1.18276 .23824 -3.96537 .99139

Mastecto -2.99414 1.18503 -2.52663 .01225 -5.33022 -.65805

Radiothe -.38276 2.14952 -.17807 .85884 -4.62015 3.85464

Standardized coefficients

coeff

Stigma_I -.29932

Age_cov .11422

Educatio -.01251

Employme .09234

Social_p .15071

Insuranc -.03452

Major_Ps -.15700

Chemo_co -.09364

Mastecto -.17827

Radiothe -.01389

| **Benjamini-Hochberg Procedure** | | | | |
| --- | --- | --- | --- | --- |
|  |  | **Ascending P-value s** | **I = ranking** | **(I/10)* 0.10** |
|  | **Stigma** | **.00001** | **1** | **.01** |
|  | **Social participation frequency** | **.017** | **2** | **.02** |
|  | **Mastectomy** | **.012** | **3** | **.03** |
|  | **Major Psychological issues** | **.013** | **4** | **.04** |
|  | **Age** | **~~.073~~** | **5** | **.05** |
|  | **Employment** | **~~.213~~** | **6** | **.06** |
|  | **Chemotherapy** | **~~.238~~** | **7** | **.07** |
|  | **Insurance** | **~~.584~~** | **8** | **.08** |
|  | **Radiotherapy** | **~~.858~~** | **9** | **.09** |
|  | **Education** | **~~.864~~** | **10** | **.10** |

**************************************************************************

OUTCOME VARIABLE:

FACT_B

Model Summary

R R-sq MSE F df1 df2 p

.61259 .37526 262.28208 11.41273 11.00000 209.00000 .00000

Model

coeff se t p LLCI ULCI

constant 96.63792 11.41689 8.46447 .00000 74.13088 119.14496

Stigma_I -1.61005 .24455 -6.58361 .00000 -2.09216 -1.12794

SOC .40246 .09069 4.43757 .00001 .22367 .58126

Age_cov -.12872 .12463 -1.03286 .30286 -.37441 .11697

Educatio -5.47788 3.31609 -1.65191 .10005 -12.01515 1.05939

Employme -5.70402 3.52512 -1.61811 .10715 -12.65338 1.24533

Social_p 1.32328 .78374 1.68842 .09282 -.22177 2.86832

Insuranc 5.52380 3.90146 1.41583 .15831 -2.16747 13.21507

Major_Ps -4.47285 2.66252 -1.67993 .09447 -9.72170 .77600

Chemo_co -1.87810 1.65784 -1.13286 .25857 -5.14632 1.39013

Mastecto -.39856 1.58097 -.25210 .80121 -3.51525 2.71813

Radiothe -.23226 2.82529 -.08221 .93456 -5.80199 5.33746

Standardized coefficients

coeff

Stigma_I -.39691

SOC .27253

Age_cov -.05883

Educatio -.10786

Employme -.10688

Social_p .09546

Insuranc .07953

Major_Ps -.09585

Chemo_co -.08009

Mastecto -.01607

Radiothe -.00571

| **Benjamini-Hochberg Procedure** | | | | |
| --- | --- | --- | --- | --- |
|  |  | **Ascending P-value s** | **I = ranking** | **(I/11)* 0.10** |
|  | **Stigma** | **.00000** | **1** | **.009** |
|  | **Sense of Coherence** | **.00001** | **2** | **.01818** |
|  | **Major Psychological issues** | **~~.094~~** | **3** | **.027** |
|  | **Social participation frequency** | **~~.092~~** | **4** | **.03636** |
|  | **Employment** | **~~.107~~** | **5** | **.045** |
|  | **Education** | **~~.100~~** | **6** | **.054** |
|  | **Age** | **~~.122~~** | **7** | **.063** |
|  | **Insurance** | **~~.158~~** | **8** | **.072** |
|  | **Chemotherapy** | **~~.258~~** | **9** | **.081** |
|  | **Mastectomy** | **~~.801~~** | **10** | **.0909** |
|  | **Radiotherapy** | **~~.934~~** | **11** | **.100** |

************************** TOTAL EFFECT MODEL ****************************

OUTCOME VARIABLE:

FACT_B

Model Summary

R R-sq MSE F df1 df2 p

.56249 .31640 285.62775 9.71966 10.00000 210.00000 .00000

Model

coeff se t p LLCI ULCI

constant 124.01187 10.02536 12.36982 .00000 104.24862 143.77512

Stigma_I -1.94096 .24305 -7.98573 .00000 -2.42009 -1.46182

Age_cov -.06061 .12907 -.46963 .63910 -.31504 .19382

Educatio -5.65102 3.46028 -1.63311 .10394 -12.47236 1.17033

Employme -4.36095 3.66508 -1.18986 .23544 -11.58601 2.86412

Social_p 1.89265 .80684 2.34575 .01992 .30210 3.48319

Insuranc 4.87036 4.06850 1.19709 .23262 -3.14997 12.89070

Major_Ps -6.46957 2.73853 -2.36242 .01907 -11.86810 -1.07103

Chemo_co -2.47656 1.72431 -1.43626 .15242 -5.87574 .92263

Mastecto -1.60359 1.62531 -.98664 .32496 -4.80761 1.60043

Radiothe -.38631 2.94813 -.13104 .89587 -6.19803 5.42541

Standardized coefficients

coeff

Stigma_I -.47848

Age_cov -.02770

Educatio -.11127

Employme -.08171

Social_p .13653

Insuranc .07012

Major_Ps -.13863

Chemo_co -.10561

Mastecto -.06465

Radiothe -.00949

| **Benjamini-Hochberg Procedure** | | | | |
| --- | --- | --- | --- | --- |
|  |  | **Ascending P-value s** | **I = ranking** | **(I/10)* 0.10** |
|  | **Stigma** | **.00000** | **1** | **.01** |
|  | **Major Psychological issues** | **.0190** | **2** | **.02** |
|  | **Social participation frequency** | **.0199** | **3** | **.03** |
|  | **Education** | **~~.103~~** | **4** | **.04** |
|  | **Chemotherapy** | **~~.152~~** | **5** | **.05** |
|  | **Insurance** | **~~.232~~** | **6** | **.06** |
|  | **Employment** | **~~.235~~** | **7** | **.07** |
|  | **Mastectomy** | **~~.324~~** | **8** | **.08** |
|  | **Age** | **~~.639~~** | **9** | **.09** |
|  | **Radiotherapy** | **~~.898~~** | **10** | **.10** |

************** TOTAL, DIRECT, AND INDIRECT EFFECTS OF X ON Y **************

Total effect of X on Y

Effect se t p LLCI ULCI c_ps c_cs

-1.94096 .24305 -7.98573 .00000 -2.42009 -1.46182 -.09719 -.47848

Direct effect of X on Y

Effect se t p LLCI ULCI c'_ps c'_cs

-1.61005 .24455 -6.58361 .00000 -2.09216 -1.12794 -.08062 -.39691

Indirect effect(s) of X on Y:

Effect BootSE BootLLCI BootULCI

SOC -.33090 .09514 -.53925 -.16432

Partially standardized indirect effect(s) of X on Y:

Effect BootSE BootLLCI BootULCI

SOC -.01657 .00472 -.02679 -.00838

Completely standardized indirect effect(s) of X on Y:

Effect BootSE BootLLCI BootULCI

SOC -.08157 .02295 -.12999 -.04080

*********************** ANALYSIS NOTES AND ERRORS ************************

Level of confidence for all confidence intervals in output:

95.0000

Number of bootstrap samples for percentile bootstrap confidence intervals:

5000

***************** PROCESS Procedure for SPSS Version 3.5 *****************

Written by Andrew F. Hayes, Ph.D. www.afhayes.com

Documentation available in Hayes (2018). www.guilford.com/p/hayes3

**************************************************************************

**Model : 4**

**Y : FACT_B**

**X : Stigma_I**

**M : Emoinfo = emotional informational support**

Covariates:

Age_cov Educatio Employme Social_p Insuranc Major_Ps Chemo_co Mastecto Radiothe

Sample

Size: 221

**************************************************************************

OUTCOME VARIABLE:

Emoinfo

Model Summary

R R-sq MSE F df1 df2 p

.36001 .12960 .06415 3.12695 10.00000 210.00000 .00096

Model

coeff se t p LLCI ULCI

constant .51430 .15025 3.42300 .00074 .21811 .81049

Stigma_I -.01133 .00364 -3.10925 .00214 -.01851 -.00414

Age_cov .00164 .00193 .84938 .39664 -.00217 .00546

Educatio -.01597 .05186 -.30795 .75843 -.11820 .08626

Employme -.01900 .05493 -.34599 .72969 -.12728 .08928

Social_p .00346 .01209 .28655 .77474 -.02037 .02730

Insuranc .04089 .06097 .67054 .50325 -.07931 .16108

Major_Ps -.13273 .04104 -3.23408 .00142 -.21364 -.05183

Chemo_co -.00108 .02584 -.04187 .96664 -.05202 .04986

Mastecto -.01604 .02436 -.65839 .51101 -.06406 .03198

Radiothe .03875 .04418 .87701 .38148 -.04835 .12585

Standardized coefficients

coeff

Stigma_I -.21022

Age_cov .05653

Educatio -.02368

Employme -.02681

Social_p .01882

Insuranc .04432

Major_Ps -.21415

Chemo_co -.00347

Mastecto -.04868

Radiothe .07168

| **Benjamini-Hochberg Procedure** | | | | |
| --- | --- | --- | --- | --- |
|  |  | **Ascending P-value s** | **I = ranking** | **(I/10)* 0.10** |
|  | **Major Psychological issues** | **.001** | **1** | **.01** |
|  | **Stigma** | **.002** | **2** | **.02** |
|  | **Radiotherapy** | **~~.381~~** | **3** | **.03** |
|  | **Age** | **~~.396~~** | **4** | **.04** |
|  | **Insurance** | **~~.503~~** | **5** | **.05** |
|  | **Mastectomy** | **~~.511~~** | **6** | **.06** |
|  | **Employment** | **~~.729~~** | **7** | **.07** |
|  | **Education** | **~~.758~~** | **8** | **.08** |
|  | **Social participation frequency** | **~~.774~~** | **9** | **.09** |
|  | **Chemotherapy** | **~~.966~~** | **10** | **.10** |

**************************************************************************

OUTCOME VARIABLE:

FACT_B

Model Summary

R R-sq MSE F df1 df2 p

.59237 .35090 272.51067 10.27120 11.00000 209.00000 .00000

Model

coeff se t p LLCI ULCI

constant 116.30265 10.06193 11.55868 .00000 96.46675 136.13854

Stigma_I -1.77119 .24281 -7.29455 .00000 -2.24986 -1.29252

Emoinfo 14.98978 4.49753 3.33289 .00102 6.12345 23.85612

Age_cov -.08524 .12628 -.67499 .50043 -.33419 .16371

Educatio -5.41163 3.38066 -1.60076 .11094 -12.07620 1.25293

Employme -4.07607 3.58095 -1.13827 .25631 -11.13550 2.98335

Social_p 1.84071 .78825 2.33519 .02048 .28677 3.39465

Insuranc 4.25751 3.97823 1.07020 .28576 -3.58510 12.10011

Major_Ps -4.47994 2.74071 -1.63459 .10364 -9.88293 .92305

Chemo_co -2.46034 1.68426 -1.46078 .14558 -5.78065 .85998

Mastecto -1.36320 1.58919 -.85779 .39199 -4.49609 1.76970

Radiothe -.96715 2.88491 -.33524 .73778 -6.65440 4.72010

Standardized coefficients

coeff

Stigma_I -.43663

Emoinfo .19909

Age_cov -.03896

Educatio -.10656

Employme -.07637

Social_p .13278

Insuranc .06130

Major_Ps -.09600

Chemo_co -.10491

Mastecto -.05496

Radiothe -.02376

| **Benjamini-Hochberg Procedure** | | | | |
| --- | --- | --- | --- | --- |
|  |  | **Ascending P-value s** | **I = ranking** | **(I/11)* 0.10** |
|  | **Stigma** | **.00000** | **1** | **.009** |
|  | **Emotional informational support** | **.001** | **2** | **.01818** |
|  | **Social participation frequency** | **.020** | **3** | **.02727** |
|  | **Major Psychological issues** | **~~.103~~** | **4** | **.03636** |
|  | **Education** | **~~.110~~** | **5** | **.045** |
|  | **Chemotherapy** | **~~.154~~** | **6** | **.054** |
|  | **Employment** | **~~.256~~** | **7** | **.063** |
|  | **Insurance** | **~~.285~~** | **8** | **.072** |
|  | **Mastectomy** | **~~.391~~** | **9** | **.081** |
|  | **Age** | **~~.500~~** | **10** | **.0909** |
|  | **Radiotherapy** | **~~.737~~** | **11** | **.100** |

************************** TOTAL EFFECT MODEL ****************************

OUTCOME VARIABLE:

FACT_B

Model Summary

R R-sq MSE F df1 df2 p

.56249 .31640 285.62775 9.71966 10.00000 210.00000 .00000

Model

coeff se t p LLCI ULCI

constant 124.01187 10.02536 12.36982 .00000 104.24862 143.77512

Stigma_I -1.94096 .24305 -7.98573 .00000 -2.42009 -1.46182

Age_cov -.06061 .12907 -.46963 .63910 -.31504 .19382

Educatio -5.65102 3.46028 -1.63311 .10394 -12.47236 1.17033

Employme -4.36095 3.66508 -1.18986 .23544 -11.58601 2.86412

Social_p 1.89265 .80684 2.34575 .01992 .30210 3.48319

Insuranc 4.87036 4.06850 1.19709 .23262 -3.14997 12.89070

Major_Ps -6.46957 2.73853 -2.36242 .01907 -11.86810 -1.07103

Chemo_co -2.47656 1.72431 -1.43626 .15242 -5.87574 .92263

Mastecto -1.60359 1.62531 -.98664 .32496 -4.80761 1.60043

Radiothe -.38631 2.94813 -.13104 .89587 -6.19803 5.42541

Standardized coefficients

coeff

Stigma_I -.47848

Age_cov -.02770

Educatio -.11127

Employme -.08171

Social_p .13653

Insuranc .07012

Major_Ps -.13863

Chemo_co -.10561

Mastecto -.06465

Radiothe -.00949

| **Benjamini-Hochberg Procedure** | | | | |
| --- | --- | --- | --- | --- |
|  |  | **Ascending P-value s** | **I = ranking** | **(I/10)* 0.10** |
|  | **Stigma** | **.00000** | **1** | **.01** |
|  | **Major Psychological issues** | **.0190** | **2** | **.02** |
|  | **Social participation frequency** | **.0199** | **3** | **.03** |
|  | **Education** | **~~.103~~** | **4** | **.04** |
|  | **Chemotherapy** | **~~.152~~** | **5** | **.05** |
|  | **Insurance** | **~~.232~~** | **6** | **.06** |
|  | **Employment** | **~~.235~~** | **7** | **.07** |
|  | **Mastectomy** | **~~.324~~** | **8** | **.08** |
|  | **Age** | **~~.639~~** | **9** | **.09** |
|  | **Radiotherapy** | **~~.898~~** | **10** | **.10** |

************** TOTAL, DIRECT, AND INDIRECT EFFECTS OF X ON Y **************

Total effect of X on Y

Effect se t p LLCI ULCI c_ps c_cs

-1.94096 .24305 -7.98573 .00000 -2.42009 -1.46182 -.09719 -.47848

Direct effect of X on Y

Effect se t p LLCI ULCI c'_ps c'_cs

-1.77119 .24281 -7.29455 .00000 -2.24986 -1.29252 -.08869 -.43663

Indirect effect(s) of X on Y:

Effect BootSE BootLLCI BootULCI

Emoinfo -.16977 .06660 -.31166 -.05008

Partially standardized indirect effect(s) of X on Y:

Effect BootSE BootLLCI BootULCI

Emoinfo -.00850 .00331 -.01558 -.00250

Completely standardized indirect effect(s) of X on Y:

Effect BootSE BootLLCI BootULCI

Emoinfo -.04185 .01650 -.07706 -.01216

*********************** ANALYSIS NOTES AND ERRORS ************************

Level of confidence for all confidence intervals in output:

95.0000

Number of bootstrap samples for percentile bootstrap confidence intervals:

5000

***************** PROCESS Procedure for SPSS Version 3.5 *****************

Written by Andrew F. Hayes, Ph.D. www.afhayes.com

Documentation available in Hayes (2018). www.guilford.com/p/hayes3

**************************************************************************

**Model : 4**

**Y : FACT_B**

**X : Stigma_I**

**M : Affectionate support**

Covariates:

Age_cov Educatio Employme Social_p Insuranc Major_Ps Chemo_co Mastecto Radiothe

Sample

Size: 221

**************************************************************************

OUTCOME VARIABLE:

Affectio

Model Summary

R R-sq MSE F df1 df2 p

.37797 .14286 .05276 3.50013 10.00000 210.00000 .00027

Model

coeff se t p LLCI ULCI

constant .48253 .13625 3.54144 .00049 .21393 .75112

Stigma_I -.00910 .00330 -2.75500 .00638 -.01561 -.00259

Age_cov .00105 .00175 .59605 .55178 -.00241 .00450

Educatio -.01455 .04703 -.30944 .75729 -.10726 .07815

Employme -.02925 .04981 -.58719 .55770 -.12744 .06894

Social_p .00296 .01097 .27031 .78719 -.01865 .02458

Insuranc .07932 .05529 1.43455 .15290 -.02968 .18832

Major_Ps -.11830 .03722 -3.17845 .00170 -.19167 -.04493

Chemo_co -.00828 .02343 -.35349 .72408 -.05448 .03791

Mastecto -.03515 .02209 -1.59143 .11302 -.07870 .00839

Radiothe .05027 .04007 1.25454 .21104 -.02872 .12925

Standardized coefficients

coeff

Stigma_I -.18484

Age_cov .03937

Educatio -.02361

Employme -.04515

Social_p .01762

Insuranc .09410

Major_Ps -.20885

Chemo_co -.02910

Mastecto -.11678

Radiothe .10175

| **Benjamini-Hochberg Procedure** | | | | |
| --- | --- | --- | --- | --- |
|  |  | **Ascending P-value s** | **I = ranking** | **(I/10)* 0.10** |
|  | **Major Psychological issues** | **.001** | **1** | **.01** |
|  | **Stigma** | **.006** | **2** | **.02** |
|  | **Mastectomy** | **~~.113~~** | **3** | **.03** |
|  | **Insurance** | **~~.152~~** | **4** | **.04** |
|  | **Radiotherapy** | **~~.211~~** | **5** | **.05** |
|  | **Age** | **~~.551~~** | **6** | **.06** |
|  | **Employment** | **~~.557~~** | **7** | **.07** |
|  | **Chemotherapy** | **~~.724~~** | **8** | **.08** |
|  | **Education** | **~~.757~~** | **9** | **.09** |
|  | **Social participation frequency** | **~~.787~~** | **10** | **.10** |

**************************************************************************

OUTCOME VARIABLE:

FACT_B

Model Summary

R R-sq MSE F df1 df2 p

.59858 .35829 269.40588 10.60854 11.00000 209.00000 .00000

Model

coeff se t p LLCI ULCI

constant 115.22250 10.02304 11.49576 .00000 95.46328 134.98173

Stigma_I -1.77519 .24028 -7.38805 .00000 -2.24887 -1.30151

Affectio 18.21535 4.93121 3.69389 .00028 8.49406 27.93664

Age_cov -.07966 .12545 -.63496 .52615 -.32697 .16766

Educatio -5.38594 3.36135 -1.60231 .11060 -12.01244 1.24057

Employme -3.82818 3.56240 -1.07461 .28379 -10.85102 3.19467

Social_p 1.83866 .78373 2.34603 .01991 .29363 3.38369

Insuranc 3.42550 3.97059 .86272 .38928 -4.40204 11.25304

Major_Ps -4.31475 2.72285 -1.58465 .11456 -9.68252 1.05302

Chemo_co -2.32566 1.67513 -1.38835 .16651 -5.62798 .97665

Mastecto -.96326 1.58797 -.60660 .54477 -4.09376 2.16723

Radiothe -1.30192 2.87390 -.45302 .65101 -6.96746 4.36363

Standardized coefficients

coeff

Stigma_I -.43762

Affectio .22108

Age_cov -.03641

Educatio -.10605

Employme -.07173

Social_p .13264

Insuranc .04932

Major_Ps -.09246

Chemo_co -.09917

Mastecto -.03884

Radiothe -.03199

| **Benjamini-Hochberg Procedure** | | | | |
| --- | --- | --- | --- | --- |
|  |  | **Ascending P-value s** | **I = ranking** | **(I/11)* 0.10** |
|  | **Stigma** | **.00000** | **1** | **.009** |
|  | **Affectionate support** | **.00028** | **2** | **.01818** |
|  | **Social participation frequency** | **.019** | **3** | **.02727** |
|  | **Major Psychological issues** | **~~.114~~** | **4** | **.03636** |
|  | **Education** | **~~.110~~** | **5** | **.045** |
|  | **Chemotherapy** | **~~.166~~** | **6** | **.054** |
|  | **Employment** | **~~.283~~** | **7** | **.063** |
|  | **Insurance** | **~~.389~~** | **8** | **.072** |
|  | **Age** | **~~.526~~** | **9** | **.081** |
|  | **Mastectomy** | **~~.544~~** | **10** | **.0909** |
|  | **Radiotherapy** | **~~.651~~** | **11** | **.100** |

************************** TOTAL EFFECT MODEL ****************************

OUTCOME VARIABLE:

FACT_B

Model Summary

R R-sq MSE F df1 df2 p

.56249 .31640 285.62775 9.71966 10.00000 210.00000 .00000

Model

coeff se t p LLCI ULCI

constant 124.01187 10.02536 12.36982 .00000 104.24862 143.77512

Stigma_I -1.94096 .24305 -7.98573 .00000 -2.42009 -1.46182

Age_cov -.06061 .12907 -.46963 .63910 -.31504 .19382

Educatio -5.65102 3.46028 -1.63311 .10394 -12.47236 1.17033

Employme -4.36095 3.66508 -1.18986 .23544 -11.58601 2.86412

Social_p 1.89265 .80684 2.34575 .01992 .30210 3.48319

Insuranc 4.87036 4.06850 1.19709 .23262 -3.14997 12.89070

Major_Ps -6.46957 2.73853 -2.36242 .01907 -11.86810 -1.07103

Chemo_co -2.47656 1.72431 -1.43626 .15242 -5.87574 .92263

Mastecto -1.60359 1.62531 -.98664 .32496 -4.80761 1.60043

Radiothe -.38631 2.94813 -.13104 .89587 -6.19803 5.42541

Standardized coefficients

coeff

Stigma_I -.47848

Age_cov -.02770

Educatio -.11127

Employme -.08171

Social_p .13653

Insuranc .07012

Major_Ps -.13863

Chemo_co -.10561

Mastecto -.06465

Radiothe -.00949

| **Benjamini-Hochberg Procedure** | | | | |
| --- | --- | --- | --- | --- |
|  |  | **Ascending P-value s** | **I = ranking** | **(I/10)* 0.10** |
|  | **Stigma** | **.00000** | **1** | **.01** |
|  | **Major Psychological issues** | **.0190** | **2** | **.02** |
|  | **Social participation frequency** | **.0199** | **3** | **.03** |
|  | **Education** | **~~.103~~** | **4** | **.04** |
|  | **Chemotherapy** | **~~.152~~** | **5** | **.05** |
|  | **Insurance** | **~~.232~~** | **6** | **.06** |
|  | **Employment** | **~~.235~~** | **7** | **.07** |
|  | **Mastectomy** | **~~.324~~** | **8** | **.08** |
|  | **Age** | **~~.639~~** | **9** | **.09** |
|  | **Radiotherapy** | **~~.898~~** | **10** | **.10** |

************** TOTAL, DIRECT, AND INDIRECT EFFECTS OF X ON Y **************

Total effect of X on Y

Effect se t p LLCI ULCI c_ps c_cs

-1.94096 .24305 -7.98573 .00000 -2.42009 -1.46182 -.09719 -.47848

Direct effect of X on Y

Effect se t p LLCI ULCI c'_ps c'_cs

-1.77519 .24028 -7.38805 .00000 -2.24887 -1.30151 -.08889 -.43762

Indirect effect(s) of X on Y:

Effect BootSE BootLLCI BootULCI

Affectio -.16577 .07314 -.32186 -.03809

Partially standardized indirect effect(s) of X on Y:

Effect BootSE BootLLCI BootULCI

Affectio -.00830 .00360 -.01594 -.00193

Completely standardized indirect effect(s) of X on Y:

Effect BootSE BootLLCI BootULCI

Affectio -.04087 .01809 -.07942 -.00926

*********************** ANALYSIS NOTES AND ERRORS ************************

Level of confidence for all confidence intervals in output:

95.0000

Number of bootstrap samples for percentile bootstrap confidence intervals:

5000

***************** PROCESS Procedure for SPSS Version 3.5 *****************

Written by Andrew F. Hayes, Ph.D. www.afhayes.com

Documentation available in Hayes (2018). www.guilford.com/p/hayes3

**************************************************************************

**Model : 4**

**Y : FACT_B**

**X : Stigma_I**

**M : Tangible support**

Covariates:

Age_cov Educatio Employme Social_p Insuranc Major_Ps Chemo_co Mastecto Radiothe

Sample

Size: 221

**************************************************************************

OUTCOME VARIABLE:

Tangible

Model Summary

R R-sq MSE F df1 df2 p

.36445 .13282 .05649 3.21649 10.00000 210.00000 .00071

Model

coeff se t p LLCI ULCI

constant .39310 .14099 2.78817 .00579 .11516 .67103

Stigma_I -.00884 .00342 -2.58739 .01035 -.01558 -.00211

Age_cov .00193 .00182 1.06059 .29010 -.00165 .00550

Educatio .01051 .04866 .21603 .82918 -.08542 .10644

Employme .00466 .05154 .09047 .92800 -.09694 .10627

Social_p -.01229 .01135 -1.08312 .28000 -.03466 .01008

Insuranc .08211 .05722 1.43514 .15274 -.03068 .19490

Major_Ps -.12284 .03851 -3.18964 .00164 -.19876 -.04692

Chemo_co -.00498 .02425 -.20536 .83749 -.05278 .04282

Mastecto -.02154 .02286 -.94221 .34717 -.06659 .02352

Radiothe .05682 .04146 1.37039 .17203 -.02491 .13855

Standardized coefficients

coeff

Stigma_I -.17461

Age_cov .07046

Educatio .01658

Employme .00700

Social_p -.07100

Insuranc .09469

Major_Ps -.21081

Chemo_co -.01701

Mastecto -.06954

Radiothe .11179

| **Benjamini-Hochberg Procedure** | | | | |
| --- | --- | --- | --- | --- |
|  |  | **Ascending P-value s** | **I = ranking** | **(I/10)* 0.10** |
|  | **Major Psychological issues** | **.001** | **1** | **.01** |
|  | **Stigma** | **.01** | **2** | **.02** |
|  | **Insurance** | **~~.152~~** | **3** | **.03** |
|  | **Radiotherapy** | **~~.172~~** | **4** | **.04** |
|  | **Social participation frequency** | **~~.280~~** | **5** | **.05** |
|  | **Age** | **~~.290~~** | **6** | **.06** |
|  | **Mastectomy** | **~~.347~~** | **7** | **.07** |
|  | **Education** | **~~.829~~** | **8** | **.08** |
|  | **Chemotherapy** | **~~.837~~** | **9** | **.09** |
|  | **Employment** | **~~.928~~** | **10** | **.10** |

**************************************************************************

OUTCOME VARIABLE:

FACT_B

Model Summary

R R-sq MSE F df1 df2 p

.57680 .33270 280.15232 9.47278 11.00000 209.00000 .00000

Model

coeff se t p LLCI ULCI

constant 119.69592 10.11091 11.83830 .00000 99.76348 139.62837

Stigma_I -1.84386 .24452 -7.54075 .00000 -2.32590 -1.36182

Tangible 10.97938 4.85968 2.25928 .02490 1.39909 20.55966

Age_cov -.08175 .12816 -.63784 .52427 -.33441 .17091

Educatio -5.76643 3.42734 -1.68248 .09397 -12.52302 .99015

Employme -4.41215 3.62985 -1.21552 .22554 -11.56796 2.74367

Social_p 2.02758 .80130 2.53037 .01213 .44792 3.60725

Insuranc 3.96883 4.04902 .98019 .32812 -4.01334 11.95099

Major_Ps -5.12086 2.77707 -1.84398 .06660 -10.59553 .35380

Chemo_co -2.42188 1.70788 -1.41807 .15766 -5.78876 .94499

Mastecto -1.36714 1.61306 -.84755 .39766 -4.54709 1.81281

Radiothe -1.01011 2.93276 -.34442 .73087 -6.79170 4.77148

Standardized coefficients

coeff

Stigma_I -.45455

Tangible .13709

Age_cov -.03736

Educatio -.11354

Employme -.08267

Social_p .14627

Insuranc .05714

Major_Ps -.10973

Chemo_co -.10327

Mastecto -.05512

Radiothe -.02482

| **Benjamini-Hochberg Procedure** | | | | |
| --- | --- | --- | --- | --- |
|  |  | **Ascending P-values** | **I = ranking** | **(I/11)* 0.10** |
|  | **Stigma** | **.00000** | **1** | **.009** |
|  | **Social participation frequency** | **.012** | **2** | **.018** |
|  | **Tangible support** | **.024** | **3** | **.027** |
|  | **Major Psychological issues** | **~~.066~~** | **4** | **.03636** |
|  | **Education** | **~~.093~~** | **5** | **.045** |
|  | **Chemotherapy** | **~~.157~~** | **6** | **.054** |
|  | **Employment** | **~~.225~~** | **7** | **.063** |
|  | **Insurance** | **~~.328~~** | **8** | **.072** |
|  | **Mastectomy** | **~~.397~~** | **9** | **.081** |
|  | **Age** | **~~.524~~** | **10** | **.0909** |
|  | **Radiotherapy** | **~~.730~~** | **11** | **.100** |

************************** TOTAL EFFECT MODEL ****************************

OUTCOME VARIABLE:

FACT_B

Model Summary

R R-sq MSE F df1 df2 p

.56249 .31640 285.62775 9.71966 10.00000 210.00000 .00000

Model

coeff se t p LLCI ULCI

constant 124.01187 10.02536 12.36982 .00000 104.24862 143.77512

Stigma_I -1.94096 .24305 -7.98573 .00000 -2.42009 -1.46182

Age_cov -.06061 .12907 -.46963 .63910 -.31504 .19382

Educatio -5.65102 3.46028 -1.63311 .10394 -12.47236 1.17033

Employme -4.36095 3.66508 -1.18986 .23544 -11.58601 2.86412

Social_p 1.89265 .80684 2.34575 .01992 .30210 3.48319

Insuranc 4.87036 4.06850 1.19709 .23262 -3.14997 12.89070

Major_Ps -6.46957 2.73853 -2.36242 .01907 -11.86810 -1.07103

Chemo_co -2.47656 1.72431 -1.43626 .15242 -5.87574 .92263

Mastecto -1.60359 1.62531 -.98664 .32496 -4.80761 1.60043

Radiothe -.38631 2.94813 -.13104 .89587 -6.19803 5.42541

Standardized coefficients

coeff

Stigma_I -.47848

Age_cov -.02770

Educatio -.11127

Employme -.08171

Social_p .13653

Insuranc .07012

Major_Ps -.13863

Chemo_co -.10561

Mastecto -.06465

Radiothe -.00949

| **Benjamini-Hochberg Procedure** | | | | |
| --- | --- | --- | --- | --- |
|  |  | **Ascending P-value s** | **I = ranking** | **(I/10)* 0.10** |
|  | **Stigma** | **.00000** | **1** | **.01** |
|  | **Major Psychological issues** | **.0190** | **2** | **.02** |
|  | **Social participation frequency** | **.0199** | **3** | **.03** |
|  | **Education** | **~~.103~~** | **4** | **.04** |
|  | **Chemotherapy** | **~~.152~~** | **5** | **.05** |
|  | **Insurance** | **~~.232~~** | **6** | **.06** |
|  | **Employment** | **~~.235~~** | **7** | **.07** |
|  | **Mastectomy** | **~~.324~~** | **8** | **.08** |
|  | **Age** | **~~.639~~** | **9** | **.09** |
|  | **Radiotherapy** | **~~.898~~** | **10** | **.10** |

************** TOTAL, DIRECT, AND INDIRECT EFFECTS OF X ON Y **************

Total effect of X on Y

Effect se t p LLCI ULCI c_ps c_cs

-1.94096 .24305 -7.98573 .00000 -2.42009 -1.46182 -.09719 -.47848

Direct effect of X on Y

Effect se t p LLCI ULCI c'_ps c'_cs

-1.84386 .24452 -7.54075 .00000 -2.32590 -1.36182 -.09233 -.45455

Indirect effect(s) of X on Y:

Effect BootSE BootLLCI BootULCI

Tangible -.09710 .05586 -.21923 -.00574

Partially standardized indirect effect(s) of X on Y:

Effect BootSE BootLLCI BootULCI

Tangible -.00486 .00279 -.01092 -.00029

Completely standardized indirect effect(s) of X on Y:

Effect BootSE BootLLCI BootULCI

Tangible -.02394 .01386 -.05473 -.00137

*********************** ANALYSIS NOTES AND ERRORS ************************

Level of confidence for all confidence intervals in output:

95.0000

Number of bootstrap samples for percentile bootstrap confidence intervals:

5000

***************** PROCESS Procedure for SPSS Version 3.5 *****************

Written by Andrew F. Hayes, Ph.D. www.afhayes.com

Documentation available in Hayes (2018). www.guilford.com/p/hayes3

**************************************************************************

**Model : 4**

**Y : FACT_B**

**X : Stigma_I**

**M : Positive social interaction**

Covariates:

Age_cov Educatio Employme Social_p Insuranc Major_Ps Chemo_co Mastecto Radiothe

Sample

Size: 221

**************************************************************************

OUTCOME VARIABLE:

Positive

Model Summary

R R-sq MSE F df1 df2 p

.34064 .11604 .05623 2.75668 10.00000 210.00000 .00325

Model

coeff se t p LLCI ULCI

constant .52551 .14067 3.73583 .00024 .24821 .80281

Stigma_I -.01295 .00341 -3.79812 .00019 -.01968 -.00623

Age_cov .00060 .00181 .33404 .73868 -.00297 .00417

Educatio .01962 .04855 .40417 .68650 -.07609 .11533

Employme .02577 .05143 .50115 .61679 -.07560 .12715

Social_p .00119 .01132 .10550 .91608 -.02112 .02351

Insuranc .05715 .05709 1.00117 .31790 -.05538 .16969

Major_Ps -.08447 .03842 -2.19820 .02903 -.16021 -.00872

Chemo_co -.00020 .02419 -.00842 .99329 -.04790 .04749

Mastecto -.00918 .02281 -.40250 .68773 -.05414 .03578

Radiothe .02742 .04137 .66295 .50809 -.05412 .10897

Standardized coefficients

coeff

Stigma_I -.25878

Age_cov .02241

Educatio .03131

Employme .03914

Social_p .00698

Insuranc .06669

Major_Ps -.14669

Chemo_co -.00070

Mastecto -.02999

Radiothe .05460

| **Benjamini-Hochberg Procedure** | | | | |
| --- | --- | --- | --- | --- |
|  |  | **Ascending P-value s** | **I = ranking** | **(I/10)* 0.10** |
|  | **Stigma** | **.0001** | **1** | **.01** |
|  | **Major Psychological issues** | **~~.029~~** | **2** | **.02** |
|  | **Insurance** | **~~.317~~** | **3** | **.03** |
|  | **Radiotherapy** | **~~.508~~** | **4** | **.04** |
|  | **Employment** | **~~.616~~** | **5** | **.05** |
|  | **Education** | **~~.686~~** | **6** | **.06** |
|  | **Mastectomy** | **~~.687~~** | **7** | **.07** |
|  | **Age** | **~~.738~~** | **8** | **.08** |
|  | **Social participation frequency** | **~~.916~~** | **9** | **.09** |
|  | **Chemotherapy** | **~~.993~~** | **10** | **.10** |

**************************************************************************

OUTCOME VARIABLE:

FACT_B

Model Summary

R R-sq MSE F df1 df2 p

.61222 .37482 262.46902 11.39107 11.00000 209.00000 .00000

Model

coeff se t p LLCI ULCI

constant 113.06327 9.92455 11.39228 .00000 93.49821 132.62834

Stigma_I -1.67109 .24086 -6.93800 .00000 -2.14592 -1.19627

Positive 20.83419 4.71449 4.41918 .00002 11.54013 30.12824

Age_cov -.07322 .12376 -.59162 .55474 -.31719 .17075

Educatio -6.05985 3.31833 -1.82618 .06925 -12.60154 .48184

Employme -4.89788 3.51546 -1.39324 .16503 -11.82818 2.03242

Social_p 1.86777 .77346 2.41482 .01660 .34298 3.39255

Insuranc 3.67963 3.90937 .94123 .34767 -4.02722 11.38649

Major_Ps -4.70980 2.65519 -1.77381 .07755 -9.94420 .52459

Chemo_co -2.47231 1.65293 -1.49571 .13624 -5.73087 .78625

Mastecto -1.41235 1.55863 -.90615 .36590 -4.48500 1.66030

Radiothe -.95765 2.82904 -.33851 .73532 -6.53477 4.61946

Standardized coefficients

coeff

Stigma_I -.41196

Positive .25707

Age_cov -.03346

Educatio -.11932

Employme -.09177

Social_p .13474

Insuranc .05298

Major_Ps -.10092

Chemo_co -.10542

Mastecto -.05694

Radiothe -.02353

| **Benjamini-Hochberg Procedure** | | | | |
| --- | --- | --- | --- | --- |
|  |  | **Ascending P-value s** | **I = ranking** | **(I/11)* 0.10** |
|  | **Stigma** | **.00000** | **1** | **.009** |
|  | **Positive social interaction** | **.00002** | **2** | **.01818** |
|  | **Social participation frequency** | **.016** | **3** | **.02727** |
|  | **Education** | **~~.069~~** | **4** | **.03636** |
|  | **Major Psychological issues** | **~~.077~~** | **5** | **.045** |
|  | **Chemotherapy** | **~~.136~~** | **6** | **.054** |
|  | **Employment** | **~~.165~~** | **7** | **.063** |
|  | **Insurance** | **~~.347~~** | **8** | **.072** |
|  | **Mastectomy** | **~~.365~~** | **9** | **.081** |
|  | **Age** | **~~.554~~** | **10** | **.0909** |
|  | **Radiotherapy** | **~~.735~~** | **11** | **.100** |

************************** TOTAL EFFECT MODEL ****************************

OUTCOME VARIABLE:

FACT_B

Model Summary

R R-sq MSE F df1 df2 p

.56249 .31640 285.62775 9.71966 10.00000 210.00000 .00000

Model

coeff se t p LLCI ULCI

constant 124.01187 10.02536 12.36982 .00000 104.24862 143.77512

Stigma_I -1.94096 .24305 -7.98573 .00000 -2.42009 -1.46182

Age_cov -.06061 .12907 -.46963 .63910 -.31504 .19382

Educatio -5.65102 3.46028 -1.63311 .10394 -12.47236 1.17033

Employme -4.36095 3.66508 -1.18986 .23544 -11.58601 2.86412

Social_p 1.89265 .80684 2.34575 .01992 .30210 3.48319

Insuranc 4.87036 4.06850 1.19709 .23262 -3.14997 12.89070

Major_Ps -6.46957 2.73853 -2.36242 .01907 -11.86810 -1.07103

Chemo_co -2.47656 1.72431 -1.43626 .15242 -5.87574 .92263

Mastecto -1.60359 1.62531 -.98664 .32496 -4.80761 1.60043

Radiothe -.38631 2.94813 -.13104 .89587 -6.19803 5.42541

Standardized coefficients

coeff

Stigma_I -.47848

Age_cov -.02770

Educatio -.11127

Employme -.08171

Social_p .13653

Insuranc .07012

Major_Ps -.13863

Chemo_co -.10561

Mastecto -.06465

Radiothe -.00949

| **Benjamini-Hochberg Procedure** | | | | |
| --- | --- | --- | --- | --- |
|  |  | **Ascending P-value s** | **I = ranking** | **(I/10)* 0.10** |
|  | **Stigma** | **.00000** | **1** | **.01** |
|  | **Major Psychological issues** | **.0190** | **2** | **.02** |
|  | **Social participation frequency** | **.0199** | **3** | **.03** |
|  | **Education** | **~~.103~~** | **4** | **.04** |
|  | **Chemotherapy** | **~~.152~~** | **5** | **.05** |
|  | **Insurance** | **~~.232~~** | **6** | **.06** |
|  | **Employment** | **~~.235~~** | **7** | **.07** |
|  | **Mastectomy** | **~~.324~~** | **8** | **.08** |
|  | **Age** | **~~.639~~** | **9** | **.09** |
|  | **Radiotherapy** | **~~.898~~** | **10** | **.10** |

************** TOTAL, DIRECT, AND INDIRECT EFFECTS OF X ON Y **************

Total effect of X on Y

Effect se t p LLCI ULCI c_ps c_cs

-1.94096 .24305 -7.98573 .00000 -2.42009 -1.46182 -.09719 -.47848

Direct effect of X on Y

Effect se t p LLCI ULCI c'_ps c'_cs

-1.67109 .24086 -6.93800 .00000 -2.14592 -1.19627 -.08368 -.41196

Indirect effect(s) of X on Y:

Effect BootSE BootLLCI BootULCI

Positive -.26986 .08733 -.44743 -.11000

Partially standardized indirect effect(s) of X on Y:

Effect BootSE BootLLCI BootULCI

Positive -.01351 .00431 -.02230 -.00565

Completely standardized indirect effect(s) of X on Y:

Effect BootSE BootLLCI BootULCI

Positive -.06653 .02176 -.11057 -.02645

*********************** ANALYSIS NOTES AND ERRORS ************************

Level of confidence for all confidence intervals in output:

95.0000

Number of bootstrap samples for percentile bootstrap confidence intervals:

5000

***************** PROCESS Procedure for SPSS Version 3.5 *****************

Written by Andrew F. Hayes, Ph.D. www.afhayes.com

Documentation available in Hayes (2018). www.guilford.com/p/hayes3

**************************************************************************

**Model : 4**

**Y : FACT_B**

**X : Stigma_I**

**M : MOS_SSS = total social support**

Covariates:

Age_cov Educatio Employme Social_p Insuranc Major_Ps Chemo_co Mastecto Radiothe

Sample

Size: 221

**************************************************************************

OUTCOME VARIABLE:

MOS_SSS

Model Summary

R R-sq MSE F df1 df2 p

.35287 .12451 .06905 2.98667 10.00000 210.00000 .00153

Model

coeff se t p LLCI ULCI

constant .46456 .15587 2.98037 .00322 .15728 .77183

Stigma_I -.01095 .00378 -2.89783 .00416 -.01840 -.00350

Age_cov .00169 .00201 .84386 .39971 -.00226 .00565

Educatio -.03744 .05380 -.69584 .48730 -.14349 .06862

Employme -.02411 .05698 -.42304 .67270 -.13644 .08823

Social_p -.00090 .01254 -.07168 .94292 -.02563 .02383

Insuranc .07701 .06326 1.21750 .22478 -.04768 .20171

Major_Ps -.12186 .04258 -2.86202 .00464 -.20579 -.03792

Chemo_co -.00198 .02681 -.07371 .94131 -.05483 .05087

Mastecto -.01605 .02527 -.63501 .52612 -.06586 .03377

Radiothe .05665 .04584 1.23597 .21785 -.03371 .14701

Standardized coefficients

coeff

Stigma_I -.19649

Age_cov .05633

Educatio -.05365

Employme -.03288

Social_p -.00472

Insuranc .08071

Major_Ps -.19006

Chemo_co -.00613

Mastecto -.04709

Radiothe .10131

| **Benjamini-Hochberg Procedure** | | | | |
| --- | --- | --- | --- | --- |
|  |  | **Ascending P-value s** | **I = ranking** | **(I/10)* 0.10** |
|  | **Stigma** | **.0041** | **1** | **.01** |
|  | **Major Psychological issues** | **.0046** | **2** | **.02** |
|  | **Radiotherapy** | **~~.217~~** | **3** | **.03** |
|  | **Insurance** | **~~.224~~** | **4** | **.04** |
|  | **Age** | **~~.399~~** | **5** | **.05** |
|  | **Education** | **~~.487~~** | **6** | **.06** |
|  | **Mastectomy** | **~~.526~~** | **7** | **.07** |
|  | **Employment** | **~~.672~~** | **8** | **.08** |
|  | **Chemotherapy** | **~~.941~~** | **9** | **.09** |
|  | **Social participation frequency** | **~~.942~~** | **10** | **.10** |

**************************************************************************

OUTCOME VARIABLE:

FACT_B

Model Summary

R R-sq MSE F df1 df2 p

.59707 .35650 270.15967 10.52593 11.00000 209.00000 .00000

Model

coeff se t p LLCI ULCI

constant 116.77525 9.95419 11.73126 .00000 97.15175 136.39874

Stigma_I -1.77037 .24106 -7.34411 .00000 -2.24559 -1.29515

MOS_SSS 15.57751 4.31650 3.60882 .00038 7.06804 24.08698

Age_cov -.08699 .12574 -.69187 .48979 -.33486 .16088

Educatio -5.06786 3.36916 -1.50419 .13404 -11.70976 1.57404

Employme -3.98543 3.56598 -1.11763 .26501 -11.01532 3.04446

Social_p 1.90666 .78470 2.42979 .01595 .35972 3.45360

Insuranc 3.67067 3.97074 .92443 .35633 -4.15716 11.49851

Major_Ps -4.57131 2.71479 -1.68385 .09370 -9.92319 .78058

Chemo_co -2.44577 1.67699 -1.45843 .14622 -5.75177 .86022

Mastecto -1.35363 1.58221 -.85553 .39324 -4.47276 1.76550

Radiothe -1.26882 2.87760 -.44093 .65972 -6.94166 4.40402

Standardized coefficients

coeff

Stigma_I -.43643

MOS_SSS .21401

Age_cov -.03976

Educatio -.09979

Employme -.07468

Social_p .13754

Insuranc .05285

Major_Ps -.09796

Chemo_co -.10429

Mastecto -.05458

Radiothe -.03117

| **Benjamini-Hochberg Procedure** | | | | |
| --- | --- | --- | --- | --- |
|  |  | **Ascending P-value s** | **I = ranking** | **(I/11)* 0.10** |
|  | **Stigma** | **.00000** | **1** | **.009** |
|  | **Total social support** | **.00038** | **2** | **.01818** |
|  | **Social participation frequency** | **.015** | **3** | **.02727** |
|  | **Major Psychological issues** | **~~.093~~** | **4** | **.03636** |
|  | **Education** | **~~.134~~** | **5** | **.045** |
|  | **Chemotherapy** | **~~.146~~** | **6** | **.054** |
|  | **Employment** | **~~.265~~** | **7** | **.063** |
|  | **Insurance** | **~~.356~~** | **8** | **.072** |
|  | **Mastectomy** | **~~.393~~** | **9** | **.081** |
|  | **Age** | **~~.489~~** | **10** | **.0909** |
|  | **Radiotherapy** | **~~.659~~** | **11** | **.100** |

************************** TOTAL EFFECT MODEL ****************************

OUTCOME VARIABLE:

FACT_B

Model Summary

R R-sq MSE F df1 df2 p

.56249 .31640 285.62775 9.71966 10.00000 210.00000 .00000

Model

coeff se t p LLCI ULCI

constant 124.01187 10.02536 12.36982 .00000 104.24862 143.77512

Stigma_I -1.94096 .24305 -7.98573 .00000 -2.42009 -1.46182

Age_cov -.06061 .12907 -.46963 .63910 -.31504 .19382

Educatio -5.65102 3.46028 -1.63311 .10394 -12.47236 1.17033

Employme -4.36095 3.66508 -1.18986 .23544 -11.58601 2.86412

Social_p 1.89265 .80684 2.34575 .01992 .30210 3.48319

Insuranc 4.87036 4.06850 1.19709 .23262 -3.14997 12.89070

Major_Ps -6.46957 2.73853 -2.36242 .01907 -11.86810 -1.07103

Chemo_co -2.47656 1.72431 -1.43626 .15242 -5.87574 .92263

Mastecto -1.60359 1.62531 -.98664 .32496 -4.80761 1.60043

Radiothe -.38631 2.94813 -.13104 .89587 -6.19803 5.42541

Standardized coefficients

coeff

Stigma_I -.47848

Age_cov -.02770

Educatio -.11127

Employme -.08171

Social_p .13653

Insuranc .07012

Major_Ps -.13863

Chemo_co -.10561

Mastecto -.06465

Radiothe -.00949

| **Benjamini-Hochberg Procedure** | | | | |
| --- | --- | --- | --- | --- |
|  |  | **Ascending P-value s** | **I = ranking** | **(I/10)* 0.10** |
|  | **Stigma** | **.00000** | **1** | **.01** |
|  | **Major Psychological issues** | **.0190** | **2** | **.02** |
|  | **Social participation frequency** | **.0199** | **3** | **.03** |
|  | **Education** | **~~.103~~** | **4** | **.04** |
|  | **Chemotherapy** | **~~.152~~** | **5** | **.05** |
|  | **Insurance** | **~~.232~~** | **6** | **.06** |
|  | **Employment** | **~~.235~~** | **7** | **.07** |
|  | **Mastectomy** | **~~.324~~** | **8** | **.08** |
|  | **Age** | **~~.639~~** | **9** | **.09** |
|  | **Radiotherapy** | **~~.898~~** | **10** | **.10** |

************** TOTAL, DIRECT, AND INDIRECT EFFECTS OF X ON Y **************

Total effect of X on Y

Effect se t p LLCI ULCI c_ps c_cs

-1.94096 .24305 -7.98573 .00000 -2.42009 -1.46182 -.09719 -.47848

Direct effect of X on Y

Effect se t p LLCI ULCI c'_ps c'_cs

-1.77037 .24106 -7.34411 .00000 -2.24559 -1.29515 -.08865 -.43643

Indirect effect(s) of X on Y:

Effect BootSE BootLLCI BootULCI

MOS_SSS -.17058 .06914 -.31470 -.04606

Partially standardized indirect effect(s) of X on Y:

Effect BootSE BootLLCI BootULCI

MOS_SSS -.00854 .00342 -.01574 -.00237

Completely standardized indirect effect(s) of X on Y:

Effect BootSE BootLLCI BootULCI

MOS_SSS -.04205 .01717 -.07799 -.01134

*********************** ANALYSIS NOTES AND ERRORS ************************

Level of confidence for all confidence intervals in output:

95.0000

Number of bootstrap samples for percentile bootstrap confidence intervals:

5000

***************** PROCESS Procedure for SPSS Version 3.5 *****************

Written by Andrew F. Hayes, Ph.D. www.afhayes.com

Documentation available in Hayes (2018). www.guilford.com/p/hayes3

**************************************************************************

**Model : 4**

**Y : FACT_B**

**X : Stigma_I**

**M : CopPosit = Positive reframing coping**

Covariates:

Age_cov Educatio Employme Social_p Insuranc Major_Ps Chemo_co Mastecto Radiothe

Sample

Size: 219

**************************************************************************

OUTCOME VARIABLE:

CopPosit

Model Summary

R R-sq MSE F df1 df2 p

.33083 .10945 .80153 2.55639 10.00000 208.00000 .00623

Model

coeff se t p LLCI ULCI

constant 3.43955 .53168 6.46918 .00000 2.39137 4.48773

Stigma_I -.04736 .01300 -3.64170 .00034 -.07299 -.02172

Age_cov -.00942 .00691 -1.36268 .17446 -.02305 .00421

Educatio -.04722 .18338 -.25752 .79703 -.40875 .31430

Employme .10310 .19421 .53086 .59608 -.27977 .48596

Social_p -.02313 .04283 -.54008 .58972 -.10756 .06130

Insuranc .01560 .21558 .07238 .94237 -.40939 .44060

Major_Ps -.06339 .14611 -.43382 .66487 -.35143 .22466

Chemo_co -.03581 .09155 -.39120 .69605 -.21630 .14467

Mastecto -.01352 .08634 -.15663 .87569 -.18374 .15669

Radiothe .25174 .15646 1.60896 .10914 -.05671 .56018

Standardized coefficients

coeff

Stigma_I -.25100

Age_cov -.09232

Educatio -.02011

Employme .04178

Social_p -.03606

Insuranc .00486

Major_Ps -.02917

Chemo_co -.03285

Mastecto -.01172

Radiothe .13323

| **Benjamini-Hochberg Procedure** | | | | |
| --- | --- | --- | --- | --- |
|  |  | **Ascending P-value s** | **I = ranking** | **(I/10)* 0.10** |
|  | **Stigma** | **.0003** | **1** | **.01** |
|  | **Radiotherapy** | **~~.109~~** | **2** | **.02** |
|  | **Age** | **~~.174~~** | **3** | **.03** |
|  | **Social participation frequency** | **~~.589~~** | **4** | **.04** |
|  | **Employment** | **~~.596~~** | **5** | **.05** |
|  | **Major Psychological issues** | **~~.664~~** | **6** | **.06** |
|  | **Chemotherapy** | **~~.696~~** | **7** | **.07** |
|  | **Education** | **~~.797~~** | **8** | **.08** |
|  | **Mastectomy** | **~~.875~~** | **9** | **.09** |
|  | **Insurance** | **~~.942~~** | **10** | **.10** |

**************************************************************************

OUTCOME VARIABLE:

FACT_B

Model Summary

R R-sq MSE F df1 df2 p

.59672 .35607 271.80092 10.40592 11.00000 207.00000 .00000

Model

coeff se t p LLCI ULCI

constant 108.43610 10.73069 10.10524 .00000 87.28065 129.59155

Stigma_I -1.75683 .24698 -7.11313 .00000 -2.24376 -1.26990

CopPosit 4.52948 1.27683 3.54743 .00048 2.01221 7.04675

Age_cov .00031 .12786 .00246 .99804 -.25175 .25238

Educatio -5.38262 3.37745 -1.59369 .11253 -12.04124 1.27600

Employme -4.76776 3.57868 -1.33227 .18424 -11.82309 2.28758

Social_p 1.96182 .78921 2.48581 .01372 .40590 3.51773

Insuranc 4.80697 3.96983 1.21088 .22732 -3.01952 12.63347

Major_Ps -6.39392 2.69181 -2.37533 .01845 -11.70080 -1.08704

Chemo_co -2.42906 1.68647 -1.44032 .15129 -5.75392 .89580

Mastecto -1.45467 1.59007 -.91485 .36134 -4.58947 1.68014

Radiothe -1.70245 2.89903 -.58725 .55768 -7.41785 4.01296

Standardized coefficients

coeff

Stigma_I -.43102

CopPosit .20966

Age_cov .00014

Educatio -.10609

Employme -.08944

Social_p .14157

Insuranc .06933

Major_Ps -.13621

Chemo_co -.10314

Mastecto -.05836

Radiothe -.04171

| **Benjamini-Hochberg Procedure** | | | | |
| --- | --- | --- | --- | --- |
|  |  | **Ascending P-values** | **I = ranking** | **(I/11)* 0.10** |
|  | **Stigma** | **.00000** | **1** | **.009** |
|  | **Positive reframing** | **.0004** | **2** | **.01818** |
|  | **Social participation frequency** | **.013** | **3** | **.02727** |
|  | **Major Psychological issues** | **.018** | **4** | **.03636** |
|  | **Education** | **~~.112~~** | **5** | **.045** |
|  | **Employment** | **~~.184~~** | **6** | **.054** |
|  | **Chemotherapy** | **~~.151~~** | **7** | **.063** |
|  | **Mastectomy** | **~~.361~~** | **8** | **.072** |
|  | **Insurance** | **~~.227~~** | **9** | **.081** |
|  | **Radiotherapy** | **~~.557~~** | **10** | **.0909** |
|  | **Age** | **~~.998~~** | **11** | **.100** |

************************** TOTAL EFFECT MODEL ****************************

OUTCOME VARIABLE:

FACT_B

Model Summary

R R-sq MSE F df1 df2 p

.56296 .31693 286.93846 9.65061 10.00000 208.00000 .00000

Model

coeff se t p LLCI ULCI

constant 124.01548 10.05977 12.32786 .00000 104.18329 143.84767

Stigma_I -1.97133 .24605 -8.01205 .00000 -2.45640 -1.48627

Age_cov -.04235 .13079 -.32381 .74640 -.30019 .21549

Educatio -5.59652 3.46968 -1.61298 .10826 -12.43676 1.24372

Employme -4.30078 3.67450 -1.17044 .24316 -11.54482 2.94325

Social_p 1.85705 .81032 2.29175 .02292 .25956 3.45454

Insuranc 4.87765 4.07883 1.19584 .23312 -3.16351 12.91880

Major_Ps -6.68103 2.76450 -2.41672 .01652 -12.13106 -1.23099

Chemo_co -2.59127 1.73216 -1.49598 .13617 -6.00611 .82357

Mastecto -1.51592 1.63365 -.92794 .35452 -4.73655 1.70471

Radiothe -.56221 2.96030 -.18992 .84956 -6.39825 5.27382

Standardized coefficients

coeff

Stigma_I -.48364

Age_cov -.01921

Educatio -.11031

Employme -.08068

Social_p .13401

Insuranc .07035

Major_Ps -.14233

Chemo_co -.11003

Mastecto -.06082

Radiothe -.01377

| **Benjamini-Hochberg Procedure** | | | | |
| --- | --- | --- | --- | --- |
|  |  | **Ascending P-value s** | **I = ranking** | **(I/10)* 0.10** |
|  | **Stigma** | **.00000** | **1** | **.01** |
|  | **Major Psychological issues** | **.016** | **2** | **.02** |
|  | **Social participation frequency** | **.022** | **3** | **.03** |
|  | **Education** | **~~.108~~** | **4** | **.04** |
|  | **Chemotherapy** | **~~.136~~** | **5** | **.05** |
|  | **Insurance** | **~~.233~~** | **6** | **.06** |
|  | **Employment** | **~~.234~~** | **7** | **.07** |
|  | **Mastectomy** | **~~.354~~** | **8** | **.08** |
|  | **Age** | **~~.746~~** | **9** | **.09** |
|  | **Radiotherapy** | **~~.849~~** | **10** | **.10** |

************** TOTAL, DIRECT, AND INDIRECT EFFECTS OF X ON Y **************

Total effect of X on Y

Effect se t p LLCI ULCI c_ps c_cs

-1.97133 .24605 -8.01205 .00000 -2.45640 -1.48627 -.09847 -.48364

Direct effect of X on Y

Effect se t p LLCI ULCI c'_ps c'_cs

-1.75683 .24698 -7.11313 .00000 -2.24376 -1.26990 -.08775 -.43102

Indirect effect(s) of X on Y:

Effect BootSE BootLLCI BootULCI

CopPosit -.21450 .08352 -.39547 -.06385

Partially standardized indirect effect(s) of X on Y:

Effect BootSE BootLLCI BootULCI

CopPosit -.01071 .00411 -.01948 -.00340

Completely standardized indirect effect(s) of X on Y:

Effect BootSE BootLLCI BootULCI

CopPosit -.05263 .02055 -.09600 -.01626

*********************** ANALYSIS NOTES AND ERRORS ************************

Level of confidence for all confidence intervals in output:

95.0000

Number of bootstrap samples for percentile bootstrap confidence intervals:

5000

***************** PROCESS Procedure for SPSS Version 3.5 *****************

Written by Andrew F. Hayes, Ph.D. www.afhayes.com

Documentation available in Hayes (2018). www.guilford.com/p/hayes3

**************************************************************************

**Model : 4**

**Y : FACT_B**

**X : Stigma_I**

**M : Activeco = active coping**

Covariates:

Age_cov Educatio Employme Social_p Insuranc Major_Ps Chemo_co Mastecto Radiothe

Sample

Size: 221

**************************************************************************

OUTCOME VARIABLE:

Activeco

Model Summary

R R-sq MSE F df1 df2 p

.26036 .06779 .67205 1.52706 10.00000 210.00000 .13138

Model

coeff se t p LLCI ULCI

constant 2.93761 .48630 6.04078 .00000 1.97896 3.89626

Stigma_I -.03310 .01179 -2.80723 .00547 -.05634 -.00986

Age_cov .00261 .00626 .41677 .67727 -.00973 .01495

Educatio -.12851 .16785 -.76565 .44474 -.45939 .20237

Employme .10211 .17778 .57436 .56634 -.24835 .45257

Social_p .03567 .03914 .91135 .36316 -.04148 .11282

Insuranc .14093 .19735 .71410 .47596 -.24811 .52997

Major_Ps -.10039 .13284 -.75575 .45064 -.36226 .16147

Chemo_co .02878 .08364 .34409 .73112 -.13610 .19366

Mastecto .13340 .07884 1.69201 .09213 -.02202 .28881

Radiothe -.07485 .14300 -.52343 .60123 -.35676 .20706

Standardized coefficients

coeff

Stigma_I -.19642

Age_cov .02871

Educatio -.06092

Employme .04606

Social_p .06194

Insuranc .04885

Major_Ps -.05179

Chemo_co .02954

Mastecto .12948

Radiothe -.04427

| **Benjamini-Hochberg Procedure** | | | | |
| --- | --- | --- | --- | --- |
|  |  | **Ascending P-value s** | **I = ranking** | **(I/10)* 0.10** |
|  | **Stigma** | **.005** | **1** | **.01** |
|  | **Mastectomy** | **~~.092~~** | **2** | **.02** |
|  | **Social participation frequency** | **~~.363~~** | **3** | **.03** |
|  | **Education** | **~~.444~~** | **4** | **.04** |
|  | **Major Psychological issues** | **~~.450~~** | **5** | **.05** |
|  | **Insurance** | **~~.475~~** | **6** | **.06** |
|  | **Employment** | **~~.566~~** | **7** | **.07** |
|  | **Radiotherapy** | **~~.601~~** | **8** | **.08** |
|  | **Age** | **~~.677~~** | **9** | **.09** |
|  | **Chemotherapy** | **~~.731~~** | **10** | **.10** |

1**************************************************************************

OUTCOME VARIABLE:

FACT_B

Model Summary

R R-sq MSE F df1 df2 p

.57540 .33108 280.82883 9.40419 11.00000 209.00000 .00000

Model

coeff se t p LLCI ULCI

constant 115.13536 10.76990 10.69048 .00000 93.90379 136.36692

Stigma_I -1.84095 .24548 -7.49930 .00000 -2.32489 -1.35701

Activeco 3.02168 1.41062 2.14209 .03334 .24081 5.80254

Age_cov -.06850 .12803 -.53501 .59321 -.32089 .18390

Educatio -5.26269 3.43588 -1.53169 .12711 -12.03612 1.51073

Employme -4.66949 3.63701 -1.28388 .20060 -11.83943 2.50044

Social_p 1.78487 .80161 2.22660 .02704 .20459 3.36516

Insuranc 4.44453 4.03907 1.10038 .27243 -3.51801 12.40707

Major_Ps -6.16621 2.71912 -2.26773 .02437 -11.52663 -.80580

Chemo_co -2.56352 1.71025 -1.49892 .13540 -5.93507 .80803

Mastecto -2.00667 1.62255 -1.23674 .21757 -5.20533 1.19199

Radiothe -.16013 2.92516 -.05474 .95640 -5.92674 5.60648

Standardized coefficients

coeff

Stigma_I -.45383

Activeco .12551

Age_cov -.03131

Educatio -.10362

Employme -.08749

Social_p .12876

Insuranc .06399

Major_Ps -.13213

Chemo_co -.10931

Mastecto -.08091

Radiothe -.00393

| **Benjamini-Hochberg Procedure** | | | | |
| --- | --- | --- | --- | --- |
|  |  | **Ascending P-values** | **I = ranking** | **(I/11)* 0.10** |
|  | **Stigma** | **.00000** | **1** | **.009** |
|  | **Major Psychological issues** | **.024** | **2** | **.01818** |
|  | **Social participation frequency** | **.027** | **3** | **.02727** |
|  | **Ative coping** | **.033** | **4** | **.03636** |
|  | **Education** | **~~.127~~** | **5** | **.045** |
|  | **Chemotherapy** | **~~.135~~** | **6** | **.054** |
|  | **Employment** | **~~.200~~** | **7** | **.063** |
|  | **Mastectomy** | **~~.217~~** | **8** | **.072** |
|  | **Insurance** | **~~.272~~** | **9** | **.081** |
|  | **Age** | **~~.593~~** | **10** | **.0909** |
|  | **Radiotherapy** | **~~.956~~** | **11** | **.100** |

************************** TOTAL EFFECT MODEL ****************************

OUTCOME VARIABLE:

FACT_B

Model Summary

R R-sq MSE F df1 df2 p

.56249 .31640 285.62775 9.71966 10.00000 210.00000 .00000

Model

coeff se t p LLCI ULCI

constant 124.01187 10.02536 12.36982 .00000 104.24862 143.77512

Stigma_I -1.94096 .24305 -7.98573 .00000 -2.42009 -1.46182

Age_cov -.06061 .12907 -.46963 .63910 -.31504 .19382

Educatio -5.65102 3.46028 -1.63311 .10394 -12.47236 1.17033

Employme -4.36095 3.66508 -1.18986 .23544 -11.58601 2.86412

Social_p 1.89265 .80684 2.34575 .01992 .30210 3.48319

Insuranc 4.87036 4.06850 1.19709 .23262 -3.14997 12.89070

Major_Ps -6.46957 2.73853 -2.36242 .01907 -11.86810 -1.07103

Chemo_co -2.47656 1.72431 -1.43626 .15242 -5.87574 .92263

Mastecto -1.60359 1.62531 -.98664 .32496 -4.80761 1.60043

Radiothe -.38631 2.94813 -.13104 .89587 -6.19803 5.42541

Standardized coefficients

coeff

Stigma_I -.47848

Age_cov -.02770

Educatio -.11127

Employme -.08171

Social_p .13653

Insuranc .07012

Major_Ps -.13863

Chemo_co -.10561

Mastecto -.06465

Radiothe -.00949

| **Benjamini-Hochberg Procedure** | | | | |
| --- | --- | --- | --- | --- |
|  |  | **Ascending P-value s** | **I = ranking** | **(I/10)* 0.10** |
|  | **Stigma** | **.00000** | **1** | **.01** |
|  | **Major Psychological issues** | **.0190** | **2** | **.02** |
|  | **Social participation frequency** | **.0199** | **3** | **.03** |
|  | **Education** | **~~.103~~** | **4** | **.04** |
|  | **Chemotherapy** | **~~.152~~** | **5** | **.05** |
|  | **Insurance** | **~~.232~~** | **6** | **.06** |
|  | **Employment** | **~~.235~~** | **7** | **.07** |
|  | **Mastectomy** | **~~.324~~** | **8** | **.08** |
|  | **Age** | **~~.639~~** | **9** | **.09** |
|  | **Radiotherapy** | **~~.898~~** | **10** | **.10** |

************** TOTAL, DIRECT, AND INDIRECT EFFECTS OF X ON Y **************

Total effect of X on Y

Effect se t p LLCI ULCI c_ps c_cs

-1.94096 .24305 -7.98573 .00000 -2.42009 -1.46182 -.09719 -.47848

Direct effect of X on Y

Effect se t p LLCI ULCI c'_ps c'_cs

-1.84095 .24548 -7.49930 .00000 -2.32489 -1.35701 -.09218 -.45383

Indirect effect(s) of X on Y:

Effect BootSE BootLLCI BootULCI

Activeco -.10001 .06448 -.25010 -.00332

Partially standardized indirect effect(s) of X on Y:

Effect BootSE BootLLCI BootULCI

Activeco -.00501 .00322 -.01244 -.00018

Completely standardized indirect effect(s) of X on Y:

Effect BootSE BootLLCI BootULCI

Activeco -.02465 .01582 -.06126 -.00088

*********************** ANALYSIS NOTES AND ERRORS ************************

Level of confidence for all confidence intervals in output:

95.0000

Number of bootstrap samples for percentile bootstrap confidence intervals:

5000

***************** PROCESS Procedure for SPSS Version 3.5 *****************

Written by Andrew F. Hayes, Ph.D. www.afhayes.com

Documentation available in Hayes (2018). www.guilford.com/p/hayes3

**************************************************************************

**Model : 4**

**Y : FACT_B**

**X : Stigma_I**

**M : copAccep = acceptance coping**

Covariates:

Age_cov Educatio Employme Social_p Insuranc Major_Ps Chemo_co Mastecto Radiothe

Sample

Size: 220

**************************************************************************

OUTCOME VARIABLE:

copAccep

Model Summary

R R-sq MSE F df1 df2 p

.32195 .10365 .05680 2.41687 10.00000 209.00000 .00968

Model

coeff se t p LLCI ULCI

constant .51797 .14152 3.65996 .00032 .23897 .79696

Stigma_I -.00740 .00345 -2.14869 .03281 -.01420 -.00061

Age_cov .00317 .00182 1.74118 .08312 -.00042 .00676

Educatio -.03797 .04880 -.77799 .43745 -.13418 .05824

Employme .08544 .05170 1.65274 .09988 -.01647 .18735

Social_p -.01322 .01138 -1.16155 .24675 -.03566 .00922

Insuranc .01042 .05739 .18149 .85616 -.10271 .12354

Major_Ps .00045 .03885 .01156 .99079 -.07615 .07705

Chemo_co -.04830 .02433 -1.98533 .04842 -.09627 -.00034

Mastecto -.04955 .02297 -2.15714 .03214 -.09483 -.00427

Radiothe .05223 .04159 1.25566 .21064 -.02977 .13423

Standardized coefficients

coeff

Stigma_I -.14785

Age_cov .11782

Educatio -.06082

Employme .13025

Social_p -.07757

Insuranc .01220

Major_Ps .00078

Chemo_co -.16717

Mastecto -.16199

Radiothe .10428

| **Benjamini-Hochberg Procedure** | | | | |
| --- | --- | --- | --- | --- |
|  |  | **Ascending P-value s** | **I = ranking** | **(I/10)* 0.10** |
|  | **Mastectomy** | **~~.0321~~** | **1** | **.01** |
|  | **Stigma** | **~~.0328~~** | **2** | **.02** |
|  | **Chemotherapy** | **~~.048~~** | **3** | **.03** |
|  | **Age** | **~~.083~~** | **4** | **.04** |
|  | **Employment** | **~~.099~~** | **5** | **.05** |
|  | **Radiotherapy** | **~~.210~~** | **6** | **.06** |
|  | **Social participation frequency** | **~~.246~~** | **7** | **.07** |
|  | **Education** | **~~.437~~** | **8** | **.08** |
|  | **Insurance** | **~~.856~~** | **9** | **.09** |
|  | **Major Psychological issues** | **~~.990~~** | **10** | **.10** |

**************************************************************************

OUTCOME VARIABLE:

FACT_B

Model Summary

R R-sq MSE F df1 df2 p

.58432 .34143 276.83100 9.80335 11.00000 208.00000 .00000

Model

coeff se t p LLCI ULCI

constant 116.81455 10.19186 11.46155 .00000 96.72195 136.90714

Stigma_I -1.84342 .24320 -7.57992 .00000 -2.32287 -1.36397

copAccep 14.15125 4.82908 2.93043 .00376 4.63103 23.67146

Age_cov -.10597 .12799 -.82792 .40867 -.35830 .14636

Educatio -5.13291 3.41215 -1.50431 .13402 -11.85974 1.59391

Employme -5.54777 3.63251 -1.52726 .12822 -12.70902 1.61348

Social_p 2.08733 .79730 2.61800 .00949 .51551 3.65916

Insuranc 4.74853 4.00662 1.18517 .23730 -3.15027 12.64733

Major_Ps -6.56335 2.71252 -2.41965 .01640 -11.91091 -1.21579

Chemo_co -1.81008 1.71449 -1.05575 .29231 -5.19009 1.56994

Mastecto -.87128 1.62137 -.53738 .59158 -4.06770 2.32514

Radiothe -1.15256 2.91479 -.39542 .69294 -6.89888 4.59376

Standardized coefficients

coeff

Stigma_I -.45307

copAccep .17417

Age_cov -.04848

Educatio -.10119

Employme -.10409

Social_p .15071

Insuranc .06848

Major_Ps -.13987

Chemo_co -.07710

Mastecto -.03506

Radiothe -.02832

| **Benjamini-Hochberg Procedure** | | | | |
| --- | --- | --- | --- | --- |
|  |  | **Ascending P-values** | **I = ranking** | **(I/11)* 0.10** |
|  | **Stigma** | **.00000** | **1** | **.009** |
|  | **Acceptance** | **.0037** | **2** | **.01818** |
|  | **Social participation frequency** | **.009** | **3** | **.02727** |
|  | **Major Psychological issues** | **.016** | **4** | **.03636** |
|  | **Employment** | **~~.128~~** | **5** | **.045** |
|  | **Education** | **~~.134~~** | **6** | **.054** |
|  | **Insurance** | **~~.237~~** | **7** | **.063** |
|  | **Chemotherapy** | **~~.292~~** | **8** | **.072** |
|  | **Age** | **~~.408~~** | **9** | **.081** |
|  | **Mastectomy** | **~~.591~~** | **10** | **.0909** |
|  | **Radiotherapy** | **~~.692~~** | **11** | **.100** |

************************** TOTAL EFFECT MODEL ****************************

OUTCOME VARIABLE:

FACT_B

Model Summary

R R-sq MSE F df1 df2 p

.56057 .31424 286.88088 9.57726 10.00000 209.00000 .00000

Model

coeff se t p LLCI ULCI

constant 124.14444 10.05790 12.34298 .00000 104.31650 143.97239

Stigma_I -1.94820 .24488 -7.95561 .00000 -2.43096 -1.46544

Age_cov -.06112 .12936 -.47246 .63709 -.31614 .19390

Educatio -5.67023 3.46851 -1.63477 .10360 -12.50799 1.16752

Employme -4.33871 3.67392 -1.18095 .23897 -11.58141 2.90400

Social_p 1.90021 .80904 2.34874 .01977 .30530 3.49513

Insuranc 4.89592 4.07838 1.20046 .23132 -3.14412 12.93595

Major_Ps -6.55700 2.76132 -2.37459 .01847 -12.00060 -1.11339

Chemo_co -2.49362 1.72911 -1.44214 .15076 -5.90236 .91511

Mastecto -1.57247 1.63246 -.96325 .33653 -4.79068 1.64573

Radiothe -.41346 2.95610 -.13987 .88890 -6.24105 5.41413

Standardized coefficients

coeff

Stigma_I -.47882

Age_cov -.02796

Educatio -.11179

Employme -.08140

Social_p .13720

Insuranc .07060

Major_Ps -.13974

Chemo_co -.10622

Mastecto -.06327

Radiothe -.01016

| **Benjamini-Hochberg Procedure** | | | | |
| --- | --- | --- | --- | --- |
|  |  | **Ascending P-value s** | **I = ranking** | **(I/10)* 0.10** |
|  | **Stigma** | **.00000** | **1** | **.01** |
|  | **Major Psychological issues** | **.018** | **2** | **.02** |
|  | **Social participation frequency** | **.019** | **3** | **.03** |
|  | **Education** | **~~.103~~** | **4** | **.04** |
|  | **Chemotherapy** | **~~.150~~** | **5** | **.05** |
|  | **Insurance** | **~~.231~~** | **6** | **.06** |
|  | **Employment** | **~~.238~~** | **7** | **.07** |
|  | **Mastectomy** | **~~.336~~** | **8** | **.08** |
|  | **Age** | **~~.637~~** | **9** | **.09** |
|  | **Radiotherapy** | **~~.888~~** | **10** | **.10** |

************** TOTAL, DIRECT, AND INDIRECT EFFECTS OF X ON Y **************

Total effect of X on Y

Effect se t p LLCI ULCI c_ps c_cs

-1.94820 .24488 -7.95561 .00000 -2.43096 -1.46544 -.09750 -.47882

Direct effect of X on Y

Effect se t p LLCI ULCI c'_ps c'_cs

-1.84342 .24320 -7.57992 .00000 -2.32287 -1.36397 -.09226 -.45307

Indirect effect(s) of X on Y:

Effect BootSE BootLLCI BootULCI

copAccep -.10477 .05936 -.23310 -.00476

Partially standardized indirect effect(s) of X on Y:

Effect BootSE BootLLCI BootULCI

copAccep -.00524 .00295 -.01164 -.00024

Completely standardized indirect effect(s) of X on Y:

Effect BootSE BootLLCI BootULCI

copAccep -.02575 .01447 -.05701 -.00121

*********************** ANALYSIS NOTES AND ERRORS ************************

Level of confidence for all confidence intervals in output:

95.0000

Number of bootstrap samples for percentile bootstrap confidence intervals:

5000

***************** PROCESS Procedure for SPSS Version 3.5 *****************

Written by Andrew F. Hayes, Ph.D. www.afhayes.com

Documentation available in Hayes (2018). www.guilford.com/p/hayes3

**************************************************************************

**Model : 4**

**Y : FACT_B**

**X : Stigma_I**

**M1 : Comprehesibility**

**M2 : Manageabability**

**M3 : Meaningfulness**

**M4 : MOS_SSS = total social support**

**M5 : CopPosit = positive reframing**

Covariates:

Age_cov Educatio Employme Social_p Insuranc Major_Ps Chemo_co Mastecto Radiothe

Sample

Size: 219

**************************************************************************

OUTCOME VARIABLE:

Comprehe

Model Summary

R R-sq MSE F df1 df2 p

.37350 .13950 36.80129 3.37213 10.00000 208.00000 .00043

Model

coeff se t p LLCI ULCI

constant 28.40618 3.60268 7.88474 .00000 21.30374 35.50862

Stigma_I -.31516 .08812 -3.57670 .00043 -.48888 -.14145

Age_cov .04333 .04684 .92508 .35600 -.04901 .13567

Educatio -.32797 1.24258 -.26394 .79209 -2.77764 2.12171

Employme 1.81374 1.31594 1.37829 .16959 -.78054 4.40803

Social_p .37360 .29020 1.28742 .19938 -.19850 .94571

Insuranc -.56031 1.46074 -.38358 .70168 -3.44006 2.31945

Major_Ps -1.83801 .99004 -1.85650 .06480 -3.78982 .11379

Chemo_co -1.13661 .62033 -1.83226 .06834 -2.35956 .08634

Mastecto -1.49233 .58505 -2.55075 .01147 -2.64572 -.33893

Radiothe -1.04589 1.06016 -.98654 .32501 -3.13593 1.04415

Standardized coefficients

coeff

Stigma_I -.24233

Age_cov .06161

Educatio -.02026

Employme .10664

Social_p .08449

Insuranc -.02533

Major_Ps -.12272

Chemo_co -.15126

Mastecto -.18764

Radiothe -.08030

| **Benjamini-Hochberg Procedure** | | | | |
| --- | --- | --- | --- | --- |
|  |  | **Ascending P-value s** | **I = ranking** | **(I/10)* 0.10** |
|  | **Stigma** | **.0004** | **1** | **.01** |
|  | **Mastectomy** | **.011** | **2** | **.02** |
|  | **Major Psychological issues** | **~~.064~~** | **3** | **.03** |
|  | **Chemotherapy** | **~~.068~~** | **4** | **.04** |
|  | **Employment** | **~~.169~~** | **5** | **.05** |
|  | **Social participation frequency** | **~~.199~~** | **6** | **.06** |
|  | **Radiotherapy** | **~~.325~~** | **7** | **.07** |
|  | **Age** | **~~.356~~** | **8** | **.08** |
|  | **Insurance** | **~~.701~~** | **9** | **.09** |
|  | **Education** | **~~.792~~** | **10** | **.10** |

**************************************************************************

OUTCOME VARIABLE:

Manageab

Model Summary

R R-sq MSE F df1 df2 p

.35135 .12344 30.49049 2.92923 10.00000 208.00000 .00186

Model

coeff se t p LLCI ULCI

constant 17.30925 3.27926 5.27840 .00000 10.84440 23.77411

Stigma_I -.26142 .08021 -3.25943 .00130 -.41954 -.10330

Age_cov .06617 .04263 1.55215 .12215 -.01788 .15022

Educatio 1.51749 1.13104 1.34168 .18116 -.71228 3.74725

Employme .88209 1.19780 .73642 .46231 -1.47931 3.24348

Social_p .50006 .26415 1.89312 .05973 -.02069 1.02080

Insuranc -1.12291 1.32961 -.84454 .39934 -3.74415 1.49832

Major_Ps -1.65245 .90117 -1.83368 .06813 -3.42904 .12414

Chemo_co -.05289 .56464 -.09367 .92546 -1.16605 1.06027

Mastecto -.70809 .53253 -1.32966 .18509 -1.75794 .34177

Radiothe .09824 .96499 .10181 .91901 -1.80417 2.00066

Standardized coefficients

coeff

Stigma_I -.22288

Age_cov .10433

Educatio .10394

Employme .05750

Social_p .12540

Insuranc -.05628

Major_Ps -.12233

Chemo_co -.00780

Mastecto -.09872

Radiothe .00836

| **Benjamini-Hochberg Procedure** | | | | |
| --- | --- | --- | --- | --- |
|  |  | **Ascending P-value s** | **I = ranking** | **(I/10)* 0.10** |
|  | **Stigma** | **.001** | **1** | **.01** |
|  | **Social participation frequency** | **~~.059~~** | **2** | **.02** |
|  | **Major Psychological issues** | **~~.068~~** | **3** | **.03** |
|  | **Age** | **~~.122~~** | **4** | **.04** |
|  | **Education** | **~~.181~~** | **5** | **.05** |
|  | **Mastectomy** | **~~.185~~** | **6** | **.06** |
|  | **Insurance** | **~~.399~~** | **7** | **.07** |
|  | **Employment** | **~~.736~~** | **8** | **.08** |
|  | **Radiotherapy** | **~~.919~~** | **9** | **.09** |
|  | **Chemotherapy** | **~~.925~~** | **10** | **.10** |

**************************************************************************

OUTCOME VARIABLE:

Meaningf

Model Summary

R R-sq MSE F df1 df2 p

.43827 .19208 20.04765 4.94515 10.00000 208.00000 .00000

Model

coeff se t p LLCI ULCI

constant 21.99116 2.65904 8.27033 .00000 16.74902 27.23329

Stigma_I -.24362 .06504 -3.74594 .00023 -.37184 -.11541

Age_cov .06993 .03457 2.02298 .04436 .00178 .13809

Educatio -1.54766 .91712 -1.68752 .09300 -3.35570 .26039

Employme .61881 .97126 .63712 .52475 -1.29597 2.53358

Social_p .50570 .21419 2.36102 .01915 .08344 .92795

Insuranc .00323 1.07813 .00300 .99761 -2.12224 2.12870

Major_Ps -1.36998 .73073 -1.87482 .06222 -2.81056 .07060

Chemo_co -.31410 .45785 -.68604 .49345 -1.21673 .58852

Mastecto -.82346 .43181 -1.90699 .05790 -1.67475 .02783

Radiothe .54173 .78248 .69232 .48951 -1.00088 2.08433

Standardized coefficients

coeff

Stigma_I -.24592

Age_cov .13054

Educatio -.12551

Employme .04776

Social_p .15015

Insuranc .00019

Major_Ps -.12008

Chemo_co -.05488

Mastecto -.13593

Radiothe .05460

| **Benjamini-Hochberg Procedure** | | | | |
| --- | --- | --- | --- | --- |
|  |  | **Ascending P-value s** | **I = ranking** | **(I/10)* 0.10** |
|  | **Stigma** | **.0002** | **1** | **.01** |
|  | **Social participation frequency** | **.019** | **2** | **.02** |
|  | **Age** | **~~.044~~** | **3** | **.03** |
|  | **Mastectomy** | **~~.057~~** | **4** | **.04** |
|  | **Major Psychological issues** | **~~.062~~** | **5** | **.05** |
|  | **Education** | **~~.093~~** | **6** | **.06** |
|  | **Radiotherapy** | **~~.489~~** | **7** | **.07** |
|  | **Employment** | **~~.524~~** | **8** | **.08** |
|  | **Chemotherapy** | **~~.493~~** | **9** | **.09** |
|  | **Insurance** | **~~.997~~** | **10** | **.10** |

**************************************************************************

OUTCOME VARIABLE:

MOS_SSS

Model Summary

R R-sq MSE F df1 df2 p

.34356 .11803 .06736 2.78361 10.00000 208.00000 .00299

Model

coeff se t p LLCI ULCI

constant .45133 .15413 2.92819 .00379 .14747 .75519

Stigma_I -.00955 .00377 -2.53373 .01202 -.01698 -.00212

Age_cov .00133 .00200 .66567 .50636 -.00262 .00528

Educatio -.03675 .05316 -.69133 .49013 -.14156 .06805

Employme -.02766 .05630 -.49132 .62372 -.13865 .08333

Social_p -.00085 .01242 -.06857 .94540 -.02533 .02362

Insuranc .07432 .06249 1.18918 .23572 -.04889 .19752

Major_Ps -.10845 .04236 -2.56037 .01117 -.19195 -.02495

Chemo_co .00229 .02654 .08621 .93138 -.05003 .05461

Mastecto -.02110 .02503 -.84289 .40026 -.07044 .02825

Radiothe .06329 .04536 1.39536 .16440 -.02613 .15271

Standardized coefficients

coeff

Stigma_I -.17379

Age_cov .04488

Educatio -.05372

Employme -.03848

Social_p -.00456

Insuranc .07949

Major_Ps -.17134

Chemo_co .00721

Mastecto -.06277

Radiothe .11499

| **Benjamini-Hochberg Procedure** | | | | |
| --- | --- | --- | --- | --- |
|  |  | **Ascending P-value s** | **I = ranking** | **(I/10)* 0.10** |
|  | **Stigma** | **.012** | **1** | **.01** |
|  | **Major Psychological issues** | **.011** | **2** | **.02** |
|  | **Radiotherapy** | **~~.164~~** | **3** | **.03** |
|  | **Insurance** | **~~.235~~** | **4** | **.04** |
|  | **Mastectomy** | **~~.400~~** | **5** | **.05** |
|  | **Education** | **~~.490~~** | **6** | **.06** |
|  | **Age** | **~~.506~~** | **7** | **.07** |
|  | **Employment** | **~~.623~~** | **8** | **.08** |
|  | **Chemotherapy** | **~~.931~~** | **9** | **.09** |
|  | **Social participation frequency** | **~~.945~~** | **10** | **.10** |

**************************************************************************

OUTCOME VARIABLE:

CopPosit

Model Summary

R R-sq MSE F df1 df2 p

.33083 .10945 .80153 2.55639 10.00000 208.00000 .00623

Model

coeff se t p LLCI ULCI

constant 3.43955 .53168 6.46918 .00000 2.39137 4.48773

Stigma_I -.04736 .01300 -3.64170 .00034 -.07299 -.02172

Age_cov -.00942 .00691 -1.36268 .17446 -.02305 .00421

Educatio -.04722 .18338 -.25752 .79703 -.40875 .31430

Employme .10310 .19421 .53086 .59608 -.27977 .48596

Social_p -.02313 .04283 -.54008 .58972 -.10756 .06130

Insuranc .01560 .21558 .07238 .94237 -.40939 .44060

Major_Ps -.06339 .14611 -.43382 .66487 -.35143 .22466

Chemo_co -.03581 .09155 -.39120 .69605 -.21630 .14467

Mastecto -.01352 .08634 -.15663 .87569 -.18374 .15669

Radiothe .25174 .15646 1.60896 .10914 -.05671 .56018

Standardized coefficients

coeff

Stigma_I -.25100

Age_cov -.09232

Educatio -.02011

Employme .04178

Social_p -.03606

Insuranc .00486

Major_Ps -.02917

Chemo_co -.03285

Mastecto -.01172

Radiothe .13323

| **Benjamini-Hochberg Procedure** | | | | |
| --- | --- | --- | --- | --- |
|  |  | **Ascending P-value s** | **I = ranking** | **(I/10)* 0.10** |
|  | **Stigma** | **.0003** | **1** | **.01** |
|  | **Radiotherapy** | **~~.109~~** | **2** | **.02** |
|  | **Age** | **~~.174~~** | **3** | **.03** |
|  | **Social participation frequency** | **~~.589~~** | **4** | **.04** |
|  | **Employment** | **~~.596~~** | **5** | **.05** |
|  | **Major Psychological issues** | **~~.664~~** | **6** | **.06** |
|  | **Chemotherapy** | **~~.696~~** | **7** | **.07** |
|  | **Education** | **~~.797~~** | **8** | **.08** |
|  | **Mastectomy** | **~~.875~~** | **9** | **.09** |
|  | **Insurance** | **~~.942~~** | **10** | **.10** |

**************************************************************************

OUTCOME VARIABLE:

FACT_B

Model Summary

R R-sq MSE F df1 df2 p

.65417 .42794 246.22271 10.12398 15.00000 203.00000 .00000

Model
[truncated: 5,617 more chars]
