# Supplementary material for: Stigma and Quality of Life in Women With Breast Cancer: Mediation and Moderation Model of Social Support, Sense of Coherence, and Coping Strategies
Source: Front Psychol. 2022 Feb 14;13:657992. doi: 10.3389/fpsyg.2022.657992 (PMC8882621; doi:10.3389/fpsyg.2022.657992)

**Moderation Figures**

**Figure S1**


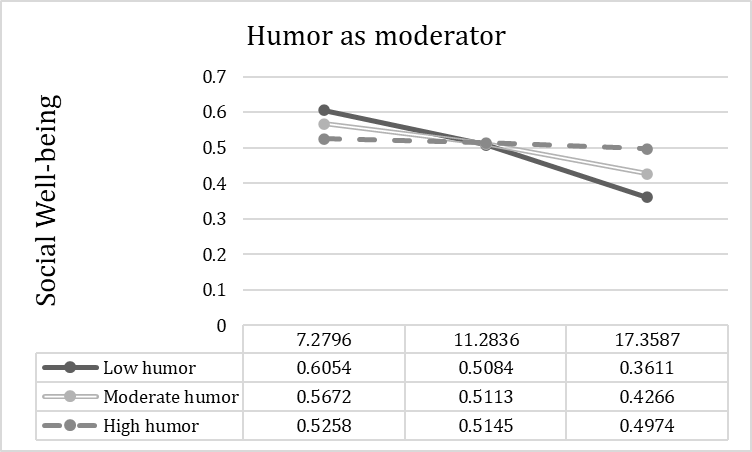


**Figure S2**

**Figure S3**


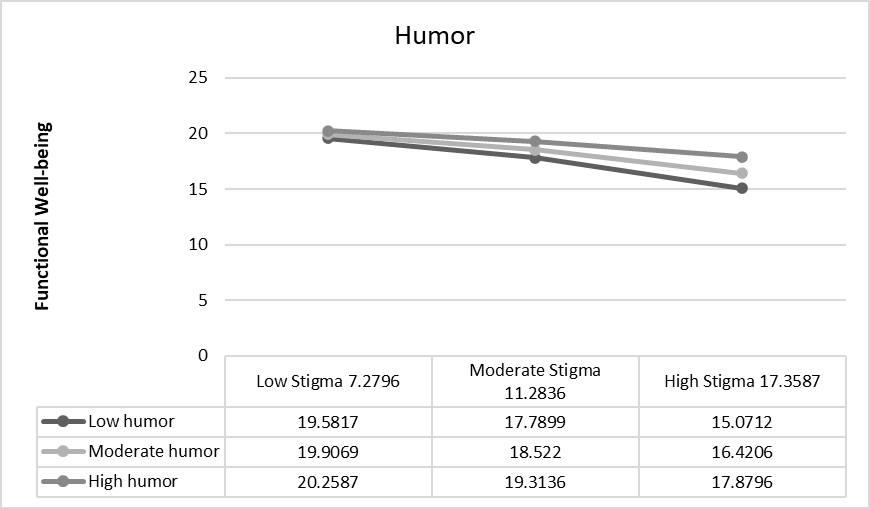


**Figure S4**


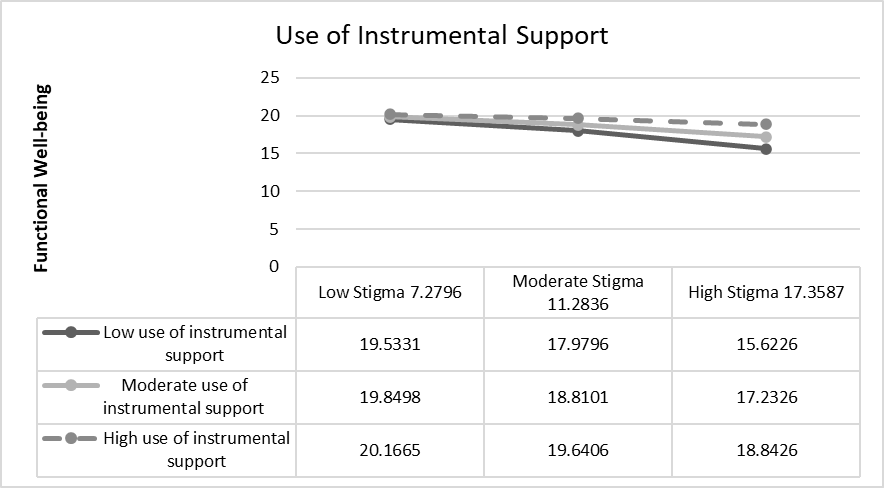


**Figure S5**


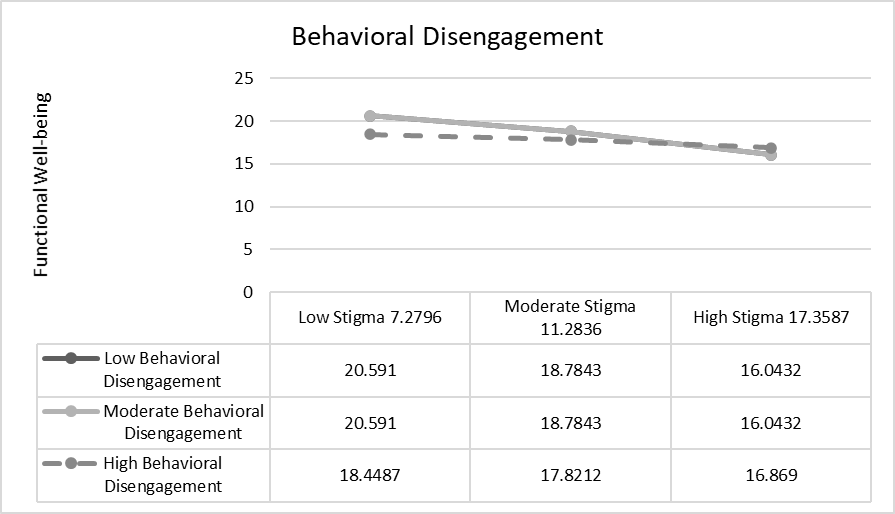


**Figure S6**


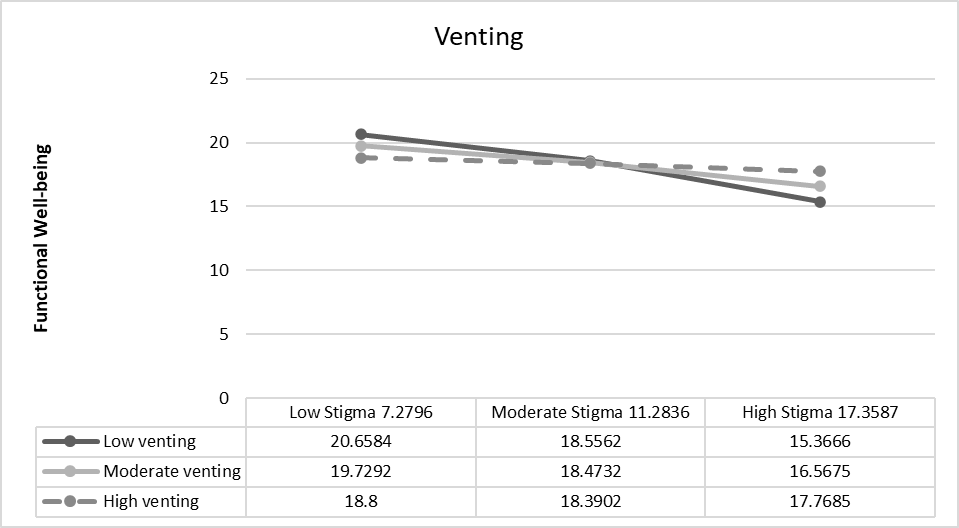


**Figure S7**


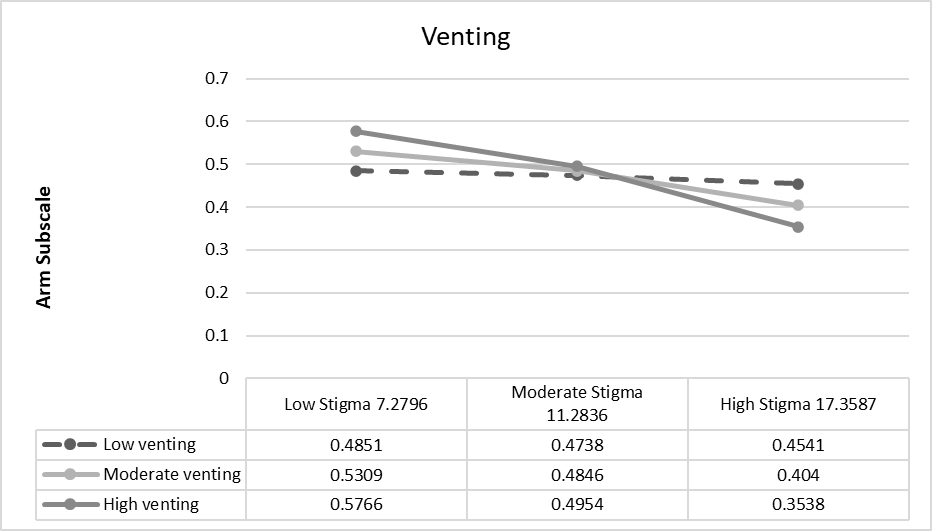


**Figure S8**


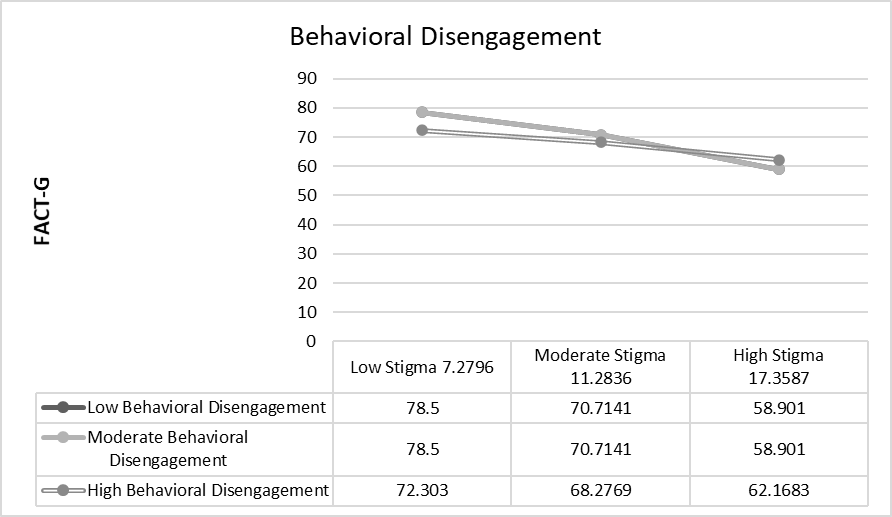


**Figure S9**


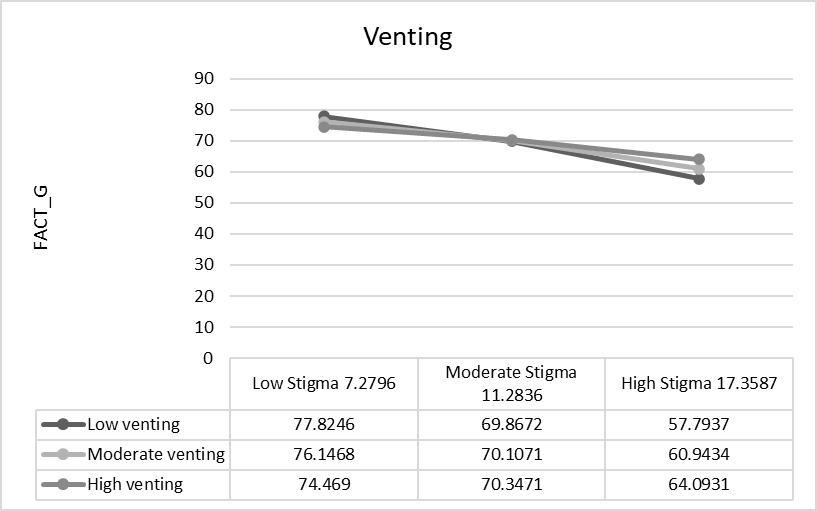


**Figure S10**


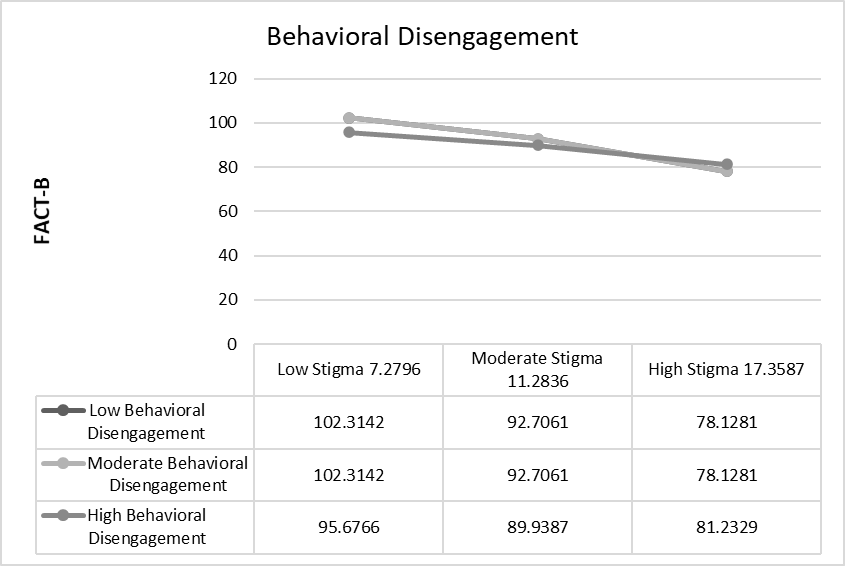


**Figure S10**


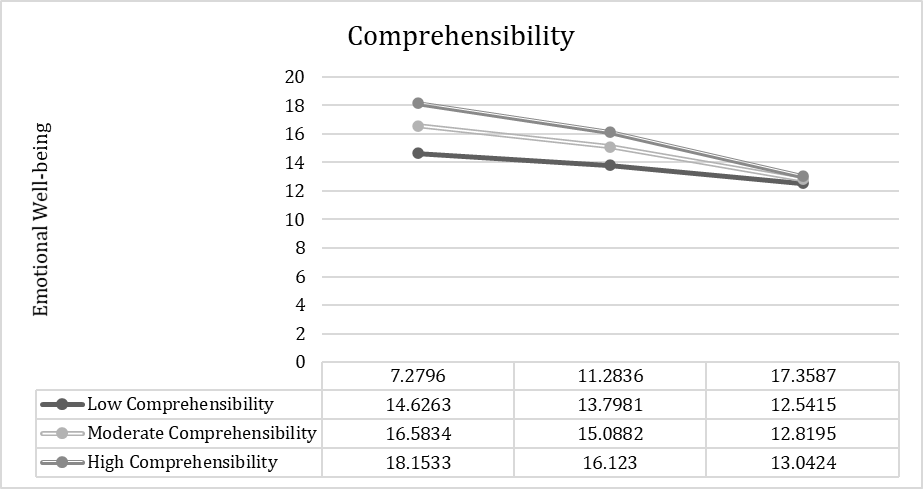


**Figure S12**


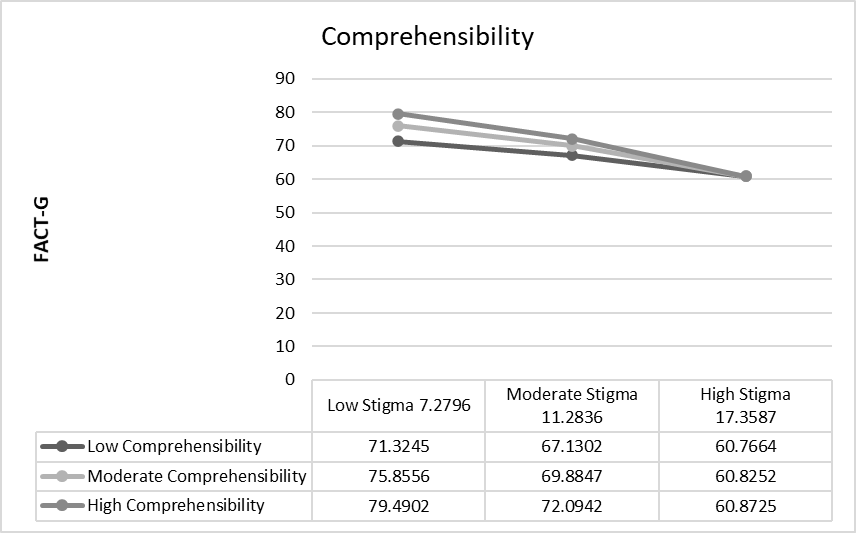


**Figure S13**


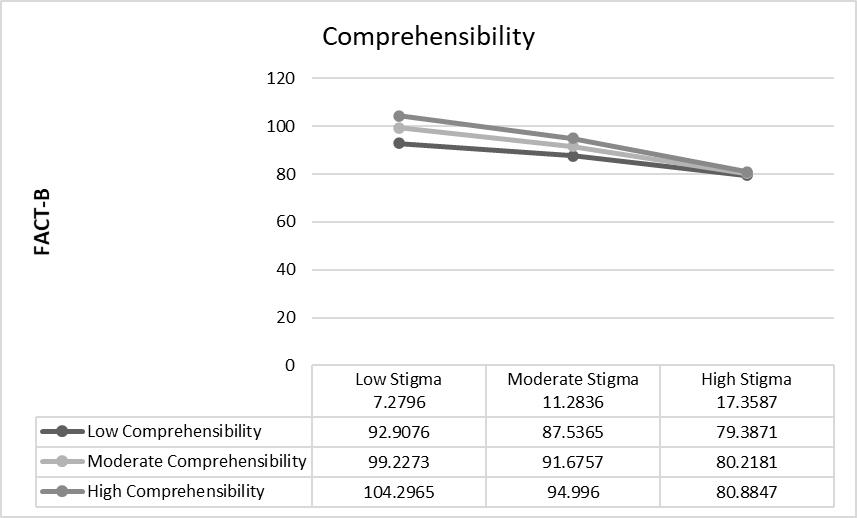

Supplement: Supplementary file 2 [file Data_Sheet_2.docx]
