# Supplementary material for: Stigma and Quality of Life in Women With Breast Cancer: Mediation and Moderation Model of Social Support, Sense of Coherence, and Coping Strategies
Source: Front Psychol. 2022 Feb 14;13:657992. doi: 10.3389/fpsyg.2022.657992 (PMC8882621; doi:10.3389/fpsyg.2022.657992)
Supplement: Supplementary file 3 [file Data_Sheet_3.docx]

**Moderation Analyses**

***Note*. Only the results of interaction effects with P < 0.10 are reported. If the interaction effect was Significant (P < 0.10), the analysis was repeated with mean centering to evaluate the P-values using Benjamini-Hochberg Procedure with false discovery rate (FDR) of 0.2. Approved results are highlighted in Green and non-approved results are in Yellow.**

**************************************************************************

Model : 1

Y : PWB

X : Stigma_I

W : Comprehe

Covariates:

Age_cov Educatio Employme Social_p Insuranc Major_Ps Chemo_co Mastecto Radiothe

Sample

Size: 221

**************************************************************************

OUTCOME VARIABLE:

PWB

Model Summary

R R-sq MSE F df1 df2 p

.4469 .1997 37.3631 4.3256 12.0000 208.0000 .0000

Model

coeff se t p LLCI ULCI

constant 23.8357 3.5425 6.7285 .0000 16.8520 30.8195

Stigma_I -.5335 .0917 -5.8166 .0000 -.7143 -.3527

Comprehe -.0211 .0699 -.3024 .7627 -.1590 .1167

Int_1 -.0224 .0135 -1.6576 .0989 -.0490 .0042

Age_cov -.0651 .0469 -1.3882 .1666 -.1576 .0274

Educatio -2.9764 1.2518 -2.3778 .0183 -5.4442 -.5087

Employme -2.7077 1.3320 -2.0328 .0433 -5.3337 -.0817

Social_p .3386 .2932 1.1549 .2495 -.2394 .9165

Insuranc .6922 1.4724 .4701 .6388 -2.2105 3.5950

Major_Ps -.0609 .9997 -.0609 .9515 -2.0318 1.9100

Chemo_co -.0766 .6305 -.1214 .9035 -1.3195 1.1664

Mastecto .1605 .5969 .2690 .7882 -1.0161 1.3372

Radiothe -1.2654 1.0728 -1.1795 .2396 -3.3804 .8497

| **Benjamini-Hochberg Procedure** | | | | |
| --- | --- | --- | --- | --- |
|  |  | **Ascending P-value s** | **I = ranking** | **(I/12)* 0.20** |
|  | **Stigma** | **.0000** | **1** | **.0166** |
|  | **Education** | **.0183** | **2** | **.033** |
|  | **Employment** | **.0433** | **3** | **.050** |
|  | **Interaction Effect** | **~~.0989~~** | **4** | **.066** |
|  | **Comprehensibility** | **~~.7627~~** | **5** | **.0833** |
|  | **Age** | **~~.1666~~** | **6** | **.10** |
|  | **Radiotherapy** | **~~.2396~~** | **7** | **.116** |
|  | **Social participation frequency** | **~~.2495~~** | **8** | **.133** |
|  | **Insurance** | **~~.6388~~** | **9** | **.15** |
|  | **Mastectomy** | **~~.7882~~** | **10** | **.166** |
|  | **Chemotherapy** | **~~.9035~~** | **11** | **.183** |
|  | **Major psychological problems** | **~~.9515~~** | **12** | **.20** |

Product terms key:

Int_1 : Stigma_I x Comprehe

Test(s) of highest order unconditional interaction(s):

R2-chng F df1 df2 p

X*W .0106 2.7477 1.0000 208.0000 .0989

----------

Focal predict: Stigma_I (X)

Mod var: Comprehe (W)

Conditional effects of the focal predictor at values of the moderator(s):

Comprehe Effect se t p LLCI ULCI

13.5200 -.3788 .1209 -3.1324 .0020 -.6173 -.1404

21.0000 -.5462 .0931 -5.8639 .0000 -.7298 -.3625

27.0000 -.6804 .1365 -4.9851 .0000 -.9494 -.4113

Moderator value(s) defining Johnson-Neyman significance region(s):

Value % below % above

10.1896 6.7873 93.2127

Conditional effect of focal predictor at values of the moderator:

Comprehe Effect se t p LLCI ULCI

5.0000 -.1883 .2150 -.8757 .3822 -.6122 .2356

6.5000 -.2218 .1968 -1.1269 .2611 -.6099 .1662

8.0000 -.2554 .1791 -1.4257 .1555 -.6085 .0978

9.5000 -.2889 .1620 -1.7835 .0760 -.6083 .0304

10.1896 -.3044 .1544 -1.9714 .0500 -.6087 .0000

11.0000 -.3225 .1457 -2.2137 .0279 -.6097 -.0353

12.5000 -.3560 .1305 -2.7292 .0069 -.6132 -.0988

14.0000 -.3896 .1168 -3.3365 .0010 -.6198 -.1594

15.5000 -.4231 .1052 -4.0218 .0001 -.6305 -.2157

17.0000 -.4567 .0966 -4.7296 .0000 -.6470 -.2663

18.5000 -.4902 .0916 -5.3497 .0000 -.6709 -.3096

20.0000 -.5238 .0911 -5.7525 .0000 -.7033 -.3443

21.5000 -.5573 .0949 -5.8738 .0000 -.7444 -.3703

23.0000 -.5909 .1026 -5.7568 .0000 -.7932 -.3885

24.5000 -.6244 .1135 -5.5008 .0000 -.8482 -.4006

26.0000 -.6580 .1267 -5.1926 .0000 -.9078 -.4082

27.5000 -.6915 .1416 -4.8842 .0000 -.9707 -.4124

29.0000 -.7251 .1577 -4.5991 .0000 -1.0359 -.4143

30.5000 -.7586 .1746 -4.3450 .0000 -1.1029 -.4144

32.0000 -.7922 .1922 -4.1221 .0001 -1.1711 -.4133

33.5000 -.8257 .2102 -3.9276 .0001 -1.2402 -.4113

35.0000 -.8593 .2287 -3.7579 .0002 -1.3101 -.4085

Data for visualizing the conditional effect of the focal predictor:

Paste text below into a SPSS syntax window and execute to produce plot.

DATA LIST FREE/

Stigma_I Comprehe PWB .

BEGIN DATA.

7.2796 13.5200 18.8792

11.2836 13.5200 17.3623

17.3587 13.5200 15.0608

7.2796 21.0000 19.5176

11.2836 21.0000 17.3308

17.3587 21.0000 14.0129

7.2796 27.0000 20.0297

11.2836 27.0000 17.3055

17.3587 27.0000 13.1723

END DATA.

GRAPH/SCATTERPLOT=

Stigma_I WITH PWB BY Comprehe .

*********************** ANALYSIS NOTES AND ERRORS ************************

Level of confidence for all confidence intervals in output:

95.0000

W values in conditional tables are the 16th, 50th, and 84th percentiles.

**************************************************************************

Model : 1

Y : SWBwn

X : Stigma_I

W : copHumor

Covariates:

Age_cov Educatio Employme Social_p Insuranc Major_Ps Chemo_co Mastecto Radiothe

Sample

Size: 220

**************************************************************************

OUTCOME VARIABLE:

SWBwn

Model Summary

R R-sq MSE F df1 df2 p

.4607 .2123 .0669 4.6479 12.0000 207.0000 .0000

Model

coeff se t p LLCI ULCI

constant .3564 .1502 2.3723 .0186 .0602 .6526

Stigma_I -.0133 .0038 -3.5238 .0005 -.0207 -.0058

copHumor .0505 .0772 .6540 .5138 -.1018 .2028

Int_1 .0499 .0167 2.9900 .0031 .0170 .0828

Age_cov .0003 .0020 .1375 .8908 -.0037 .0042

Educatio -.0159 .0530 -.3004 .7642 -.1204 .0886

Employme .0144 .0562 .2571 .7973 -.0963 .1252

Social_p .0087 .0124 .7045 .4819 -.0157 .0332

Insuranc .1080 .0629 1.7160 .0876 -.0161 .2320

Major_Ps -.1441 .0426 -3.3796 .0009 -.2282 -.0600

Chemo_co -.0472 .0267 -1.7677 .0786 -.0998 .0054

Mastecto -.0039 .0251 -.1547 .8772 -.0533 .0455

Radiothe .0948 .0452 2.0988 .0370 .0058 .1839

| **Benjamini-Hochberg Procedure** | | | | |
| --- | --- | --- | --- | --- |
|  |  | **Ascending P-value s** | **I = ranking** | **(I/12)* 0.20** |
|  | **Stigma** | **.0005** | **1** | **.0166** |
|  | **Major psychological problems** | **.0009** | **2** | **.033** |
|  | **Interaction Effect** | **.0031** | **3** | **.050** |
|  | **Radiotherapy** | **.0370** | **4** | **.066** |
|  | **Chemotherapy** | **.0786** | **5** | **.0833** |
|  | **Insurance** | **.0876** | **6** | **.10** |
|  | **Social participation frequency** | **~~.4819~~** | **7** | **.116** |
|  | **Humor** | **~~.5138~~** | **8** | **.133** |
|  | **Education** | **~~.7642~~** | **9** | **.15** |
|  | **Employment** | **~~.7973~~** | **10** | **.166** |
|  | **Mastectomy** | **~~.8772~~** | **11** | **.183** |
|  | **Age** | **~~.8908~~** | **12** | **.20** |

Product terms key:

Int_1 : Stigma_I x copHumor

Test(s) of highest order unconditional interaction(s):

R2-chng F df1 df2 p

X*W .0340 8.9398 1.0000 207.0000 .0031

----------

Focal predict: Stigma_I (X)

Mod var: copHumor (W)

Conditional effects of the focal predictor at values of the moderator(s):

copHumor Effect se t p LLCI ULCI

.2960 -.0242 .0051 -4.7634 .0000 -.0343 -.0142

.5023 -.0139 .0038 -3.7121 .0003 -.0214 -.0065

.7253 -.0028 .0053 -.5314 .5957 -.0133 .0076

Moderator value(s) defining Johnson-Neyman significance region(s):

Value % below % above

.6154 73.1818 26.8182

Conditional effect of focal predictor at values of the moderator:

copHumor Effect se t p LLCI ULCI

-.1890 -.0485 .0121 -3.9955 .0001 -.0724 -.0245

-.1267 -.0453 .0111 -4.0693 .0001 -.0673 -.0234

-.0644 -.0422 .0102 -4.1529 .0000 -.0623 -.0222

-.0021 -.0391 .0092 -4.2474 .0000 -.0573 -.0210

.0602 -.0360 .0083 -4.3535 .0000 -.0523 -.0197

.1225 -.0329 .0074 -4.4701 .0000 -.0474 -.0184

.1848 -.0298 .0065 -4.5919 .0000 -.0426 -.0170

.2471 -.0267 .0057 -4.7042 .0000 -.0379 -.0155

.3094 -.0236 .0049 -4.7708 .0000 -.0333 -.0138

.3717 -.0205 .0043 -4.7171 .0000 -.0290 -.0119

.4339 -.0174 .0039 -4.4241 .0000 -.0251 -.0096

.4962 -.0142 .0038 -3.7913 .0002 -.0217 -.0068

.5585 -.0111 .0039 -2.8745 .0045 -.0188 -.0035

.6154 -.0083 .0042 -1.9715 .0500 -.0166 .0000

.6208 -.0080 .0043 -1.8889 .0603 -.0164 .0004

.6831 -.0049 .0048 -1.0196 .3091 -.0144 .0046

.7454 -.0018 .0055 -.3271 .7439 -.0127 .0091

.8077 .0013 .0063 .2047 .8380 -.0112 .0138

.8700 .0044 .0072 .6116 .5415 -.0098 .0186

.9323 .0075 .0081 .9266 .3552 -.0085 .0235

.9946 .0106 .0090 1.1746 .2415 -.0072 .0285

1.0569 .0137 .0100 1.3733 .1711 -.0060 .0335

Data for visualizing the conditional effect of the focal predictor:

Paste text below into a SPSS syntax window and execute to produce plot.

DATA LIST FREE/

Stigma_I copHumor SWBwn .

BEGIN DATA.

7.2796 .2960 .6054

11.2836 .2960 .5084

17.3587 .2960 .3611

7.2796 .5023 .5672

11.2836 .5023 .5113

17.3587 .5023 .4266

7.2796 .7253 .5258

11.2836 .7253 .5145

17.3587 .7253 .4974

END DATA.

GRAPH/SCATTERPLOT=

Stigma_I WITH SWBwn BY copHumor .

*********************** ANALYSIS NOTES AND ERRORS ************************

Level of confidence for all confidence intervals in output:

95.0000

**************************************************************************

Model : 1

Y : SWBwn

X : Stigma_I

W : copBehav

Covariates:

Age_cov Educatio Employme Social_p Insuranc Major_Ps Chemo_co Mastecto Radiothe

Sample

Size: 219

**************************************************************************

OUTCOME VARIABLE:

SWBwn

Model Summary

R R-sq MSE F df1 df2 p

.4361 .1902 .0680 4.0310 12.0000 206.0000 .0000

Model

coeff se t p LLCI ULCI

constant .3839 .1520 2.5248 .0123 .0841 .6836

Stigma_I -.0140 .0040 -3.5355 .0005 -.0218 -.0062

copBehav -.0765 .1472 -.5197 .6038 -.3667 .2137

Int_1 .0479 .0275 1.7387 .0836 -.0064 .1022

Age_cov -.0009 .0020 -.4496 .6535 -.0049 .0031

Educatio -.0170 .0535 -.3174 .7513 -.1224 .0884

Employme .0074 .0571 .1286 .8978 -.1053 .1200

Social_p .0100 .0126 .7957 .4271 -.0148 .0347

Insuranc .0878 .0628 1.3983 .1635 -.0360 .2117

Major_Ps -.1562 .0426 -3.6673 .0003 -.2402 -.0722

Chemo_co -.0267 .0270 -.9884 .3241 -.0800 .0266

Mastecto .0019 .0255 .0744 .9408 -.0484 .0522

Radiothe .0939 .0457 2.0559 .0411 .0039 .1840

| **Benjamini-Hochberg Procedure** | | | | |
| --- | --- | --- | --- | --- |
|  |  | **Ascending P-value s** | **I = ranking** | **(I/12)* 0.20** |
|  | **Stigma** | **.0005** | **1** | **.0166** |
|  | **Major psychological problems** | **.0003** | **2** | **.033** |
|  | **Radiotherapy** | **.04111** | **3** | **.050** |
|  | **Interaction Effect** | **~~.0836~~** | **4** | **.066** |
|  | **Insurance** | **~~.1635~~** | **5** | **.0833** |
|  | **Chemotherapy** | **~~.3241~~** | **6** | **.10** |
|  | **Social participation frequency** | **~~.4271~~** | **7** | **.116** |
|  | **Behavioral Disengagement** | **~~.6038~~** | **8** | **.133** |
|  | **Age** | **~~.6535~~** | **9** | **.15** |
|  | **Education** | **~~.7513~~** | **10** | **.166** |
|  | **Employment** | **~~.8978~~** | **11** | **.183** |
|  | **Mastectomy** | **~~.9408~~** | **12** | **.20** |

Product terms key:

Int_1 : Stigma_I x copBehav

Test(s) of highest order unconditional interaction(s):

R2-chng F df1 df2 p

X*W .0119 3.0231 1.0000 206.0000 .0836

----------

Focal predict: Stigma_I (X)

Mod var: copBehav (W)

Conditional effects of the focal predictor at values of the moderator(s):

copBehav Effect se t p LLCI ULCI

.4061 -.0191 .0053 -3.5925 .0004 -.0296 -.0086

.4061 -.0191 .0053 -3.5925 .0004 -.0296 -.0086

.6802 -.0060 .0055 -1.0810 .2809 -.0169 .0049

Moderator value(s) defining Johnson-Neyman significance region(s):

Value % below % above

.6196 80.3653 19.6347

Conditional effect of focal predictor at values of the moderator:

copBehav Effect se t p LLCI ULCI

.4061 -.0191 .0053 -3.5925 .0004 -.0296 -.0086

.4293 -.0180 .0049 -3.6682 .0003 -.0277 -.0083

.4526 -.0169 .0045 -3.7140 .0003 -.0259 -.0079

.4759 -.0158 .0043 -3.7086 .0003 -.0242 -.0074

.4992 -.0147 .0040 -3.6279 .0004 -.0226 -.0067

.5225 -.0135 .0039 -3.4530 .0007 -.0213 -.0058

.5458 -.0124 .0039 -3.1809 .0017 -.0201 -.0047

.5691 -.0113 .0040 -2.8308 .0051 -.0192 -.0034

.5923 -.0102 .0042 -2.4377 .0156 -.0184 -.0019

.6156 -.0091 .0045 -2.0384 .0428 -.0179 -.0003

.6196 -.0089 .0045 -1.9715 .0500 -.0178 .0000

.6389 -.0080 .0048 -1.6602 .0984 -.0174 .0015

.6622 -.0068 .0052 -1.3179 .1890 -.0171 .0034

.6855 -.0057 .0056 -1.0163 .3107 -.0169 .0054

.7088 -.0046 .0061 -.7545 .4514 -.0167 .0075

.7320 -.0035 .0066 -.5285 .5977 -.0166 .0096

.7553 -.0024 .0072 -.3336 .7390 -.0165 .0117

.7786 -.0013 .0077 -.1652 .8690 -.0165 .0139

.8019 -.0002 .0083 -.0190 .9849 -.0164 .0161

.8252 .0010 .0088 .1085 .9137 -.0165 .0184

.8485 .0021 .0094 .2203 .8259 -.0165 .0206

.8718 .0032 .0100 .3189 .7501 -.0165 .0229

Data for visualizing the conditional effect of the focal predictor:

Paste text below into a SPSS syntax window and execute to produce plot.

DATA LIST FREE/

Stigma_I copBehav SWBwn .

BEGIN DATA.

7.2796 .4061 .5932

11.2836 .4061 .5166

17.3587 .4061 .4005

7.2796 .4061 .5932

11.2836 .4061 .5166

17.3587 .4061 .4005

7.2796 .6802 .5098

11.2836 .6802 .4859

17.3587 .6802 .4495

END DATA.

GRAPH/SCATTERPLOT=

Stigma_I WITH SWBwn BY copBehav .

*********************** ANALYSIS NOTES AND ERRORS ************************

Level of confidence for all confidence intervals in output:

95.0000

**************************************************************************

Model : 1

Y : EWB

X : Stigma_I

W : Comprehe

Covariates:

Age_cov Educatio Employme Social_p Insuranc Major_Ps Chemo_co Mastecto Radiothe

Sample

Size: 221

**************************************************************************

OUTCOME VARIABLE:

EWB

Model Summary

R R-sq MSE F df1 df2 p

.5007 .2507 21.4020 5.7993 12.0000 208.0000 .0000

Model

coeff se t p LLCI ULCI

constant 15.7696 2.6811 5.8818 .0000 10.4840 21.0552

Stigma_I -.3608 .0694 -5.1981 .0000 -.4977 -.2240

Comprehe .1556 .0529 2.9410 .0036 .0513 .2599

Int_1 -.0223 .0102 -2.1809 .0303 -.0424 -.0021

Age_cov .0105 .0355 .2959 .7676 -.0595 .0805

Educatio .4187 .9474 .4419 .6590 -1.4490 2.2864

Employme -.1434 1.0081 -.1422 .8870 -2.1308 1.8440

Social_p .3598 .2219 1.6219 .1063 -.0776 .7973

Insuranc .6277 1.1144 .5633 .5738 -1.5692 2.8247

Major_Ps -1.5350 .7566 -2.0287 .0438 -3.0266 -.0433

Chemo_co -.1694 .4772 -.3551 .7229 -1.1102 .7713

Mastecto -.5775 .4517 -1.2784 .2025 -1.4681 .3131

Radiothe -.9700 .8120 -1.1946 .2336 -2.5707 .6308

| **Benjamini-Hochberg Procedure** | | | | |
| --- | --- | --- | --- | --- |
|  |  | **Ascending P-value s** | **I = ranking** | **(I/12)* 0.20** |
|  | **Stigma** | **.0000** | **1** | **.0166** |
|  | **Comprehensibility** | **.0036** | **2** | **.033** |
|  | **Interaction Effect** | **.0303** | **3** | **.050** |
|  | **Major psychological problems** | **.0438** | **4** | **.066** |
|  | **Social participation frequency** | **~~.1063~~** | **5** | **.0833** |
|  | **Mastectomy** | **~~.2025~~** | **6** | **.10** |
|  | **Radiotherapy** | **~~.2336~~** | **7** | **.116** |
|  | **Insurance** | **~~.5738~~** | **8** | **.133** |
|  | **Education** | **~~.6590~~** | **9** | **.15** |
|  | **Chemotherapy** | **~~.7229~~** | **10** | **.166** |
|  | **Age** | **~~.7676~~** | **11** | **.183** |
|  | **Employment** | **~~.8870~~** | **12** | **.20** |

Product terms key:

Int_1 : Stigma_I x Comprehe

Test(s) of highest order unconditional interaction(s):

R2-chng F df1 df2 p

X*W .0171 4.7563 1.0000 208.0000 .0303

----------

Focal predict: Stigma_I (X)

Mod var: Comprehe (W)

Conditional effects of the focal predictor at values of the moderator(s):

Comprehe Effect se t p LLCI ULCI

13.5200 -.2068 .0915 -2.2597 .0249 -.3873 -.0264

21.0000 -.3734 .0705 -5.2978 .0000 -.5124 -.2345

27.0000 -.5071 .1033 -4.9092 .0000 -.7107 -.3034

Moderator value(s) defining Johnson-Neyman significance region(s):

Value % below % above

12.7875 11.3122 88.6878

Conditional effect of focal predictor at values of the moderator:

Comprehe Effect se t p LLCI ULCI

5.0000 -.0171 .1627 -.1050 .9165 -.3379 .3037

6.5000 -.0505 .1490 -.3389 .7350 -.3442 .2432

8.0000 -.0839 .1356 -.6189 .5367 -.3512 .1834

9.5000 -.1173 .1226 -.9568 .3398 -.3590 .1244

11.0000 -.1507 .1103 -1.3670 .1731 -.3681 .0666

12.5000 -.1841 .0987 -1.8649 .0636 -.3788 .0105

12.7875 -.1905 .0966 -1.9714 .0500 -.3811 .0000

14.0000 -.2175 .0884 -2.4616 .0146 -.3918 -.0433

15.5000 -.2509 .0796 -3.1515 .0019 -.4079 -.0940

17.0000 -.2844 .0731 -3.8910 .0001 -.4284 -.1403

18.5000 -.3178 .0694 -4.5817 .0000 -.4545 -.1810

20.0000 -.3512 .0689 -5.0959 .0000 -.4870 -.2153

21.5000 -.3846 .0718 -5.3553 .0000 -.5262 -.2430

23.0000 -.4180 .0777 -5.3807 .0000 -.5711 -.2648

24.5000 -.4514 .0859 -5.2540 .0000 -.6208 -.2820

26.0000 -.4848 .0959 -5.0551 .0000 -.6739 -.2957

27.5000 -.5182 .1072 -4.8359 .0000 -.7295 -.3070

29.0000 -.5516 .1193 -4.6229 .0000 -.7869 -.3164

30.5000 -.5850 .1321 -4.4272 .0000 -.8456 -.3245

32.0000 -.6184 .1455 -4.2519 .0000 -.9052 -.3317

33.5000 -.6519 .1591 -4.0966 .0001 -.9655 -.3382

35.0000 -.6853 .1731 -3.9596 .0001 -1.0264 -.3441

Data for visualizing the conditional effect of the focal predictor:

Paste text below into a SPSS syntax window and execute to produce plot.

DATA LIST FREE/

Stigma_I Comprehe EWB .

BEGIN DATA.

7.2796 13.5200 14.6263

11.2836 13.5200 13.7981

17.3587 13.5200 12.5415

7.2796 21.0000 16.5834

11.2836 21.0000 15.0882

17.3587 21.0000 12.8195

7.2796 27.0000 18.1533

11.2836 27.0000 16.1230

17.3587 27.0000 13.0424

END DATA.

GRAPH/SCATTERPLOT=

Stigma_I WITH EWB BY Comprehe .

*********************** ANALYSIS NOTES AND ERRORS ************************

Level of confidence for all confidence intervals in output:

95.0000

W values in conditional tables are the 16th, 50th, and 84th percentiles.

**************************************************************************

Model : 1

Y : EWB

X : Stigma_I

W : copBehav

Covariates:

Age_cov Educatio Employme Social_p Insuranc Major_Ps Chemo_co Mastecto Radiothe

Sample

Size: 219

**************************************************************************

OUTCOME VARIABLE:

EWB

Model Summary

R R-sq MSE F df1 df2 p

.4662 .2174 22.3162 4.7677 12.0000 206.0000 .0000

Model

coeff se t p LLCI ULCI

constant 15.9858 2.7541 5.8044 .0000 10.5560 21.4156

Stigma_I -.4191 .0717 -5.8469 .0000 -.5605 -.2778

copBehav -.2711 2.6662 -.1017 .9191 -5.5276 4.9854

Int_1 1.0342 .4991 2.0723 .0395 .0503 2.0181

Age_cov .0214 .0367 .5828 .5607 -.0510 .0937

Educatio .3558 .9685 .3674 .7137 -1.5536 2.2652

Employme .2098 1.0352 .2027 .8396 -1.8312 2.2509

Social_p .4726 .2274 2.0783 .0389 .0243 .9209

Insuranc .5576 1.1378 .4901 .6246 -1.6856 2.8008

Major_Ps -1.9174 .7716 -2.4851 .0137 -3.4385 -.3962

Chemo_co -.2871 .4896 -.5863 .5583 -1.2524 .6783

Mastecto -.8210 .4623 -1.7760 .0772 -1.7324 .0904

Radiothe -1.0978 .8276 -1.3266 .1861 -2.7294 .5338

| **Benjamini-Hochberg Procedure** | | | | |
| --- | --- | --- | --- | --- |
|  |  | **Ascending P-value s** | **I = ranking** | **(I/12)* 0.20** |
|  | **Stigma** | **.0000** | **1** | **.0166** |
|  | **Major psychological problems** | **.0137** | **2** | **.033** |
|  | **Social participation frequency** | **.0389** | **3** | **.050** |
|  | **Interaction Effect** | **.0395** | **4** | **.066** |
|  | **Mastectomy** | **.0772** | **5** | **.0833** |
|  | **Radiotherapy** | **~~.1861~~** | **6** | **.10** |
|  | **Chemotherapy** | **~~.5583~~** | **7** | **.116** |
|  | **Age** | **~~.5607~~** | **8** | **.133** |
|  | **Insurance** | **~~.6246~~** | **9** | **.15** |
|  | **Education** | **~~.7137~~** | **10** | **.166** |
|  | **Employment** | **~~.8396~~** | **11** | **.183** |
|  | **Behavioral disengagement** | **~~.9191~~** | **12** | **.20** |

Product terms key:

Int_1 : Stigma_I x copBehav

Test(s) of highest order unconditional interaction(s):

R2-chng F df1 df2 p

X*W .0163 4.2942 1.0000 206.0000 .0395

----------

Focal predict: Stigma_I (X)

Mod var: copBehav (W)

Conditional effects of the focal predictor at values of the moderator(s):

copBehav Effect se t p LLCI ULCI

.4061 -.5298 .0964 -5.4961 .0000 -.7199 -.3398

.4061 -.5298 .0964 -5.4961 .0000 -.7199 -.3398

.6802 -.2463 .1003 -2.4555 .0149 -.4441 -.0486

Moderator value(s) defining Johnson-Neyman significance region(s):

Value % below % above

.7077 93.6073 6.3927

Conditional effect of focal predictor at values of the moderator:

copBehav Effect se t p LLCI ULCI

.4061 -.5298 .0964 -5.4961 .0000 -.7199 -.3398

.4293 -.5058 .0889 -5.6888 .0000 -.6810 -.3305

.4526 -.4817 .0824 -5.8479 .0000 -.6441 -.3193

.4759 -.4576 .0770 -5.9397 .0000 -.6095 -.3057

.4992 -.4335 .0732 -5.9236 .0000 -.5778 -.2892

.5225 -.4094 .0710 -5.7634 .0000 -.5495 -.2694

.5458 -.3854 .0708 -5.4455 .0000 -.5249 -.2458

.5691 -.3613 .0724 -4.9914 .0000 -.5040 -.2186

.5923 -.3372 .0758 -4.4507 .0000 -.4866 -.1878

.6156 -.3131 .0807 -3.8804 .0001 -.4722 -.1540

.6389 -.2890 .0869 -3.3259 .0010 -.4604 -.1177

.6622 -.2650 .0941 -2.8143 .0054 -.4506 -.0793

.6855 -.2409 .1022 -2.3569 .0194 -.4424 -.0394

.7077 -.2178 .1105 -1.9715 .0500 -.4357 .0000

.7088 -.2168 .1109 -1.9550 .0519 -.4354 .0018

.7320 -.1927 .1201 -1.6049 .1100 -.4294 .0440

.7553 -.1686 .1296 -1.3007 .1948 -.4242 .0870

.7786 -.1445 .1395 -1.0359 .3015 -.4197 .1306

.8019 -.1205 .1497 -.8049 .4218 -.4156 .1746

.8252 -.0964 .1600 -.6024 .5476 -.4119 .2191

.8485 -.0723 .1705 -.4241 .6720 -.4085 .2639

.8718 -.0482 .1812 -.2662 .7903 -.4054 .3089

Data for visualizing the conditional effect of the focal predictor:

Paste text below into a SPSS syntax window and execute to produce plot.

DATA LIST FREE/

Stigma_I copBehav EWB .

BEGIN DATA.

7.2796 .4061 17.2395

11.2836 .4061 15.1180

17.3587 .4061 11.8992

7.2796 .4061 17.2395

11.2836 .4061 15.1180

17.3587 .4061 11.8992

7.2796 .6802 15.8184

11.2836 .6802 14.8321

17.3587 .6802 13.3357

END DATA.

GRAPH/SCATTERPLOT=

Stigma_I WITH EWB BY copBehav .

*********************** ANALYSIS NOTES AND ERRORS ************************

Level of confidence for all confidence intervals in output:

95.0000

**************************************************************************

Model : 1

Y : FWB

X : Stigma_I

W : copHumor

Covariates:

Age_cov Educatio Employme Social_p Insuranc Major_Ps Chemo_co Mastecto Radiothe

Sample

Size: 220

**************************************************************************

OUTCOME VARIABLE:

FWB

Model Summary

R R-sq MSE F df1 df2 p

.4819 .2322 21.1118 5.2178 12.0000 207.0000 .0000

Model

coeff se t p LLCI ULCI

constant 20.7898 2.6684 7.7912 .0000 15.5292 26.0505

Stigma_I -.3392 .0669 -5.0724 .0000 -.4710 -.2074

copHumor 3.9066 1.3720 2.8473 .0049 1.2016 6.6115

Int_1 .4926 .2965 1.6613 .0982 -.0920 1.0772

Age_cov -.0222 .0355 -.6246 .5329 -.0922 .0479

Educatio -1.2679 .9414 -1.3469 .1795 -3.1239 .5880

Employme -.2979 .9978 -.2986 .7655 -2.2651 1.6692

Social_p .4598 .2203 2.0869 .0381 .0254 .8942

Insuranc 1.7381 1.1176 1.5552 .1214 -.4653 3.9414

Major_Ps -.4171 .7574 -.5507 .5825 -1.9103 1.0761

Chemo_co -.8944 .4741 -1.8865 .0606 -1.8290 .0403

Mastecto -1.0748 .4452 -2.4143 .0166 -1.9525 -.1971

Radiothe .2464 .8025 .3070 .7591 -1.3357 1.8285

| **Benjamini-Hochberg Procedure** | | | | |
| --- | --- | --- | --- | --- |
|  |  | **Ascending P-value s** | **I = ranking** | **(I/12)* 0.20** |
|  | **Stigma** | **.0000** | **1** | **.0166** |
|  | **Humor** | **.0049** | **2** | **.033** |
|  | **Mastectomy** | **.0166** | **3** | **.050** |
|  | **Social participation frequency** | **.0381** | **4** | **.066** |
|  | **Chemotherapy** | **.0606** | **5** | **.0833** |
|  | **Interaction Effect** | **.0982** | **6** | **.10** |
|  | **Insurance** | **~~.1214~~** | **7** | **.116** |
|  | **Education** | **~~.1795~~** | **8** | **.133** |
|  | **Age** | **~~.5329~~** | **9** | **.15** |
|  | **Major psychological problems** | **~~.5825~~** | **10** | **.166** |
|  | **Radiotherapy** | **~~.7591~~** | **11** | **.183** |
|  | **Employment** | **~~.7655~~** | **12** | **.20** |

Product terms key:

Int_1 : Stigma_I x copHumor

Test(s) of highest order unconditional interaction(s):

R2-chng F df1 df2 p

X*W .0102 2.7598 1.0000 207.0000 .0982

----------

Focal predict: Stigma_I (X)

Mod var: copHumor (W)

Conditional effects of the focal predictor at values of the moderator(s):

copHumor Effect se t p LLCI ULCI

.2960 -.4475 .0904 -4.9505 .0000 -.6257 -.2693

.5023 -.3459 .0667 -5.1832 .0000 -.4775 -.2143

.7253 -.2360 .0941 -2.5089 .0129 -.4215 -.0506

Moderator value(s) defining Johnson-Neyman significance region(s):

Value % below % above

.7798 86.3636 13.6364

Conditional effect of focal predictor at values of the moderator:

copHumor Effect se t p LLCI ULCI

-.1890 -.6864 .2154 -3.1869 .0017 -1.1111 -.2618

-.1267 -.6557 .1979 -3.3133 .0011 -1.0459 -.2656

-.0644 -.6251 .1806 -3.4604 .0007 -.9812 -.2689

-.0021 -.5944 .1636 -3.6329 .0004 -.9169 -.2718

.0602 -.5637 .1469 -3.8362 .0002 -.8534 -.2740

.1225 -.5330 .1307 -4.0765 .0001 -.7908 -.2752

.1848 -.5023 .1153 -4.3584 .0000 -.7295 -.2751

.2471 -.4716 .1008 -4.6806 .0000 -.6703 -.2730

.3094 -.4409 .0878 -5.0233 .0000 -.6140 -.2679

.3717 -.4103 .0771 -5.3232 .0000 -.5622 -.2583

.4339 -.3796 .0697 -5.4465 .0000 -.5170 -.2422

.4962 -.3489 .0668 -5.2264 .0000 -.4805 -.2173

.5585 -.3182 .0688 -4.6228 .0000 -.4539 -.1825

.6208 -.2875 .0755 -3.8075 .0002 -.4364 -.1386

.6831 -.2568 .0857 -2.9960 .0031 -.4258 -.0878

.7454 -.2261 .0984 -2.2988 .0225 -.4201 -.0322

.7798 -.2092 .1061 -1.9715 .0500 -.4184 .0000

.8077 -.1955 .1126 -1.7351 .0842 -.4175 .0266

.8700 -.1648 .1280 -1.2873 .1994 -.4171 .0876

.9323 -.1341 .1441 -.9306 .3531 -.4181 .1500

.9946 -.1034 .1607 -.6435 .5206 -.4202 .2134

1.0569 -.0727 .1776 -.4093 .6827 -.4229 .2775

Data for visualizing the conditional effect of the focal predictor:

Paste text below into a SPSS syntax window and execute to produce plot.

DATA LIST FREE/

Stigma_I copHumor FWB .

BEGIN DATA.

7.2796 .2960 19.5817

11.2836 .2960 17.7899

17.3587 .2960 15.0712

7.2796 .5023 19.9069

11.2836 .5023 18.5220

17.3587 .5023 16.4206

7.2796 .7253 20.2587

11.2836 .7253 19.3136

17.3587 .7253 17.8796

END DATA.

GRAPH/SCATTERPLOT=

Stigma_I WITH FWB BY copHumor .

*********************** ANALYSIS NOTES AND ERRORS ************************

Level of confidence for all confidence intervals in output:

95.0000

W values in conditional tables are the 16th, 50th, and 84th percentiles.

**************************************************************************

Model : 1

Y : FWB

X : Stigma_I

W : copUIS

Covariates:

Age_cov Educatio Employme Social_p Insuranc Major_Ps Chemo_co Mastecto Radiothe

Sample

Size: 220

**************************************************************************

OUTCOME VARIABLE:

FWB

Model Summary

R R-sq MSE F df1 df2 p

.4845 .2347 21.0442 5.2900 12.0000 207.0000 .0000

Model

coeff se t p LLCI ULCI

constant 21.4574 2.6557 8.0797 .0000 16.2217 26.6932

Stigma_I -.2888 .0678 -4.2620 .0000 -.4224 -.1552

copUIS .9235 .3497 2.6410 .0089 .2341 1.6128

Int_1 .1283 .0692 1.8548 .0651 -.0081 .2647

Age_cov -.0295 .0353 -.8365 .4039 -.0990 .0400

Educatio -1.2345 .9400 -1.3134 .1905 -3.0877 .6186

Employme .0139 1.0010 .0139 .9889 -1.9596 1.9875

Social_p .4275 .2208 1.9361 .0542 -.0078 .8628

Insuranc 1.1761 1.1073 1.0622 .2894 -1.0068 3.3591

Major_Ps -.5124 .7512 -.6821 .4959 -1.9933 .9686

Chemo_co -.8337 .4722 -1.7657 .0789 -1.7646 .0972

Mastecto -1.0322 .4439 -2.3256 .0210 -1.9073 -.1572

Radiothe .3593 .8010 .4486 .6542 -1.2199 1.9385

| **Benjamini-Hochberg Procedure** | | | | |
| --- | --- | --- | --- | --- |
|  |  | **Ascending P-value s** | **I = ranking** | **(I/12)* 0.20** |
|  | **Stigma** | **.0000** | **1** | **.0166** |
|  | **Use of instrumental support** | **.0089** | **2** | **.033** |
|  | **Mastectomy** | **.0210** | **3** | **.050** |
|  | **Social participation frequency** | **.0542** | **4** | **.066** |
|  | **Interaction Effect** | **.0651** | **5** | **.0833** |
|  | **Chemotherapy** | **.0789** | **6** | **.10** |
|  | **Education** | **~~.1905~~** | **7** | **.116** |
|  | **Insurance** | **~~.2894~~** | **8** | **.133** |
|  | **Age** | **~~.4039~~** | **9** | **.15** |
|  | **Major psychological problems** | **~~.4959~~** | **10** | **.166** |
|  | **Radiotherapy** | **~~.6542~~** | **11** | **.183** |
|  | **Employment** | **~~.9889~~** | **12** | **.20** |

Product terms key:

Int_1 : Stigma_I x copUIS

Test(s) of highest order unconditional interaction(s):

R2-chng F df1 df2 p

X*W .0127 3.4402 1.0000 207.0000 .0651

----------

Focal predict: Stigma_I (X)

Mod var: copUIS (W)

Conditional effects of the focal predictor at values of the moderator(s):

copUIS Effect se t p LLCI ULCI

2.0000 -.3880 .0782 -4.9603 .0000 -.5422 -.2338

3.0000 -.2597 .0723 -3.5900 .0004 -.4023 -.1171

4.0000 -.1313 .1180 -1.1133 .2669 -.3639 .1012

Moderator value(s) defining Johnson-Neyman significance region(s):

Value % below % above

3.5673 79.5455 20.4545

Conditional effect of focal predictor at values of the moderator:

copUIS Effect se t p LLCI ULCI

1.0000 -.5163 .1288 -4.0100 .0001 -.7702 -.2625

1.1500 -.4971 .1200 -4.1424 .0001 -.7336 -.2605

1.3000 -.4778 .1115 -4.2849 .0000 -.6977 -.2580

1.4500 -.4586 .1034 -4.4359 .0000 -.6624 -.2548

1.6000 -.4393 .0957 -4.5918 .0000 -.6279 -.2507

1.7500 -.4201 .0885 -4.7455 .0000 -.5946 -.2456

1.9000 -.4008 .0821 -4.8846 .0000 -.5626 -.2390

2.0500 -.3816 .0765 -4.9903 .0000 -.5323 -.2308

2.2000 -.3623 .0719 -5.0366 .0000 -.5042 -.2205

2.3500 -.3431 .0687 -4.9942 .0000 -.4785 -.2076

2.5000 -.3238 .0669 -4.8390 .0000 -.4558 -.1919

2.6500 -.3046 .0667 -4.5642 .0000 -.4361 -.1730

2.8000 -.2853 .0681 -4.1873 .0000 -.4197 -.1510

2.9500 -.2661 .0711 -3.7448 .0002 -.4062 -.1260

3.1000 -.2468 .0753 -3.2781 .0012 -.3953 -.0984

3.2500 -.2276 .0807 -2.8216 .0052 -.3866 -.0686

3.4000 -.2083 .0869 -2.3965 .0174 -.3797 -.0369

3.5500 -.1891 .0939 -2.0129 .0454 -.3743 -.0039

3.5673 -.1869 .0948 -1.9715 .0500 -.3737 .0000

3.7000 -.1698 .1015 -1.6729 .0959 -.3700 .0303

3.8500 -.1506 .1096 -1.3744 .1708 -.3666 .0654

4.0000 -.1313 .1180 -1.1133 .2669 -.3639 .1012

Data for visualizing the conditional effect of the focal predictor:

Paste text below into a SPSS syntax window and execute to produce plot.

DATA LIST FREE/

Stigma_I copUIS FWB .

BEGIN DATA.

7.2796 2.0000 19.5331

11.2836 2.0000 17.9796

17.3587 2.0000 15.6226

7.2796 3.0000 19.8498

11.2836 3.0000 18.8101

17.3587 3.0000 17.2326

7.2796 4.0000 20.1665

11.2836 4.0000 19.6406

17.3587 4.0000 18.8426

END DATA.

GRAPH/SCATTERPLOT=

Stigma_I WITH FWB BY copUIS .

*********************** ANALYSIS NOTES AND ERRORS ************************

Level of confidence for all confidence intervals in output:

95.0000

W values in conditional tables are the 16th, 50th, and 84th percentiles.

**************************************************************************

Model : 1

Y : FWB

X : Stigma_I

W : copBehav

Covariates:

Age_cov Educatio Employme Social_p Insuranc Major_Ps Chemo_co Mastecto Radiothe

Sample

Size: 219

**************************************************************************

OUTCOME VARIABLE:

FWB

Model Summary

R R-sq MSE F df1 df2 p

.4646 .2158 21.6163 4.7253 12.0000 206.0000 .0000

Model

coeff se t p LLCI ULCI

constant 20.6755 2.7105 7.6278 .0000 15.3316 26.0195

Stigma_I -.3362 .0706 -4.7658 .0000 -.4753 -.1971

copBehav -2.7118 2.6240 -1.0334 .3026 -7.8851 2.4616

Int_1 1.0741 .4912 2.1869 .0299 .1058 2.0425

Age_cov -.0324 .0361 -.8974 .3706 -.1036 .0388

Educatio -1.1920 .9532 -1.2505 .2125 -3.0712 .6872

Employme -.3564 1.0189 -.3498 .7268 -2.3652 1.6524

Social_p .5069 .2238 2.2650 .0246 .0657 .9481

Insuranc 1.2844 1.1198 1.1470 .2527 -.9233 3.4922

Major_Ps -.6989 .7594 -.9203 .3585 -2.1960 .7983

Chemo_co -.4904 .4819 -1.0175 .3101 -1.4405 .4597

Mastecto -.9945 .4550 -2.1858 .0300 -1.8915 -.0975

Radiothe .2334 .8145 .2866 .7747 -1.3724 1.8392

| **Benjamini-Hochberg Procedure** | | | | |
| --- | --- | --- | --- | --- |
|  |  | **Ascending P-value s** | **I = ranking** | **(I/12)* 0.20** |
|  | **Stigma** | **.0000** | **1** | **.0166** |
|  | **Social participation frequency** | **.0246** | **2** | **.033** |
|  | **Interaction Effect** | **.0299** | **3** | **.050** |
|  | **Mastectomy** | **.0300** | **4** | **.066** |
|  | **Education** | **~~.2125~~** | **5** | **.0833** |
|  | **Insurance** | **~~.2527~~** | **6** | **.10** |
|  | **Chemotherapy** | **~~.3101~~** | **7** | **.116** |
|  | **Behavioral disengagement** | **~~.3026~~** | **8** | **.133** |
|  | **Major psychological problems** | **~~.3585~~** | **9** | **.15** |
|  | **Age** | **~~.3706~~** | **10** | **.166** |
|  | **Employment** | **~~.7268~~** | **11** | **.183** |
|  | **Radiotherapy** | **~~.7747~~** | **12** | **.20** |

Product terms key:

Int_1 : Stigma_I x copBehav

Test(s) of highest order unconditional interaction(s):

R2-chng F df1 df2 p

X*W .0182 4.7827 1.0000 206.0000 .0299

----------

Focal predict: Stigma_I (X)

Mod var: copBehav (W)

Conditional effects of the focal predictor at values of the moderator(s):

copBehav Effect se t p LLCI ULCI

.4061 -.4512 .0949 -4.7557 .0000 -.6383 -.2642

.4061 -.4512 .0949 -4.7557 .0000 -.6383 -.2642

.6802 -.1567 .0987 -1.5876 .1139 -.3514 .0379

Moderator value(s) defining Johnson-Neyman significance region(s):

Value % below % above

.6583 80.3653 19.6347

Conditional effect of focal predictor at values of the moderator:

copBehav Effect se t p LLCI ULCI

.4061 -.4512 .0949 -4.7557 .0000 -.6383 -.2642

.4293 -.4262 .0875 -4.8709 .0000 -.5987 -.2537

.4526 -.4012 .0811 -4.9489 .0000 -.5610 -.2414

.4759 -.3762 .0758 -4.9613 .0000 -.5257 -.2267

.4992 -.3512 .0720 -4.8754 .0000 -.4932 -.2092

.5225 -.3262 .0699 -4.6648 .0000 -.4640 -.1883

.5458 -.3011 .0696 -4.3238 .0000 -.4385 -.1638

.5691 -.2761 .0712 -3.8763 .0001 -.4166 -.1357

.5923 -.2511 .0746 -3.3678 .0009 -.3981 -.1041

.6156 -.2261 .0794 -2.8472 .0049 -.3827 -.0695

.6389 -.2011 .0855 -2.3512 .0197 -.3697 -.0325

.6583 -.1802 .0914 -1.9715 .0500 -.3605 .0000

.6622 -.1761 .0927 -1.9004 .0588 -.3588 .0066

.6855 -.1511 .1006 -1.5020 .1346 -.3494 .0472

.7088 -.1261 .1091 -1.1551 .2494 -.3412 .0891

.7320 -.1010 .1182 -.8551 .3935 -.3340 .1319

.7553 -.0760 .1276 -.5959 .5519 -.3276 .1755

.7786 -.0510 .1373 -.3716 .7106 -.3218 .2197

.8019 -.0260 .1473 -.1766 .8600 -.3164 .2644

.8252 -.0010 .1575 -.0064 .9949 -.3115 .3095

.8485 .0240 .1678 .1431 .8864 -.3069 .3549

.8718 .0490 .1783 .2749 .7836 -.3025 .4005

Data for visualizing the conditional effect of the focal predictor:

Paste text below into a SPSS syntax window and execute to produce plot.

DATA LIST FREE/

Stigma_I copBehav FWB .

BEGIN DATA.

7.2796 .4061 20.5910

11.2836 .4061 18.7843

17.3587 .4061 16.0432

7.2796 .4061 20.5910

11.2836 .4061 18.7843

17.3587 .4061 16.0432

7.2796 .6802 18.4487

11.2836 .6802 17.8212

17.3587 .6802 16.8690

END DATA.

GRAPH/SCATTERPLOT=

Stigma_I WITH FWB BY copBehav .

*********************** ANALYSIS NOTES AND ERRORS ************************

Level of confidence for all confidence intervals in output:

95.0000

W values in conditional tables are the 16th, 50th, and 84th percentiles.

**************************************************************************

Model : 1

Y : FWB

X : Stigma_I

W : copVenti

Covariates:

Age_cov Educatio Employme Social_p Insuranc Major_Ps Chemo_co Mastecto Radiothe

Sample

Size: 219

**************************************************************************

OUTCOME VARIABLE:

FWB

Model Summary

R R-sq MSE F df1 df2 p

.4695 .2205 21.4887 4.8552 12.0000 206.0000 .0000

Model

coeff se t p LLCI ULCI

constant 21.4118 2.6894 7.9616 .0000 16.1095 26.7141

Stigma_I -.3378 .0679 -4.9766 .0000 -.4716 -.2040

copVenti .0747 .3687 .2027 .8396 -.6522 .8017

Int_1 .2113 .0818 2.5845 .0104 .0501 .3726

Age_cov -.0364 .0359 -1.0135 .3120 -.1072 .0344

Educatio -1.0567 .9517 -1.1103 .2682 -2.9331 .8197

Employme -.4165 1.0110 -.4120 .6808 -2.4098 1.5767

Social_p .4585 .2234 2.0527 .0414 .0181 .8989

Insuranc 1.1623 1.1170 1.0405 .2993 -1.0400 3.3645

Major_Ps -.6903 .7567 -.9123 .3627 -2.1821 .8015

Chemo_co -.6598 .4756 -1.3871 .1669 -1.5975 .2780

Mastecto -1.0394 .4471 -2.3245 .0211 -1.9209 -.1578

Radiothe .2292 .8157 .2810 .7790 -1.3789 1.8373

| **Benjamini-Hochberg Procedure** | | | | |
| --- | --- | --- | --- | --- |
|  |  | **Ascending P-value s** | **I = ranking** | **(I/12)* 0.20** |
|  | **Stigma** | **.0000** | **1** | **.0166** |
|  | **Interaction Effect** | **.0104** | **2** | **.033** |
|  | **Mastectomy** | **.0211** | **3** | **.050** |
|  | **Social participation frequency** | **.0414** | **4** | **.066** |
|  | **Chemotherapy** | **~~.1669~~** | **5** | **.0833** |
|  | **Insurance** | **~~.2993~~** | **6** | **.10** |
|  | **Education** | **~~.2682~~** | **7** | **.116** |
|  | **Age** | **~~.3120~~** | **8** | **.133** |
|  | **Major psychological problems** | **~~.3627~~** | **9** | **.15** |
|  | **Employment** | **~~.6808~~** | **10** | **.166** |
|  | **Radiotherapy** | **~~.7790~~** | **11** | **.183** |
|  | **Venting** | **~~.8396~~** | **12** | **.20** |

Product terms key:

Int_1 : Stigma_I x copVenti

Test(s) of highest order unconditional interaction(s):

R2-chng F df1 df2 p

X*W .0253 6.6796 1.0000 206.0000 .0104

----------

Focal predict: Stigma_I (X)

Mod var: copVenti (W)

Conditional effects of the focal predictor at values of the moderator(s):

copVenti Effect se t p LLCI ULCI

1.5000 -.5250 .1030 -5.0996 .0000 -.7280 -.3220

2.5000 -.3137 .0678 -4.6256 .0000 -.4474 -.1800

3.5000 -.1024 .1094 -.9354 .3507 -.3181 .1134

Moderator value(s) defining Johnson-Neyman significance region(s):

Value % below % above

3.1545 82.6484 17.3516

Conditional effect of focal predictor at values of the moderator:

copVenti Effect se t p LLCI ULCI

1.0000 -.6307 .1364 -4.6226 .0000 -.8997 -.3617

1.1500 -.5990 .1259 -4.7564 .0000 -.8473 -.3507

1.3000 -.5673 .1158 -4.8998 .0000 -.7956 -.3390

1.4500 -.5356 .1061 -5.0494 .0000 -.7447 -.3265

1.6000 -.5039 .0969 -5.1979 .0000 -.6950 -.3128

1.7500 -.4722 .0886 -5.3309 .0000 -.6468 -.2976

1.9000 -.4405 .0812 -5.4243 .0000 -.6006 -.2804

2.0500 -.4088 .0751 -5.4412 .0000 -.5569 -.2607

2.2000 -.3771 .0707 -5.3355 .0000 -.5164 -.2378

2.3500 -.3454 .0682 -5.0669 .0000 -.4798 -.2110

2.5000 -.3137 .0678 -4.6256 .0000 -.4474 -.1800

2.6500 -.2820 .0697 -4.0482 .0001 -.4193 -.1447

2.8000 -.2503 .0735 -3.4039 .0008 -.3953 -.1053

2.9500 -.2186 .0791 -2.7624 .0063 -.3746 -.0626

3.1000 -.1869 .0861 -2.1699 .0312 -.3567 -.0171

3.1545 -.1754 .0889 -1.9715 .0500 -.3507 .0000

3.2500 -.1552 .0942 -1.6473 .1010 -.3409 .0305

3.4000 -.1235 .1031 -1.1976 .2325 -.3268 .0798

3.5500 -.0918 .1127 -.8147 .4162 -.3139 .1303

3.7000 -.0601 .1227 -.4898 .6248 -.3020 .1818

3.8500 -.0284 .1331 -.2133 .8313 -.2908 .2340

4.0000 .0033 .1438 .0231 .9816 -.2801 .2868

Data for visualizing the conditional effect of the focal predictor:

Paste text below into a SPSS syntax window and execute to produce plot.

DATA LIST FREE/

Stigma_I copVenti FWB .

BEGIN DATA.

7.2796 1.5000 20.6584

11.2836 1.5000 18.5562

17.3587 1.5000 15.3666

7.2796 2.5000 19.7292

11.2836 2.5000 18.4732

17.3587 2.5000 16.5675

7.2796 3.5000 18.8000

11.2836 3.5000 18.3902

17.3587 3.5000 17.7685

END DATA.

GRAPH/SCATTERPLOT=

Stigma_I WITH FWB BY copVenti .

*********************** ANALYSIS NOTES AND ERRORS ************************

Level of confidence for all confidence intervals in output:

95.0000

W values in conditional tables are the 16th, 50th, and 84th percentiles.

**************************************************************************

Model : 1

Y : Arm.norm

X : Stigma_I

W : copVenti

Covariates:

Age_cov Educatio Employme Social_p Insuranc Major_Ps Chemo_co Mastecto Radiothe

Sample

Size: 153

**************************************************************************

OUTCOME VARIABLE:

Arm.norm

Model Summary

R R-sq MSE F df1 df2 p

.4466 .1995 .0633 2.9068 12.0000 140.0000 .0013

Model

coeff se t p LLCI ULCI

constant .5451 .1747 3.1195 .0022 .1996 .8905

Stigma_I -.0106 .0042 -2.4985 .0136 -.0190 -.0022

copVenti .0013 .0245 .0546 .9565 -.0470 .0497

Int_1 -.0087 .0050 -1.7480 .0827 -.0186 .0011

Age_cov -.0005 .0024 -.1960 .8449 -.0053 .0043

Educatio -.1385 .0637 -2.1739 .0314 -.2645 -.0125

Employme -.0498 .0681 -.7306 .4662 -.1844 .0849

Social_p -.0003 .0133 -.0234 .9813 -.0267 .0260

Insuranc .0744 .0695 1.0700 .2864 -.0631 .2119

Major_Ps -.0044 .0491 -.0893 .9289 -.1014 .0927

Chemo_co .0029 .0300 .0953 .9242 -.0565 .0622

Mastecto -.0983 .0436 -2.2547 .0257 -.1846 -.0121

Radiothe .0924 .0482 1.9157 .0574 -.0030 .1877

| **Benjamini-Hochberg Procedure** | | | | |
| --- | --- | --- | --- | --- |
|  |  | **Ascending P-value s** | **I = ranking** | **(I/12)* 0.20** |
|  | **Stigma** | **.0136** | **1** | **.0166** |
|  | **Mastectomy** | **.0257** | **2** | **.033** |
|  | **Education** | **.0314** | **3** | **.050** |
|  | **Radiotherapy** | **.0574** | **4** | **.066** |
|  | **Interaction Effect** | **.0827** | **5** | **.0833** |
|  | **Insurance** | **~~.2864~~** | **6** | **.10** |
|  | **Employment** | **~~.4662~~** | **7** | **.116** |
|  | **Age** | **~~.8449~~** | **8** | **.133** |
|  | **Chemotherapy** | **~~.9242~~** | **9** | **.15** |
|  | **Major psychological problems** | **~~.9289~~** | **10** | **.166** |
|  | **Venting** | **~~.9565~~** | **11** | **.183** |
|  | **Social participation frequency** | **~~.9813~~** | **12** | **.20** |

Product terms key:

Int_1 : Stigma_I x copVenti

Test(s) of highest order unconditional interaction(s):

R2-chng F df1 df2 p

X*W .0175 3.0556 1.0000 140.0000 .0827

----------

Focal predict: Stigma_I (X)

Mod var: copVenti (W)

Conditional effects of the focal predictor at values of the moderator(s):

copVenti Effect se t p LLCI ULCI

1.5000 -.0028 .0064 -.4433 .6583 -.0154 .0098

2.5000 -.0116 .0042 -2.7225 .0073 -.0199 -.0032

3.5000 -.0203 .0067 -3.0117 .0031 -.0336 -.0070

Moderator value(s) defining Johnson-Neyman significance region(s):

Value % below % above

2.1831 47.0588 52.9412

Conditional effect of focal predictor at values of the moderator:

copVenti Effect se t p LLCI ULCI

1.0000 .0015 .0084 .1837 .8545 -.0151 .0181

1.1500 .0002 .0078 .0300 .9761 -.0151 .0156

1.3000 -.0011 .0071 -.1507 .8805 -.0152 .0131

1.4500 -.0024 .0066 -.3639 .7165 -.0154 .0106

1.6000 -.0037 .0060 -.6155 .5392 -.0156 .0082

1.7500 -.0050 .0055 -.9103 .3643 -.0159 .0059

1.9000 -.0063 .0051 -1.2494 .2136 -.0163 .0037

2.0500 -.0076 .0047 -1.6264 .1061 -.0169 .0016

2.1831 -.0088 .0044 -1.9771 .0500 -.0176 .0000

2.2000 -.0089 .0044 -2.0215 .0451 -.0177 -.0002

2.3500 -.0102 .0043 -2.4006 .0177 -.0187 -.0018

2.5000 -.0116 .0042 -2.7225 .0073 -.0199 -.0032

2.6500 -.0129 .0044 -2.9564 .0037 -.0215 -.0043

2.8000 -.0142 .0046 -3.0945 .0024 -.0232 -.0051

2.9500 -.0155 .0049 -3.1509 .0020 -.0252 -.0058

3.1000 -.0168 .0053 -3.1491 .0020 -.0273 -.0063

3.2500 -.0181 .0058 -3.1115 .0023 -.0296 -.0066

3.4000 -.0194 .0064 -3.0547 .0027 -.0320 -.0068

3.5500 -.0207 .0069 -2.9896 .0033 -.0344 -.0070

3.7000 -.0220 .0075 -2.9226 .0040 -.0369 -.0071

3.8500 -.0233 .0082 -2.8574 .0049 -.0395 -.0072

4.0000 -.0247 .0088 -2.7955 .0059 -.0421 -.0072

Data for visualizing the conditional effect of the focal predictor:

Paste text below into a SPSS syntax window and execute to produce plot.

DATA LIST FREE/

Stigma_I copVenti Arm.norm .

BEGIN DATA.

7.2796 1.5000 .4851

11.2836 1.5000 .4738

18.2634 1.5000 .4541

7.2796 2.5000 .5309

11.2836 2.5000 .4846

18.2634 2.5000 .4040

7.2796 3.5000 .5766

11.2836 3.5000 .4954

18.2634 3.5000 .3538

END DATA.

GRAPH/SCATTERPLOT=

Stigma_I WITH Arm.norm BY copVenti .

*********************** ANALYSIS NOTES AND ERRORS ************************

Level of confidence for all confidence intervals in output:

95.0000

**************************************************************************

Model : 1

Y : FACT_G

X : Stigma_I

W : Comprehe

Covariates:

Age_cov Educatio Employme Social_p Insuranc Major_Ps Chemo_co Mastecto Radiothe

Sample

Size: 221

**************************************************************************

OUTCOME VARIABLE:

FACT_G

Model Summary

R R-sq MSE F df1 df2 p

.5655 .3198 193.8637 8.1506 12.0000 208.0000 .0000

Model

coeff se t p LLCI ULCI

constant 78.6397 8.0693 9.7456 .0000 62.7316 94.5477

Stigma_I -1.4577 .2089 -6.9770 .0000 -1.8696 -1.0458

Comprehe .3233 .1592 2.0305 .0436 .0094 .6373

Int_1 -.0593 .0307 -1.9299 .0550 -.1199 .0013

Age_cov -.1360 .1068 -1.2729 .2045 -.3466 .0746

Educatio -3.9108 2.8513 -1.3716 .1717 -9.5321 1.7104

Employme -3.8152 3.0341 -1.2574 .2100 -9.7967 2.1664

Social_p 1.3263 .6678 1.9862 .0483 .0098 2.6428

Insuranc 3.7789 3.3539 1.1267 .2612 -2.8331 10.3910

Major_Ps -4.7116 2.2772 -2.0690 .0398 -9.2010 -.2222

Chemo_co -1.1984 1.4362 -.8344 .4050 -4.0297 1.6329

Mastecto -.9815 1.3596 -.7219 .4711 -3.6618 1.6988

Radiothe -.3472 2.4438 -.1421 .8872 -5.1650 4.4706

| **Benjamini-Hochberg Procedure** | | | | |
| --- | --- | --- | --- | --- |
|  |  | **Ascending P-value s** | **I = ranking** | **(I/12)* 0.10** |
|  | **Stigma** | **.0000** | **1** | **.0166** |
|  | **Major psychological problems** | **.0398** | **2** | **.033** |
|  | **Comprehensibility** | **.0436** | **3** | **.050** |
|  | **Social participation frequency** | **.0483** | **4** | **.066** |
|  | **Interaction Effect** | **.0550** | **5** | **.0833** |
|  | **Education** | **~~.1717~~** | **6** | **.10** |
|  | **Age** | **~~.2045~~** | **7** | **.116** |
|  | **Employment** | **~~.2100~~** | **8** | **.133** |
|  | **Insurance** | **~~.2612~~** | **9** | **.15** |
|  | **Chemotherapy** | **~~.4050~~** | **10** | **.166** |
|  | **Mastectomy** | **~~.4711~~** | **11** | **.183** |
|  | **Radiotherapy** | **~~.8872~~** | **12** | **.20** |

Product terms key:

Int_1 : Stigma_I x Comprehe

Test(s) of highest order unconditional interaction(s):

R2-chng F df1 df2 p

X*W .0122 3.7247 1.0000 208.0000 .0550

----------

Focal predict: Stigma_I (X)

Mod var: Comprehe (W)

Conditional effects of the focal predictor at values of the moderator(s):

Comprehe Effect se t p LLCI ULCI

13.5200 -1.0475 .2755 -3.8023 .0002 -1.5907 -.5044

21.0000 -1.4913 .2122 -7.0290 .0000 -1.9095 -1.0730

27.0000 -1.8472 .3109 -5.9418 .0000 -2.4601 -1.2343

Moderator value(s) defining Johnson-Neyman significance region(s):

Value % below % above

8.7590 3.6199 96.3801

Conditional effect of focal predictor at values of the moderator:

Comprehe Effect se t p LLCI ULCI

5.0000 -.5421 .4898 -1.1069 .2696 -1.5077 .4234

6.5000 -.6311 .4484 -1.4075 .1608 -1.5151 .2529

8.0000 -.7201 .4080 -1.7648 .0791 -1.5245 .0843

8.7590 -.7651 .3881 -1.9714 .0500 -1.5302 .0000

9.5000 -.8091 .3690 -2.1925 .0295 -1.5366 -.0816

11.0000 -.8980 .3318 -2.7063 .0074 -1.5522 -.2439

12.5000 -.9870 .2972 -3.3216 .0011 -1.5729 -.4012

14.0000 -1.0760 .2660 -4.0455 .0001 -1.6004 -.5517

15.5000 -1.1650 .2397 -4.8611 .0000 -1.6375 -.6925

17.0000 -1.2540 .2199 -5.7012 .0000 -1.6876 -.8204

18.5000 -1.3430 .2087 -6.4337 .0000 -1.7545 -.9314

20.0000 -1.4319 .2074 -6.9040 .0000 -1.8408 -1.0230

21.5000 -1.5209 .2161 -7.0368 .0000 -1.9470 -1.0948

23.0000 -1.6099 .2338 -6.8857 .0000 -2.0708 -1.1490

24.5000 -1.6989 .2586 -6.5700 .0000 -2.2086 -1.1891

26.0000 -1.7879 .2886 -6.1940 .0000 -2.3569 -1.2188

27.5000 -1.8768 .3225 -5.8193 .0000 -2.5127 -1.2410

29.0000 -1.9658 .3591 -5.4739 .0000 -2.6738 -1.2578

30.5000 -2.0548 .3977 -5.1665 .0000 -2.8389 -1.2707

32.0000 -2.1438 .4378 -4.8971 .0000 -3.0068 -1.2808

33.5000 -2.2328 .4789 -4.6623 .0000 -3.1769 -1.2886

35.0000 -2.3217 .5209 -4.4574 .0000 -3.3486 -1.2949

Data for visualizing the conditional effect of the focal predictor:

Paste text below into a SPSS syntax window and execute to produce plot.

DATA LIST FREE/

Stigma_I Comprehe FACT_G .

BEGIN DATA.

7.2796 13.5200 71.3245

11.2836 13.5200 67.1302

17.3587 13.5200 60.7664

7.2796 21.0000 75.8556

11.2836 21.0000 69.8847

17.3587 21.0000 60.8252

7.2796 27.0000 79.4902

11.2836 27.0000 72.0942

17.3587 27.0000 60.8725

END DATA.

GRAPH/SCATTERPLOT=

Stigma_I WITH FACT_G BY Comprehe .

*********************** ANALYSIS NOTES AND ERRORS ************************

Level of confidence for all confidence intervals in output:

95.0000

**************************************************************************

Model : 1

Y : FACT_G

X : Stigma_I

W : copBehav

Covariates:

Age_cov Educatio Employme Social_p Insuranc Major_Ps Chemo_co Mastecto Radiothe

Sample

Size: 219

**************************************************************************

OUTCOME VARIABLE:

FACT_G

Model Summary

R R-sq MSE F df1 df2 p

.5564 .3095 197.9405 7.6957 12.0000 206.0000 .0000

Model

coeff se t p LLCI ULCI

constant 77.4052 8.2023 9.4371 .0000 61.2341 93.5764

Stigma_I -1.5779 .2135 -7.3908 .0000 -1.9988 -1.1570

copBehav -6.3341 7.9404 -.7977 .4260 -21.9891 9.3208

Int_1 3.4252 1.4863 2.3045 .0222 .4949 6.3555

Age_cov -.0764 .1093 -.6992 .4852 -.2918 .1390

Educatio -3.9339 2.8843 -1.3639 .1741 -9.6205 1.7527

Employme -3.2842 3.0832 -1.0652 .2880 -9.3628 2.7945

Social_p 1.4741 .6772 2.1767 .0306 .1389 2.8093

Insuranc 3.5438 3.3886 1.0458 .2969 -3.1370 10.2247

Major_Ps -5.5189 2.2979 -2.4017 .0172 -10.0493 -.9885

Chemo_co -1.3100 1.4583 -.8983 .3701 -4.1850 1.5651

Mastecto -1.3744 1.3768 -.9982 .3193 -4.0888 1.3401

Radiothe -.7931 2.4647 -.3218 .7479 -5.6523 4.0661

| **Benjamini-Hochberg Procedure** | | | | |
| --- | --- | --- | --- | --- |
|  |  | **Ascending P-value s** | **I = ranking** | **(I/12)* 0.20** |
|  | **Stigma** | **.0000** | **1** | **.0166** |
|  | **Major psychological problems** | **.0172** | **2** | **.033** |
|  | **Interaction Effect** | **.0222** | **3** | **.050** |
|  | **Social participation frequency** | **.0306** | **4** | **.066** |
|  | **Education** | **~~.1741~~** | **5** | **.0833** |
|  | **Employment** | **~~.2880~~** | **6** | **.10** |
|  | **Insurance** | **~~.2969~~** | **7** | **.116** |
|  | **Mastectomy** | **~~.3193~~** | **8** | **.133** |
|  | **Chemotherapy** | **~~.3701~~** | **9** | **.15** |
|  | **Behavioral disengagement** | **~~.4260~~** | **10** | **.166** |
|  | **Age** | **~~.4852~~** | **11** | **.183** |
|  | **Radiotherapy** | **~~.7479~~** | **12** | **.20** |

Product terms key:

Int_1 : Stigma_I x copBehav

Test(s) of highest order unconditional interaction(s):

R2-chng F df1 df2 p

X*W .0178 5.3109 1.0000 206.0000 .0222

----------

Focal predict: Stigma_I (X)

Mod var: copBehav (W)

Conditional effects of the focal predictor at values of the moderator(s):

copBehav Effect se t p LLCI ULCI

.4061 -1.9445 .2871 -6.7729 .0000 -2.5106 -1.3785

.4061 -1.9445 .2871 -6.7729 .0000 -2.5106 -1.3785

.6802 -1.0055 .2988 -3.3657 .0009 -1.5945 -.4165

Moderator value(s) defining Johnson-Neyman significance region(s):

Value % below % above

.7531 93.6073 6.3927

Conditional effect of focal predictor at values of the moderator:

copBehav Effect se t p LLCI ULCI

.4061 -1.9445 .2871 -6.7729 .0000 -2.5106 -1.3785

.4293 -1.8648 .2648 -7.0429 .0000 -2.3868 -1.3428

.4526 -1.7850 .2453 -7.2766 .0000 -2.2687 -1.3014

.4759 -1.7053 .2294 -7.4322 .0000 -2.1576 -1.2529

.4992 -1.6255 .2180 -7.4578 .0000 -2.0552 -1.1958

.5225 -1.5457 .2116 -7.3059 .0000 -1.9629 -1.1286

.5458 -1.4660 .2108 -6.9559 .0000 -1.8815 -1.0505

.5691 -1.3862 .2156 -6.4309 .0000 -1.8112 -.9612

.5923 -1.3065 .2256 -5.7903 .0000 -1.7513 -.8616

.6156 -1.2267 .2403 -5.1047 .0000 -1.7005 -.7529

.6389 -1.1470 .2588 -4.4316 .0000 -1.6572 -.6367

.6622 -1.0672 .2804 -3.8062 .0002 -1.6200 -.5144

.6855 -.9875 .3044 -3.2442 .0014 -1.5875 -.3874

.7088 -.9077 .3302 -2.7485 .0065 -1.5588 -.2566

.7320 -.8279 .3576 -2.3153 .0216 -1.5330 -.1229

.7531 -.7558 .3834 -1.9715 .0500 -1.5116 .0000

.7553 -.7482 .3861 -1.9377 .0540 -1.5095 .0131

.7786 -.6684 .4156 -1.6085 .1093 -1.4878 .1509

.8019 -.5887 .4458 -1.3206 .1881 -1.4675 .2902

.8252 -.5089 .4766 -1.0679 .2868 -1.4485 .4306

.8485 -.4292 .5078 -.8451 .3990 -1.4304 .5721

.8718 -.3494 .5395 -.6476 .5179 -1.4131 .7143

Data for visualizing the conditional effect of the focal predictor:

Paste text below into a SPSS syntax window and execute to produce plot.

DATA LIST FREE/

Stigma_I copBehav FACT_G .

BEGIN DATA.

7.2796 .4061 78.5000

11.2836 .4061 70.7141

17.3587 .4061 58.9010

7.2796 .4061 78.5000

11.2836 .4061 70.7141

17.3587 .4061 58.9010

7.2796 .6802 72.3030

11.2836 .6802 68.2769

17.3587 .6802 62.1683

END DATA.

GRAPH/SCATTERPLOT=

Stigma_I WITH FACT_G BY copBehav .

*********************** ANALYSIS NOTES AND ERRORS ************************

Level of confidence for all confidence intervals in output:

95.0000

**************************************************************************

Model : 1

Y : FACT_G

X : Stigma_I

W : copVenti

Covariates:

Age_cov Educatio Employme Social_p Insuranc Major_Ps Chemo_co Mastecto Radiothe

Sample

Size: 219

**************************************************************************

OUTCOME VARIABLE:

FACT_G

Model Summary

R R-sq MSE F df1 df2 p

.5513 .3040 199.5383 7.4966 12.0000 206.0000 .0000

Model

coeff se t p LLCI ULCI

constant 79.3471 8.1953 9.6821 .0000 63.1898 95.5045

Stigma_I -1.5631 .2068 -7.5567 .0000 -1.9709 -1.1553

copVenti .5974 1.1236 .5317 .5955 -1.6178 2.8125

Int_1 .4790 .2492 1.9222 .0560 -.0123 .9702

Age_cov -.0872 .1094 -.7970 .4264 -.3029 .1285

Educatio -3.6611 2.9002 -1.2624 .2082 -9.3789 2.0568

Employme -3.3989 3.0808 -1.1033 .2712 -9.4729 2.6750

Social_p 1.3675 .6807 2.0091 .0458 .0256 2.7095

Insuranc 3.2485 3.4038 .9544 .3410 -3.4623 9.9594

Major_Ps -5.5252 2.3057 -2.3963 .0175 -10.0710 -.9793

Chemo_co -1.8643 1.4493 -1.2863 .1998 -4.7217 .9931

Mastecto -1.4366 1.3625 -1.0543 .2930 -4.1229 1.2497

Radiothe -.5988 2.4855 -.2409 .8099 -5.4992 4.3015

| **Benjamini-Hochberg Procedure** | | | | |
| --- | --- | --- | --- | --- |
|  |  | **Ascending P-value s** | **I = ranking** | **(I/12)* 0.20** |
|  | **Stigma** | **.0000** | **1** | **.0166** |
|  | **Major psychological problems** | **.0175** | **2** | **.033** |
|  | **Social participation frequency** | **.0458** | **3** | **.050** |
|  | **Interaction Effect** | **.0560** | **4** | **.066** |
|  | **Chemotherapy** | **~~.1998~~** | **5** | **.0833** |
|  | **Education** | **~~.2082~~** | **6** | **.10** |
|  | **Employment** | **~~.2712~~** | **7** | **.116** |
|  | **Mastectomy** | **~~.2930~~** | **8** | **.133** |
|  | **Insurance** | **~~.3410~~** | **9** | **.15** |
|  | **Age** | **~~.4264~~** | **10** | **.166** |
|  | **Venting** | **~~.5955~~** | **11** | **.183** |
|  | **Radiotherapy** | **~~.8099~~** | **12** | **.20** |

Product terms key:

Int_1 : Stigma_I x copVenti

Test(s) of highest order unconditional interaction(s):

R2-chng F df1 df2 p

X*W .0125 3.6947 1.0000 206.0000 .0560

----------

Focal predict: Stigma_I (X)

Mod var: copVenti (W)

Conditional effects of the focal predictor at values of the moderator(s):

copVenti Effect se t p LLCI ULCI

1.5000 -1.9874 .3137 -6.3347 .0000 -2.6059 -1.3688

2.5000 -1.5084 .2067 -7.2994 .0000 -1.9158 -1.1010

3.5000 -1.0295 .3334 -3.0876 .0023 -1.6868 -.3721

Moderator value(s) defining Johnson-Neyman significance region(s):

Value % below % above

3.9189 91.7808 8.2192

Conditional effect of focal predictor at values of the moderator:

copVenti Effect se t p LLCI ULCI

1.0000 -2.2269 .4158 -5.3561 .0000 -3.0465 -1.4072

1.1500 -2.1550 .3838 -5.6156 .0000 -2.9116 -1.3984

1.3000 -2.0832 .3528 -5.9046 .0000 -2.7787 -1.3876

1.4500 -2.0113 .3232 -6.2227 .0000 -2.6486 -1.3741

1.6000 -1.9395 .2954 -6.5654 .0000 -2.5219 -1.3571

1.7500 -1.8676 .2699 -6.9194 .0000 -2.3998 -1.3355

1.9000 -1.7958 .2475 -7.2569 .0000 -2.2837 -1.3079

2.0500 -1.7239 .2289 -7.5302 .0000 -2.1753 -1.2726

2.2000 -1.6521 .2154 -7.6712 .0000 -2.0767 -1.2275

2.3500 -1.5803 .2077 -7.6078 .0000 -1.9898 -1.1707

2.5000 -1.5084 .2067 -7.2994 .0000 -1.9158 -1.1010

2.6500 -1.4366 .2123 -6.7678 .0000 -1.8551 -1.0181

2.8000 -1.3647 .2241 -6.0909 .0000 -1.8065 -.9230

2.9500 -1.2929 .2411 -5.3618 .0000 -1.7683 -.8175

3.1000 -1.2210 .2624 -4.6525 .0000 -1.7385 -.7036

3.2500 -1.1492 .2871 -4.0033 .0001 -1.7152 -.5832

3.4000 -1.0773 .3142 -3.4287 .0007 -1.6968 -.4579

3.5500 -1.0055 .3433 -2.9291 .0038 -1.6823 -.3287

3.7000 -.9337 .3738 -2.4975 .0133 -1.6707 -.1966

3.8500 -.8618 .4055 -2.1251 .0348 -1.6614 -.0623

3.9189 -.8288 .4204 -1.9715 .0500 -1.6577 .0000

4.0000 -.7900 .4381 -1.8030 .0728 -1.6538 .0738

Data for visualizing the conditional effect of the focal predictor:

Paste text below into a SPSS syntax window and execute to produce plot.

DATA LIST FREE/

Stigma_I copVenti FACT_G .

BEGIN DATA.

7.2796 1.5000 77.8246

11.2836 1.5000 69.8672

17.3587 1.5000 57.7937

7.2796 2.5000 76.1468

11.2836 2.5000 70.1071

17.3587 2.5000 60.9434

7.2796 3.5000 74.4690

11.2836 3.5000 70.3471

17.3587 3.5000 64.0931

END DATA.

GRAPH/SCATTERPLOT=

Stigma_I WITH FACT_G BY copVenti .

*********************** ANALYSIS NOTES AND ERRORS ************************

Level of confidence for all confidence intervals in output:

95.0000

**************************************************************************

Model : 1

Y : FACT_B

X : Stigma_I

W : Comprehe

Covariates:

Age_cov Educatio Employme Social_p Insuranc Major_Ps Chemo_co Mastecto Radiothe

Sample

Size: 221

**************************************************************************

OUTCOME VARIABLE:

FACT_B

Model Summary

R R-sq MSE F df1 df2 p

.5937 .3525 273.1338 9.4374 12.0000 208.0000 .0000

Model

coeff se t p LLCI ULCI

constant 98.7785 9.5780 10.3131 .0000 79.8962 117.6608

Stigma_I -1.8448 .2480 -7.4391 .0000 -2.3337 -1.3559

Comprehe .4983 .1890 2.6361 .0090 .1256 .8709

Int_1 -.0728 .0365 -1.9955 .0473 -.1447 -.0009

Age_cov -.1021 .1268 -.8049 .4218 -.3520 .1479

Educatio -5.4191 3.3845 -1.6012 .1109 -12.0913 1.2532

Employme -5.1614 3.6014 -1.4332 .1533 -12.2614 1.9385

Social_p 1.6400 .7926 2.0691 .0398 .0774 3.2026

Insuranc 5.3477 3.9810 1.3433 .1806 -2.5006 13.1960

Major_Ps -5.3129 2.7030 -1.9655 .0507 -10.6417 .0159

Chemo_co -1.6302 1.7047 -.9563 .3400 -4.9908 1.7305

Mastecto -.9068 1.6138 -.5619 .5748 -4.0882 2.2747

Radiothe -.3099 2.9007 -.1068 .9150 -6.0284 5.4086

| **Benjamini-Hochberg Procedure** | | | | |
| --- | --- | --- | --- | --- |
|  |  | **Ascending P-value s** | **I = ranking** | **(I/12)* 0.20** |
|  | **Stigma** | **.0000** | **1** | **.0166** |
|  | **Comprehensibility** | **.0090** | **2** | **.033** |
|  | **Social participation frequency** | **.0398** | **3** | **.050** |
|  | **Interaction Effect** | **.0473** | **4** | **.066** |
|  | **Major psychological problems** | **.0507** | **5** | **.0833** |
|  | **Education** | **~~.1109~~** | **6** | **.10** |
|  | **Employment** | **~~.1533~~** | **7** | **.116** |
|  | **Insurance** | **~~.1806~~** | **8** | **.133** |
|  | **Chemotherapy** | **~~.3400~~** | **9** | **.15** |
|  | **Age** | **~~.4218~~** | **10** | **.166** |
|  | **Mastectomy** | **~~.5748~~** | **11** | **.183** |
|  | **Radiotherapy** | **~~.9150~~** | **12** | **.20** |

Product terms key:

Int_1 : Stigma_I x Comprehe

Test(s) of highest order unconditional interaction(s):

R2-chng F df1 df2 p

X*W .0124 3.9819 1.0000 208.0000 .0473

----------

Focal predict: Stigma_I (X)

Mod var: Comprehe (W)

Conditional effects of the focal predictor at values of the moderator(s):

Comprehe Effect se t p LLCI ULCI

13.5200 -1.3414 .3270 -4.1022 .0001 -1.9861 -.6968

21.0000 -1.8860 .2518 -7.4894 .0000 -2.3825 -1.3896

27.0000 -2.3228 .3690 -6.2949 .0000 -3.0503 -1.5954

Moderator value(s) defining Johnson-Neyman significance region(s):

Value % below % above

8.1131 3.6199 96.3801

Conditional effect of focal predictor at values of the moderator:

Comprehe Effect se t p LLCI ULCI

5.0000 -.7212 .5813 -1.2405 .2162 -1.8672 .4249

6.5000 -.8304 .5322 -1.5602 .1202 -1.8796 .2189

8.0000 -.9396 .4843 -1.9400 .0537 -1.8944 .0152

8.1131 -.9478 .4808 -1.9714 .0500 -1.8956 .0000

9.5000 -1.0488 .4380 -2.3944 .0175 -1.9123 -.1853

11.0000 -1.1580 .3939 -2.9400 .0037 -1.9345 -.3815

12.5000 -1.2672 .3527 -3.5926 .0004 -1.9625 -.5718

14.0000 -1.3764 .3157 -4.3597 .0000 -1.9988 -.7540

15.5000 -1.4856 .2845 -5.2225 .0000 -2.0464 -.9248

17.0000 -1.5948 .2611 -6.1086 .0000 -2.1095 -1.0801

18.5000 -1.7040 .2478 -6.8774 .0000 -2.1925 -1.2155

20.0000 -1.8132 .2462 -7.3652 .0000 -2.2985 -1.3279

21.5000 -1.9224 .2565 -7.4934 .0000 -2.4282 -1.4166

23.0000 -2.0316 .2775 -7.3207 .0000 -2.5787 -1.4845

24.5000 -2.1408 .3069 -6.9750 .0000 -2.7459 -1.5357

26.0000 -2.2500 .3426 -6.5672 .0000 -2.9255 -1.5746

27.5000 -2.3592 .3828 -6.1627 .0000 -3.1139 -1.6045

29.0000 -2.4684 .4263 -5.7907 .0000 -3.3088 -1.6281

30.5000 -2.5776 .4721 -5.4602 .0000 -3.5083 -1.6470

32.0000 -2.6868 .5196 -5.1709 .0000 -3.7112 -1.6625

33.5000 -2.7960 .5684 -4.9188 .0000 -3.9167 -1.6754

35.0000 -2.9052 .6183 -4.6991 .0000 -4.1241 -1.6864

Data for visualizing the conditional effect of the focal predictor:

Paste text below into a SPSS syntax window and execute to produce plot.

DATA LIST FREE/

Stigma_I Comprehe FACT_B .

BEGIN DATA.

7.2796 13.5200 92.9076

11.2836 13.5200 87.5365

17.3587 13.5200 79.3871

7.2796 21.0000 99.2273

11.2836 21.0000 91.6757

17.3587 21.0000 80.2181

7.2796 27.0000 104.2965

11.2836 27.0000 94.9960

17.3587 27.0000 80.8847

END DATA.

GRAPH/SCATTERPLOT=

Stigma_I WITH FACT_B BY Comprehe .

*********************** ANALYSIS NOTES AND ERRORS ************************

Level of confidence for all confidence intervals in output:

95.0000

W values in conditional tables are the 16th, 50th, and 84th percentiles.

**************************************************************************

Model : 1

Y : FACT_B

X : Stigma_I

W : copBehav

Covariates:

Age_cov Educatio Employme Social_p Insuranc Major_Ps Chemo_co Mastecto Radiothe

Sample

Size: 219

**************************************************************************

OUTCOME VARIABLE:

FACT_B

Model Summary

R R-sq MSE F df1 df2 p

.5751 .3308 283.8437 8.4855 12.0000 206.0000 .0000

Model

coeff se t p LLCI ULCI

constant 97.8671 9.8221 9.9639 .0000 78.5022 117.2319

Stigma_I -2.0223 .2557 -7.9099 .0000 -2.5263 -1.5182

copBehav -7.4632 9.5086 -.7849 .4334 -26.2098 11.2835

Int_1 3.5258 1.7798 1.9810 .0489 .0168 7.0348

Age_cov -.0182 .1308 -.1388 .8897 -.2761 .2398

Educatio -5.4839 3.4540 -1.5877 .1139 -12.2936 1.3258

Employme -4.3490 3.6921 -1.1779 .2402 -11.6281 2.9302

Social_p 1.8333 .8110 2.2606 .0248 .2344 3.4321

Insuranc 4.9755 4.0579 1.2261 .2215 -3.0247 12.9758

Major_Ps -6.6113 2.7517 -2.4026 .0172 -12.0364 -1.1862

Chemo_co -2.0292 1.7463 -1.1620 .2466 -5.4721 1.4136

Mastecto -1.4746 1.6487 -.8944 .3721 -4.7251 1.7759

Radiothe -.9736 2.9514 -.3299 .7418 -6.7925 4.8452

| **Benjamini-Hochberg Procedure** | | | | |
| --- | --- | --- | --- | --- |
|  |  | **Ascending P-value s** | **I = ranking** | **(I/12)* 0.20** |
|  | **Stigma** | **.0000** | **1** | **.0166** |
|  | **Major psychological problems** | **.0172** | **2** | **.033** |
|  | **Social participation frequency** | **.0248** | **3** | **.050** |
|  | **Interaction Effect** | **.0489** | **4** | **.066** |
|  | **Education** | **~~.1139~~** | **5** | **.0833** |
|  | **Insurance** | **~~.2215~~** | **6** | **.10** |
|  | **Employment** | **~~.2402~~** | **7** | **.116** |
|  | **Chemotherapy** | **~~.2466~~** | **8** | **.133** |
|  | **Mastectomy** | **~~.3721~~** | **9** | **.15** |
|  | **Behavioral disengagement** | **~~.4334~~** | **10** | **.166** |
|  | **Radiotherapy** | **~~.7418~~** | **11** | **.183** |
|  | **Age** | **~~.8897~~** | **12** | **.20** |

Product terms key:

Int_1 : Stigma_I x copBehav

Test(s) of highest order unconditional interaction(s):

R2-chng F df1 df2 p

X*W .0127 3.9244 1.0000 206.0000 .0489

----------

Focal predict: Stigma_I (X)

Mod var: copBehav (W)

Conditional effects of the focal predictor at values of the moderator(s):

copBehav Effect se t p LLCI ULCI

.4061 -2.3996 .3438 -6.9797 .0000 -3.0775 -1.7218

.4061 -2.3996 .3438 -6.9797 .0000 -3.0775 -1.7218

.6802 -1.4330 .3578 -4.0056 .0001 -2.1384 -.7277

Moderator value(s) defining Johnson-Neyman significance region(s):

Value % below % above

.7946 95.8904 4.1096

Conditional effect of focal predictor at values of the moderator:

copBehav Effect se t p LLCI ULCI

.4061 -2.3996 .3438 -6.9797 .0000 -3.0775 -1.7218

.4293 -2.3175 .3171 -7.3094 .0000 -2.9426 -1.6924

.4526 -2.2354 .2938 -7.6099 .0000 -2.8146 -1.6563

.4759 -2.1533 .2748 -7.8373 .0000 -2.6950 -1.6117

.4992 -2.0712 .2610 -7.9357 .0000 -2.5858 -1.5567

.5225 -1.9891 .2534 -7.8511 .0000 -2.4887 -1.4896

.5458 -1.9070 .2524 -7.5564 .0000 -2.4046 -1.4095

.5691 -1.8249 .2581 -7.0698 .0000 -2.3339 -1.3160

.5923 -1.7428 .2702 -6.4504 .0000 -2.2755 -1.2101

.6156 -1.6607 .2878 -5.7711 .0000 -2.2281 -1.0934

.6389 -1.5786 .3099 -5.0936 .0000 -2.1897 -.9676

.6622 -1.4966 .3358 -4.4572 .0000 -2.1585 -.8346

.6855 -1.4145 .3645 -3.8807 .0001 -2.1330 -.6959

.7088 -1.3324 .3955 -3.3690 .0009 -2.1120 -.5527

.7320 -1.2503 .4282 -2.9196 .0039 -2.0945 -.4060

.7553 -1.1682 .4624 -2.5264 .0123 -2.0798 -.2565

.7786 -1.0861 .4976 -2.1824 .0302 -2.0672 -.1049

.7946 -1.0298 .5223 -1.9715 .0500 -2.0596 .0000

.8019 -1.0040 .5338 -1.8808 .0614 -2.0564 .0485

.8252 -.9219 .5707 -1.6154 .1078 -2.0470 .2032

.8485 -.8398 .6081 -1.3809 .1688 -2.0387 .3592

.8718 -.7577 .6461 -1.1727 .2423 -2.0314 .5161

Data for visualizing the conditional effect of the focal predictor:

Paste text below into a SPSS syntax window and execute to produce plot.

DATA LIST FREE/

Stigma_I copBehav FACT_B .

BEGIN DATA.

7.2796 .4061 102.3142

11.2836 .4061 92.7061

17.3587 .4061 78.1281

7.2796 .4061 102.3142

11.2836 .4061 92.7061

17.3587 .4061 78.1281

7.2796 .6802 95.6766

11.2836 .6802 89.9387

17.3587 .6802 81.2329

END DATA.

GRAPH/SCATTERPLOT=

Stigma_I WITH FACT_B BY copBehav .

*********************** ANALYSIS NOTES AND ERRORS ************************

Level of confidence for all confidence intervals in output:

95.0000

W values in conditional tables are the 16th, 50th, and 84th percentiles

**************************************************************************

Model : 1

Y : FACT_B

X : Stigma_I

W : copVenti

Covariates:

Age_cov Educatio Employme Social_p Insuranc Major_Ps Chemo_co Mastecto Radiothe

Sample

Size: 219

**************************************************************************

OUTCOME VARIABLE:

FACT_B

Model Summary

R R-sq MSE F df1 df2 p

.5715 .3266 285.6225 8.3258 12.0000 206.0000 .0000

Model

coeff se t p LLCI ULCI

constant 100.0416 9.8050 10.2032 .0000 80.7107 119.3725

Stigma_I -2.0106 .2475 -8.1244 .0000 -2.4985 -1.5227

copVenti .4493 1.3442 .3342 .7386 -2.2010 3.0995

Int_1 .5050 .2981 1.6938 .0918 -.0828 1.0927

Age_cov -.0313 .1309 -.2388 .8115 -.2893 .2268

Educatio -5.1907 3.4698 -1.4959 .1362 -12.0316 1.6503

Employme -4.4658 3.6859 -1.2116 .2271 -11.7328 2.8011

Social_p 1.7233 .8144 2.1162 .0355 .1178 3.3289

Insuranc 4.6638 4.0724 1.1452 .2534 -3.3651 12.6928

Major_Ps -6.6121 2.7586 -2.3969 .0174 -12.0509 -1.1734

Chemo_co -2.5975 1.7340 -1.4980 .1357 -6.0162 .8212

Mastecto -1.5654 1.6302 -.9603 .3380 -4.7794 1.6485

Radiothe -.8158 2.9737 -.2743 .7841 -6.6787 5.0471

| **Benjamini-Hochberg Procedure** | | | | |
| --- | --- | --- | --- | --- |
|  |  | **Ascending P-value s** | **I = ranking** | **(I/12)* 0.20** |
|  | **Stigma** | **.0000** | **1** | **.0166** |
|  | **Major psychological problems** | **.0174** | **2** | **.033** |
|  | **Social participation frequency** | **.0355** | **3** | **.050** |
|  | **Interaction Effect** | **~~.0918~~** | **4** | **.066** |
|  | **Chemotherapy** | **~~.1357~~** | **5** | **.0833** |
|  | **Education** | **~~.1362~~** | **6** | **.10** |
|  | **Employment** | **~~.2271~~** | **7** | **.116** |
|  | **Insurance** | **~~.2534~~** | **8** | **.133** |
|  | **Mastectomy** | **~~.3380~~** | **9** | **.15** |
|  | **Venting** | **~~.7386~~** | **10** | **.166** |
|  | **Radiotherapy** | **~~.7841~~** | **11** | **.183** |
|  | **Age** | **~~.8115~~** | **12** | **.20** |

Product terms key:

Int_1 : Stigma_I x copVenti

Test(s) of highest order unconditional interaction(s):

R2-chng F df1 df2 p

X*W .0094 2.8691 1.0000 206.0000 .0918

----------

Focal predict: Stigma_I (X)

Mod var: copVenti (W)

Conditional effects of the focal predictor at values of the moderator(s):

copVenti Effect se t p LLCI ULCI

1.5000 -2.4579 .3754 -6.5483 .0000 -3.1979 -1.7179

2.5000 -1.9530 .2472 -7.8990 .0000 -2.4404 -1.4655

3.5000 -1.4480 .3989 -3.6300 .0004 -2.2344 -.6615

There are no statistical significance transition points within the observed

range of the moderator found using the Johnson-Neyman method.

Conditional effect of focal predictor at values of the moderator:

copVenti Effect se t p LLCI ULCI

1.0000 -2.7104 .4974 -5.4489 .0000 -3.6911 -1.7297

1.1500 -2.6347 .4591 -5.7384 .0000 -3.5399 -1.7295

1.3000 -2.5589 .4221 -6.0623 .0000 -3.3911 -1.7267

1.4500 -2.4832 .3867 -6.4213 .0000 -3.2456 -1.7208

1.6000 -2.4074 .3534 -6.8116 .0000 -3.1042 -1.7106

1.7500 -2.3317 .3229 -7.2204 .0000 -2.9684 -1.6950

1.9000 -2.2559 .2961 -7.6198 .0000 -2.8396 -1.6722

2.0500 -2.1802 .2739 -7.9597 .0000 -2.7202 -1.6402

2.2000 -2.1044 .2577 -8.1673 .0000 -2.6124 -1.5964

2.3500 -2.0287 .2485 -8.1633 .0000 -2.5187 -1.5387

2.5000 -1.9530 .2472 -7.8990 .0000 -2.4404 -1.4655

2.6500 -1.8772 .2540 -7.3918 .0000 -2.3779 -1.3765

2.8000 -1.8015 .2681 -6.7201 .0000 -2.3300 -1.2730

2.9500 -1.7257 .2885 -5.9819 .0000 -2.2945 -1.1569

3.1000 -1.6500 .3140 -5.2548 .0000 -2.2690 -1.0309

3.2500 -1.5742 .3434 -4.5836 .0000 -2.2514 -.8971

3.4000 -1.4985 .3759 -3.9861 .0001 -2.2396 -.7573

3.5500 -1.4227 .4107 -3.4641 .0006 -2.2325 -.6130

3.7000 -1.3470 .4473 -3.0116 .0029 -2.2288 -.4652

3.8500 -1.2712 .4852 -2.6201 .0094 -2.2278 -.3147

4.0000 -1.1955 .5242 -2.2807 .0236 -2.2290 -.1620

Data for visualizing the conditional effect of the focal predictor:

Paste text below into a SPSS syntax window and execute to produce plot.

DATA LIST FREE/

Stigma_I copVenti FACT_B .

BEGIN DATA.

7.2796 1.5000 101.7263

11.2836 1.5000 91.8848

17.3587 1.5000 76.9528

7.2796 2.5000 99.7769

11.2836 2.5000 91.9573

17.3587 2.5000 80.0930

7.2796 3.5000 97.8274

11.2836 3.5000 92.0297

17.3587 3.5000 83.2331

END DATA.

GRAPH/SCATTERPLOT=

Stigma_I WITH FACT_B BY copVenti .

*********************** ANALYSIS NOTES AND ERRORS ************************

Level of confidence for all confidence intervals in output:

95.0000

W values in conditional tables are the 16th, 50th, and 84th percentiles.
